# Midline incisional hernia guidelines: the European Hernia Society

**DOI:** 10.1093/bjs/znad284

**Published:** 2023-09-19

**Authors:** David L Sanders, Maciej M Pawlak, Maarten P Simons, Theo Aufenacker, Andrea Balla, Cigdem Berger, Frederik Berrevoet, Andrew C de Beaux, Barbora East, Nadia A Henriksen, Miloslav Klugar, Alena Langaufová, Marc Miserez, Salvador Morales-Conde, Agneta Montgomery, Patrik K Pettersson, Wolfgang Reinpold, Yohann Renard, Simona Slezáková, Thomas Whitehead-Clarke, Cesare Stabilini

**Affiliations:** Academic Department of Abdominal Wall Surgery, Royal Devon University Foundation Healthcare Trust, North Devon District Hospital, Barnstaple, UK; University of Exeter Medical School, Exeter, UK; Academic Department of Abdominal Wall Surgery, Royal Devon University Foundation Healthcare Trust, North Devon District Hospital, Barnstaple, UK; University of Exeter Medical School, Exeter, UK; Department of Surgery, OLVG Hospital Amsterdam, Amsterdam, The Netherlands; Department of Surgery, Rijnstate Hospital Arnhem, Arnhem, The Netherlands; IRCCS San Raffaele Scientific Institute, Milan, Italy; Hamburg Hernia Centre, Department of Hernia and Abdominal Wall Surgery, Helios Mariahilf Hospital Hamburg, Teaching Hospital of the University of Hamburg, Hamburg, Germany; Department for General and HPB Surgery and Liver Transplantation, Ghent University Hospital, Ghent, Belgium; Department of Surgery, Spire Murrayfield Hospital, Edinburgh, UK; 3rd Department of Surgery at 1st Medical Faculty of Charles University, Motol University Hospital, Prague, Czech Republic; Department of Gastrointestinal and Hepatic Diseases, University of Copenhagen, Herlev Hospital, Copenhagen, Denmark; The Czech National Centre for Evidence-Based Healthcare and Knowledge Translation (Cochrane Czech Republic, Czech CEBHC: JBI Centre of Excellence, Masaryk University GRADE Centre), Institute of Biostatistics and Analyses, Faculty of Medicine, Masaryk University, Brno, Czech Republic; Department of Health Sciences, Faculty of Medicine, Masaryk University, Brno, Czech Republic; Department of Abdominal Surgery, University Hospital Gasthuisberg, KU Leuven, Leuven, Belgium; Unit of Innovation in Minimally Invasive Surgery, Department of General and Digestive Surgery, University Hospital Virgen del Rocio, University of Sevilla, Sevilla, Spain; Department of Surgery, Skåne University Hospital, Malmö, Sweden; Department of Clinical Sciences, Malmö Faculty of Medicine, Lund University, Lund, Sweden; Department of Surgery, Skåne University Hospital, Malmö, Sweden; Department of Clinical Sciences, Malmö Faculty of Medicine, Lund University, Lund, Sweden; Hamburg Hernia Centre, Department of Hernia and Abdominal Wall Surgery, Helios Mariahilf Hospital Hamburg, Teaching Hospital of the University of Hamburg, Hamburg, Germany; Reims Champagne-Ardennes, Department of General, Digestive and Endocrine Surgery, Robert Debré University Hospital, Reims, France; The Czech National Centre for Evidence-Based Healthcare and Knowledge Translation (Cochrane Czech Republic, Czech CEBHC: JBI Centre of Excellence, Masaryk University GRADE Centre), Institute of Biostatistics and Analyses, Faculty of Medicine, Masaryk University, Brno, Czech Republic; Centre for 3D Models of Health and Disease, Division of Surgery and Interventional Science, University College London, London, UK; Department of Surgery, University of Genoa, Genoa, Italy; Policlinico San Martino, IRCCS, Genoa, Italy

## Introduction

Since the introduction of anaesthesia by Morton in 1846, and as survivable abdominal surgery became more common, so did the incidence of incisional hernias. Since then, more than 4000 peer-reviewed articles have been published on the topic, many of which have tried to reduce the incidence or introduce techniques to improve outcomes from surgical repair. Despite this, the incidence of incisional hernias and the recurrence rates after repair remain high. A wide range of incisional hernia rates are reported^1–5^. A meta-analysis including over 14 000 patients reported a weighted incidence of 12.8 per cent 2 years after a midline incision, and that one-third of patients with an incisional hernia undergo surgical repair^6^. Recurrence rates after repair of incisional hernia range between 23 and 50 per cent, with increasing rates of complications and re-recurrence after each subsequent failed repair^7^. Arguably, no other benign disease has seen so little improvement in terms of surgical outcome.

The Society of American Gastrointestinal Endoscopic Surgeons (SAGES) published guidelines on laparoscopic ventral hernia repair (which included incisional hernia) in 2016^8^. An expert-guided consensus for the management of all types of ventral hernias exists^9^, and the World Society of Emergency Surgery (WSES) addressed emergency repairs of both primary ventral and incisional hernias^10^. Similarly, the International EndoHernia Society (IEHS) published guidelines on the laparoscopic repair of both primary ventral and incisional hernias in 2014^11^ and updated these in 2019^12^. However, to date, no guidelines have been published exclusively focusing on the treatment of incisional hernias.

The focus of debate about incisional hernias is often about the more complex end of the spectrum, including large incisional hernias requiring a component separation or hernias occurring in incisions that are close to bony prominences (for example subcostal or flank hernias). Whilst these are important topics and certainly of interest, the authors wanted to focus these guidelines on the assessment and treatment of the most common incisional hernias faced by general surgeons and in primary care, and where the greatest body of evidence was likely to lie to be able to produce robust guideline recommendations. Therefore, these guidelines focus on midline incisional hernias in adult patients where it is anticipated that the fascial defect could be closed without performing an advanced technique such as a component separation, or any other adjunctive technique facilitating myofascial closure.

## Methods

### Guidelines group

The incisional hernia guidelines project was approved by the European Hernia Society (EHS) board in July 2019. Two coordinators were appointed to manage the project. To ensure robust methodological support a Cochrane and grading of recommendations, assessment, development, and evaluation (GRADE) methodology team from the Czech National Centre for Evidence-Based Healthcare at Masaryk University was included in the guidelines group. The guidelines group was selected by the coordinators from the membership of the EHS and included general surgeons from various sub-specialties and specialist abdominal wall surgeons. A patient representative was invited to all group meetings, and was involved in prioritizing outcome parameters. Conflicts of interest for each member were recorded transparently at the beginning of the project. The meetings were funded by the EHS and the *British Journal of Surgery* (*BJS*). The EHS and the *BJS* had no influence on the content of the guidelines. There was no involvement from industry.

### Timeline and meetings

The protocol, including key questions (KQs) and timeline, was approved by the 19 participants at an introductory meeting for the guidelines held in London in February 2020. A further virtual meeting with a focus on GRADE methodology was held in November 2020 and there was a face-to-face meeting in Prague in May 2022 that focused on the outcomes for each KQ and gathering of expert evidence where required. All guidelines group members participated in a minimum of two of the three meetings.

### Methodology

This guideline follows GRADE methodology^13,14^. The guidelines group determined the scope of the clinical KQs. For each KQ, the relevant population, intervention, and outcome based on the (PICO, Patient, Intervention, Comparison, Outcome) concept were decided. The individual outcomes were rated by the expert panel on a scale of 1–9 based on their importance (critical, 9–7; important, 6–4; and of limited importance, 3–1); final agreement on the outcome rating was reached by consensus. Outcomes of limited importance were excluded.

#### Eligibility criteria

Eligibility criteria for inclusion in the guidelines were adult (greater than 18 years) patients with a primary incisional hernia; with a no larger than 10 cm fascial defect.

#### Literature searches

The preferred study designs to answer KQs were systematic reviews and RCTs. If the KQ was not answered by experimental designs (randomized, quasi, and pseudo-controlled trials) and systematic reviews, the selection criteria for studies was expanded to include analytical observational studies (cohort, case–control, and analytical cross-sectional studies).

Systematic literature searches were carried out to find all clinical and health evidence relevant to the guideline KQs. During the scoping stage in July 2020, guideline repositories and databases (GIN (Guidlines International Network), BIGG (International Database for Grade Guidelines), Epistemonikos GRADE Guidelines Repository, ECRI (Emergency Care Research Institute) Guidelines Trust, and MAGICapp (MAGIC authoring and publication platform (MAGICapp) – for guidelines and evidence summaries)), websites of guideline developers (NICE (The National Institute for Health and Care Excellence), SIGN (Scottish Intercollegiate Guidelines Network), AWMF (Institut für Medizinisches Wissensmanagement), and GuíaSalud), and hernia society websites (EHS, Americas Hernia Society, and British Hernia Society) were searched for guidelines on incisional hernias as per the GRADE framework^14^. As no relevant guidelines were identified, the authors proceeded with a search in the database Epistemonikos to retrieve systematic reviews on incisional hernias.

Where systematic reviews either only partially answered a KQ or did not answer it, the search strategies were newly designed using relevant index terms and free-text terms. Study-type filters for controlled clinical trials, systematic reviews, case–control studies, and cohort studies developed by Canadian Agency for Drugs and Technologies in Health (CADTH)^15^ or the Health Science Center at Houston, The University of Texas^16^ were applied in all searches. Limits were applied to only include human studies and exclude non-relevant publication types such as historical articles, letters, editorials, and conference abstracts. The following databases were searched with limitation to English written records up to March 2021: MEDLINE (Ovid), Embase (Ovid), and the Cochrane Library. Reference lists of relevant studies were screened additionally to identify further studies meeting the eligibility criteria. For the complete identification of relevant evidence, handsearching was also performed.

The search results for each KQ were de-duplicated in EndNote X9.2 (Clarivate Analytics) using the method described by Bramer *et al.*^17^.

#### Study selection

Documents were uploaded to Rayyan^18^ and sorted according to their publication type determined by the search filters. First, titles/abstracts of controlled clinical trials and systematic reviews were screened and, from these, relevant full texts were screened for eligibility. Screening was performed independently by at least two surgeons responsible for the KQ. A third reviewer (D.L.S. or A.C.d.B if D.L.S was a primary reviewer) was used in the case of discrepancies between two reviewers (KQ1, D.L.S., and M.M.P.; KQ2, T.W.-C. and A.M.; KQ3, D.L.S. and T.W.-C.; KQ4, C.B. and A.C.d.B.; KQ5, F.B. and P.K.P.; KQ6, P.K.P., N.A.H., and F.B.; KQ7, A.C.d.B., M.P.S., and Y.R.; KQ8, N.A.H., W.R., C.B., and N.A.H.; KQ9, A.M., B.E., and T.A.; KQ10, C.S., M.P.S., and S.M.-C.; KQ11, A.C.d.B. and B.E.; KQ12, S.M.-C. and A.C.d.B.; KQ13, T.A. and M.M.; KQ14, Y.R., A.E., and C.S.; and KQ15, M.M. and A.C.d.B.). Titles/abstracts and full texts of case–control and cohort studies were only screened (using the same process as described above) if insufficient evidence was found in controlled clinical trials and systematic reviews.

#### Data extraction and quality assessment

The quality assessment was conducted independently by two methodologists (A.L. and S.S.). RCTs were assessed using the Cochrane risk-of-bias tool for randomized trials, Review Manager 5.4. The quality assessment of studies with different designs was performed using the Joanna Briggs Institute (JBI) critical appraisal tools. A third methodologist (M.K.) assisted with conflicting decisions.

Data from included studies were extracted independently by two methodologists (A.L. and S.S.). This included study details (author name, year, and follow-up) and population characteristics (age, sex, BMI, and other available patient characteristics). The extracted data obtained for interventions, comparisons, and outcomes correspond to the specific KQ.

#### Data synthesis and analysis

Quantitative data were pooled in statistical meta-analyses using Cochrane Review Manager 5.4, where possible. Where statistical pooling was not possible, synthesis without meta-analyses was performed. When the direct scientific evidence was missing for some outcomes, expert evidence was extracted in alignment with the GRADE framework^19,20^ using expert evidence forms for each content expert within the guidelines panel^21^.

Pooled ORs (for dichotomous data) and weighted mean differences (for continuous data) and their 95 per cent confidence intervals (c.i.) were calculated. For one KQ (KQ2), diagnostic accuracy and overall accuracy by summary receiver operating characteristics (SROC) was calculated. Sensitivity analyses were performed for every result where possible based on the number of included studies and differences in the risk of bias or indirectness.

Random- or fixed-effects meta-analyses were used to obtain methodologically sound results for pooling according to the number of included studies and the size of the included body of evidence^22,23^. Heterogeneity was evaluated using Cochrane chi-squared and *I*^2^ tests. Cochrane chi-squared value *P* < 0.100 and *I*^2^ statistics greater than or equal to 50 per cent show important heterogeneity.

#### Certainty of evidence

The certainty of the evidence was assessed by grading of recommendation, which was performed by a lead methodologist (M.K.) in consultation with lead surgeons for each KQ in all eight domains of GRADE.

Summary of Findings tables were created using the GRADEpro GDT tool. The overall certainty of the evidence was rated for each outcome as^24–27^: high (confidence that the true effect is similar to the estimated effect); moderate (true effect is probably close to the estimated effect); low (true effect might be markedly different from the estimated effect); or very low (true effect is probably markedly different from the estimated effect).

#### Development of recommendations and reaching of consensus

The guidelines panel met at the face-to-face meeting in Prague in May 2022. The GRADE Summary of Findings tables for each KQ were presented with all supporting materials (meta-analyses, risk-of-bias assessment, and extraction tables). All parts of the GRADE Evidence to Decision frameworks were used in facilitating the process of formulating the recommendations (both formal recommendations and good practice statements). The consensus was reached by the iterative discussion of all panellists for each recommendation.

Moreover, to achieve the most robust consensus possible, the guideline leaders decided to present a summary of the evidence for each recommendation and a proposal for the wording of the specific recommendation at the EHS 2022 Annual International Congress in Manchester. The threshold for approval of the wording of the recommendation was preset at 66.6 per cent (two-thirds) approval of those present. If this consensus was not achieved the recommendation was re-evaluated and reworded taking into account the feedback from the discussion at the congress presentation. One KQ fell below this threshold and needed re-discussion/rewording with the guidelines group.

## Results

A total of 15 KQs were formulated that were further synthesized into 13 questions due to significant overlap after analysis was performed.

The results for each of these is presented below along with ‘recommendations’ if there was sufficient quality of evidence or a ‘good practice statement’ where the quality of evidence was insufficient to make a formal recommendation. The detailed search strategies for each KQ are shown in *Table S1*. For each KQ a detailed Summary of Findings table, which details the number of studies analysed, the certainty assessment (including risk of bias, inconsistency, indirectness, and imprecision), the number of patients with and without exposure, the relative and absolute effect size, and the certainty of evidence, is included in each section’s Summary of Findings Table.

**Key Question 1: What are the risk factors for developing an incisional hernia after previous abdominal surgery?**

**Good Practice Statement A:** Patients should be advised that high BMI, smoking, diabetes, and immunosuppression are risk factors for developing an incisional hernia after abdominal surgery.

**Good Practice Statement B:** Surgeons should be aware that midline incisions have a higher risk of incisional hernia than off-midline incisions.

**Good Practice Statement C:** Surgeons should be aware that single incision laparoscopic surgery (SILS), trocar sites 10 mm and larger, and umbilical site trocars have a higher risk of incisional hernia (trocar-site hernias).

**Good Practice Statement D:** Surgeons should be advised that the combination of a continuous small-bites suturing technique with a slowly absorbable suture reduces the risk of incisional hernia.

**Good Practice Statement E:** Surgeons should be aware that surgical site infection (SSI) after abdominal surgery is a risk factor for developing an incisional hernia and appears to have the biggest impact when compared with other risk factors.

It is important to be aware of potential modifiable risk factors so that patients can be pre-optimized where possible before elective abdominal surgery. In addition, in both emergency and elective settings, awareness of risk factors for incisional hernia may influence closure technique and the potential use of prophylactic mesh for high-risk patients.

### Search results

**Fig. 1:**
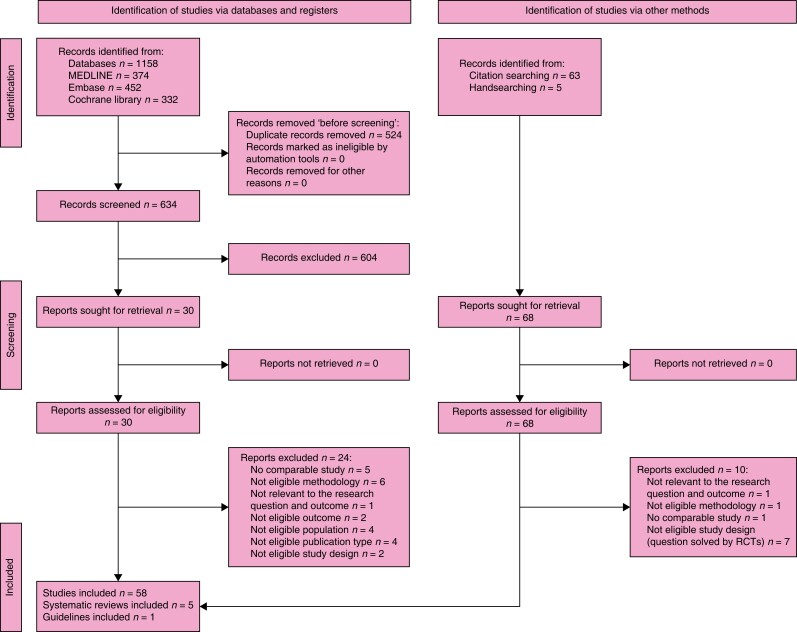
PRISMA flow diagram for Key Question 1

The search retrieved 1158 records. After the duplicates were removed, the titles and abstracts of 634 records were screened. A total of 30 reports were selected for full-text retrieval and were assessed for eligibility. A total of 24 reports were excluded. In total, three studies and three systematic reviews met the inclusion criteria. After checking the references of relevant publications and further handsearching, another 68 reports whose full texts were evaluated for eligibility were assessed. As a result, 55 studies, two systematic reviews, and one guideline and its recent update were included in the review. The full study selection process is presented in a PRISMA flow diagram (shown in *Fig. 1*). The Summary of Findings for KQ1 is shown in *Table S2*.

Follow-up for the studies varied considerably with mean follow-up ranging from 2 to 5.9 years. There was not enough evidence in the literature analysed to reliably report the effect of age or collagen disorders as independent risk factors.

### Evidence for Good Practice Statement A: patient-related risk factors

#### Diabetes

**Fig. 2:**
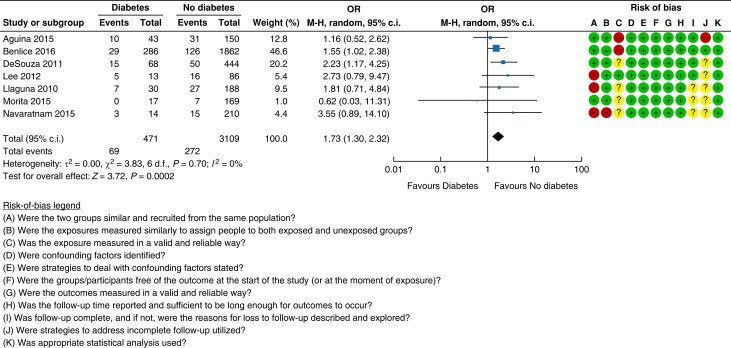
Forest plot: diabetes as a risk factor for incisional hernia

A total of seven cohort studies^28–34^ met the inclusion criteria for assessing diabetes as a risk factor for developing an incisional hernia. In the majority of studies this was included as a secondary outcome measure. The overall certainty of evidence was low. Pooled analysis revealed that the risk of incisional hernia in patients with diabetes was 14.6 per cent (69/471 patients) compared with 8.7 per cent (272/3109 patients) in the non-diabetic group (OR 1.73 (95 per cent c.i. 1.30 to 2.32)); see the forest plot and risk-of-bias assessment for included studies in *Fig. 2*. There was no differentiation in the studies between insulin- and non-insulin dependent diabetes or the level of diabetic control.

#### Obesity

In an observational cohort study with a low certainty of evidence, including 737 726 patients undergoing abdominal surgery, individuals with a BMI in the overweight or obese category (greater than or equal to 25 kg/m^2^) had an increased risk of incisional hernia (OR 95 per cent c.i. 1.7 to 5.5; *P* < 3.1 × 10^−20^)^35^. The risk increased proportionately with increasing BMI.

#### Smoking

**Fig. 3:**
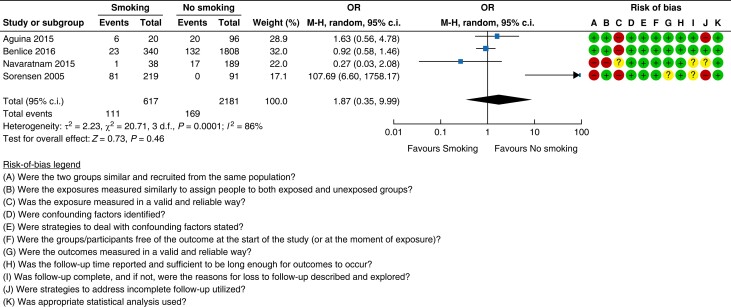
Forest plot: smoking as a risk factor for incisional hernia

A total of four cohort studies with a very low certainty of evidence assessed smoking as a risk factor for incisional hernia after abdominal surgery^28,29,34,36^. Pooled analysis revealed an 18 per cent (111/617 patients) risk of incisional hernia in smokers compared with a 7.7 per cent (169/2181 patients) risk in non-smokers and ex-smokers (OR 1.87 (95 per cent c.i. 1.36 to 2.57)). The forest plot and risk-of-bias assessment are shown in *Fig. 3*.

#### Immunosuppression

**Fig. 4:**
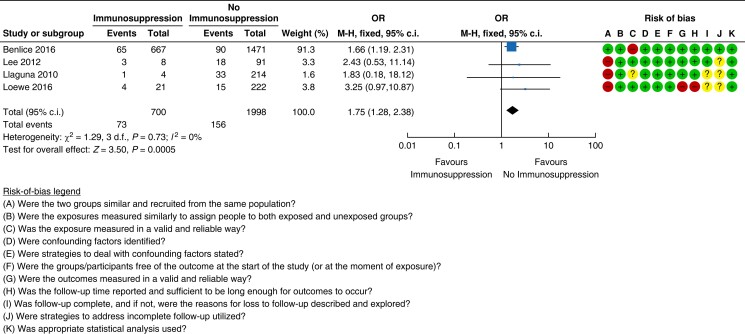
Forest plot: immunosuppression as a risk factor for incisional hernia

A total of four cohort studies with a very low certainty of evidence assessed immunosuppression as a risk factor for incisional hernia after abdominal surgery^29,31,32,37^. Pooled analysis from these studies revealed a 10.4 per cent (73/700 patients) risk of incisional hernia in immunosuppressed patients compared with a 7.8 per cent (156/1998 patients) risk in patients with no immunosuppression (OR 1.75 (95 per cent c.i. 1.28 to 2.38)) (see *Fig. 4*).

### Evidence for Good Practice Statements B, C, D, and E: surgery-related risk factors

#### Type of incision and closure

**Fig. 5:**
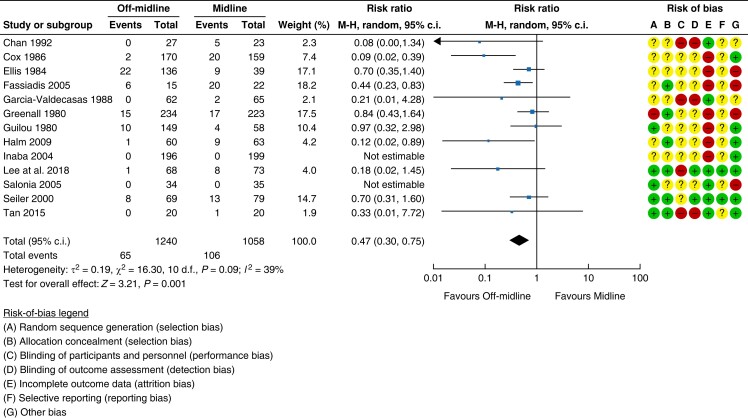
Forest plot: type of incision as a risk factor for incisional hernia

The type of abdominal incision is important in providing good access, especially in the emergency setting, but also in minimizing the risk of incisional hernia formation. The abdominal wall closure guidelines published in 2015 and updated in 2022 recommended transverse or paramedian incisions over midline incisions where possible to reduce the risk of incisional hernia^38,39^. However, there was no mention of potential nerve damage, leading to muscle degeneration. Source data from the RCTs included in the abdominal wall closure guidelines were reassessed using GRADE methodology. A total of 12 RCTs met the quality criteria for inclusion^40–52^ and one additional RCT^52^ was included that was published subsequent to the publication of the original guidelines. A total of nine studies compared transverse *versus* midline incisions and a total of four studies compared paramedian *versus* midline incisions. The overall certainty of evidence was low with significant heterogeneity both for type of incision and closure technique, and also in the method of detecting a hernia at follow-up (see risk-of-bias analysis in *Fig. 5*). Pooled data comparing off-midline (transverse and paramedian) *versus* midline incision with a median follow-up of 30 months were generated using a meta-analysis with a low certainty of evidence. The risk of an incisional hernia in the midline group was 10.0 per cent (106/1058 patients) compared with 5.2 per cent (65/1240 patients) in the off-midline group (Relative risk (RR) 0.47; 95 per cent c.i. 0.3 to 0.75); the forest plot is shown in *Fig. 5*.

The update of the abdominal closure guidelines recommends a continuous small-bites suturing technique with a slowly absorbable suture for the closure of elective midline incisions based on three RCTs published since the 2015 guidelines. The quality of evidence was low and the strength of recommendation was weak. Nevertheless, as a significant and important part of incisional hernia prevention, the authors of these guidelines decided to include a statement on abdominal wall closure as a surgical risk factor for developing an incisional hernia. For more information regarding optimal closing techniques and mesh augmentations, the authors refer readers to the full text of the updated guidelines for closure of abdominal wall incisions from the European and American Hernia Societies^39^.

#### Single incision laparoscopic surgery *versus* conventional laparoscopic surgery

**Fig. 6:**
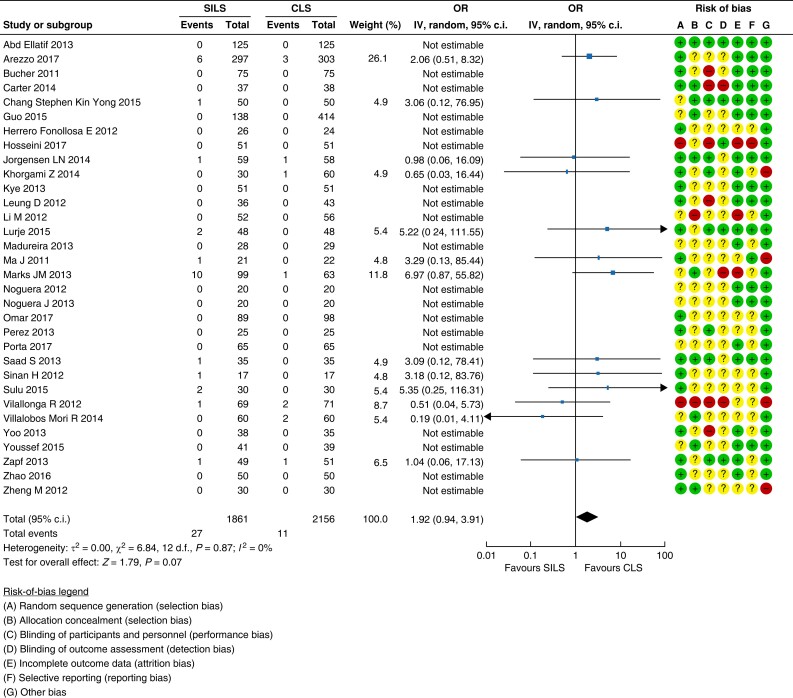
Forest plot: single incision laparoscopic surgery as a risk factor for incisional hernia SILS, single incision laparoscopic surgery; CLS, conventional laparoscopic surgery.

A total of 32 RCTs were identified that compared incisional hernia (port site hernia) after SILS *versus* conventional laparoscopic surgery^53–84^. The overall certainty of evidence was low. Pooled analysis revealed a risk of developing an incisional hernia of 1.5 per cent (27/1861 patients) with SILS *versus* 0.5 per cent (11/2156 patients) with conventional laparoscopic surgery (OR 1.92 (95 per cent c.i. 0.94 to 3.91)); see the forest plot and risk-of-bias assessment in *Fig. 6*.

#### Surgical site infection

**Fig. 7:**
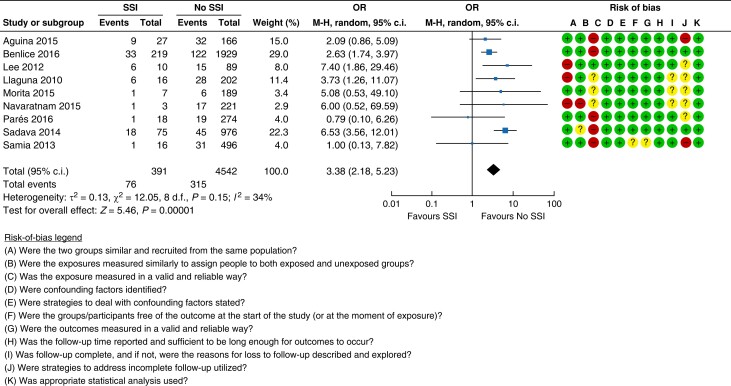
Forest plot: surgical site infection as a risk factor for incisional hernia SSI, surgical site infection.

It is well documented that SSI impairs wound healing. A total of nine studies assessed the impact of SSI as a risk factor for developing an incisional hernia^28,29,31–34,85–87^. Pooled analysis suggested a risk of incisional hernia of 19.4 per cent (76/391 patients) after having an SSI compared with 6.9 per cent (315/4542 patients) with no SSI (OR 3.38 (95 per cent c.i. 2.18 to 5.23)); see the forest plot and risk-of-bias assessment in *Fig. 7*.

There was no evidence for any other independent risk factors from the literature.

**Key Question 2: (a) Do all patients with an incisional hernia require imaging? and (b) What is the best modality?**

**Recommendation A:** For patients with a suspected incisional hernia where clinical examination has not given a definitive diagnosis, medical imaging to establish the diagnosis is suggested; from the evidence CT is the most sensitive investigation.

However, if the cost and radiation exposure are a concern then ultrasonography or MRI with Valsalva is suggested (conditional recommendation, low certainty evidence).

**Good Practice Statement B**: For patients with an incisional hernia (where surgery is being considered), the guidelines panel recommends using CT or MRI for preoperative planning.

Medical imaging is frequently used before surgery to characterize incisional hernias. Medical imaging may also play an important role in diagnosis where the presence of a hernia is not obvious on clinical examination.

### Search results

**Fig. 8:**
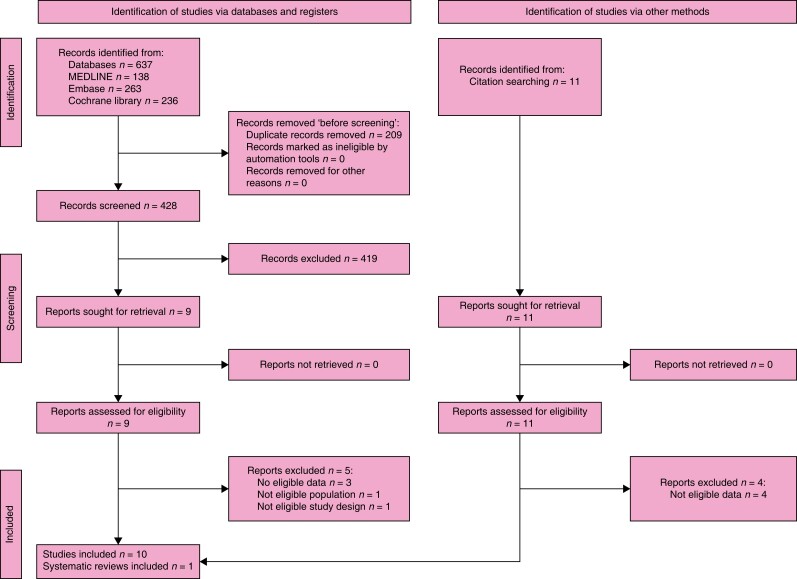
PRISMA flow diagram for Key Question 2

The search retrieved 637 records. After the duplicates were removed, the titles and abstracts of 428 records were screened. A total of nine were selected for full-text retrieval and were assessed for eligibility. A total of five were excluded and a total of three studies and one systematic review met the inclusion criteria. Checking the references of relevant publications identified a further 11 publications whose full texts were evaluated for eligibility and seven of these studies met the inclusion criteria. The full study selection process is presented in a PRISMA flow diagram (shown in *Fig. 8*). The Summary of Findings for KQ2 is shown in *Table S3*.

### Evidence for Recommendation A: diagnostic accuracy of examination comparing different imaging techniques for incisional hernia

#### Ultrasound *versus* physical examination

**Fig. 9:**
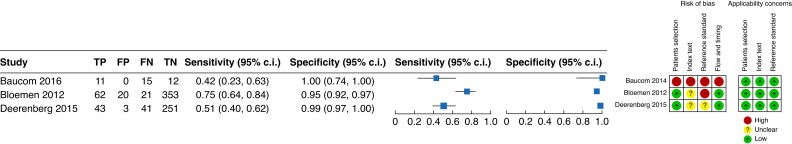
Forest plot: diagnostic accuracy of physical examination compared with ultrasound TP, true positives; FP, false positives; FN, false negatives; TN, true negatives.

A total of three cross-sectional studies^1,88,89^ were included for this analysis. They included a total of 832 patients. Using ultrasound as a reference standard, physical examination alone was found to have a sensitivity between 0.42 and 0.75, and a specificity between 0.95 and 1.00. The forest plot and risk-of-bias assessment for these studies are shown in *Fig. 9*.

#### CT *versus* physical examination

**Fig. 10:**
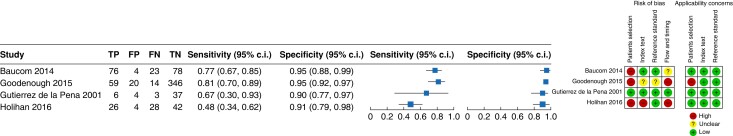
Forest plot: diagnostic accuracy of physical examination compared with CT TP, true positives; FP, false positives; FN, false negatives; TN, true negatives.

A total of four cross-sectional studies^90–93^ were extracted from the Kroese *et al*.^94^ systematic review that directly compared CT with physical examination. When compared with CT, physical examination was found to have a sensitivity between 0.48 and 0.81, and a specificity between 0.9 and 0.95. *Figure 10* shows the forest plot and risk-of-bias assessment for these studies.

#### Physical examination *versus* intraoperative findings

A total of two studies^91,92^ were included that directly assessed the accuracy of physical examination against intraoperative findings. The first study, of 50 patients, reported a sensitivity of 0.75 (95 per cent c.i. 0.35 to 0.97) and a specificity of 0.9 (95 per cent c.i. 0.77 to 0.97).^92^ The second study, a smaller study by Holihan *et al*.^91^, reported a sensitivity of 0.79 (95 per cent c.i. 0.49 to 0.95) and specificity of 0.75 (95 per cent c.i. 0.19 to 0.99).

#### CT *versus* intraoperative findings

A total of three cross-sectional studies^91,92,95^ assessed the accuracy of CT compared with intraoperative findings; two small studies of only 12–18 patients and one larger study of 50 patients were included. The largest of the three studies described a sensitivity for CT of 1.0 (95 per cent c.i. 0.63 to 1.0) and a specificity of 0.98 (95 per cent c.i. 0.87 to 1.0).

#### CT *versus* ultrasound

A total of two cross-sectional studies^3,96^ directly compared ultrasound and CT imaging for incisional hernia diagnosis; 40 and 181 patients were included respectively. In the larger study, by Beck *et al.*^96^, ultrasound was found to have a sensitivity of 0.98 (95 per cent c.i. 0.93 to 1.0) and a specificity of 0.88 (95 per cent c.i. 0.79 to 0.94) when compared with CT. den Hartog *et al.*^3^ showed ultrasound having a sensitivity of 0.71 (95 per cent c.i. 0.49 to 0.87) and a specificity of 1.0 (95 per cent c.i. 0.79 to 1.0).

**Fig. 11:**
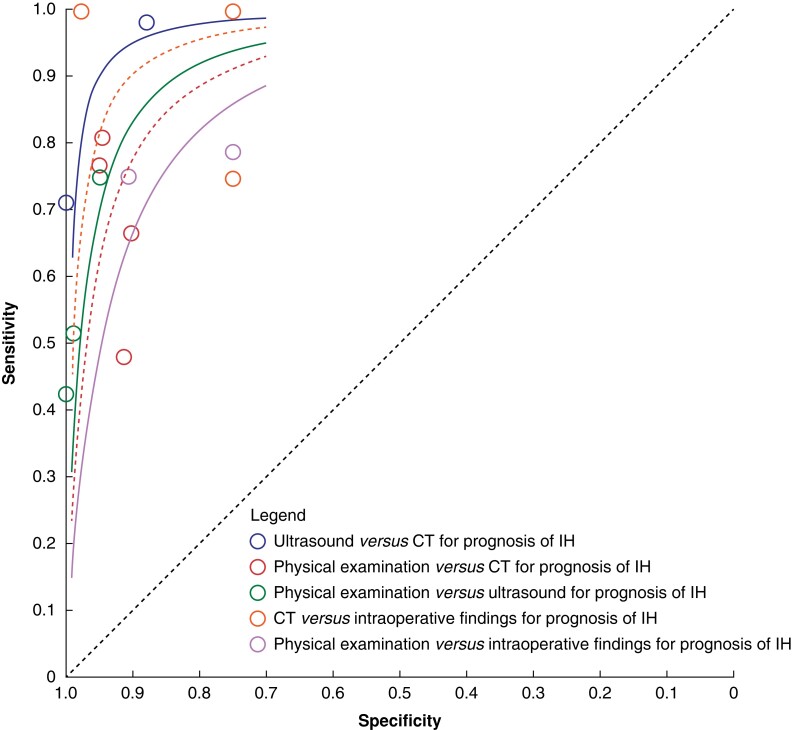
Summary receiver operating characteristic plot for different imaging modalities IH, incisional hernia.

Using all the data analysed for Recommendation A, an SROC plot was produced to show the relative diagnostic accuracy for different imaging modalities (shown in *Fig. 11*). The only reference standard available with 100 per cent sensitivity and specificity is intraoperative diagnosis. Based on the existing evidence, tests, and comparisons available, the SROC plot shows that the most accurate investigation is CT. The second most accurate is ultrasound, which has good accuracy. The least accurate way of diagnosing an incisional hernia is physical examination. Interestingly, none of the published studies looked at the accuracy of MRI, which could be used as an alternative to CT.

### Summary of Findings for Good Practice Statement B

Good Practice Statement B was developed as a result of consultation amongst experts from the guidelines panel and generation of expert evidence.

All members of the group agreed they would use cross-sectional imaging for the majority of incisional hernia cases, and that the need for cross-sectional imaging increases with the size and complexity of the hernia. It was agreed that young patients with small incisional hernias (such as small trocar-site hernias) may not require imaging. Factors likely to affect the need for imaging include the size of the hernia, the complexity of the hernia (loss of domain and multiple previous surgeries), or the suspicion or diagnosis of other pathologies of interest (for example malignancy). The expert evidence suggested that cross-sectional imaging was required to better understand the anatomy of the hernia, assess possible fascial closure, visualize the quality and degree of retraction of the rectus muscles, and provide optimal information for surgical planning. It was suggested that ultrasound lacks the specific detail or accuracy required to image incisional hernias.

Whilst the expert evidence suggested the use of CT, MRI was also recognized as an alternative. CT may be easier to access, with easier ability for surgeons to interpret images. MRI should, however, be considered in cases where radiation exposure is of concern^28^.

**Key Question 3: Is it possible to predict from imaging whether the fascial closure will be possible?**

**Recommendation A:** The guidelines panel suggests that it is not possible to accurately predict with CT whether the fascial defect can be closed without myofascial release (component separation) or peritoneal flap technique (conditional recommendation, very low certainty evidence).

**Good Practice Statement B:** For patients with a midline incisional hernia, it is likely that the fascia will not be able to be closed without myofascial release if on preoperative CT any of the following apply: the defect width is over 8 cm; the area of the hernia is over 164 cm^2^; the rectus/defect ratio is less than 1.34; or the component separation index (CSI) is over 0.146. For hernias approaching or above these measures, the guidelines panel suggests that only surgeons who are competent in advanced techniques such as component separation or peritoneal flap should perform surgery.

The ability to achieve fascial closure during incisional hernia repair can have a significant impact upon prognosis. A number of techniques are available to help achieve fascial closure in large or complex hernias. To help establish whether such techniques may be necessary, preoperative imaging may help to characterize each hernia. This section explores whether there is evidence to support this.

### Search results

**Fig. 12:**
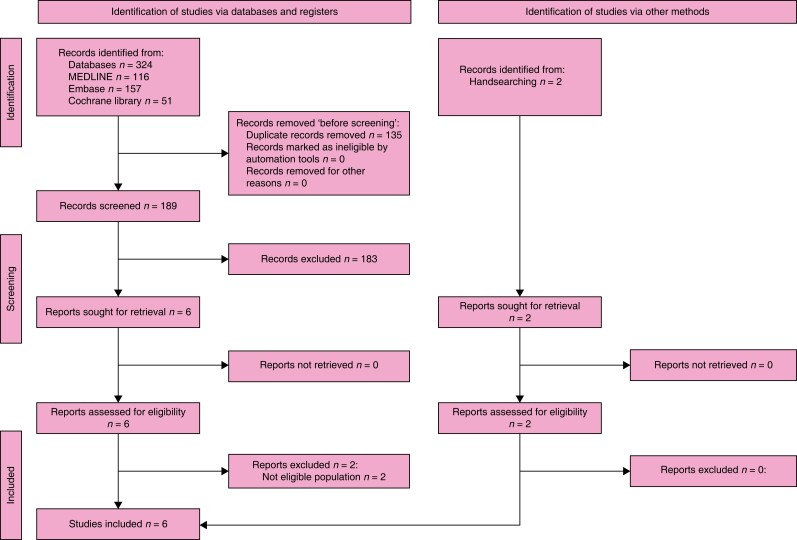
PRISMA flow diagram for Key Question 3

The search retrieved 324 records. After the duplicates were removed, the titles and abstracts of 189 records were screened. A total of six studies were selected for full-text retrieval and were assessed for eligibility. A total of two studies were excluded and a total of four studies met the inclusion criteria. Moreover, handsearching identified another two studies whose full texts were evaluated for eligibility and included in the review. The full study selection process is presented in a PRISMA flow diagram (shown in *Fig. 12*). The Summary of Findings is shown in *Table S4*.

### Evidence for Recommendation A and Good Practice Statement B

Amongst the studies identified, three relevant cross-sectional studies were included, each describing different factors that may influence the likelihood of successful fascial closure in patients who have ‘not’ undergone component separation. Whilst these studies provided insufficient consensus to establish firm recommendations, they supplied evidence that helped form Good Practice Statement B.

#### Hernia defect width

This is a relatively simple measure on cross-sectional imaging and is defined as the maximum diameter between the edges of the rectus abdominis muscles. Two cross-sectional studies assessed the effect of hernia defect width upon fascial closure^97,98^. Love *et al.*^97^ reviewed 342 patients and identified a mean(s.d.) hernia width for patients requiring myofascial release (134 patients) of 12.78(s.d. 3.9) cm, whereas the mean(s.d.) defect width of those not requiring fascial release (208 patients) was 7.53(s.d. 3.8) cm (*P* < 0.001). Blair *et al.*^98^ identified similar values of 11.5(s.d. 5.2) and 7.6(s.d. 4.8) cm respectively (*P* = 0.002).

Blair *et al.*^98^ went on to perform an area under the ROC curve (AUC) analysis to identify the specific hernia width most predictive of the need for myofascial release. Their analysis concluded that a defect width of over 8.3 cm (AUC 0.72) was indicative of an inability to achieve fascial closure without myofascial release.

#### Hernia defect area

This slightly more complex measurement on cross-sectional imaging is calculated by taking the maximum hernia length and multiplying it by the maximum hernia width. Two cross-sectional studies^98,99^ reviewed the effect of hernia defect area upon the likelihood of successful fascial closure. Both studies calculated hernia area by considering them as an ellipse—defined by the largest width and length of the defect.

Blair *et al.*^98^ reviewed 151 open ventral hernia repairs. The mean(s.d.) defect area was 167.4(s.d. 77.4) cm^2^ for patients requiring myofascial release (n=35) and 41.7(s.d. 35.7) cm^2^ for those who did not (n=116). A smaller study of 26 patients by Bellio *et al.*^99^ arrived at respective measurements of 115(s.d. 93) and 49.4(s.d. 85) cm^2^.

Both studies also performed AUC analyses to identify a specific defect area where fascial closure was unlikely to be achieved without myofascial release. Blair *et al.*^98^ concluded that a hernia area of over 164 cm^2^ was most predictive of the need for myofascial release, with Bellio *et al.*^99^ arriving at a similar figure of 156 cm^2^ (relaxed not under Valsalva).

#### Rectus/defect ratio

The rectus defect ratio is defined as the combined maximum width of both rectus muscles divided by the maximum defect width. Love *et al.*^97^ reviewed 342 patients; 208 without myofascial release and 134 with myofascial release. The mean(s.d.) rectus defect ratio was 1.22(s.d. 0.93) for the patients that needed myofascial release and 2.42(1.39) for those that did not.

#### Component separation index

The CSI was first defined by Christy *et al.*^100^ as a hernia’s widest angle of diastasis (calculated from the abdominal aorta) divided by 360. One cross-sectional study analysed the relationship between the CSI and the likelihood of fascial closure. Love *et al.*^97^ found that patients requiring myofascial release (n=134) had a mean(s.d.) CSI of 0.178(s.d. 0.075), whereas those that did not (n=208) had a mean(s.d.) CSI of 0.104(s.d. 0.05).

Love *et al.*^97^ also produced an AUC analysis concluding that a CSI of greater than 0.146 was most accurately predictive of the need for myofascial release.

**Key Question 4: (a) Do all incisional hernias need surgical treatment? and (b) What are the important outcome measures in treatment of incisional hernias?**

**Good Practice Statement A:** For patients with a reducible midline incisional hernia, the risk of an acute hernia accident (strangulation or bowel obstruction) is low (1 per cent in the first year and 2.5 per cent by 5 years).

**Good Practice Statement B:** For patients with symptoms that adversely affect their quality of life (and are medically fit enough for surgery), the guidelines panel suggests surgical repair; after detailed discussion with the patient about the risks and benefits of surgery or watchful waiting.

**Good Practice Statement C:** For patients undergoing treatment for an incisional hernia, the guidelines panel suggests that the most important outcome measure is quality of life. The most important components of quality of life may vary between patients.

**Good Practice Statement D:** For patients undergoing treatment for an incisional hernia, the guidelines panel suggests that other important outcome measures are recurrence, surgical site occurrences, mesh infection, mortality, chronic pain, and cost-effectiveness.

Incisional hernia surgery is not without risk, and it is possible that not everyone’s quality of life will be improved by surgery. Therefore, for some patients, watchful waiting can be a better choice than surgery. Research on outcome measures after incisional hernia repair has tended to focus on results that are important to healthcare systems such as recurrence or surgical site occurrences. Data collection on quality of life before and after incisional hernia repair is lacking in the incisional hernia literature.

### Search results

This KQ was created by combining two KQs; therefore, two searches and literature assessments were performed:

First, do all incisional hernias need surgical treatment?

**Fig. 13:**
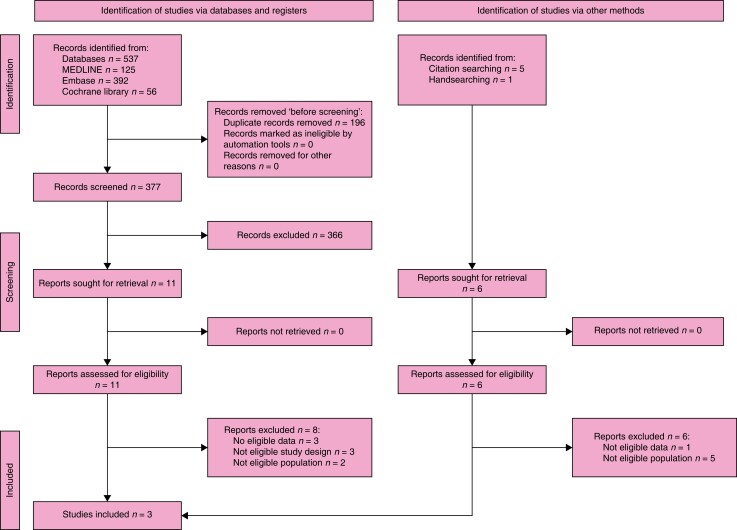
PRISMA flow diagram for Key Question 4(a)

The search retrieved 573 records. After the duplicates were removed, the titles and abstracts of 377 records were screened. A total of 11 reports were selected for full-text retrieval and were assessed for eligibility. A total of eight studies were excluded and a total of three studies met the inclusion criteria. Moreover, handsearching identified another six studies whose full texts were evaluated for eligibility, but all were excluded. The full study selection process is presented in a PRISMA flow diagram (shown in *Fig. 13*).

Second, what are the important outcome measures in treatment of incisional hernia?

**Fig. 14:**
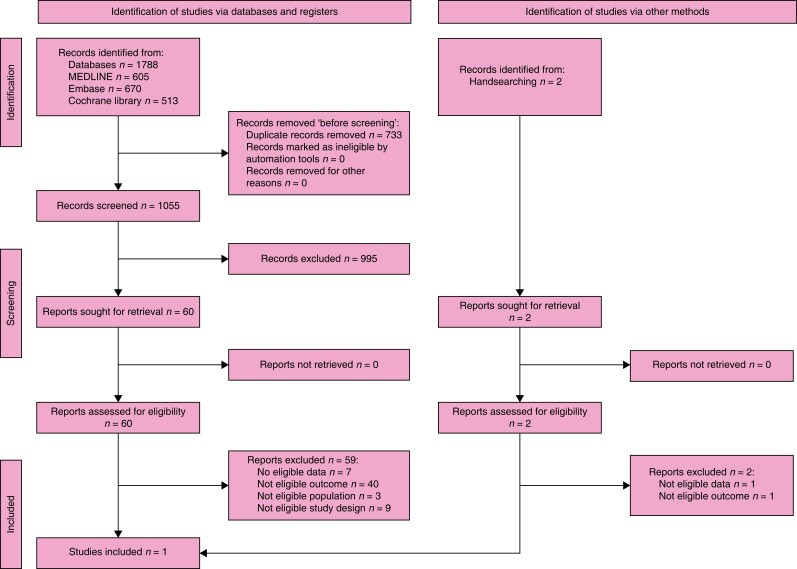
PRISMA flow diagram for Key Question 4(b)

The search retrieved 1788 records. After the duplicates were removed, the titles and abstracts of 1055 records were screened. A total of 60 studies were selected for full-text retrieval and were assessed for eligibility. A total of 59 studies were excluded and only one study met the inclusion criteria. Moreover, handsearching identified two further studies whose full texts were evaluated for eligibility and included in the review. The full study selection process is presented in a PRISMA flow diagram (shown in *Fig. 14*). The Summary of Findings is shown in *Table S5*.

### Evidence for Good Practice Statements A and B

Four of the included studies considered the safety and outcomes of a watchful waiting approach for incisional hernias. In a large observational series of 23 022 people with an incisional hernia undergoing non-operative management with follow-up of up to 8 years, the risk of an acute hernia event at 1 year was 1.24 per cent, increasing to 2.59 per cent by 5 years^101^. Crossover to elective incisional hernia repair due to symptoms was analysed at two time points, within 3 months from diagnosis (early crossover) and between 3 months and 5 years after diagnosis (late crossover). Crossover at these time points was 21.9 and 9.8 per cent respectively.

Similarly a study including 104 patients reported a crossover to surgery from the watchful waiting group of 32.7 per cent at 4 years^102^. Interestingly, in this study, 8 out of 104 (24 per cent) crossed over due to emergency presentation.

Lauscher *et al.*^103^ divided 90 patients undergoing incisional hernia repair into two groups based on preoperative pain scores on a 0–10 visual analogue scale (VAS) (group one, preoperative VAS score of 0–3 (43 patients); and group two, VAS score of 4–10 (47 patients)). The symptomatic group (group two) showed a significant reduction in clinically relevant pain, from 100 to 14.0 per cent (*P* < 0.001), whilst, of those in group one, 7.5 per cent had a VAS score greater than 3 at 18 months after surgery, making their symptoms worse. Despite this, the majority of patients in both groups felt that their symptoms were better after surgery (77.5 *versus* 79.1 per cent), suggesting preoperative pain is not the only important symptom.

Two further articles focused on quality-of-life improvement, measured using Short Form 36 (SF36), in patients undergoing laparoscopic and open incisional hernia repair compared with those awaiting surgery. Both studies reported that the open and laparoscopic incisional hernia repair groups had a significant improvement in quality of life, as measured using SF36, abdominal wall symptoms, and VAS pain scores^104,105^.

### Evidence for Good Practice Statements C and D

Disappointingly, there was a lack of reliable data in the literature on the most important outcome measures for patients undergoing treatment for incisional hernias. It has recently been recognized that there is an unacceptable heterogeneity in reporting outcomes used in the hernia literature and efforts have been made to create a core outcome data set^106^. Due to the absence of data in the literature analysed, evidence for Good Practice Statements C and D were generated using expert evidence.

**Key Question 5: (a) What are the important modifiable risk factors that should be optimized before surgery? and (b) What is the effect of pre-optimization?**

**Good Practice Statement A:** For patients undergoing treatment for an incisional hernia, the important modifiable risk factors are high BMI, poorly controlled diabetes, and smoking.

**Good Practice Statement B:** For patients undergoing treatment for an incisional hernia, the guidelines panel recommends patient pre-optimization before surgery. This includes targeted weight loss (if high BMI), good diabetic control (measured by HbA1c), smoking cessation, and improved pulmonary fitness.

**Good Practice Statement C:** For patients with a symptomatic incisional hernia who are unable to lose weight after a dedicated weight loss programme over a pre-optimization interval and where surgery is technically possible, the guidelines panel suggests that the increased risks of delaying surgery (worsening quality of life and enlarging fascial defect) may outweigh the benefits of further weight loss, but this needs careful discussion of the risks and benefits with the patient.

The majority of patients with an incisional hernia are managed in the elective setting without a time-critical need for surgery. This enables thorough preoperative planning and physiological optimization of the patient to minimize the risk of wound complications and increase the chance of success from surgery^107^. Although the overall evidence on the effects of pre-optimization is limited in incisional hernia patients, there is consensus among experts regarding the role of preoperative assessment and optimization of patients with obesity, with diabetes, who smoke, and with poor nutritional and/or physical status^108^.

### Search results

**Fig. 15:**
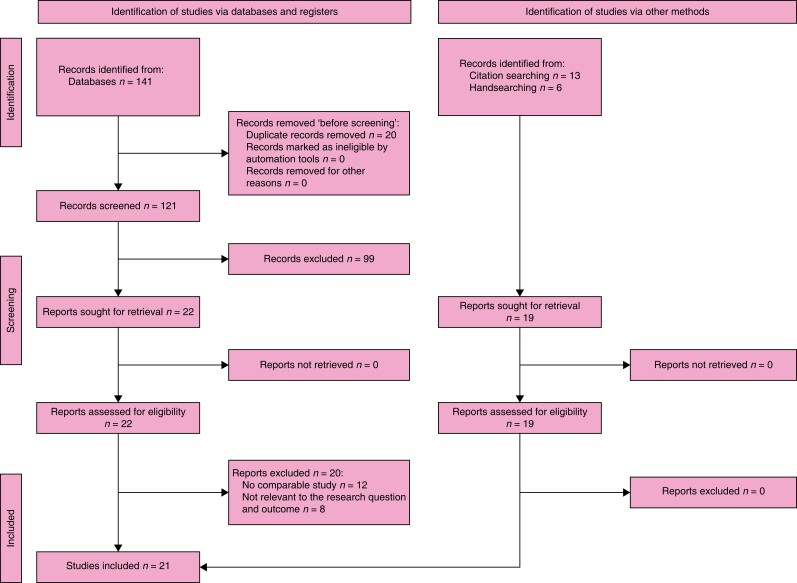
PRISMA flow diagram for Key Question 5

The search retrieved 141 records. After duplicates were removed, the titles and abstracts of 121 records were screened independently by three authors. A total of 22 studies were selected for full-text retrieval and were assessed for eligibility. All but two studies had to be excluded as they did not meet the inclusion criteria. Moreover, checking references of relevant publications and handsearching identified 20 other studies whose full texts were evaluated for eligibility. As a result, 22 observational studies and one systematic review were included in the review. The full study selection process is presented in a PRISMA flow diagram (shown in *Fig. 15*).

### Evidence for Good Practice Statements A, B, and C

#### BMI

Obesity has a well-documented impact on complications after incisional hernia repair, including wound necrosis, SSI, reoperation, and hernia recurrence^109,110^. In a retrospective analysis conducted using data from the American College of Surgeons National Surgical Quality Improvement Program, patients were stratified into seven BMI classes, as well as by type of hernia (reducible *versus* irreducible) and type of incisional hernia (primary *versus* recurrent). A total of 102 191 patients, 58.5 per cent of whom were obese, were included. When stratified by BMI class, higher classes were associated with an increase in all postoperative complications (*P* < 0.0001) with a steady increase in complication rates with increasing BMI class^111^.

To pre-optimize patients, the most commonly used approach is lifestyle modification, preferably by consulting a dietician and fitness coach or physiotherapist. However, significant weight loss can take a long time, especially when patients are not fully motivated or have limited activity due to pain. Therefore, it is important that patients understand that the risks of postoperative complications are directly associated with a higher BMI. Enrolment in formal weight loss programmes is often recommended in the literature, but the participation is low, despite encouragement from surgeons, free programmes, and accessible platforms^112,113^. Nevertheless, participation does correlate with more successful weight loss^112,113^.

Although further weight loss may still be beneficial^111^, most surgeons agree on offering elective surgery to those with a BMI of less than 30 kg/m² and advising weight loss above 35 kg/m²^9^. However, the effect of weight loss on improving outcomes has not been well studied.

A subject of debate has been whether patients should have bariatric surgery to aid weight loss before incisional hernia repair^114,115^. Incisional hernia repair can either be performed simultaneously or more commonly deferred until weight loss has been achieved as a staged procedure. There is very little evidence in the literature about whether this improves outcomes and in many healthcare systems rapid access to bariatric surgery presents logistical challenges.

#### Diabetes

Considerable data exist that poor glycaemic control in the perioperative interval (up to 60 days) increases the risk of postoperative wound complications^116,117^. Glycosylated haemoglobin (HbA1c) is a measure that reflects long-term blood sugar levels and a target HbA1C level of less than 7.0 per cent represents good diabetic control^9^. A meta-analysis of 15 studies found that intensive perioperative glucose control significantly reduces the risk of postoperative SSI in both patients with and without diabetes. Furthermore, intensive glucose control is not associated with a significantly higher risk of hypoglycaemia-related serious adverse events^118^.

Two national databases were analysed to determine the effect of varying severity of diabetes mellitus on ventral hernia repair outcomes. Just over 70 000 patients with diabetes undergoing ventral hernia repair (primary and incisional) were compared with non-diabetic patients. There was an increased complication rate in diabetics compared with non-diabetics. Insulin-dependent or complicated diabetes had significantly worse outcomes after open repair, with higher rates of minor complications (17.3 *versus* 12.7 per cent; *P* < 0.0001) and 58 per cent greater odds of major complications than patients with non-insulin-dependent or uncomplicated diabetes^119^.

#### Smoking

Smoking is a well-established risk factor for the occurrence of postoperative wound complications and long-term hernia recurrence after open incisional hernia repair^120–122^. In a propensity matched study using data from the American College of Surgeons National Surgical Quality Improvement Program, 136 485 non- or ex-smokers were compared with 32 973 current smokers undergoing ventral hernia repair (primary and incisional). The study concluded that patients who smoked at the time of repair had an increased likelihood of postoperative mortality within 30 days (OR 1.45; *P* < 0.05), any morbidity within 30 days (OR 1.35; *P* < 0.0001), wound morbidity within 30 days (OR 1.40; *P* < 0.0001), respiratory morbidity within 30 days (OR 1.14; *P* < 0.0001), and cardiac morbidity within 30 days (OR 1.88; *P* < 0.0001) compared with non/ex-smoker patients^123^. Furthermore, a study including 15 016 patients cared for by 454 surgeons showed that surgeons who pre-optimized patients with regard to weight loss and smoking cessation had better clinical outcomes^124^.

In the study by Sørensen *et al.*^125^, a total of 344 patients scheduled to undergo open inguinal or incisional hernia repair were exposed to various types of smoking cessation instructions. The results showed that patients receiving smoking cessation instructions were more likely to commit to complete smoking cessation compared with patients receiving no instructions (19 *versus* 2 per cent)^125^. Borad *et al.*^123^ also identified smoking not only as a modifiable risk factor with a significant impact on outcomes in patients undergoing ventral hernia repair, but also observed that a delay in surgery and promoting smoking cessation before surgery may help reduce the odds of adverse 30-day postoperative outcomes. In a Cochrane review of 13 RCTs recruiting smokers before elective surgery, again, not specifically ventral hernia repairs, 7 trials looked at the association of preoperative abstinence with postoperative complications. After intensive interventions a reduction in all complications (RR 0.42) and wound morbidity (RR 0.31) was found. However, intervention less than 4 weeks from surgery did not demonstrate a significant impact on morbidity and was less likely to lead to long-term smoking cessation^126^. This would suggest that greater than 4 weeks of smoking cessation is required before surgery.

#### Physical therapy

A recent study assessed the outcomes of a 4-week trimodal prehabilitation programme combining physical therapy, nutritional support, and psychological preparation before major abdominal surgery, including large incisional hernia patients. The study prospectively evaluated 60 patients entering this programme and showed improvement of patients’ functional reserves, quality of life, and psychological status^127^.

An RCT assessed the use of preoperative physical therapy before ventral hernia repair (primary and incisional)^120,122^. An initial publication of results reported promising early outcomes in the group who had preoperative physical therapy compared with those who did not, with lower rates of seroma^122^. However, in the follow-up publication, the long-term results did not show any benefit, with similarly high complication rates in both groups^120^. In addition, there was a high conversion rate to emergency surgery whilst undergoing prehabilitation.

A more recent meta-analysis of RCTs that included subjects undergoing abdominal surgery, randomized to prehabilitation programmes or not, found that inspiratory muscle training, aerobic exercise, and/or resistance training can decrease postoperative complications (OR 0.59). Most dramatic was the reduction in pulmonary complications (OR 0.27)^128^.

**Key Question 6: What is the difference in outcome for mesh *versus* suture repair in incisional hernia repair?**

**Recommendation A:** For patients with a midline incisional hernia a mesh-based repair technique is recommended (strong recommendation, very low certainty evidence).

### Search results

**Fig. 16:**
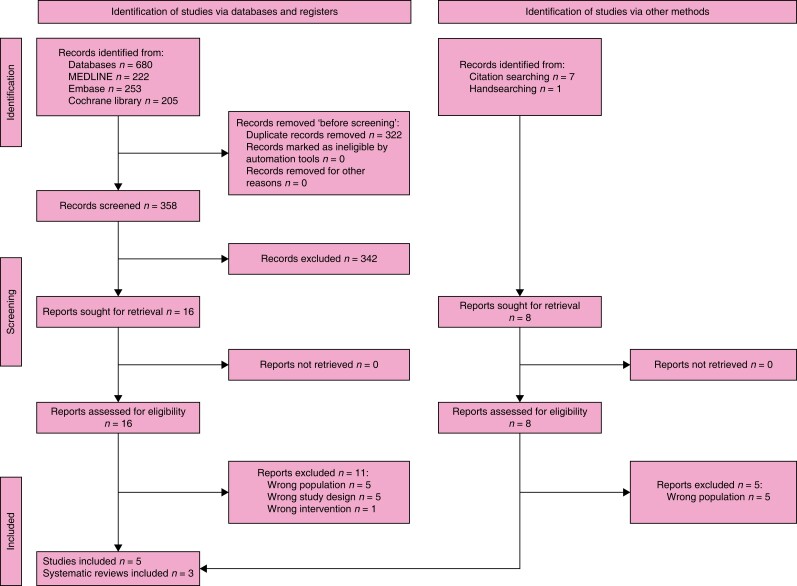
PRISMA flow diagram for Key Question 6

The search retrieved 680 records. After the duplicates were removed, the titles and abstracts of 358 records were screened. A total of 16 studies were selected for full-text retrieval and were assessed for eligibility. A total of 11 reports were excluded and a total of three studies and two systematic reviews met the inclusion criteria^129–133^. Checking references of relevant publications and handsearching identified another eight reports whose full texts were evaluated for eligibility. From these, two studies and one systematic review were included in the review^134–136^. The full study selection process is presented in a PRISMA flow diagram (shown in *Fig. 16*). The Summary of Findings is shown in *Table S6*.

### Evidence for Recommendation A

**Fig. 17:**
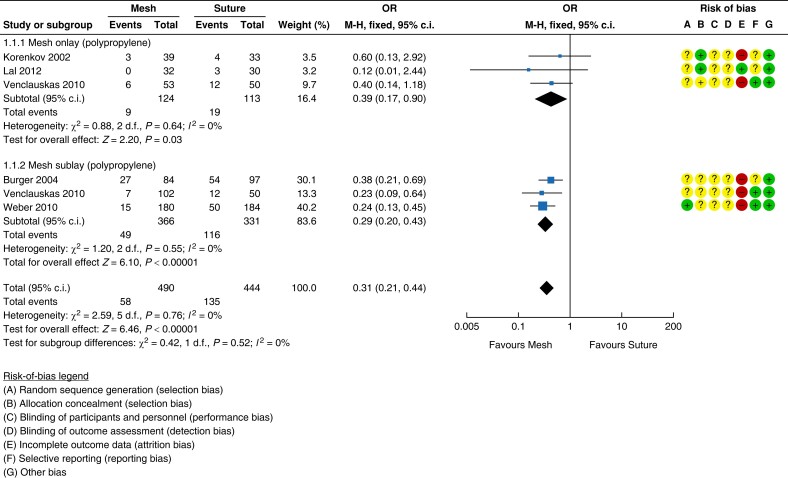
Forest plot: mesh *versus* suture risk of recurrence

Five RCTs assessed the difference in outcome for mesh *versus* suture incisional hernia repair^129–133^. In these studies suture techniques were compared with polypropylene mesh placed in either the onlay or retrorectus position^129–133^. Overall study quality was poor with a high risk of bias (see *Fig. 17*).

#### Recurrence

Mesh resulted in a lower risk of recurrence when compared with suture repair, reaching a statistically significant difference (five studies, 934 patients; mesh 11.8 per cent (58/490) *versus* suture 30.4 per cent (135/444); OR 0.31 (95 per cent c.i. 0.21 to 0.44); *P* < 0.00001)^129–133^.

When studies were pooled by mesh position (onlay or retrorectus), both mesh positions showed statistically significant lower recurrence rates compared with suture repair (onlay: three studies, 237 patients; mesh 7.3 per cent (9/124) *versus* suture 16.8 per cent (19/113); OR 0.39 (95 per cent c.i. 0.17 to 0.90); *P* = 0.003, *I*^2^ = 0 per cent; fixed-effect model; and retrorectus: three studies, 697 patients; mesh 13.4 per cent (49/366) *versus* suture 35 per cent (116/331); OR 0.29 (95 per cent c.i. 0.20 to 0.43); *P* < 0.00001; *I*^2^ = 0 per cent; fixed-effect model). *Figure 17* shows the forest plot for recurrence.

#### Infection

**Fig. 18:**
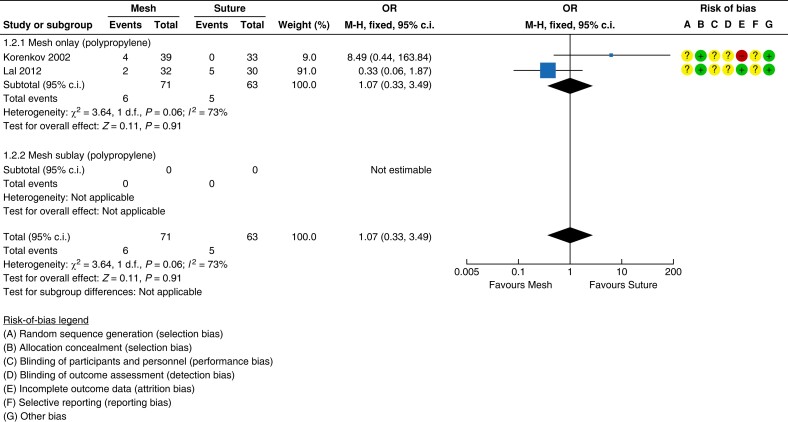
Forest plot: mesh *versus* suture risk of infection

No statistically significant difference in infection rate occurred with mesh *versus* suture repair (two studies, 134 patients; mesh 8.5 per cent (6/71) *versus* suture 7.9 per cent (5/63); OR 1.07 (95 per cent c.i. 0.33 to 3.49); *P* = 0.003; *I*^2^ = 73 per cent; fixed-effect model)^129,133^. See *Fig. 18*.

#### Haematoma

**Fig. 19:**
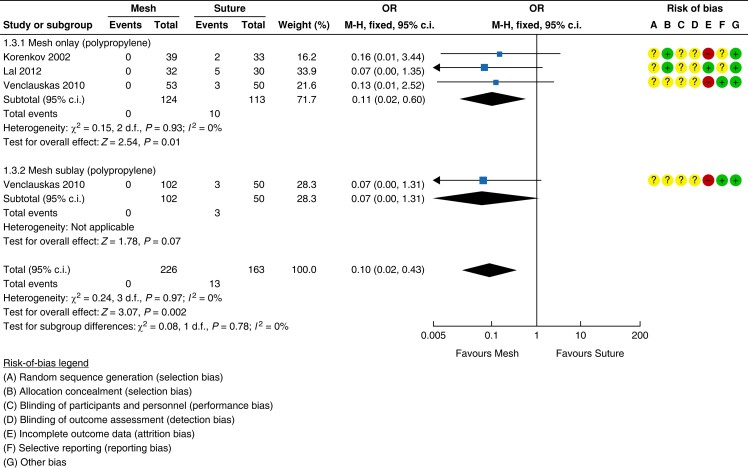
Forest plot: mesh *versus* suture risk of haematoma

Postoperative haematoma was statistically significantly lower using mesh-based repairs compared with suture repairs (three studies, 389 patients; mesh 0 per cent (0/226) *versus* suture 7.8 per cent (13/163); OR 0.10 (95 per cent c.i. 0.02 to 0.43); *P* = 0.002; *I*^2^ = 0 per cent; fixed-effect model)^129,132,133^. The forest plot is shown in *Fig. 19*.

#### Seroma

**Fig. 20:**
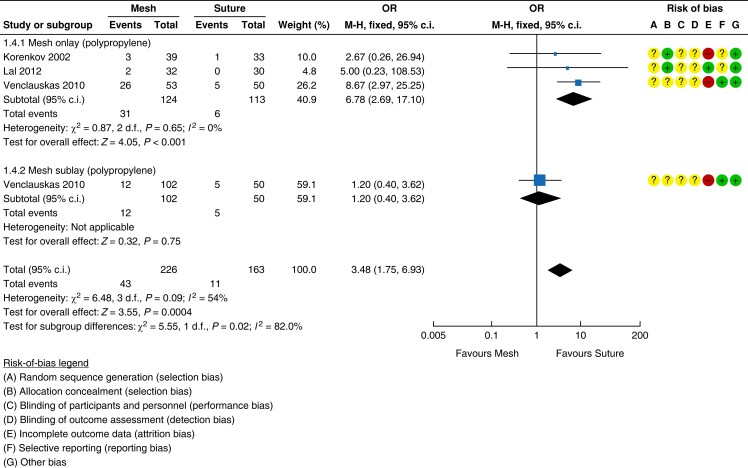
Forest plot: mesh *versus* suture risk of seroma

Suture repair was reported as having a statistically significant lower rate of seroma in comparison with mesh repair (three studies, 389 patients; mesh 19 per cent (43/226) *versus* suture 6.7 per cent (11/163); OR 3.48 (95 per cent c.i. 1.75 to 6.93); *P* = 0.0004; *I*^2^ = 54 per cent; fixed-effect model)^129,132,133^. This was the case for both onlay (three studies, 237 patients; mesh 25 per cent (31/124) *versus* suture 5.3 per cent (6/113); OR 6.78 (95 per cent c.i. 2.69 to 17.10); *P* < 0.0001; *I*^2^ = 0 per cent; fixed-effect model) and retrorectus (one study, 152 patients; mesh 11.8 per cent (12/102) *versus* suture 10 per cent (11/163); OR 1.20 (95 per cent c.i. 0.40 to 3.62); *P* = 0.75) mesh placement. The forest plot is shown in *Fig. 20*.

There was no difference in length of stay using suture or mesh repair.

**Key Question 7: What is the difference in outcome considering different positions of mesh in incisional hernia repair?**

**Recommendation A:** For patients with a midline incisional hernia, the guidelines panel recommends that mesh should be placed in the retromuscular plane (strong recommendation, very low certainty evidence).

**Good Practice Statement A**: Surgeons performing incisional hernia repair should be familiar with the technique for positioning the mesh in different planes (including onlay, retromuscular, and intraperitoneal).

**Good Practice Statement B**: For patients with a midline incisional hernia, the guidelines panel suggests that any mesh in the abdominal cavity exposed to the abdominal viscera should be used with caution due to the risk of long-term complications at any subsequent abdominal surgery.

Terminology and nomenclature to describe mesh position within the abdominal wall is often inconsistent and varies with surgeon/institutional interpretation. It is important that uniform terminology is used for consistency of clinical management and to allow for an evidence-based comparison of different techniques. In an effort to establish this, Parker *et al.*^137^ have provided an international classification produced by Delphi methods on the different mesh placement planes. The most commonly used of these are onlay (on the fascia below the subcutaneous fat), retrorectus (between the rectus muscle and the posterior rectus sheath), preperitoneal (between the posterior rectus sheath and the peritoneum), and intraperitoneal (inside the peritoneal cavity against the peritoneum)^137^. The term retromuscular encompasses both the retrorectus and preperitoneal planes. The optimal mesh plane should be associated with a low recurrence rate, a low risk of complications such as seroma, haematoma, SSI, and adhesions, and, finally, a low risk of mesh sensation, acute pain, and chronic pain.

### Search results

**Fig. 21:**
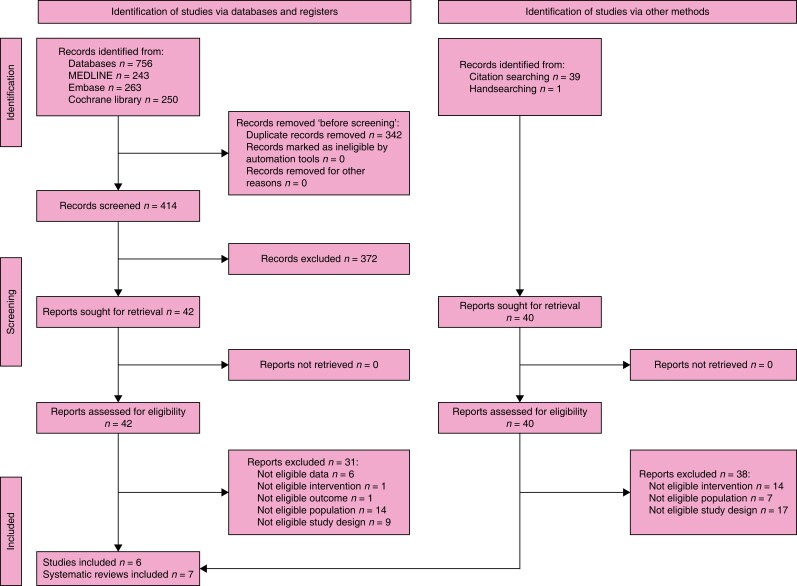
PRISMA flow diagram for Key Question 7

The search retrieved 756 records. After the duplicates were removed, the titles and abstracts of 414 records were screened. A total of 42 reports were selected for full-text retrieval and were assessed for eligibility. A total of 31 reports were excluded. A total of four studies and seven reviews met the inclusion criteria. Handsearching and checking references identified another 40 reports whose full texts were evaluated for eligibility and two studies were included. The full study selection process is presented in a PRISMA flow diagram (shown in *Fig. 21*). The Summary of Findings is shown in *Table S7*.

### Evidence for Recommendation A and Good Practice Statements A and B

#### Onlay *versus* retrorectus

**Fig. 22:**
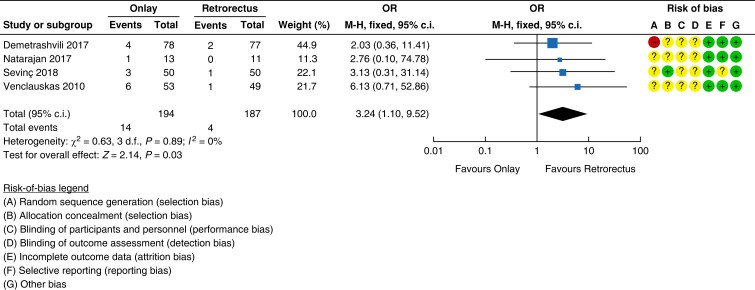
Forest plot: onlay *versus* retrorectus risk of recurrence

**Fig. 23:**
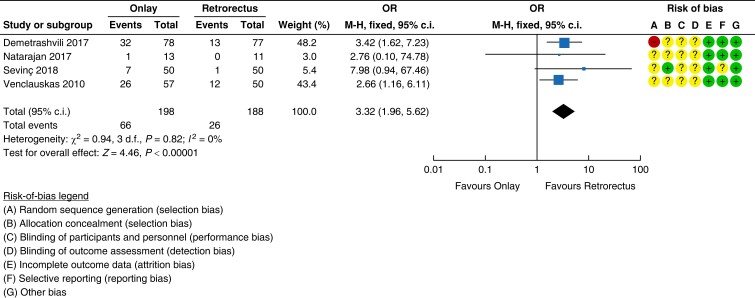
Forest plot: onlay *versus* retrorectus risk of seroma

Four RCTs of low to moderate quality compared open onlay with retrorectus mesh placement for elective repair of midline incisional hernias^132,138–140^. Pooled analysis revealed an increased risk of recurrence, when placing the mesh in the onlay position (7.2 per cent (14/194)) compared with the retrorectus position (2.1 per cent (4/187)) (forest plot in *Fig. 22*). Furthermore, the risk of seroma was increased with the use of an onlay mesh position (33.3 per cent (66/198)) compared with a retrorectus mesh position (13.8 per cent (26/188)) (*Fig. 23*). For other wound-related complications such as haematoma and surgical site occurrences, the rates were also higher with the use of onlay mesh (see *Table S8*). There were no data on pain. For the pooled analysis of the RCTs, the risk of bias was high and the imprecision was serious, leading to a very low certainty of the evidence.

Furthermore, four systematic reviews and meta-analyses were identified^141–144^. Albino *et al.*^141^ assessed 62 studies from 1996 to 2012 comparing onlay, interposition, retrorectus, and intraperitoneal mesh placement for all types of ventral hernias (primary and incisional), including both open and laparoscopic approaches. It was concluded that intraperitoneal and retrorectus mesh placement was associated with a lower risk of recurrence and complications than other mesh positions^141^. Sosin *et al.*^144^ updated that review evaluating 51 further studies from 2013 to 2018 using the same inclusion criteria and concluded that retrorectus mesh placement was associated with a lower risk of recurrence than intraperitoneal mesh placement. Timmermans *et al*.^142^ included two RCTs and seven cohort studies comprising nearly 2000 patients and concluded that recurrence rates and surgical site occurrences were decreased when placing the mesh in the retrorectus position compared with the onlay position. Holihan *et al.*^143^ in a network meta-analysis of 20 RCTs including both primary ventral and incisional hernias found that retrorectus mesh placement resulted in the lowest risks of recurrence and SSI.

#### Onlay *versus* intraperitoneal

Only one small low-quality RCT compared open onlay with open intraperitoneal mesh and concluded that the risk of recurrence was 27.3 per cent (6/22) for onlay *versus* 0.0 per cent (0/19) for open intraperitoneal with an OR of 15.26 (95 per cent c.i. 0.80 to 293.6). The risk of seroma was 31.8 per cent (7/22) for onlay *versus* 0.0 per cent (0/19) for open intraperitoneal with an OR of 18.87 (95 per cent c.i. 1.00 to 356.74)^145^.

#### Minimally invasive retrorectus (mini- or less-open sublay) *versus* laparoscopic IntraPeritoneal Onlay Mesh

One cohort study from the German Hernia Registry evaluated the endoscopically assisted mini- or less-open sublay (MILOS) repair compared with a propensity matched group of laparoscopic (IntraPeritoneal Onlay Mesh (IPOM)) repairs and found that the MILOS repair with mesh in the retrorectus position was associated with decreased complications, recurrence rate (2.2 per cent (10/463) with MILOS *versus* 7.3 per cent (34/463) with laparoscopic IPOM; OR 0.28 (95 per cent c.i. 0.14 to 0.57)), and pain at 1 year after surgery^146^.

#### Summing up the evidence

The pooled analysis of the RCTs revealed a low certainty of the evidence as the systematic reviews and meta-analysis included heterogeneous data with different surgical approaches, sometimes also mixing primary and incisional ventral hernia cohorts. There was also significant publication bias.

Retromuscular mesh placement for midline incisional hernia repairs seems to have better outcomes than other mesh positions and the strength of recommendation was therefore upgraded to strong by the guidelines panel. However, there may be cases where retromuscular mesh placement is not possible or very difficult and therefore it is important to be familiar with the surgical technique for placing the mesh in other positions.

Due to the risk of intraperitoneal adhesions, and with the growing popularity of alternative minimally invasive methods for retromuscular repair such as MILOS and extended Totally ExtraPeritoneal (eTEP), which are showing promising results, it is suggested to keep the mesh out of the peritoneal cavity where possible to limit contact with the viscera.

**Key Question 8: What is the difference in outcome between techniques (open, laparoscopic, and robotic) for incisional hernia repair?**

**Good Practice Statement A**: For patients with a midline incisional hernia, the guidelines panel suggests that laparoscopic, robotic, or open surgery may be appropriate depending on the patient and hernia characteristics and provided the surgeon has appropriate expertise.

The choice of technique for incisional hernia repair is often decided by surgeon preference and expertise. Irrespective of the approach used, the surgeon should be trained in the technique. The technique should be performed in the correct way, with a focus on preservation and restoration of abdominal wall function and careful tissue handling. Furthermore, the decision to operate and the choice of technique should involve the informed consent process and should be a shared decision between the patient and their surgeon^147^.

### Search results

**Fig. 24:**
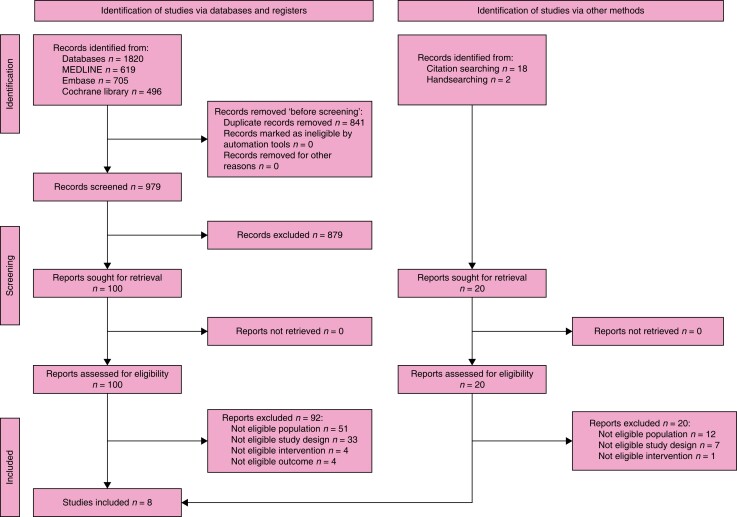
PRISMA flow diagram for Key Question 8

The search retrieved 1820 records. After the duplicates were removed, the titles and abstracts of 979 records were screened. A total of 100 reports were selected for full-text retrieval and were assessed for eligibility. A total of eight studies met the inclusion criteria and a total of 92 reports were excluded. Moreover, checking references of relevant publications and handsearching identified another 20 reports whose full texts were evaluated for eligibility, but all were excluded. The full study selection process is presented in a PRISMA flow diagram (shown in *Fig. 24*).

The Summary of Findings is shown in *Table S8*.

### Evidence for Good Practice Statement A

#### Recurrence

**Fig. 25:**
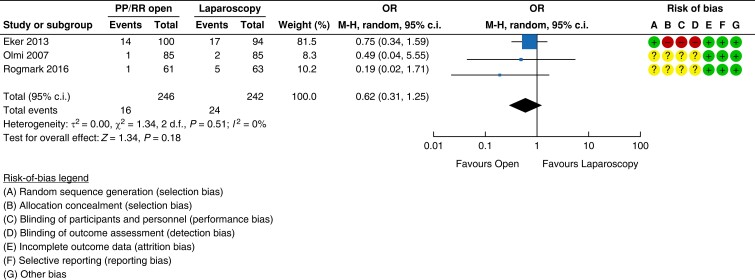
Forest plot: retrorectus *versus* IntraPeritoneal Onlay Mesh (IPOM) risk of recurrence PP, pre-peritoneal; RR, retrorectus.

Three RCTs collectively randomized 488 patients undergoing incisional hernia repair into either open retrorectus or an IPOM repair^148–150^. Of these, 440 patients completed at least 1 year of follow-up. In all three RCTs, the hernia defect was not closed in the majority of the IPOM patients and in an unknown number of the open cases. Furthermore, there is a high risk of bias amongst these studies and therefore only a very low certainty of evidence was achieved. The recurrence rates were 10 per cent (24/243) for the IPOM group and 6 per cent (16/252) for the open retrorectus group. There was no statistically significant difference between the groups (OR 0.62 (95 per cent c.i. 0.31 to 1.25); RR 0.68 (95 per cent c.i. 0.37 to 1.23)) (see *Fig. 25*).

#### Surgical site infection and perioperative complications

**Fig. 26:**
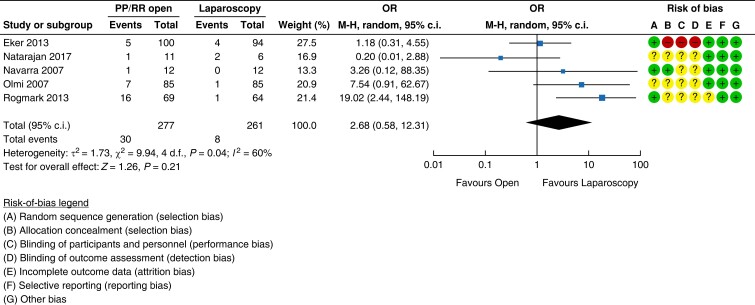
Forest plot: retrorectus *versus* IntraPeritoneal Onlay Mesh (IPOM) risk of surgical site infection

A lower rate of SSI is one of the most commonly described advantages of minimally invasive surgery. Five trials have reported the incidence of SSI in their short-term follow-up^105,139,148,149,151^. There was a higher proportion of those with superficial SSI in the open retrorectus group compared with the laparoscopic IPOM group (10.8 per cent (30/277) *versus* 3.1 per cent (8/261)). This difference did not reach statistical significance as evidenced by the wide confidence intervals shown in the forest plot in *Fig. 26* (OR 2.68 (95 per cent c.i. 0.58 to 12.31); RR 2.43 (95 per cent c.i. 0.58 to10.14)). However, the number of deep SSI events requiring intervention was similar (1.5 per cent in both randomized groups; 5/277 in the open retrorectus group *versus* 4/261 in the laparoscopic IPOM group; OR 1.07 (95 per cent c.i. 0.30 to 3.83)).

The laparoscopic approach was associated with a higher risk of perioperative complications (8 per cent (14/170) *versus* 2 per cent (3/179)). A number of patients had to be converted to open surgery (13/158). Within the analysed studies, 4.6 per cent (12/261) of patients undergoing laparoscopic repair went on to have a laparotomy during the course of the follow-up compared with 3.2 per cent (8/275) of patients in the open group (OR 0.69 (95 per cent c.i. 0.28 to 1.66)).

#### Length of stay and return to activity

Length of stay is another parameter mentioned as an advantage of laparoscopic surgery. In two studies that have reported length of stay, it was shorter for laparoscopic surgery (2.7–5.7 days) compared with open surgery (9.9 days)^139,149^. The main reason mentioned for prolonged length of stay for open surgery was drain placement and issues regarding soft tissues. However, in a third study, length of stay was the same (2 days for each group)^149^.

Return to activity was not measured in a standardized way amongst the selected studies. Olmi *et al.*^149^ reported a faster return to activity after laparoscopic IPOM compared with open retrorectus repair (13 *versus* 25 days). Natarjan *et al.*^139^ reported the percentage of people being able to return to activity after 2 weeks. In the open retrorectus group, 81 per cent (9/11) were active, whereas this was only the case for 66 per cent (4/6) after IPOM repair.

#### Cosmesis

There were no data given on patient satisfaction with regard to changes in abdominal cosmesis after incisional hernia repair.

#### Robotic approach

Despite large-scale uptake over recent years of robotic surgery for incisional hernia repair, the guidelines panel only identified one RCT comparing laparoscopic *versus* robotic repair of ventral hernias both with an IPOM+ technique with reported outcomes at 1 month and 1 year. This included a heterogeneous group of patients with primary and incisional hernias and thus this paper was excluded from the meta-analysis. In another study, the recurrence rates were similar between the groups (8.5 per cent (5/59) in the laparoscopic group *versus* 2.2 per cent (4/65) in the robotic group; OR 1.41 (95 per cent c.i. 0.36 to 5.53)). Interestingly, only in the region of 50 per cent of patients in both groups reported resolution of symptoms after surgery^152^.

#### Summing up the evidence

While the guidelines panel analysed all available literature regarding open compared with minimally invasive surgery for midline incisional hernia of up to 10 cm in diameter during the initial search, disappointingly, there was no evidence on newer variations of laparoscopic techniques such as IPOM+ technique (which includes closure of the defect), or other more sophisticated minimally invasive operations placing mesh in the retrorectus space or eTEP. Furthermore, there are no current comparative studies of open *versus* laparoscopic or robot-assisted incisional hernia repair with mesh placed in the retrorectus position.

**Key Question 9: Is there a benefit of primary fascial closure in midline incisional hernia mesh repair?**

**Recommendation A:** For patients having repair of a midline incisional hernia (laparoscopic or open repair), the guidelines panel recommends that the fascial defect should be closed and bridging with a mesh should be avoided (strong recommendation, low certainty evidence).

One of the goals of incisional hernia surgery is to try to restore the abdominal wall anatomy and function. In keeping with this, closure of the fascial defect is considered an essential component of open repair and is also thought to be beneficial in laparoscopic repair. However, the effect of closure of the defect both in terms of recurrence rate and patient-reported outcomes is unclear.

### Search results

**Fig. 27:**
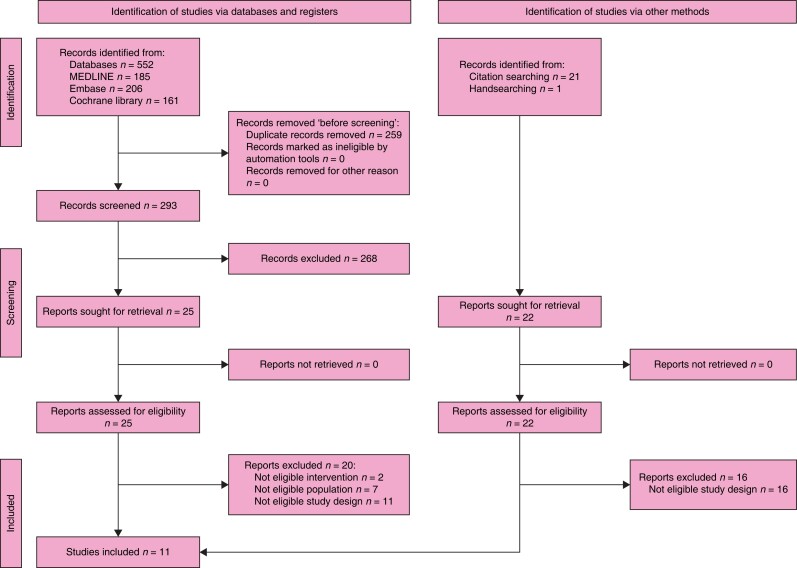
PRISMA flow diagram for Key Question 9

The search retrieved 552 records. After the duplicates were removed, the titles and abstracts of 293 records were screened. A total of 25 reports were selected for full-text retrieval and were assessed for eligibility. A total of 20 reports were excluded. A total of five studies met the inclusion criteria. Handsearching and checking the references identified another 22 reports whose full texts were evaluated for eligibility and six studies were included. The full study selection process is presented in a PRISMA flow diagram (shown in *Fig. 27*). The Summary of Findings is shown in *Table S9*.

### Evidence for Recommendation A

Three RCTs were identified concerning the difference in outcome for fascial closure *versus* bridging in laparoscopic incisional hernia repair, all published in 2020^153–155^. No studies were found comparing defect closure *versus* bridging in open surgery. The following variables were evaluated in the meta-analysis: recurrence, haematoma, seroma, pain, and length of stay.

#### Recurrence

**Fig. 28:**
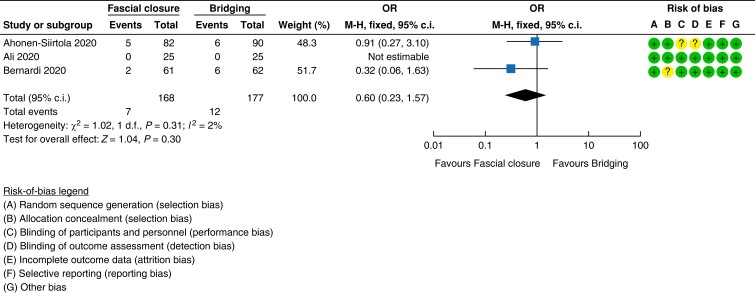
Forest plot: fascial closure *versus* bridging risk of recurrence

Fascial closure resulted in a lower risk of recurrence when compared with bridging^153–155^; fascial closure 4.2 per cent (7/68) *versus* bridging 6.8 per cent (12/177); OR 0.60 (95 per cent c.i. 0.23 to 1.57) (*Fig. 28*).

#### Haematoma/seroma

**Fig. 29:**
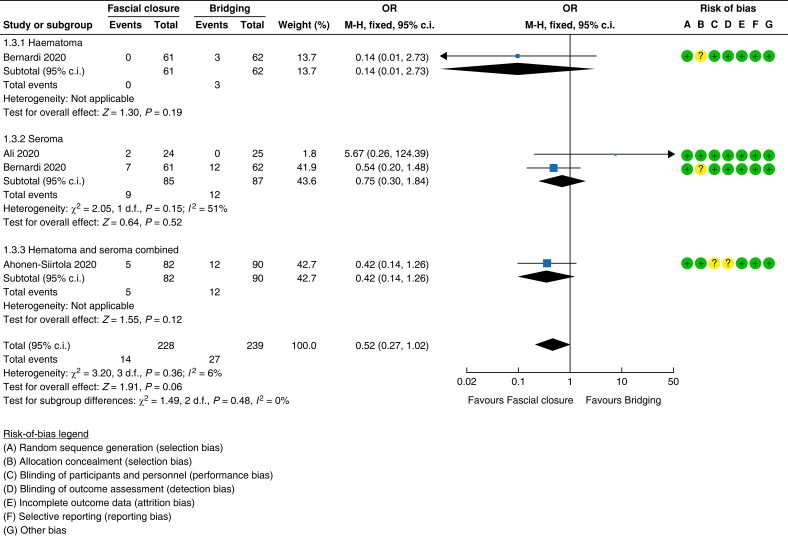
Forest plot: fascial closure *versus* bridging risk of seroma and haematoma

The three RCTs did not uniformly measure seroma and haematoma separately. In one study, fascial closure resulted in a lower risk of haematoma and seroma combined when compared with bridging (fascial closure 6.1 per cent (5/82) *versus* bridging 13.3 per cent (12/90); OR 0.42 (95 per cent c.i. 0.30 to 1.26))^153,155^. In another study, fascial closure resulted in a lower risk of haematoma when compared with bridging (fascial closure 0 per cent (0/61) *versus* bridging 4.8 per cent (0/61); OR 0.14 (95 per cent c.i. 0.01 to 2.73))^154^. In two RCTs, fascial closure resulted in a lower risk of seroma when compared with bridging (10.6 per cent (9/85) *versus* bridging 13.8 per cent (12/85); OR 0.75 (95 per cent c.i. 0.30 to 1.84)). When the results of all three RCTs were pooled for seroma and/or haematoma, the benefit of fascial closure still failed to reach statistical significance (fascial closure 6.1 per cent (14/228) *versus* bridging 11.3 per cent (27/239); OR 0.52 (95 per cent c.i. 0.27 to 1.02)) (*Fig. 29*)^153–155^.

There was no difference in postoperative pain or length of stay. Only one RCT analysed quality of life and reported a statistically non-significant benefit of fascial closure^154^.

There are a small number of RCTs looking at fascial closure in laparoscopic incisional hernia repair of low quality, and none assessing the impact in open surgery. For all studies, imprecision was scored as serious. In laparoscopic incisional hernia repair, there appears to be a decreased risk of recurrence, haematoma, or seroma formation with fascial closure.

**Key Question 10: What is the difference in the outcome using different techniques for mesh fixation in (a) intraperitoneal and (b) extraperitoneal mesh placement for incisional hernia repair?**

**Good Practice Statement A:** For patients undergoing surgery using a laparoscopic intraperitoneal onlay mesh, the guidelines panel suggests that a variety of methods including glues, tacks, and sutures (both absorbable and non-absorbable) are possible, with little difference in clinical outcomes.

**Good Practice Statement B:** For patients having an open retrorectus repair of a midline incisional hernia, whilst the original description described the use of transfascial sutures, the guidelines panel suggests that other options such as fixation to the posterior layer or self-fixing meshes are acceptable and may reduce the risk of chronic pain.

Fixation of mesh placed in the intraperitoneal position is necessary. The options include penetrating fixation, with tacks (permanent or absorbable, and single crown or double crown), staples, or sutures (permanent or absorbable), which can be transfascial or placed as tacking stitches, and non-penetrating fixation with glue (fibrin or cyanoacrylate based). Indeed, many surgeons use a combination of these.

As well as fixation, closure of the defect, the mesh landing zone, and mesh type may influence outcomes. Differences in tacker construct such as depth of penetration and cross-sectional design to reduce pull out, as well as the number and location of tacks used per square centimetre of mesh, may influence outcomes. Absorbable fixation was designed in an effort to minimize long-term chronic pain; however, injury to a nerve may occur at the time of tack or suture insertion, and therefore resorption may not influence long-term chronic pain.

Similarly, mesh placed in the preperitoneal, retrorectus, or onlay plane in open surgery may have no fixation, be self-fixing, involve suture fixation (permanent or absorbable either to the posterior fascia or transfascial), or involve glue (fibrin based or cyanoacrylate based).

### Search results

**Fig. 30:**
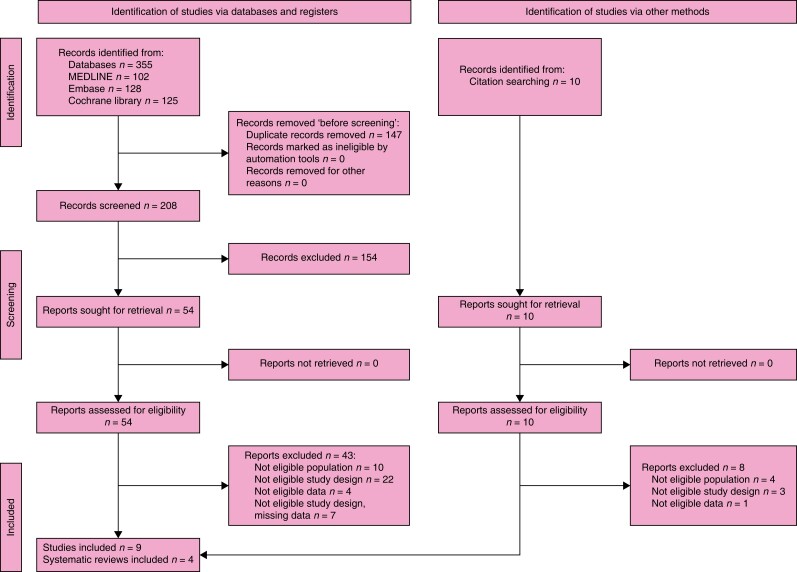
PRISMA flow diagram for Key Question 10

The search retrieved 355 records. After the duplicates were removed, the titles and abstracts of 208 records were screened. A total of 54 reports were selected for full-text retrieval and were assessed for eligibility. A total of 43 reports were excluded and a total of seven studies and four systematic reviews met the inclusion criteria. Checking references of relevant publications and handsearching identified another 10 reports whose full texts were evaluated for eligibility; two of these studies were included in the review. The full study selection process is presented in a PRISMA flow diagram (shown in *Fig. 30*).

The Summary of Findings is shown in *Table S10*.

### Evidence for Good Practice Statement A

An RCT comparing double crown permanent tacker (DCPT), double crown absorbable tacker (DCAT), and glue (75 patients) reported no difference in quality of life, postoperative pain, surgical site occurrences, length of stay, or recurrence^156^. Similarly, another RCT, comparing DCPT *versus* DCAT (both had additional four-corner transfascial permanent sutures) (90 patients), reported no difference in quality of life, length of stay, chronic pain, or recurrence^157,158^. Two small RCTs compared DCPT with permanent transfascial sutures (36 and 72 patients) and reported that the transfascial suture group had more pain 4 h after surgery^159^ and at 6 weeks. There was no difference in pain at 6 months, with similar length of stay and recurrence^160^.

### Evidence for Good Practice Statement B

No RCTs comparing open fixation met the inclusion criteria for these guidelines. Expert opinion was generated using the GRADE expert evidence forms, but opinion was divided, with one-third favouring transfascial sutures and two-thirds against their use due to pain.

Two small cohort trials (26 and 50) respectively and one larger cohort trial (244) assessed the use of self-fixing meshes compared with fixation with transfascial sutures^161–163^. The two small studies suggested that the self-fixing mesh resulted in less inpatient narcotic analgesia use^161^, and reduced early postoperative pain^162^. However, the larger study reported increased seroma, wound events, and reoperation rates in the self-gripping mesh group^163^.

**Key Question 11: What is the benefit of enhanced recovery after surgery (ERAS) in incisional hernia repair?**

**Good Practice Statement A:** For patients having repair of a midline incisional hernia, the guidelines panel suggests that there is not sufficient evidence to recommend enhanced recovery protocols.

ERAS is gaining more and more acceptance in different fields of surgery^164^. The benefit of ERAS in incisional hernia repair in the authors’ target group of patients with hernias up to 10 cm in width is unclear.

### Search results

**Fig. 31:**
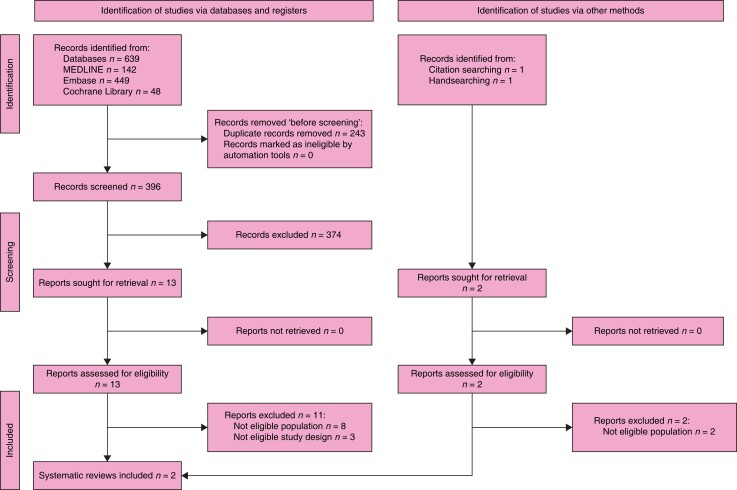
PRISMA flow diagram for Key Question 11

The search retrieved 639 records. After the duplicates were removed, the titles and abstracts of 396 records were screened. A total of 13 reports were selected for full-text retrieval and were assessed for eligibility. A total of 11 reports were excluded and a total of 2 systematic reviews met the inclusion criteria. Checking references of relevant publications and handsearching identified another two reports whose full texts were evaluated for eligibility, but these were excluded. The full study selection process is presented in a PRISMA flow diagram (shown in *Fig. 31*).

The Summary of Findings is shown in *Table S11*.

### Evidence for Good Practice Statement A

The use of ERAS protocols in incisional hernia repair is promising. Two systematic reviews and meta-analyses evaluating the use of ERAS in complex abdominal wall reconstruction have been published recently^165,166^. Given that the focus of these studies was complex incisional hernias and not more simple midline hernias (the focus of these guidelines) the evidence is indirect. The first publication, by Sartori *et al.*^165^ includes five retrospective cohort papers (search up to April 2020). The length of hospital stay was significantly lower in the ERAS group (albeit only 0.6 days) without increasing the overall postoperative morbidity and readmission rate. However, according to GRADE criteria, the quality of evidence was very low to low for all endpoints. In addition, there was large heterogeneity with respect to the complexity of the surgery performed (for example component separation techniques in 29–100 per cent of the patients), as well as the ERAS protocols across the different studies. The authors also report that it is unclear whether any change in the discharge criteria after the introduction of an ERAS pathway may have changed the length of stay in the included studies. Two months later, another meta-analysis on the same topic included four of the same papers (search up to end of November 2019), together with one additional paper not included in the first meta-analysis^163^. The conclusions were similar with a decreased length of stay in the ERAS group of 0.89 days.

Considering the methodological aspects and the fact that the type of abdominal wall defects included in the various studies is not representative for the patient population of the authors’ guidelines, the panel found only indirect evidence, which is not sufficient to give a clinical recommendation.

It thus suggests that ERAS protocols for non-complex incisional hernia repair should be used in experimental and cohort studies to investigate their effectiveness in this patient group.

**Key Question 12: Should prophylactic antibiotics be used in the elective repair of incisional hernia in adult patients?**

**Recommendation A:** For patients having repair of a midline incisional hernia, the guidelines panel suggests a single prophylactic dose of antibiotic (according to local hospital policy). If the operation is longer than 4 h, the guidelines panel suggests a second prophylactic dose, depending on the antibiotic used, amount of blood loss, and surgical approach (conditional recommendation, very low certainty evidence).

The need for prophylactic antibiotics during hernia repair varies between institutions and cases, dependent upon both patient-specific and procedure-specific risk factors. This KQ explores the evidence for their use.

### Search results

**Fig. 32:**
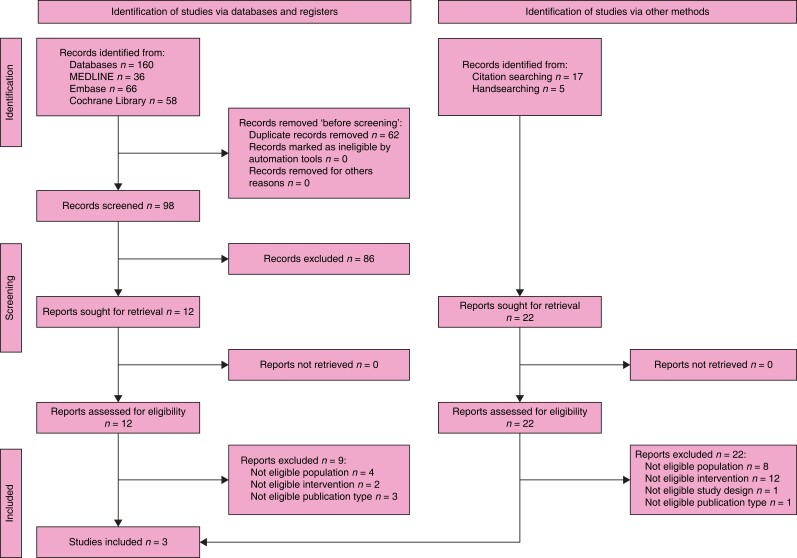
PRISMA flow diagram for Key Question 12

The search retrieved 160 records. After duplicates were removed, the titles and abstracts of 98 records were screened. A total of 12 reports were selected for full-text retrieval and were assessed for eligibility. A total of nine studies were excluded and a total of three studies met the inclusion criteria. Checking references of relevant publications and handsearching identified another 22 studies whose full texts were evaluated for eligibility, but were excluded. The full study selection process is presented in a PRISMA flow diagram (shown in *Fig. 32*). The Summary of Findings is shown in *Table S12*.

### Evidence for Recommendation A

Two meta-analyses were identified, but later excluded, either due to an incorrect patient cohort (mostly inguinal hernias)^167^ or insufficient data regarding the effect of antibiotics^142^. A total of four RCTs were identified, three of which were excluded either due to an unsuitable research question^168,169^ or an inappropriate study cohort^170^. One further RCT by Abramov *et al.*^171^ involved a mixed patient cohort of both umbilical and incisional hernia repairs, but produced a 16-patient subgroup analysis of incisional hernia patients that was included for the authors’ analysis.

Two cohort studies were identified and included. Rios *et al.*^172^ developed a prospective study evaluating antibiotic prophylaxis in 216 incisional hernia repairs. Despite their study including 139 patients with large (greater than 10 cm) incisional hernia and 40 patients with non-midline incisional hernia, results were deemed relevant and therefore included. Kirchhoff *et al.*^173^ examined the impact of antibiotic prophylaxis on the rates of SSI and reoperations in 13 513 patients undergoing laparoscopic incisional hernia repair. Whilst 1763 (13 per cent) of patients had a large incisional hernia (greater than 10 cm) and 3413 (25 per cent) of cases were non-midline, their results were also deemed relevant and assessed.

Existing guidelines from other groups^8,11,12^ were not included due to a mixed cohort of primary ventral and inguinal hernias.

In the Abramov *et al*.^171^ RCT, 16 patients with incisional hernias were included. A total of eight patients received 1 g cefonicid 30 min before surgery and eight patients formed a control group without prophylaxis. Mesh was used in four patients from the treatment group and only two from the control group. No patient in the antibiotic prophylaxis group developed a postoperative wound infection (0/8) compared with four of the eight patients (50 per cent) in the control group.

In the prospective study of 216 incisional hernia repairs by Rios *et al.*^172^, antibiotic prophylaxis was administered in 140 patients (either a first- or second-generation cephalosporin or amoxicillin with clavulanic acid) compared with 76 patients in the control group. In total, 39 out of 216 patients (18.1 per cent) developed an SSI. From the antibiotic prophylaxis group, 19 of the 140 patients (13.6 per cent) developed an infection compared with 20 out of 76 (26.3 per cent) in the control group (*P* = 0.00991). Multivariate analysis revealed that antibiotic prophylaxis was associated with reducing postoperative infection (OR 0.23; *P* = 0.0023).

**Fig. 33:**
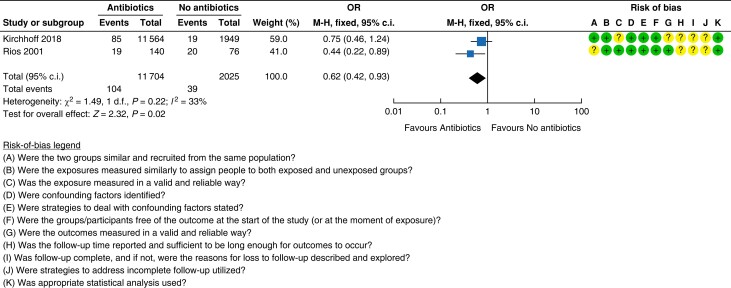
Forest plot: antibiotic prophylaxis *versus* no antibiotic prophylaxis risk of infection

In a registry-based study, Kirchhoff *et al.*^173^ analysed 13 513 laparoscopic incisional hernia repairs. SSI rates were not significantly different between the two groups after propensity-score matching analysis was carried out on 1940 patient pairs (0.57 per cent in the antibiotic prophylaxis group *versus* 0.93 per cent in the control group; OR = 0.611 (95 per cent c.i. 0.261 to 1.366); *P* = 0.265). Unadjusted analysis for the risk of deep SSI was also not significant (0.42 per cent *versus* 0.62 per cent; *P* = 0242). Multivariable analysis showed a higher risk of SSI for patients with multiple co-morbidities (OR = 1.663 (95 per cent c.i. 1.103 to 2.509); *P* = 0.015) or with larger defects (*P* = 0.035; that is W3 *versus* W1: OR = 2.084 (95 per cent c.i. 1.187 to 3.656); *P* = 0.010). Quality and risk-of-bias tables of these two cohort studies^172,173^ are reported in *Fig. 33*.

Combined analysis of the two included cohort studies^172,173^ revealed that antibiotic prophylaxis resulted in a statistically significant lower risk of postoperative SSI (two studies, 13 729 patients; 104 of 11 704 patients with prophylaxis (0.9 per cent) *versus* 39 of 2025 with no prophylaxis (1.9 per cent); OR 0.62 (95 per cent c.i. 0.42 to 0.93); *P* = 0.02; *I*^2^=0.33 per cent; fixed-effect model) (see *Fig. 33*).

#### Total and deep SSI rates—grouped analysis

The only included RCT^171^ showed no statistically significant benefit of the use of antibiotic prophylaxis on postoperative SSI rate (0/8 (0 per cent) *versus* 4/8 (50 per cent); OR 0.06 (95 per cent c.i. 0 to 1.3; *P* = 0.08; fixed-effect model).

**Key Question 13: (a) What information is important for patients after incisional hernia repair? and (b) What activities influence outcome?**

**Good Practice Statement A:** For patients having repair of a midline incisional hernia, the guidelines panel states that there is a lack of evidence-based information to provide patients with after surgery.

**Good Practice Statement B:** For patients having repair of a midline incisional hernia, the guidelines panel suggests: analgesia and dressing management should be as per local hospital policy; patients should be encouraged to actively mobilize and can do as they feel able (including sexual activity); patients should avoid heavy lifting/exercise (where they have to Valsalva) for 4 weeks (time for mesh ingrowth); patients should not swim in a public pool or the sea until the wound has healed (approximately 2 weeks) (however, can shower from day zero); patients can drive when they are able to safely perform an emergency stop without hesitation (advised to inform motor insurance company); and patients can be provided with an abdominal binder or compression clothes to wear for their comfort for first 6 weeks (advised to keep clean).

Postoperative instructions after incisional hernia repair vary depending on surgeon and at an institutional level. This KQ examines the evidence base for resuming normal activity or indeed restriction of activity after incisional hernia surgery.

### Search results

**Fig. 34:**
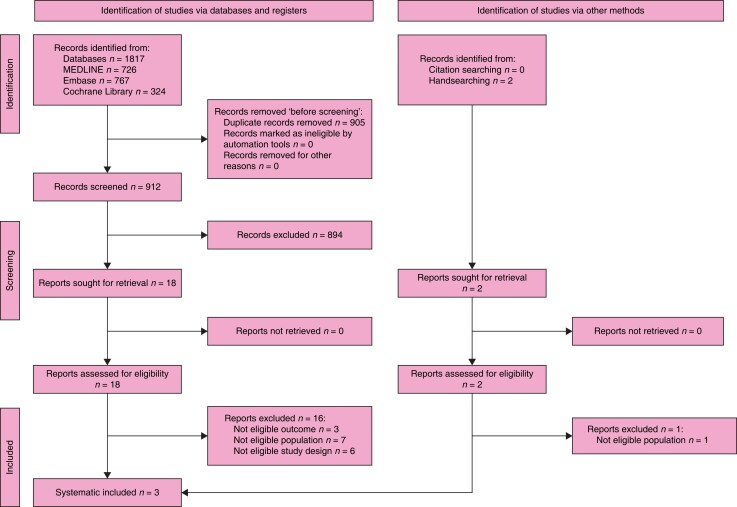
PRISMA flow diagram for Key Question 13

The search retrieved 1817 records. After the duplicates were removed, the titles and abstracts of 912 records were screened. A total of 18 reports were selected for full-text retrieval and were assessed for eligibility. A total of 16 reports were excluded and a total of two studies met the inclusion criteria. Moreover, handsearching identified another two reports whose full texts were evaluated for eligibility. As a result, three studies were included in the review. The full study selection process is presented in a PRISMA flow diagram (shown in *Fig. 34*).

The Summary of Findings is shown in *Table S13*.

### Evidence for Good Practice Statements A and B

A review of the literature provided insufficient high-quality data to help answer this KQ, making it difficult to establish rigorous evidence-based recommendations. As a result, a good practice statement was developed using a consensus of expert evidence provided by the guidelines panel. There were, however, some studies and surveys reviewed by the guidelines panel that provided information on the subject.

Whilst not specific to incisional hernia repair, one RCT^174^ analysed the effects of wearing an abdominal binder for 1 week after laparoscopic umbilical or epigastric hernia repair. No statistically significant differences were observed between the binder and non-binder groups; however, there was a lower 30-day complication rate in the binder group (0/28 *versus* 4/28– see *Table S14*). A subjective beneficial effect was also reported by 24 of the 28 patients (86 per cent, 95 per cent c.i.) in the binder group.

Recently, Schaff *et al.*^175^ published a survey including results from 127 expert hernia surgeons regarding how to manage postoperative strain and physical labour. They suggest that there is a lack of evidence, particularly regarding incisional hernias. Their survey results demonstrate that at least half of surgeons considered 4 weeks of reduced physical activity appropriate after an IPOM or retrorectus/sublay repair, but experts were far more divided regarding onlay mesh repairs or ‘complex’ repairs, where many believed 4 weeks to be insufficient.

Another survey of 48 surgeons from a German hospital group looked to gather expert opinion on the subject of postoperative rest after incisional hernia repair^176^. When asked about length of postoperative rest, 4 and 2 weeks were the most popular answers; however, substantial variation across the sample highlights the need for further research in this area.

After panel discussion, and considering the current literature, statements of expert evidence were provided by panel members to develop Good Practice Statement B.

## Discussion

### Key messages

The quantity and quality of evidence available to formulate the recommendations was limited; nevertheless, some key messages have been generated from the guidelines that, if followed, the guidelines panel believes will help improve outcomes in incisional hernia surgery. The main recommendations and good practice statements were that patients should undergo cross-sectional imaging before surgery to better understand the anatomy and appropriately plan the procedure. Surgeons and patients should understand that the main aim in treating incisional hernias is to improve the quality of life; this should help guide discussion of the benefits and risks of various treatment options to ensure patients are fully informed and are involved in the decision-making process. Patients should be pre-optimized before surgery with particular emphasis on weight loss, smoking cessation, and diabetic control. For the majority of patients, a mesh repair with fascial closure and the mesh in the retrorectus plane is recommended.

### Limitations

These European guidelines discuss the evidence base for the diagnosis and treatment of incisional hernias. The focus of the guidelines is on midline incisional hernias where it is anticipated that the fascial defect can be closed without any advanced procedure such as a component separation or any other form of myofascial release. The reason for this was that these are the most commonly encountered incisional hernias in surgical practice and therefore the largest evidence base would exist for this group. Despite placing these confines, the evidence in the literature both in terms of quantity and quality was very limited. This makes it impossible to formulate strong certainty recommendations for any of the KQs according to the GRADE methodology. Of particular interest, the majority of studies do not include any patient-reported outcome measures and there is significant variability in how clinical outcomes are assessed and defined. Furthermore, there is substantial discrepancy in the terminology used to discuss repair techniques and positions of mesh placement. Using uniform language and endpoints is important if a comparison is to be made between diagnostic or treatment modalities. Recent work has been done to provide rigorous definitions of abdominal wall planes^137^ and also to define a core outcome set for studies involving incisional hernia surgery^106^. The authors would strongly advocate the use of the standardized methods in research going forward.

Although the guidelines group aimed to represent all stakeholders and surgical specialties, it would have benefited from the participation of a plastic surgeon and a physiotherapist. Care was taken to create subgroups without group members who authored a paper relevant to the KQ or with other conflicts of interest. However, all group members are involved in hernia surgery and use meshes, which might have influenced the appraisal of the evidence and the formulation of recommendations. Efforts were made to have active patient participation, but, unfortunately, not all group meetings had patient representation. However, a patient representative critically reviewed the guidelines and their valuable comments were included.

### Implementation

To aid dissemination and implementation, the guidelines will be presented at international and national conferences, and summaries will be produced in different languages for national hernia organizations. The guidelines will also be presented on the GRADEpro website.

### Knowledge gaps

The guidelines have demonstrated the substantial gap in the evidence base for treatment of incisional hernias. Using uniform definitions and endpoints, including well-defined patient-reported outcome measures, which incorporate metrics that are important to patients and their quality of life, will be fundamental to closing this knowledge gap. Of particular value would be studies exploring what happens to patients during pre-optimization of weight and the impact that this has on outcomes. In keeping with this, it would also be useful to understand whether delaying procedures to pre-optimize can have a detrimental effect in terms of increasing hernia size and technical difficulties with repair. With major advances in minimally invasive techniques that allow closure of the fascial defect, high-quality studies comparing treatment techniques would be useful in ensuring that patients are offered optimal care. It may be that randomized trials do not provide the best methodology due to the length of follow-up required. Longitudinal cohorts making use of registry data may be more beneficial and easier to collect.

## Supplementary Material

znad284_Supplementary_Data

## Data Availability

The authors confirm that the data supporting the findings of this study are available within the article and/or the *Supplementary material*. Raw data for the forest plots are available on request.
