# Corrigendum to: Midline incisional hernia guidelines: the European Hernia Society

**DOI:** 10.1093/bjs/znad349

**Published:** 2023-10-26

This is a corrigendum to: David L Sanders, Maciej M Pawlak, Maarten P Simons, Theo Aufenacker, Andrea Balla, Cigdem Berger, Frederik Berrevoet, Andrew C de Beaux, Barbora East, Nadia A Henriksen, Miloslav Klugar, Alena Langaufová, Marc Miserez, Salvador Morales-Conde, Agneta Montgomery, Patrik K Pettersson, Wolfgang Reinpold, Yohann Renard, Simona Slezáková, Thomas Whitehead-Clarke, Cesare Stabilini, Midline incisional hernia guidelines: the European Hernia Society, British Journal of Surgery, 2023, znad284, https://doi.org/10.1093/bjs/znad284

In the originally published version of this manuscript, the affiliation for author Alena Langaufová was incorrect.

This has been corrected.

