# Supplementary material for: Midline incisional hernia guidelines: the European Hernia Society
Source: Br J Surg. 2023 Sep 19;110(12):1732–68. doi: 10.1093/bjs/znad284 (PMC10638550; doi:10.1093/bjs/znad284)
Supplement: znad284_Supplementary_Data [file znad284_supplementary_data.zip › Supplementary_materials.docx]

**Title:** Midline Incisional Hernia Guidelines: The European Hernia Society

**Authors:**

David L Sanders^1^ (United Kingdom)

Maciej M Pawlak^1^ (Poland)

Maarten P Simons^2^ (Netherlands)

Theo Aufenacker^3^ (Netherlands)

Andrea Balla^4^  (Italy)

Cigdem Berger^5^ (Germany)

Frederik Berrevoet^6^ (Belgium)

Andrew C de Beaux^7^ (United Kingdom)

Barbora East^8^  (Czech Republic)

Nadia A Henriksen^9^ (Denmark)

Miloslav Klugar^10^ (Czech Republic)

Alena Langaufová^10^ (Czech Republic)

Marc Miserez^11^ (Belgium)

Salvador Morales-Conde^12^ (Spain)

Agneta Montgomery^13^  (Sweden)

Patrik K Pettersson^13^ (Sweden)

Wolfgang Reinpold^14^ (Germany)

Yohann Renard^15^ (France)

Simona Slezáková^10^ (Czech Republic)

Thomas Whitehead-Clarke^16^ (United Kingdom)

Cesare Stabilini^17^ (Italy)

**Afilliations:**

1 a) Academic Department of Abdominal Wall Surgery

Royal Devon University Foundation Healthcare Trust

North Devon District Hospital

b) University of Exeter Medical School

2 OLVG Hospital Amsterdam

Department of Surgery

Oosterpark 7

1071 AC Amsterdam, Netherlands

3 Department of Surgery.

Rijnstate hospital Arnhem

Wagnerlaan 55

6815 AD Arnhem The Netherlands

4 IRCCS San Raffaele Scientific Institute

Via Olgettina, 60, 20132

Milan, Italy

5 Hamburg Hernia Center

Department of Hernia and Abdominal Wall Surgery

Helios Mariahilf Hospital Hamburg, Teaching Hospital of the University of Hamburg, Germany

6 Department for General and HPB Surgery and Liver Transplantation

Ghent University Hospital

Corneel Heymanslaan  10

9000 Gent, Belgium

7 Spire Murrayfield Hospital

Edinburgh, UK

EH12 6UD

8 3rd Department of Surgery at 1st Medical Faculty of Charles University

Motol University Hospital

V uvalu 84

Prague, Czech Republic

150 06

9 Dept. of Gastrointestinal and Hepatic Diseases

University of Copenhagen

Herlev Hospital

Denmark

10 The Czech National Centre for Evidence-Based Healthcare and Knowledge Translation (Cochrane Czech Republic, Czech CEBHC: JBI Centre of Excellence, Masaryk University GRADE Centre), Institute of Biostatistics and Analyses, Faculty of Medicine, Masaryk University, Brno, Czech Republic

11 Department of Abdominal Surgery

University Hospital Gasthuisberg, KULeuven,

Leuven, Belgium

12 Unit of Innovation in Minimally Invasive Surgery. Department of General and Digestive Surgery. University Hospital Virgen del Rocio

University of Sevilla (Spain)

Avd. Manuel Siurot s/n 41013 Sevilla

13 Department of Surgery Skåne University Hospital Malmö, Sweden

Department of Clinical Sciences, Malmö Faculty of Medicine Lund University Lund, Sweden

14 Abdominal Wall Surgery

Chairman and CEO Hamburg Hernia Center

Department of Hernia and Abdominal Wall Surgery

Helios Mariahilf Hospital Hamburg

Teaching Hospital of the University of Hamburg

Germany

15 Reims Champagne-Ardennes

Department of General, Digestive and Endocrine Surgery

Robert Debré University Hospital

Reims, France

16 Centre for 3D Models of Health and Disease

Division of Surgery and Interventional Science

University College London

43-45 Foley Street

London, UK

W1W7TY

17 a) University of Genoa, Department of Surgery, Genoa, Italy

b) Policlinico San Martino, IRCCS, Genoa, Italy

**Corresponding Author:** Prof. David L Sanders

Academic Department of Abdominal Wall Surgery

Royal Devon University Foundation Healthcare Trust

North Devon District Hospital

Raleigh Park

Barnstaple

EX31 4JB

dsanders3@nhs.net

**Supplementary Materials - Index**

| **Supplementary Figures and Tables** |  |
| --- | --- |

[TABLE S1: SEARCH STRATEGIES 6](#_Toc142290909)

[TABLE S2 SUMMARY OF FINDINGS FOR KQ1 144](#_Toc142290910)

[TABLE S3 SUMMARY OF FINDINGS FOR KQ2 146](#_Toc142290911)

[TABLE S4 SUMMARY OF FINDINGS FOR KQ3 152](#_Toc142290912)

[TABLE S5 SUMMARY OF FINDINGS FOR KQ4 154](#_Toc142290913)

[TABLE S7: SUMMARY OF FINDINGS FOR KQ6 156](#_Toc142290914)

[TABLE S8: SUMMARY OF FINDINGS FOR KQ7 159](#_Toc142290915)

[TABLE S9: SUMMARY OF FINDINGS FOR KQ8 162](#_Toc142290916)

[TABLE S9: SUMMARY OF FINDINGS FOR KQ9 166](#_Toc142290917)

[TABLE S10: SUMMARY OF FINDINGS FOR KQ10 168](#_Toc142290918)

[TABLE S11: SUMMARY OF FINDINGS FOR KQ11 170](#_Toc142290919)

[TABLE S12: SUMMARY OF FINDINGS FOR KQ12 172](#_Toc142290920)

[TABLE S13: SUMMARY OF FINDINGS FOR KQ13 173](#_Toc142290921)

TABLE S1: SEARCH STRATEGIES

1. **Guideline search**

The search was conducted in guideline databases, repositories or on the guideline developer's websites on July 24, 2020, using keywords hernia, hernias, and their equivalents in other languages.

**Databases, repositories and websites searched**

| Name | Website |
| --- | --- |
| Academy of Medicine of Malaysia | http://www.acadmed.org.my/index.cfm?menuid=67 |
| American Hernia Society | https://www.americanherniasociety.org/ |
| Arbeitsgemeinschaft der Wissenschaftlichen Medizinischen Fachgesellschaften (AWMF) | https://www.awmf.org/awmf-online-das-portal-der-wissenschaftlichen-medizin/awmf-aktuell.html |
| Australian Clinical Practice Guidelines | https://www.clinicalguidelines.gov.au/ |
| Belgian Health Care Knowledge Centre | https://kce.login.kanooh.be/en/publications/all-reports |
| Biblioteca de Guías de Práctica Clínica del Sistema Nacional de Salud (GuíaSalud) | http://portal.guiasalud.es/web/guest/guias-practica-clinica |
| BIGG international database of GRADE guidelinesBIGG base internacional de guías GRADE | http://sites.bvsalud.org/bigg/biblio/ |
| British Columbia Guidelines | https://www2.gov.bc.ca/gov/content/health/practitioner-professional-resources/bc-guidelines/guidelines-by-alphabetical-listing#D |
| British Hernia Society | https://www.britishherniasociety.org/ |
| CPG Infobase: Clinical Practice Guidelines | https://joulecma.ca/cpg/homepage |
| Domus medica Belgium | https://domusmedica.be/richtlijnen |
| Dynamed | https://www.dynamed.com/home/ |
| ECRI Institute Guideline Trust | https://guidelines.ecri.org/ |
| Epistemonikos GRADE guideline repository | https://www.epistemonikos.org/en/groups/grade_guideline |
| European Hernia Society | https://www.europeanherniasociety.eu/ |
| Guías de Práctica Clínica | https://guidelines.international/gpc |
| Guideline Central | https://www.guidelinecentral.com/ |
| Health Quality Ontario, Canada | https://www.hqontario.ca/Evidence-to-Improve-Care/Health-Technology-Assessment/Reviews-And-Recommendations |
| Institute for Clinical Systems Improvement (ICSI) | https://www.icsi.org/guidelines/ |
| International HTA Database | https://database.inahta.org/ |
| MAGICapp | https://app.magicapp.org/app#/guidelines |
| MaHTAS – Health Technology Assessment Section, Ministry of Health Malaysia | http://www.moh.gov.my/index.php/pages/view/135?mid=67 |
| Ministério da Sáudé, Brazil | http://www.saude.gov.br/protocolos-e-diretrizes |
| Ministerio de Salud, Chile | <https://diprece.minsal.cl/programas-de-salud/guias-clinicas/> |
| Ministerio de Salud, Columbia | http://gpc.minsalud.gov.co/gpc/SitePages/buscador_gpc.aspx |
| Ministerio de Salud, Peru | https://web.ins.gob.pe/salud-publica/publicaciones-unagesp/guias-de-practica-clinica |
| National Institute for Clinical Evidence (NICE) | https://www.nice.org.uk/guidance |
| National Patient Safety Office, Ireland | https://health.gov.ie/national-patient-safety-office/ncec/national-clinical-guidelines/ |
| Nederland Huisartsen Genootschap | https://www.nhg.org/nhg-standaarden |
| NIPH – Norwegian Institute of Public Health | https://www.fhi.no/en/qk/HTA/ |
| OSTEBA – Basque Office for Health Technology Assessment | http://www.euskadi.eus/information/clinical-practice-guidelines/web01-a3ikeost/en/ |
| Scottish Intercollegiate Guidelines Network (SIGN) | https://www.sign.ac.uk/ |
| The Canadian Task Force on Preventive Health Care | https://canadiantaskforce.ca/guidelines/published-guidelines/ |
| The GIN international guideline libraryGuidelines International Network (G-I-N) | https://g-i-n.net/international-guidelines-libraryhttp://www.g-i-n.net/ |
| Universidad Nacional de Colombia, Guías de Practica Clínica | https://unisalud.unal.edu.co/guias-practicas-clinica.html |
| Washington State Health Care Authority | https://www.hca.wa.gov/about-hca/health-technology-assessment |
| WHO | https://www.who.int/publications/guidelines/en/ |

1. **Systematic review search**

**Epistemonikos**

The search was conducted on 24^th^ July 2020.

***Databases screened by Epistemonikos****:*

- [Cochrane Database of Systematic Reviews (CDSR)](http://www.thecochranelibrary.com/) Last searched: Jul 24, 2020
- [Pubmed](http://www.ncbi.nlm.nih.gov/pubmed/) Last searched: Jul 24, 2020
- [EMBASE](https://www.elsevier.com/solutions/embase-biomedical-research) Last searched: Jul 17, 2020
- [CINAHL (The Cumulative Index to Nursing and Allied Health Literature)](https://www.ebscohost.com/nursing/products/cinahl-databases/the-cinahl-database) Last searched: Jul 17, 2020
- [PsycINFO](http://www.apa.org/pubs/databases/psycinfo/) Last searched: Jul 17, 2020
- [LILACS (Literatura Latinoamericana y del Caribe en Ciencias de la Salud)](http://lilacs.bvsalud.org/en/) Last searched: Jul 17, 2020
- [Database of Abstracts of Reviews of Effects (DARE)](http://www.crd.york.ac.uk/CRDWeb/) Last searched: Aug 24, 2017 - No new records have been added to DARE after 2015
- [The Campbell Collaboration online library](https://www.campbellcollaboration.org/better-evidence.html) Last searched: May 30, 2020
- [JBI Database of Systematic Reviews and Implementation Reports](http://journals.lww.com/jbisrir/pages/default.aspx) Last searched: Jun 1, 2020
- [EPPI-Centre Evidence Library](http://eppi.ioe.ac.uk/cms/Default.aspx?tabid=62) Last searched: Mar 9, 2020

| # | search string | # of results |
| --- | --- | --- |
| 1 | (title:("incisional hernia*" OR "cicatricial hernia*" OR "scar hernia*" OR "postoperative Hernia*" OR "post-operative Hernia*" OR "abdomen hernia*" OR "abdominal hernia*" OR "abdominal wall hernia*" OR "hernia* ventralis" OR "laparocele*" OR "ventral hernia*" OR "trocar hernia*" OR "port site hernia*") OR abstract:("incisional hernia*" OR "cicatricial hernia*" OR "scar hernia*" OR "postoperative Hernia*" OR "post-operative Hernia*" OR "abdomen hernia*" OR "abdominal hernia*" OR "abdominal wall hernia*" OR "hernia* ventralis" OR "laparocele*" OR "ventral hernia*" OR "trocar hernia*" OR "port site hernia*")) | 696 |
| 2 | #1 AND Systematic Reviews | 264 |

1. **MEDLINE (Ovid), Embase (Ovid) and Cochrane Library searches**

**KQ 1: What are risk factors for developing an incisional hernia after a previous abdominal surgery?**

**MEDLINE(R) ALL <1946 to March 12, 2021> (Ovid)**

**Search was conducted on 15^th^ March 2021.**

| # | search string | # of results |
| --- | --- | --- |
| 1 | (Randomized Controlled Trial or Controlled Clinical Trial or Pragmatic Clinical Trial or Equivalence Trial or Clinical Trial, Phase III).pt. | 617974 |
| 2 | Randomized Controlled Trial/ | 524960 |
| 3 | exp Randomized Controlled Trials as Topic/ | 144784 |
| 4 | Controlled Clinical Trial/ () | 94095 |
| 5 | exp Controlled Clinical Trials as Topic/ | 150269 |
| 6 | Randomization/ | 104832 |
| 7 | Random Allocation/ | 104832 |
| 8 | Double-Blind Method/ | 162912 |
| 9 | Double Blind Procedure/ | 0 |
| 10 | Double-Blind Studies/ | 162912 |
| 11 | Single-Blind Method/ | 29863 |
| 12 | Single Blind Procedure/ | 0 |
| 13 | Single-Blind Studies/ | 29863 |
| 14 | Placebos/ | 35374 |
| 15 | Placebo/ | 0 |
| 16 | Control Groups/ | 1725 |
| 17 | Control Group/ | 1725 |
| 18 | (random* or sham or placebo*).ti,ab,hw,kf,kw. | 1571724 |
| 19 | ((singl* or doubl*) adj (blind* or dumm* or mask*)).ti,ab,hw,kf,kw. | 244397 |
| 20 | ((tripl* or trebl*) adj (blind* or dumm* or mask*)).ti,ab,hw,kf,kw. | 1142 |
| 21 | (control* adj3 (study or studies or trial* or group*)).ti,ab,kf,kw. | 1038525 |
| 22 | (Nonrandom* or non random* or non-random* or quasi-random* or quasirandom*).ti,ab,hw,kf,kw. | 46508 |
| 23 | allocated.ti,ab,hw. | 69975 |
| 24 | ((open label or open-label) adj5 (study or studies or trial*)).ti,ab,hw,kf,kw. | 36939 |
| 25 | ((equivalence or superiority or non-inferiority or noninferiority) adj3 (study or studies or trial*)).ti,ab,hw,kf,kw. | 9217 |
| 26 | (pragmatic study or pragmatic studies).ti,ab,hw,kf,kw. | 451 |
| 27 | ((pragmatic or practical) adj3 trial*).ti,ab,hw,kf,kw. | 5816 |
| 28 | ((quasiexperimental or quasi-experimental) adj3 (study or studies or trial*)).ti,ab,hw,kf,kw. | 8734 |
| 29 | (phase adj3 (III or "3") adj3 (study or studies or trial*)).ti,hw,kf,kw. | 29992 |
| 30 | 1 or 2 or 3 or 4 or 5 or 6 or 7 or 8 or 9 or 10 or 11 or 12 or 13 or 14 or 15 or 16 or 17 or 18 or 19 or 20 or 21 or 22 or 23 or 24 or 25 or 26 or 27 or 28 or 29 | 2257829 |
| 31 | meta-analysis.pt. | 127955 |
| 32 | meta-analysis/ or systematic review/ or meta-analysis as topic/ or "meta analysis (topic)"/ or "systematic review (topic)"/ or exp technology assessment, biomedical/ | 241161 |
| 33 | ((systematic* adj3 (review* or overview*)) or (methodologic* adj3 (review* or overview*))).ti,ab,kf,kw. | 218882 |
| 34 | ((quantitative adj3 (review* or overview* or synthes*)) or (research adj3 (integrati* or overview*))).ti,ab,kf,kw. | 11951 |
| 35 | ((integrative adj3 (review* or overview*)) or (collaborative adj3 (review* or overview*)) or (pool* adj3 analy*)).ti,ab,kf,kw. | 29641 |
| 36 | (data synthes* or data extraction* or data abstraction*).ti,ab,kf,kw. | 30017 |
| 37 | (handsearch* or hand search*).ti,ab,kf,kw. | 9705 |
| 38 | (mantel haenszel or peto or der simonian or dersimonian or fixed effect* or latin square*).ti,ab,kf,kw. | 28434 |
| 39 | (met analy* or metanaly* or technology assessment* or HTA or HTAs or technology overview* or technology appraisal*).ti,ab,kf,kw. | 9958 |
| 40 | (meta regression* or metaregression*).ti,ab,kf,kw. | 10148 |
| 41 | (meta-analy* or metaanaly* or systematic review* or biomedical technology assessment* or bio-medical technology assessment*).mp,hw. | 342442 |
| 42 | (medline or cochrane or pubmed or medlars or embase or cinahl).ti,ab,hw. | 247915 |
| 43 | (cochrane or (health adj2 technology assessment) or evidence report).jw. | 19996 |
| 44 | (comparative adj3 (efficacy or effectiveness)).ti,ab,kf,kw. | 14417 |
| 45 | (outcomes research or relative effectiveness).ti,ab,kf,kw. | 9838 |
| 46 | ((indirect or indirect treatment or mixed-treatment) adj comparison*).ti,ab,kf,kw. | 2299 |
| 47 | 31 or 32 or 33 or 34 or 35 or 36 or 37 or 38 or 39 or 40 or 41 or 42 or 43 or 44 or 45 or 46 | 516309 |
| 48 | Case-Control Studies/ or Control Groups/ or Matched-Pair Analysis/ or ((case* adj5 control*) or (case adj3 comparison*) or control group*).ti,ab,kw. | 822747 |
| 49 | cohort studies/ or longitudinal studies/ or follow-up studies/ or prospective studies/ or retrospective studies/ or cohort.ti,ab. or longitudinal.ti,ab. or prospective.ti,ab. or retrospective.ti,ab. | 2794459 |
| 50 | (autobiography or bibliography or biography or case reports or classical article or letter or patient education handout or dictionary or directory or editorial or historical article or interactive tutorial or interview or introductory journal article or lecture or legal case or legislation or news or newspaper article or personal narrative or portrait or video audio media or webcast).pt. | 4310001 |
| 51 | exp animals/ not humans.sh. | 4799281 |
| 52 | (exp infant/ or exp child/ or adolescent/) not exp adult/ | 1918215 |
| 53 | 50 or 51 or 52 () | 10404345 |
| 54 | ((incisional or postoperative or post-operative or ventral) adj3 hernia?).ab,kf,kw,ti. () | 8404 |
| 55 | hernia, ventral/ or incisional hernia/ () | 7067 |
| 56 | 54 or 55 | 11263 |
| 57 | risk factors/ | 856432 |
| 58 | incidence/ | 272097 |
| 59 | ((risk or predictive) adj factor?).ab,kf,kw,ti. | 655863 |
| 60 | incidence?.ab,kf,kw,ti. | 800371 |
| 61 | (risk? adj3 (develop* or assessment?)).ab,kf,kw,ti. | 219627 |
| 62 | 57 or 58 or 59 or 60 or 61 | 2047762 |
| 63 | 56 and 62 | 2316 |
| 64 | 63 not 53 | 2067 |
| 65 | limit 64 to english language | 1860 |
| 66 | limit 65 to dt=20130301-20210315 | 1061 |
| 67 | 30 and 66 | 228 |
| 68 | 47 and 66 | 146 |
| 69 | 48 and 66 | 83 |
| 70 | 49 and 66 | 686 |

**Embase <1974 to 2021 March 12> (Ovid)**

**Search was conducted on 14^th^ March 2021.**

| # | search string | # of results |
| --- | --- | --- |
| 1 | (Randomized Controlled Trial or Controlled Clinical Trial or Pragmatic Clinical Trial or Equivalence Trial or Clinical Trial, Phase III).pt. | 0 |
| 2 | Randomized Controlled Trial/ | 651778 |
| 3 | exp Randomized Controlled Trials as Topic/ | 198855 |
| 4 | "Randomized Controlled Trial (topic)"/ | 198855 |
| 5 | Controlled Clinical Trial/ | 466838 |
| 6 | exp Controlled Clinical Trials as Topic/ | 206807 |
| 7 | "Controlled Clinical Trial (topic)"/ | 11531 |
| 8 | Randomization/ | 90619 |
| 9 | Random Allocation/ | 86809 |
| 10 | Double-Blind Method/ | 158430 |
| 11 | Double Blind Procedure/ | 182771 |
| 12 | Double-Blind Studies/ | 142095 |
| 13 | Single-Blind Method/ | 40318 |
| 14 | Single Blind Procedure/ | 42335 |
| 15 | Single-Blind Studies/ | 42335 |
| 16 | Placebos/ | 309399 |
| 17 | Placebo/ | 365195 |
| 18 | Control Groups/ | 110539 |
| 19 | Control Group/ | 110539 |
| 20 | (random* or sham or placebo*).ti,ab,hw,kw. | 2172603 |
| 21 | ((singl* or doubl*) adj (blind* or dumm* or mask*)).ti,ab,hw,kw. | 320093 |
| 22 | ((tripl* or trebl*) adj (blind* or dumm* or mask*)).ti,ab,hw,kw. | 1517 |
| 23 | (control* adj3 (study or studies or trial* or group*)).ti,ab,kw. | 1457801 |
| 24 | (Nonrandom* or non random* or non-random* or quasi-random* or quasirandom*).ti,ab,hw,kw. | 58796 |
| 25 | allocated.ti,ab,hw. | 90951 |
| 26 | ((open label or open-label) adj5 (study or studies or trial*)).ti,ab,hw,kw. | 68645 |
| 27 | ((equivalence or superiority or non-inferiority or noninferiority) adj3 (study or studies or trial*)).ti,ab,hw,kw. | 13672 |
| 28 | (pragmatic study or pragmatic studies).ti,ab,hw,kw. | 674 |
| 29 | ((pragmatic or practical) adj3 trial*).ti,ab,hw,kw. | 6189 |
| 30 | ((quasiexperimental or quasi-experimental) adj3 (study or studies or trial*)).ti,ab,hw,kw. | 14063 |
| 31 | (phase adj3 (III or "3") adj3 (study or studies or trial*)).ti,hw,kw. | 98966 |
| 32 | 1 or 2 or 3 or 4 or 5 or 6 or 7 or 8 or 9 or 10 or 11 or 12 or 13 or 14 or 15 or 16 or 17 or 18 or 19 or 20 or 21 or 22 or 23 or 24 or 25 or 26 or 27 or 28 or 29 or 30 or 31 | 3232963 |
| 33 | meta-analysis.pt. | 0 |
| 34 | meta-analysis/ or systematic review/ or meta-analysis as topic/ or "meta analysis (topic)"/ or "systematic review (topic)"/ or exp technology assessment, biomedical/ | 455174 |
| 35 | ((systematic* adj3 (review* or overview*)) or (methodologic* adj3 (review* or overview*))).ti,ab,kw. | 274697 |
| 36 | ((quantitative adj3 (review* or overview* or synthes*)) or (research adj3 (integrati* or overview*))).ti,ab,kw. | 14248 |
| 37 | ((integrative adj3 (review* or overview*)) or (collaborative adj3 (review* or overview*)) or (pool* adj3 analy*)).ti,ab,kw. | 42507 |
| 38 | (data synthes* or data extraction* or data abstraction*).ti,ab,kw. | 37317 |
| 39 | (handsearch* or hand search*).ti,ab,kw. | 11847 |
| 40 | (mantel haenszel or peto or der simonian or dersimonian or fixed effect* or latin square*).ti,ab,kw. | 37755 |
| 41 | (met analy* or metanaly* or technology assessment* or HTA or HTAs or technology overview* or technology appraisal*).ti,ab,kw. | 16298 |
| 42 | (meta regression* or metaregression*).ti,ab,kw. | 12758 |
| 43 | (meta-analy* or metaanaly* or systematic review* or biomedical technology assessment* or bio-medical technology assessment*).mp,hw. | 547838 |
| 44 | (medline or cochrane or pubmed or medlars or embase or cinahl).ti,ab,hw. | 328261 |
| 45 | (cochrane or (health adj2 technology assessment) or evidence report).jx. | 27593 |
| 46 | (comparative adj3 (efficacy or effectiveness)).ti,ab,kw. | 21142 |
| 47 | (outcomes research or relative effectiveness).ti,ab,kw. | 14232 |
| 48 | ((indirect or indirect treatment or mixed-treatment) adj comparison*).ti,ab,kw. | 4384 |
| 49 | 33 or 34 or 35 or 36 or 37 or 38 or 39 or 40 or 41 or 42 or 43 or 44 or 45 or 46 or 47 or 48 | 756660 |
| 50 | exp case control study/ or control group/ or statistical analysis/ or ((case* adj5 control*) or (case adj3 comparison*) or control group*).kw,ti,ab. | 1227352 |
| 51 | cohort analysis/ or longitudinal study/ or follow up/ or prospective study/ or retrospective study/ or cohort.ti,ab. or longitudinal.ti,ab. or prospective.ti,ab. or retrospective.ti,ab. | 4290705 |
| 52 | (conference abstract or "conference review" or editorial or letter or note or short survey).pt. | 7133520 |
| 53 | (exp animal/ or animal experiment/ or nonhuman/) not (exp human/ or human experiment/) | 6666363 |
| 54 | exp juvenile/ not exp adult/ | 2278466 |
| 55 | 52 or 53 or 54 | 14870312 |
| 56 | ((incisional or postoperative or post-operative or ventral) adj3 hernia?).ab,kw,ti. | 12011 |
| 57 | abdominal wall hernia/ or incisional hernia/ | 15742 |
| 58 | 56 or 57 | 18639 |
| 59 | risk factors/ | 714489 |
| 60 | incidence/ | 445911 |
| 61 | ((risk or predictive) adj factor?).ab,kw,ti. | 989982 |
| 62 | incidence?.ab,kw,ti. | 1152127 |
| 63 | (risk? adj3 (develop* or assessment?)).ab,kw,ti. | 322636 |
| 64 | 59 or 60 or 61 or 62 or 63 | 2610184 |
| 65 | 58 and 64 | 3952 |
| 66 | 65 not 55 | 2638 |
| 67 | limit 66 to english language | 2399 |
| 68 | limit 67 to dc=20130301-20210314 | 1372 |
| 69 | 32 and 68 | 285 |
| 70 | 49 and 68 | 167 |
| 71 | 50 and 68 | 90 |
| 72 | 51 and 68 | 955 |

**Cochrane Library**

[**Cochrane Database of Systematic Reviews**](https://www.cochranelibrary.com/)**, Issue 3 of 12, March 2021**

**Cochrane Central Register of Controlled Trials, Issue 3 of 12, March 2021**

**Search was conducted on 18^th^ March 2021.**

| **#** | **search string** | **# of results** |
| --- | --- | --- |
| **1** | ((incisional or postoperative or post-operative or ventral) NEAR/3 hernia?):ti,ab,kw | 1196 |
| **2** | MeSH descriptor: [Hernia, Ventral] this term only | 268 |
| **3** | MeSH descriptor: [Incisional Hernia] this term only | 127 |
| **4** | #1 or #2 or #3 | 1196 |
| **5** | MeSH descriptor: [Risk Factors] this term only | 24693 |
| **6** | MeSH descriptor: [Incidence] this term only | 10117 |
| **7** | ((risk or predictive) NEXT factor?):ti,ab,kw | 69351 |
| **8** | (incidence?):ti,ab,kw | 120743 |
| **9** | (risk? NEAR/3 (develop* or assessment?)):ti,ab,kw | 36575 |
| **10** | #5 or #6 or #7 or #8 or #9 | 199157 |
| **11** | #4 and #10 | 427 |
| **12** | #11 and Cochrane Database of Systematic Reviews Publication date 1.3.2013 - 18.3. 2021 | 2 |
| **13** | #11 and Cochrane Central Register of Controlled Trials Date added to CENTRAL trials database 1.3.2013 - 18.3. 2021 | 330 |

**KQ 2: Do all patients with an incisional hernia require imaging? What is the best modality?**

**MEDLINE(R) ALL <1946 to March 12, 2021> (Ovid)**

**Search was conducted on 15^th^ March 2021.**

| # | search string | # of results |
| --- | --- | --- |
| 1 | (Randomized Controlled Trial or Controlled Clinical Trial or Pragmatic Clinical Trial or Equivalence Trial or Clinical Trial, Phase III).pt. | 617974 |
| 2 | Randomized Controlled Trial/ | 524960 |
| 3 | exp Randomized Controlled Trials as Topic/ | 144784 |
| 4 | Controlled Clinical Trial/ | 94095 |
| 5 | exp Controlled Clinical Trials as Topic/ | 150269 |
| 6 | Randomization/ | 104832 |
| 7 | Random Allocation/ | 104832 |
| 8 | Double-Blind Method/ | 162912 |
| 9 | Double Blind Procedure/ | 0 |
| 10 | Double-Blind Studies/ | 162912 |
| 11 | Single-Blind Method/ | 29863 |
| 12 | Single Blind Procedure/ | 0 |
| 13 | Single-Blind Studies/ | 29863 |
| 14 | Placebos/ | 35374 |
| 15 | Placebo/ | 0 |
| 16 | Control Groups/ | 1725 |
| 17 | Control Group/ | 1725 |
| 18 | (random* or sham or placebo*).ti,ab,hw,kf,kw. | 1571724 |
| 19 | ((singl* or doubl*) adj (blind* or dumm* or mask*)).ti,ab,hw,kf,kw. | 244397 |
| 20 | ((tripl* or trebl*) adj (blind* or dumm* or mask*)).ti,ab,hw,kf,kw. | 1142 |
| 21 | (control* adj3 (study or studies or trial* or group*)).ti,ab,kf,kw. | 1038525 |
| 22 | (Nonrandom* or non random* or non-random* or quasi-random* or quasirandom*).ti,ab,hw,kf,kw. | 46508 |
| 23 | allocated.ti,ab,hw. | 69975 |
| 24 | ((open label or open-label) adj5 (study or studies or trial*)).ti,ab,hw,kf,kw. | 36939 |
| 25 | ((equivalence or superiority or non-inferiority or noninferiority) adj3 (study or studies or trial*)).ti,ab,hw,kf,kw. | 9217 |
| 26 | (pragmatic study or pragmatic studies).ti,ab,hw,kf,kw. | 451 |
| 27 | ((pragmatic or practical) adj3 trial*).ti,ab,hw,kf,kw. | 5816 |
| 28 | ((quasiexperimental or quasi-experimental) adj3 (study or studies or trial*)).ti,ab,hw,kf,kw. | 8734 |
| 29 | (phase adj3 (III or "3") adj3 (study or studies or trial*)).ti,hw,kf,kw. | 29992 |
| 30 | 1 or 2 or 3 or 4 or 5 or 6 or 7 or 8 or 9 or 10 or 11 or 12 or 13 or 14 or 15 or 16 or 17 or 18 or 19 or 20 or 21 or 22 or 23 or 24 or 25 or 26 or 27 or 28 or 29 | 2257829 |
| 31 | meta-analysis.pt. | 127955 |
| 32 | meta-analysis/ or systematic review/ or meta-analysis as topic/ or "meta analysis (topic)"/ or "systematic review (topic)"/ or exp technology assessment, biomedical/ | 241161 |
| 33 | ((systematic* adj3 (review* or overview*)) or (methodologic* adj3 (review* or overview*))).ti,ab,kf,kw. | 218882 |
| 34 | ((quantitative adj3 (review* or overview* or synthes*)) or (research adj3 (integrati* or overview*))).ti,ab,kf,kw. | 11951 |
| 35 | ((integrative adj3 (review* or overview*)) or (collaborative adj3 (review* or overview*)) or (pool* adj3 analy*)).ti,ab,kf,kw. | 29641 |
| 36 | (data synthes* or data extraction* or data abstraction*).ti,ab,kf,kw. | 30017 |
| 37 | (handsearch* or hand search*).ti,ab,kf,kw. | 9705 |
| 38 | (mantel haenszel or peto or der simonian or dersimonian or fixed effect* or latin square*).ti,ab,kf,kw. | 28434 |
| 39 | (met analy* or metanaly* or technology assessment* or HTA or HTAs or technology overview* or technology appraisal*).ti,ab,kf,kw. | 9958 |
| 40 | (meta regression* or metaregression*).ti,ab,kf,kw. | 10148 |
| 41 | (meta-analy* or metaanaly* or systematic review* or biomedical technology assessment* or bio-medical technology assessment*).mp,hw. | 342442 |
| 42 | (medline or cochrane or pubmed or medlars or embase or cinahl).ti,ab,hw. | 247915 |
| 43 | (cochrane or (health adj2 technology assessment) or evidence report).jw. | 19996 |
| 44 | (comparative adj3 (efficacy or effectiveness)).ti,ab,kf,kw. | 14417 |
| 45 | (outcomes research or relative effectiveness).ti,ab,kf,kw. | 9838 |
| 46 | ((indirect or indirect treatment or mixed-treatment) adj comparison*).ti,ab,kf,kw. | 2299 |
| 47 | 31 or 32 or 33 or 34 or 35 or 36 or 37 or 38 or 39 or 40 or 41 or 42 or 43 or 44 or 45 or 46 | 516309 |
| 48 | Case-Control Studies/ or Control Groups/ or Matched-Pair Analysis/ or ((case* adj5 control*) or (case adj3 comparison*) or control group*).ti,ab,kw. | 822747 |
| 49 | cohort studies/ or longitudinal studies/ or follow-up studies/ or prospective studies/ or retrospective studies/ or cohort.ti,ab. or longitudinal.ti,ab. or prospective.ti,ab. or retrospective.ti,ab. | 2794459 |
| 50 | (autobiography or bibliography or biography or case reports or classical article or letter or patient education handout or dictionary or directory or editorial or historical article or interactive tutorial or interview or introductory journal article or lecture or legal case or legislation or news or newspaper article or personal narrative or portrait or video audio media or webcast).pt. | 4310001 |
| 51 | exp animals/ not humans.sh. | 4799281 |
| 52 | (exp infant/ or exp child/ or adolescent/) not exp adult/ | 1918215 |
| 53 | 50 or 51 or 52 | 10404345 |
| 54 | ((incisional or postoperative or post-operative or ventral) adj3 hernia?).ab,kf,kw,ti. | 8404 |
| 55 | hernia, ventral/ or incisional hernia/ | 7067 |
| 56 | 54 or 55 | 11263 |
| 57 | imaging?.ab,kf,kw,ti. | 874548 |
| 58 | (compute* tomograph* or Compute* Assisted Tomograph* or compute* aided tomograph* or compute* axial tomograph* or CT or CAT or Electron Beam Tomograph*).ab,kf,kw,ti. | 652644 |
| 59 | (MRI or magnetic resonance or Magnetic Resonance or magnetization transfer imaging? or magnetization transfer imaging? or MR imaging? or MR tomograph* or NMR or NMRI).ab,kf,kw,ti. | 680865 |
| 60 | (ultrasound? or Ultrasonic or Ultrasonograph* or Echotomograph* or Echograph* or echoscop* or echosound? or sonograph* or sonogram? or ultrasonogram?).ab,kf,kw,ti. | 427639 |
| 61 | (scan? or scanning?).ab,kf,kw,ti. | 539389 |
| 62 | (Radiograph* or Radiology or radiological or electroradiograph* or radiogram? or ro?ntgenograph* or X Ray? or X-Ray?).ab,kf,kw,ti. | 759717 |
| 63 | exp Diagnostic Imaging/ | 2695843 |
| 64 | 57 or 58 or 59 or 60 or 61 or 62 or 63 | 4195903 |
| 65 | 56 and 64 | 1767 |
| 66 | 65 not 53 | 929 |
| 67 | limit 66 to english language | 810 |
| 68 | 30 and 67 | 110 |
| 69 | 47 and 67 | 28 |
| 70 | 48 and 67 | 33 |
| 71 | 49 and 67 | 450 |

**Embase <1974 to 2021 March 12> (Ovid)**

**Search was conducted on 15^th^ March 2021.**

| # | search string | # of results |
| --- | --- | --- |
| 1 | (Randomized Controlled Trial or Controlled Clinical Trial or Pragmatic Clinical Trial or Equivalence Trial or Clinical Trial, Phase III).pt. | 0 |
| 2 | Randomized Controlled Trial/ | 651778 |
| 3 | exp Randomized Controlled Trials as Topic/ | 198855 |
| 4 | "Randomized Controlled Trial (topic)"/ | 198855 |
| 5 | Controlled Clinical Trial/ | 466838 |
| 6 | exp Controlled Clinical Trials as Topic/ | 206807 |
| 7 | "Controlled Clinical Trial (topic)"/ | 11531 |
| 8 | Randomization/ | 90619 |
| 9 | Random Allocation/ | 86809 |
| 10 | Double-Blind Method/ | 158430 |
| 11 | Double Blind Procedure/ | 182771 |
| 12 | Double-Blind Studies/ | 142095 |
| 13 | Single-Blind Method/ | 40318 |
| 14 | Single Blind Procedure/ | 42335 |
| 15 | Single-Blind Studies/ | 42335 |
| 16 | Placebos/ | 309399 |
| 17 | Placebo/ | 365195 |
| 18 | Control Groups/ | 110539 |
| 19 | Control Group/ | 110539 |
| 20 | (random* or sham or placebo*).ti,ab,hw,kw. | 2172603 |
| 21 | ((singl* or doubl*) adj (blind* or dumm* or mask*)).ti,ab,hw,kw. | 320093 |
| 22 | ((tripl* or trebl*) adj (blind* or dumm* or mask*)).ti,ab,hw,kw. | 1517 |
| 23 | (control* adj3 (study or studies or trial* or group*)).ti,ab,kw. | 1457801 |
| 24 | (Nonrandom* or non random* or non-random* or quasi-random* or quasirandom*).ti,ab,hw,kw. | 58796 |
| 25 | allocated.ti,ab,hw. | 90951 |
| 26 | ((open label or open-label) adj5 (study or studies or trial*)).ti,ab,hw,kw. | 68645 |
| 27 | ((equivalence or superiority or non-inferiority or noninferiority) adj3 (study or studies or trial*)).ti,ab,hw,kw. | 13672 |
| 28 | (pragmatic study or pragmatic studies).ti,ab,hw,kw. | 674 |
| 29 | ((pragmatic or practical) adj3 trial*).ti,ab,hw,kw. | 6189 |
| 30 | ((quasiexperimental or quasi-experimental) adj3 (study or studies or trial*)).ti,ab,hw,kw. | 14063 |
| 31 | (phase adj3 (III or "3") adj3 (study or studies or trial*)).ti,hw,kw. | 98966 |
| 32 | 1 or 2 or 3 or 4 or 5 or 6 or 7 or 8 or 9 or 10 or 11 or 12 or 13 or 14 or 15 or 16 or 17 or 18 or 19 or 20 or 21 or 22 or 23 or 24 or 25 or 26 or 27 or 28 or 29 or 30 or 31 | 3232963 |
| 33 | meta-analysis.pt. | 0 |
| 34 | meta-analysis/ or systematic review/ or meta-analysis as topic/ or "meta analysis (topic)"/ or "systematic review (topic)"/ or exp technology assessment, biomedical/ | 455174 |
| 35 | ((systematic* adj3 (review* or overview*)) or (methodologic* adj3 (review* or overview*))).ti,ab,kw. | 274697 |
| 36 | ((quantitative adj3 (review* or overview* or synthes*)) or (research adj3 (integrati* or overview*))).ti,ab,kw. | 14248 |
| 37 | ((integrative adj3 (review* or overview*)) or (collaborative adj3 (review* or overview*)) or (pool* adj3 analy*)).ti,ab,kw. | 42507 |
| 38 | (data synthes* or data extraction* or data abstraction*).ti,ab,kw. | 37317 |
| 39 | (handsearch* or hand search*).ti,ab,kw. | 11847 |
| 40 | (mantel haenszel or peto or der simonian or dersimonian or fixed effect* or latin square*).ti,ab,kw. | 37755 |
| 41 | (met analy* or metanaly* or technology assessment* or HTA or HTAs or technology overview* or technology appraisal*).ti,ab,kw. | 16298 |
| 42 | (meta regression* or metaregression*).ti,ab,kw. | 12758 |
| 43 | (meta-analy* or metaanaly* or systematic review* or biomedical technology assessment* or bio-medical technology assessment*).mp,hw. | 547838 |
| 44 | (medline or cochrane or pubmed or medlars or embase or cinahl).ti,ab,hw. | 328261 |
| 45 | (cochrane or (health adj2 technology assessment) or evidence report).jx. | 27593 |
| 46 | (comparative adj3 (efficacy or effectiveness)).ti,ab,kw. | 21142 |
| 47 | (outcomes research or relative effectiveness).ti,ab,kw. | 14232 |
| 48 | ((indirect or indirect treatment or mixed-treatment) adj comparison*).ti,ab,kw. | 4384 |
| 49 | 33 or 34 or 35 or 36 or 37 or 38 or 39 or 40 or 41 or 42 or 43 or 44 or 45 or 46 or 47 or 48 | 756660 |
| 50 | exp case control study/ or control group/ or statistical analysis/ or ((case* adj5 control*) or (case adj3 comparison*) or control group*).ti,ab,kw. | 1227352 |
| 51 | cohort analysis/ or longitudinal study/ or follow up/ or prospective study/ or retrospective study/ or cohort.ti,ab. or longitudinal.ti,ab. or prospective.ti,ab. or retrospective.ti,ab. | 4290705 |
| 52 | (conference abstract or "conference review" or editorial or letter or note or short survey).pt. | 7133520 |
| 53 | (exp animal/ or animal experiment/ or nonhuman/) not (exp human/ or human experiment/) | 6666363 |
| 54 | exp juvenile/ not exp adult/ | 2278466 |
| 55 | 52 or 53 or 54 | 14870312 |
| 56 | ((incisional or postoperative or post-operative or ventral) adj3 hernia?).ab,kw,ti. | 12011 |
| 57 | abdominal wall hernia/ or incisional hernia/ | 15742 |
| 58 | 56 or 57 | 18639 |
| 59 | imaging?.ab,kw,ti. | 1240861 |
| 60 | (compute* tomograph* or Compute* Assisted Tomograph* or compute* aided tomograph* or compute* axial tomograph* or CT or CAT or Electron Beam Tomograph*).ab,kw,ti. | 967474 |
| 61 | (MRI or magnetic resonance or Magnetic Resonance or magnetization transfer imaging? or magnetization transfer imaging? or MR imaging? or MR tomograph* or NMR or NMRI).ab,kw,ti. | 966311 |
| 62 | (ultrasound? or Ultrasonic or Ultrasonograph* or Echotomograph* or Echograph* or echoscop* or echosound? or sonograph* or sonogram? or ultrasonogram?).ab,kw,ti. | 631647 |
| 63 | (scan? or scanning?).ab,kw,ti. | 738529 |
| 64 | (Radiograph* or Radiology or radiological or electroradiograph* or radiogram? or ro?ntgenograph* or X Ray? or X-Ray?).ab,kw,ti. | 908017 |
| 65 | exp diagnostic imaging/ | 206095 |
| 66 | exp computer assisted tomography/ | 1128268 |
| 67 | exp nuclear magnetic resonance imaging/ | 1014144 |
| 68 | exp echography/ | 830048 |
| 69 | exp radiography/ | 1147080 |
| 70 | 59 or 60 or 61 or 62 or 63 or 64 or 65 or 66 or 67 or 68 or 69 | 4998604 |
| 71 | 58 and 70 | 4170 |
| 72 | 71 not 55 | 2753 |
| 73 | limit 72 to english language | 2474 |
| 74 | 32 and 73 | 194 |
| 75 | 49 and 73 | 69 |
| 76 | 50 and 73 | 55 |
| 77 | 51 and 73 | 1012 |

**Cochrane Library**

[**Cochrane Database of Systematic Reviews**](https://www.cochranelibrary.com/)**, Issue 3 of 12, March 2021**

**Cochrane Central Register of Controlled Trials, Issue 3 of 12, March 2021**

**Search was conducted on 18^th^ March 2021.**

| **#** | **search string** | **# of results** |
| --- | --- | --- |
| **1** | ((incisional or postoperative or post-operative or ventral) NEAR/3 hernia?):ti,ab,kw | 1196 |
| **2** | MeSH descriptor: [Hernia, Ventral] this term only | 268 |
| **3** | MeSH descriptor: [Incisional Hernia] this term only | 127 |
| **4** | #1 or #2 or #3 | 1196 |
| **5** | (imaging?):ti,ab,kw | 69020 |
| **6** | ((compute* NEXT tomograph*) OR (Compute* NEXT Assisted NEXT Tomograph*) OR (compute* NEXT aided NEXT tomograph*) OR (compute* NEXT axial NEXT tomograph*) OR (Electron NEXT Beam NEXT Tomograph*) OR CT or CAT):ti,ab,kw | 89159 |
| **7** | ((magnetic NEXT resonance) OR (magnetization NEXT transfer NEXT imaging?) OR (MR NEXT imaging?) OR (MR NEXT tomograph*) OR NMR OR NMRI OR MRI):ti,ab,kw | 37183 |
| **8** | (ultrasound? or Ultrasonic or Ultrasonograph* or Echotomograph* or Echograph* or echoscop* or echosound? or sonograph* or sonogram? or ultrasonogram?):ti,ab,kw | 45455 |
| **9** | (scan? or scanning?):ti,ab,kw | 26959 |
| **10** | (Radiograph* or Radiology or radiological or electroradiograph* or radiogram? or ro?ntgenograph* or X-Ray? or (X NEXT Ray?)):ti,ab,kw | 46411 |
| **11** | MeSH descriptor: [Diagnostic Imaging] explode all trees | 47919 |
| **12** | #5 or #6 or #7 or #8 or #9 or #10 or #11 | 223742 |
| **13** | #12 AND Cochrane Database of Systematic Reviews | 0 |
| **14** | #12 AND Cochrane Central Register of Controlled Trials | 236 |

**KQ 3: Is it possible to predict from imaging whether the fascial closure will be possible?**

**MEDLINE(R) ALL <1946 to March 12, 2021> (Ovid)**

**Search was conducted on 15^th^ March 2021.**

| # | search string | # of results |
| --- | --- | --- |
| 1 | (Randomized Controlled Trial or Controlled Clinical Trial or Pragmatic Clinical Trial or Equivalence Trial or Clinical Trial, Phase III).pt. | 617974 |
| 2 | Randomized Controlled Trial/ | 524960 |
| 3 | exp Randomized Controlled Trials as Topic/ | 144784 |
| 4 | Controlled Clinical Trial/ | 94095 |
| 5 | exp Controlled Clinical Trials as Topic/ | 150269 |
| 6 | Randomization/ | 104832 |
| 7 | Random Allocation/ | 104832 |
| 8 | Double-Blind Method/ | 162912 |
| 9 | Double Blind Procedure/ | 0 |
| 10 | Double-Blind Studies/ | 162912 |
| 11 | Single-Blind Method/ | 29863 |
| 12 | Single Blind Procedure/ | 0 |
| 13 | Single-Blind Studies/ | 29863 |
| 14 | Placebos/ | 35374 |
| 15 | Placebo/ | 0 |
| 16 | Control Groups/ | 1725 |
| 17 | Control Group/ | 1725 |
| 18 | (random* or sham or placebo*).ti,ab,hw,kf,kw. | 1571724 |
| 19 | ((singl* or doubl*) adj (blind* or dumm* or mask*)).ti,ab,hw,kf,kw. | 244397 |
| 20 | ((tripl* or trebl*) adj (blind* or dumm* or mask*)).ti,ab,hw,kf,kw. | 1142 |
| 21 | (control* adj3 (study or studies or trial* or group*)).ti,ab,kf,kw. | 1038525 |
| 22 | (Nonrandom* or non random* or non-random* or quasi-random* or quasirandom*).ti,ab,hw,kf,kw. | 46508 |
| 23 | allocated.ti,ab,hw. | 69975 |
| 24 | ((open label or open-label) adj5 (study or studies or trial*)).ti,ab,hw,kf,kw. | 36939 |
| 25 | ((equivalence or superiority or non-inferiority or noninferiority) adj3 (study or studies or trial*)).ti,ab,hw,kf,kw. | 9217 |
| 26 | (pragmatic study or pragmatic studies).ti,ab,hw,kf,kw. | 451 |
| 27 | ((pragmatic or practical) adj3 trial*).ti,ab,hw,kf,kw. | 5816 |
| 28 | ((quasiexperimental or quasi-experimental) adj3 (study or studies or trial*)).ti,ab,hw,kf,kw. | 8734 |
| 29 | (phase adj3 (III or "3") adj3 (study or studies or trial*)).ti,hw,kf,kw. | 29992 |
| 30 | 1 or 2 or 3 or 4 or 5 or 6 or 7 or 8 or 9 or 10 or 11 or 12 or 13 or 14 or 15 or 16 or 17 or 18 or 19 or 20 or 21 or 22 or 23 or 24 or 25 or 26 or 27 or 28 or 29 | 2257829 |
| 31 | meta-analysis.pt. | 127955 |
| 32 | meta-analysis/ or systematic review/ or meta-analysis as topic/ or "meta analysis (topic)"/ or "systematic review (topic)"/ or exp technology assessment, biomedical/ | 241161 |
| 33 | ((systematic* adj3 (review* or overview*)) or (methodologic* adj3 (review* or overview*))).ti,ab,kf,kw. | 218882 |
| 34 | ((quantitative adj3 (review* or overview* or synthes*)) or (research adj3 (integrati* or overview*))).ti,ab,kf,kw. | 11951 |
| 35 | ((integrative adj3 (review* or overview*)) or (collaborative adj3 (review* or overview*)) or (pool* adj3 analy*)).ti,ab,kf,kw. | 29641 |
| 36 | (data synthes* or data extraction* or data abstraction*).ti,ab,kf,kw. | 30017 |
| 37 | (handsearch* or hand search*).ti,ab,kf,kw. | 9705 |
| 38 | (mantel haenszel or peto or der simonian or dersimonian or fixed effect* or latin square*).ti,ab,kf,kw. | 28434 |
| 39 | (met analy* or metanaly* or technology assessment* or HTA or HTAs or technology overview* or technology appraisal*).ti,ab,kf,kw. | 9958 |
| 40 | (meta regression* or metaregression*).ti,ab,kf,kw. | 10148 |
| 41 | (meta-analy* or metaanaly* or systematic review* or biomedical technology assessment* or bio-medical technology assessment*).mp,hw. | 342442 |
| 42 | (medline or cochrane or pubmed or medlars or embase or cinahl).ti,ab,hw. | 247915 |
| 43 | (cochrane or (health adj2 technology assessment) or evidence report).jw. | 19996 |
| 44 | (comparative adj3 (efficacy or effectiveness)).ti,ab,kf,kw. | 14417 |
| 45 | (outcomes research or relative effectiveness).ti,ab,kf,kw. | 9838 |
| 46 | ((indirect or indirect treatment or mixed-treatment) adj comparison*).ti,ab,kf,kw. | 2299 |
| 47 | 31 or 32 or 33 or 34 or 35 or 36 or 37 or 38 or 39 or 40 or 41 or 42 or 43 or 44 or 45 or 46 | 516309 |
| 48 | Case-Control Studies/ or Control Groups/ or Matched-Pair Analysis/ or ((case* adj5 control*) or (case adj3 comparison*) or control group*).ti,ab,kw. | 822747 |
| 49 | cohort studies/ or longitudinal studies/ or follow-up studies/ or prospective studies/ or retrospective studies/ or cohort.ti,ab. or longitudinal.ti,ab. or prospective.ti,ab. or retrospective.ti,ab. | 2794459 |
| 50 | (autobiography or bibliography or biography or case reports or classical article or letter or patient education handout or dictionary or directory or editorial or historical article or interactive tutorial or interview or introductory journal article or lecture or legal case or legislation or news or newspaper article or personal narrative or portrait or video audio media or webcast).pt. | 4310001 |
| 51 | exp animals/ not humans.sh. | 4799281 |
| 52 | (exp infant/ or exp child/ or adolescent/) not exp adult/ | 1918215 |
| 53 | 50 or 51 or 52 | 10404345 |
| 54 | ((incisional or postoperative or post-operative or ventral) adj3 hernia?).ab,kf,kw,ti. | 8404 |
| 55 | hernia, ventral/ or incisional hernia/ | 7067 |
| 56 | 54 or 55 | 11263 |
| 57 | imaging?.ab,kf,kw,ti. | 874548 |
| 58 | (compute* tomograph* or Compute* Assisted Tomograph* or compute* aided tomograph* or compute* axial tomograph* or CT or CAT or Electron Beam Tomograph*).ab,kf,kw,ti. | 652644 |
| 59 | (MRI or magnetic resonance or Magnetic Resonance or magnetization transfer imaging? or magnetization transfer imaging? or MR imaging? or MR tomograph* or NMR or NMRI).ab,kf,kw,ti. | 680865 |
| 60 | (ultrasound? or Ultrasonic or Ultrasonograph* or Echotomograph* or Echograph* or echoscop* or echosound? or sonograph* or sonogram? or ultrasonogram?).ab,kf,kw,ti. | 427639 |
| 61 | (scan? or scanning?).ab,kf,kw,ti. | 539389 |
| 62 | (Radiograph* or Radiology or radiological or electroradiograph* or radiogram? or ro?ntgenograph* or X Ray? or X-Ray?).ab,kf,kw,ti. | 759717 |
| 63 | exp Diagnostic Imaging/ | 2695843 |
| 64 | 57 or 58 or 59 or 60 or 61 or 62 or 63 | 4195903 |
| 65 | ((fascia? or gap? or defect? or sheath?) and (closure? or opposition? or close? or oppose? or approximat*)).ab,kf,kw,ti. | 72479 |
| 66 | (loss of domain or LOD).ab,kf,kw,ti. | 22290 |
| 67 | 65 or 66 | 94633 |
| 68 | 56 and 64 and 67 | 194 |
| 69 | 68 not 53 | 144 |
| 70 | limit 69 to english language | 136 |
| 71 | 30 and 70 | 21 |
| 72 | 47 and 70 | 7 |
| 73 | 48 and 70 | 3 |
| 74 | 49 and 70 | 88 |

**Embase <1974 to 2021 March 12> (Ovid)**

**Search was conducted on 15^th^ March 2021.**

| # | search string | # of results |
| --- | --- | --- |
| 1 | (Randomized Controlled Trial or Controlled Clinical Trial or Pragmatic Clinical Trial or Equivalence Trial or Clinical Trial, Phase III).pt. | 0 |
| 2 | Randomized Controlled Trial/ | 651778 |
| 3 | exp Randomized Controlled Trials as Topic/ | 198855 |
| 4 | "Randomized Controlled Trial (topic)"/ | 198855 |
| 5 | Controlled Clinical Trial/ | 466838 |
| 6 | exp Controlled Clinical Trials as Topic/ | 206807 |
| 7 | "Controlled Clinical Trial (topic)"/ | 11531 |
| 8 | Randomization/ | 90619 |
| 9 | Random Allocation/ | 86809 |
| 10 | Double-Blind Method/ | 158430 |
| 11 | Double Blind Procedure/ | 182771 |
| 12 | Double-Blind Studies/ | 142095 |
| 13 | Single-Blind Method/ | 40318 |
| 14 | Single Blind Procedure/ | 42335 |
| 15 | Single-Blind Studies/ | 42335 |
| 16 | Placebos/ | 309399 |
| 17 | Placebo/ | 365195 |
| 18 | Control Groups/ | 110539 |
| 19 | Control Group/ | 110539 |
| 20 | (random* or sham or placebo*).ti,ab,hw,kw. | 2172603 |
| 21 | ((singl* or doubl*) adj (blind* or dumm* or mask*)).ti,ab,hw,kw. | 320093 |
| 22 | ((tripl* or trebl*) adj (blind* or dumm* or mask*)).ti,ab,hw,kw. | 1517 |
| 23 | (control* adj3 (study or studies or trial* or group*)).ti,ab,kw. | 1457801 |
| 24 | (Nonrandom* or non random* or non-random* or quasi-random* or quasirandom*).ti,ab,hw,kw. | 58796 |
| 25 | allocated.ti,ab,hw. | 90951 |
| 26 | ((open label or open-label) adj5 (study or studies or trial*)).ti,ab,hw,kw. | 68645 |
| 27 | ((equivalence or superiority or non-inferiority or noninferiority) adj3 (study or studies or trial*)).ti,ab,hw,kw. | 13672 |
| 28 | (pragmatic study or pragmatic studies).ti,ab,hw,kw. | 674 |
| 29 | ((pragmatic or practical) adj3 trial*).ti,ab,hw,kw. | 6189 |
| 30 | ((quasiexperimental or quasi-experimental) adj3 (study or studies or trial*)).ti,ab,hw,kw. | 14063 |
| 31 | (phase adj3 (III or "3") adj3 (study or studies or trial*)).ti,hw,kw. | 98966 |
| 32 | 1 or 2 or 3 or 4 or 5 or 6 or 7 or 8 or 9 or 10 or 11 or 12 or 13 or 14 or 15 or 16 or 17 or 18 or 19 or 20 or 21 or 22 or 23 or 24 or 25 or 26 or 27 or 28 or 29 or 30 or 31 | 3232963 |
| 33 | meta-analysis.pt. | 0 |
| 34 | meta-analysis/ or systematic review/ or meta-analysis as topic/ or "meta analysis (topic)"/ or "systematic review (topic)"/ or exp technology assessment, biomedical/ | 455174 |
| 35 | ((systematic* adj3 (review* or overview*)) or (methodologic* adj3 (review* or overview*))).ti,ab,kw. | 274697 |
| 36 | ((quantitative adj3 (review* or overview* or synthes*)) or (research adj3 (integrati* or overview*))).ti,ab,kw. | 14248 |
| 37 | ((integrative adj3 (review* or overview*)) or (collaborative adj3 (review* or overview*)) or (pool* adj3 analy*)).ti,ab,kw. | 42507 |
| 38 | (data synthes* or data extraction* or data abstraction*).ti,ab,kw. | 37317 |
| 39 | (handsearch* or hand search*).ti,ab,kw. | 11847 |
| 40 | (mantel haenszel or peto or der simonian or dersimonian or fixed effect* or latin square*).ti,ab,kw. | 37755 |
| 41 | (met analy* or metanaly* or technology assessment* or HTA or HTAs or technology overview* or technology appraisal*).ti,ab,kw. | 16298 |
| 42 | (meta regression* or metaregression*).ti,ab,kw. | 12758 |
| 43 | (meta-analy* or metaanaly* or systematic review* or biomedical technology assessment* or bio-medical technology assessment*).mp,hw. | 547838 |
| 44 | (medline or cochrane or pubmed or medlars or embase or cinahl).ti,ab,hw. | 328261 |
| 45 | (cochrane or (health adj2 technology assessment) or evidence report).jx. | 27593 |
| 46 | (comparative adj3 (efficacy or effectiveness)).ti,ab,kw. | 21142 |
| 47 | (outcomes research or relative effectiveness).ti,ab,kw. | 14232 |
| 48 | ((indirect or indirect treatment or mixed-treatment) adj comparison*).ti,ab,kw. | 4384 |
| 49 | 33 or 34 or 35 or 36 or 37 or 38 or 39 or 40 or 41 or 42 or 43 or 44 or 45 or 46 or 47 or 48 | 756660 |
| 50 | exp case control study/ or control group/ or statistical analysis/ or ((case* adj5 control*) or (case adj3 comparison*) or control group*).ti,kw,ab. | 1227352 |
| 51 | cohort analysis/ or longitudinal study/ or follow up/ or prospective study/ or retrospective study/ or cohort.ti,ab. or longitudinal.ti,ab. or prospective.ti,ab. or retrospective.ti,ab. | 4290705 |
| 52 | (conference abstract or "conference review" or editorial or letter or note or short survey).pt. | 7133520 |
| 53 | (exp animal/ or animal experiment/ or nonhuman/) not (exp human/ or human experiment/) | 6666363 |
| 54 | exp juvenile/ not exp adult/ | 2278466 |
| 55 | 52 or 53 or 54 | 14870312 |
| 56 | ((incisional or postoperative or post-operative or ventral) adj3 hernia?).ab,kw,ti. | 12011 |
| 57 | abdominal wall hernia/ or incisional hernia/ | 15742 |
| 58 | 56 or 57 | 18639 |
| 59 | imaging?.ab,kw,ti. | 1240861 |
| 60 | (compute* tomograph* or Compute* Assisted Tomograph* or compute* aided tomograph* or compute* axial tomograph* or CT or CAT or Electron Beam Tomograph*).ab,kw,ti. | 967474 |
| 61 | (MRI or magnetic resonance or Magnetic Resonance or magnetization transfer imaging? or magnetization transfer imaging? or MR imaging? or MR tomograph* or NMR or NMRI).ab,kw,ti. | 966311 |
| 62 | (ultrasound? or Ultrasonic or Ultrasonograph* or Echotomograph* or Echograph* or echoscop* or echosound? or sonograph* or sonogram? or ultrasonogram?).ab,kw,ti. | 631647 |
| 63 | (scan? or scanning?).ab,kw,ti. | 738529 |
| 64 | (Radiograph* or Radiology or radiological or electroradiograph* or radiogram? or ro?ntgenograph* or X Ray? or X-Ray?).ab,kw,ti. | 908017 |
| 65 | exp diagnostic imaging/ | 206095 |
| 66 | exp computer assisted tomography/ | 1128268 |
| 67 | exp nuclear magnetic resonance imaging/ | 1014144 |
| 68 | exp echography/ | 830048 |
| 69 | exp radiography/ | 1147080 |
| 70 | 59 or 60 or 61 or 62 or 63 or 64 or 65 or 66 or 67 or 68 or 69 | 4998604 |
| 71 | ((fascia? or gap? or defect? or sheath?) and (closure? or opposition? or close? or oppose? or approximat*)).ab,kw,ti. | 91573 |
| 72 | (loss of domain or LOD).ab,kw,ti. | 28053 |
| 73 | 71 or 72 | 119452 |
| 74 | 58 and 70 and 73 | 479 |
| 75 | 74 not 55 | 265 |
| 76 | limit 75 to english language | 240 |
| 77 | 32 and 76 | 26 |
| 78 | 49 and 76 | 9 |
| 79 | 50 and 76 | 3 |
| 80 | 51 and 76 | 122 |

**Cochrane Library**

[**Cochrane Database of Systematic Reviews**](https://www.cochranelibrary.com/)**, Issue 3 of 12, March 2021**

**Cochrane Central Register of Controlled Trials, Issue 3 of 12, March 2021**

**Search was conducted on 18^th^ March 2021.**

| **#** | **search string** | **# of results** |
| --- | --- | --- |
| **1** | ((incisional or postoperative or post-operative or ventral) NEAR/3 hernia?):ti,ab,kw | 1196 |
| **2** | MeSH descriptor: [Hernia, Ventral] this term only | 268 |
| **3** | MeSH descriptor: [Incisional Hernia] this term only | 127 |
| **4** | #1 or #2 or #3 | 1196 |
| **5** | (imaging?):ti,ab,kw | 69020 |
| **6** | ((compute* NEXT tomograph*) OR (Compute* NEXT Assisted NEXT Tomograph*) OR (compute* NEXT aided NEXT tomograph*) OR (compute* NEXT axial NEXT tomograph*) OR (Electron NEXT Beam NEXT Tomograph*) OR CT or CAT):ti,ab,kw | 89159 |
| **7** | ((magnetic NEXT resonance) OR (magnetization NEXT transfer NEXT imaging?) OR (MR NEXT imaging?) OR (MR NEXT tomograph*) OR NMR OR NMRI OR MRI):ti,ab,kw | 37183 |
| **8** | (ultrasound? or Ultrasonic or Ultrasonograph* or Echotomograph* or Echograph* or echoscop* or echosound? or sonograph* or sonogram? or ultrasonogram?):ti,ab,kw | 45455 |
| **9** | (scan? or scanning?):ti,ab,kw | 26959 |
| **10** | (Radiograph* or Radiology or radiological or electroradiograph* or radiogram? or ro?ntgenograph* or X-Ray? or (X NEXT Ray?)):ti,ab,kw | 46411 |
| **11** | MeSH descriptor: [Diagnostic Imaging] explode all trees | 47919 |
| **12** | #5 OR #6 OR #7 OR #8 OR #9 OR #10 OR #11 | 223742 |
| **13** | ((fascia? or gap? or defect? or sheath?) and (closure? or opposition? or close? or oppose? or approximat*)):ti,ab,kw | 3746 |
| **14** | ("loss of domain" or LOD):ti,ab,kw | 237 |
| **15** | #13 or #14 | 3981 |
| **16** | #4 and #12 and #15 | 51 |
| **17** | #16 AND Cochrane Database of Systematic Reviews | 0 |
| **18** | #16 AND Cochrane Central Register of Controlled Trials | 51 |

**KQ 4: Do all incisional hernias need surgical treatment?**

**MEDLINE(R) ALL <1946 to March 12, 2021> (Ovid)**

**Search was conducted on 15^th^ March 2021.**

| # | search string | # of results |
| --- | --- | --- |
| 1 | (Randomized Controlled Trial or Controlled Clinical Trial or Pragmatic Clinical Trial or Equivalence Trial or Clinical Trial, Phase III).pt. | 617974 |
| 2 | Randomized Controlled Trial/ | 524960 |
| 3 | exp Randomized Controlled Trials as Topic/ | 144784 |
| 4 | Controlled Clinical Trial/ | 94095 |
| 5 | exp Controlled Clinical Trials as Topic/ | 150269 |
| 6 | Randomization/ | 104832 |
| 7 | Random Allocation/ | 104832 |
| 8 | Double-Blind Method/ | 162912 |
| 9 | Double Blind Procedure/ | 0 |
| 10 | Double-Blind Studies/ | 162912 |
| 11 | Single-Blind Method/ | 29863 |
| 12 | Single Blind Procedure/ | 0 |
| 13 | Single-Blind Studies/ | 29863 |
| 14 | Placebos/ | 35374 |
| 15 | Placebo/ | 0 |
| 16 | Control Groups/ | 1725 |
| 17 | Control Group/ | 1725 |
| 18 | (random* or sham or placebo*).ti,ab,hw,kf,kw. | 1571724 |
| 19 | ((singl* or doubl*) adj (blind* or dumm* or mask*)).ti,ab,hw,kf,kw. | 244397 |
| 20 | ((tripl* or trebl*) adj (blind* or dumm* or mask*)).ti,ab,hw,kf,kw. | 1142 |
| 21 | (control* adj3 (study or studies or trial* or group*)).ti,ab,kf,kw. | 1038525 |
| 22 | (Nonrandom* or non random* or non-random* or quasi-random* or quasirandom*).ti,ab,hw,kf,kw. | 46508 |
| 23 | allocated.ti,ab,hw. | 69975 |
| 24 | ((open label or open-label) adj5 (study or studies or trial*)).ti,ab,hw,kf,kw. | 36939 |
| 25 | ((equivalence or superiority or non-inferiority or noninferiority) adj3 (study or studies or trial*)).ti,ab,hw,kf,kw. | 9217 |
| 26 | (pragmatic study or pragmatic studies).ti,ab,hw,kf,kw. | 451 |
| 27 | ((pragmatic or practical) adj3 trial*).ti,ab,hw,kf,kw. | 5816 |
| 28 | ((quasiexperimental or quasi-experimental) adj3 (study or studies or trial*)).ti,ab,hw,kf,kw. | 8734 |
| 29 | (phase adj3 (III or "3") adj3 (study or studies or trial*)).ti,hw,kf,kw. | 29992 |
| 30 | 1 or 2 or 3 or 4 or 5 or 6 or 7 or 8 or 9 or 10 or 11 or 12 or 13 or 14 or 15 or 16 or 17 or 18 or 19 or 20 or 21 or 22 or 23 or 24 or 25 or 26 or 27 or 28 or 29 | 2257829 |
| 31 | meta-analysis.pt. | 127955 |
| 32 | meta-analysis/ or systematic review/ or meta-analysis as topic/ or "meta analysis (topic)"/ or "systematic review (topic)"/ or exp technology assessment, biomedical/ | 241161 |
| 33 | ((systematic* adj3 (review* or overview*)) or (methodologic* adj3 (review* or overview*))).ti,ab,kf,kw. | 218882 |
| 34 | ((quantitative adj3 (review* or overview* or synthes*)) or (research adj3 (integrati* or overview*))).ti,ab,kf,kw. | 11951 |
| 35 | ((integrative adj3 (review* or overview*)) or (collaborative adj3 (review* or overview*)) or (pool* adj3 analy*)).ti,ab,kf,kw. | 29641 |
| 36 | (data synthes* or data extraction* or data abstraction*).ti,ab,kf,kw. | 30017 |
| 37 | (handsearch* or hand search*).ti,ab,kf,kw. | 9705 |
| 38 | (mantel haenszel or peto or der simonian or dersimonian or fixed effect* or latin square*).ti,ab,kf,kw. | 28434 |
| 39 | (met analy* or metanaly* or technology assessment* or HTA or HTAs or technology overview* or technology appraisal*).ti,ab,kf,kw. | 9958 |
| 40 | (meta regression* or metaregression*).ti,ab,kf,kw. | 10148 |
| 41 | (meta-analy* or metaanaly* or systematic review* or biomedical technology assessment* or bio-medical technology assessment*).mp,hw. | 342442 |
| 42 | (medline or cochrane or pubmed or medlars or embase or cinahl).ti,ab,hw. | 247915 |
| 43 | (cochrane or (health adj2 technology assessment) or evidence report).jw. | 19996 |
| 44 | (comparative adj3 (efficacy or effectiveness)).ti,ab,kf,kw. | 14417 |
| 45 | (outcomes research or relative effectiveness).ti,ab,kf,kw. | 9838 |
| 46 | ((indirect or indirect treatment or mixed-treatment) adj comparison*).ti,ab,kf,kw. | 2299 |
| 47 | 31 or 32 or 33 or 34 or 35 or 36 or 37 or 38 or 39 or 40 or 41 or 42 or 43 or 44 or 45 or 46 | 516309 |
| 48 | Case-Control Studies/ or Control Groups/ or Matched-Pair Analysis/ or ((case* adj5 control*) or (case adj3 comparison*) or control group*).ti,ab,kw. | 822747 |
| 49 | cohort studies/ or longitudinal studies/ or follow-up studies/ or prospective studies/ or retrospective studies/ or cohort.ti,ab. or longitudinal.ti,ab. or prospective.ti,ab. or retrospective.ti,ab. | 2794459 |
| 50 | (autobiography or bibliography or biography or case reports or classical article or letter or patient education handout or dictionary or directory or editorial or historical article or interactive tutorial or interview or introductory journal article or lecture or legal case or legislation or news or newspaper article or personal narrative or portrait or video audio media or webcast).pt. | 4310001 |
| 51 | exp animals/ not humans.sh. | 4799281 |
| 52 | (exp infant/ or exp child/ or adolescent/) not exp adult/ | 1918215 |
| 53 | 50 or 51 or 52 | 10404345 |
| 54 | ((incisional or postoperative or post-operative or ventral) adj3 hernia?).ab,kf,kw,ti. | 8404 |
| 55 | hernia, ventral/ or incisional hernia/ | 7067 |
| 56 | 54 or 55 | 11263 |
| 57 | (Watchful waiting or "watch and wait" or WAW).ab,kf,kw,ti. | 3830 |
| 58 | Conservative.ab,kf,kw,ti. | 110730 |
| 59 | (non-surgical or nonsurgical or non surgical or non-operative or nonoperative or non operative).ab,kf,kw,ti. | 44010 |
| 60 | hernia support.ab,kf,kw,ti. | 0 |
| 61 | hernia truss*.ab,kf,kw,ti. | 2 |
| 62 | Watchful Waiting/ or Conservative Treatment/ | 7680 |
| 63 | 57 or 58 or 59 or 60 or 61 or 62 | 158696 |
| 64 | 56 and 63 | 224 |
| 65 | 64 not 53 | 165 |
| 66 | limit 65 to english language | 143 |
| 67 | 30 and 66 | 15 |
| 68 | 47 and 66 | 12 |
| 69 | 48 and 66 | 5 |
| 70 | 49 and 66 | 93 |

**Embase <1974 to 2021 March 12> (Ovid)**

**Search was conducted on 14^th^ March 2021.**

| # | search string | # of results |
| --- | --- | --- |
| 1 | (Randomized Controlled Trial or Controlled Clinical Trial or Pragmatic Clinical Trial or Equivalence Trial or Clinical Trial, Phase III).pt. | 0 |
| 2 | Randomized Controlled Trial/ | 651778 |
| 3 | exp Randomized Controlled Trials as Topic/ | 198855 |
| 4 | "Randomized Controlled Trial (topic)"/ | 198855 |
| 5 | Controlled Clinical Trial/ | 466838 |
| 6 | exp Controlled Clinical Trials as Topic/ | 206807 |
| 7 | "Controlled Clinical Trial (topic)"/ | 11531 |
| 8 | Randomization/ | 90619 |
| 9 | Random Allocation/ | 86809 |
| 10 | Double-Blind Method/ | 158430 |
| 11 | Double Blind Procedure/ | 182771 |
| 12 | Double-Blind Studies/ | 142095 |
| 13 | Single-Blind Method/ | 40318 |
| 14 | Single Blind Procedure/ | 42335 |
| 15 | Single-Blind Studies/ | 42335 |
| 16 | Placebos/ | 309399 |
| 17 | Placebo/ | 365195 |
| 18 | Control Groups/ | 110539 |
| 19 | Control Group/ | 110539 |
| 20 | (random* or sham or placebo*).ti,ab,hw,kw. | 2172603 |
| 21 | ((singl* or doubl*) adj (blind* or dumm* or mask*)).ti,ab,hw,kw. | 320093 |
| 22 | ((tripl* or trebl*) adj (blind* or dumm* or mask*)).ti,ab,hw,kw. | 1517 |
| 23 | (control* adj3 (study or studies or trial* or group*)).ti,ab,kw. | 1457801 |
| 24 | (Nonrandom* or non random* or non-random* or quasi-random* or quasirandom*).ti,ab,hw,kw. | 58796 |
| 25 | allocated.ti,ab,hw. | 90951 |
| 26 | ((open label or open-label) adj5 (study or studies or trial*)).ti,ab,hw,kw. | 68645 |
| 27 | ((equivalence or superiority or non-inferiority or noninferiority) adj3 (study or studies or trial*)).ti,ab,hw,kw. | 13672 |
| 28 | (pragmatic study or pragmatic studies).ti,ab,hw,kw. | 674 |
| 29 | ((pragmatic or practical) adj3 trial*).ti,ab,hw,kw. | 6189 |
| 30 | ((quasiexperimental or quasi-experimental) adj3 (study or studies or trial*)).ti,ab,hw,kw. | 14063 |
| 31 | (phase adj3 (III or "3") adj3 (study or studies or trial*)).ti,hw,kw. | 98966 |
| 32 | 1 or 2 or 3 or 4 or 5 or 6 or 7 or 8 or 9 or 10 or 11 or 12 or 13 or 14 or 15 or 16 or 17 or 18 or 19 or 20 or 21 or 22 or 23 or 24 or 25 or 26 or 27 or 28 or 29 or 30 or 31 | 3232963 |
| 33 | meta-analysis.pt. | 0 |
| 34 | meta-analysis/ or systematic review/ or meta-analysis as topic/ or "meta analysis (topic)"/ or "systematic review (topic)"/ or exp technology assessment, biomedical/ | 455174 |
| 35 | ((systematic* adj3 (review* or overview*)) or (methodologic* adj3 (review* or overview*))).ti,ab,kw. | 274697 |
| 36 | ((quantitative adj3 (review* or overview* or synthes*)) or (research adj3 (integrati* or overview*))).ti,ab,kw. | 14248 |
| 37 | ((integrative adj3 (review* or overview*)) or (collaborative adj3 (review* or overview*)) or (pool* adj3 analy*)).ti,ab,kw. | 42507 |
| 38 | (data synthes* or data extraction* or data abstraction*).ti,ab,kw. | 37317 |
| 39 | (handsearch* or hand search*).ti,ab,kw. | 11847 |
| 40 | (mantel haenszel or peto or der simonian or dersimonian or fixed effect* or latin square*).ti,ab,kw. | 37755 |
| 41 | (met analy* or metanaly* or technology assessment* or HTA or HTAs or technology overview* or technology appraisal*).ti,ab,kw. | 16298 |
| 42 | (meta regression* or metaregression*).ti,ab,kw. | 12758 |
| 43 | (meta-analy* or metaanaly* or systematic review* or biomedical technology assessment* or bio-medical technology assessment*).mp,hw. | 547838 |
| 44 | (medline or cochrane or pubmed or medlars or embase or cinahl).ti,ab,hw. | 328261 |
| 45 | (cochrane or (health adj2 technology assessment) or evidence report).jx. | 27593 |
| 46 | (comparative adj3 (efficacy or effectiveness)).ti,ab,kw. | 21142 |
| 47 | (outcomes research or relative effectiveness).ti,ab,kw. | 14232 |
| 48 | ((indirect or indirect treatment or mixed-treatment) adj comparison*).ti,ab,kw. | 4384 |
| 49 | 33 or 34 or 35 or 36 or 37 or 38 or 39 or 40 or 41 or 42 or 43 or 44 or 45 or 46 or 47 or 48 | 756660 |
| 50 | exp case control study/ or control group/ or statistical analysis/ or ((case* adj5 control*) or (case adj3 comparison*) or control group*).ti,kw,ab. | 1227352 |
| 51 | cohort analysis/ or longitudinal study/ or follow up/ or prospective study/ or retrospective study/ or cohort.ti,ab. or longitudinal.ti,ab. or prospective.ti,ab. or retrospective.ti,ab. | 4290705 |
| 52 | (conference abstract or "conference review" or editorial or letter or note or short survey).pt. | 7133520 |
| 53 | (exp animal/ or animal experiment/ or nonhuman/) not (exp human/ or human experiment/) | 6666363 |
| 54 | exp juvenile/ not exp adult/ | 2278466 |
| 55 | 52 or 53 or 54 | 14870312 |
| 56 | ((incisional or postoperative or post-operative or ventral) adj3 hernia?).ab,kw,ti. | 12011 |
| 57 | abdominal wall hernia/ or incisional hernia/ | 15742 |
| 58 | 56 or 57 | 18639 |
| 59 | (Watchful waiting or "watch and wait" or WAW).ab,kw,ti. | 6261 |
| 60 | Conservative.ab,kw,ti. | 146095 |
| 61 | (non-surgical or nonsurgical or non surgical or non-operative or nonoperative or non operative).ab,kw,ti. | 57224 |
| 62 | hernia support.ab,kw,ti. | 3 |
| 63 | hernia truss*.ab,kw,ti. | 1 |
| 64 | exp conservative treatment/ | 604187 |
| 65 | 59 or 60 or 61 or 62 or 63 or 64 | 750105 |
| 66 | 58 and 65 | 791 |
| 67 | 66 not 55 | 554 |
| 68 | limit 67 to english language | 508 |
| 69 | 32 and 68 | 59 |
| 70 | 49 and 68 | 30 |
| 71 | 50 and 68 | 14 |
| 72 | 51 and 68 | 289 |

**Cochrane Library**

[**Cochrane Database of Systematic Reviews**](https://www.cochranelibrary.com/)**, Issue 3 of 12, March 2021**

**Cochrane Central Register of Controlled Trials, Issue 3 of 12, March 2021**

**Search was conducted on 18^th^ March 2021.**

| **#** | **search string** | **# of results** |
| --- | --- | --- |
| **1** | ((incisional or postoperative or post-operative or ventral) NEAR/3 hernia?):ti,ab,kw | 1196 |
| **2** | MeSH descriptor: [Hernia, Ventral] this term only | 268 |
| **3** | MeSH descriptor: [Incisional Hernia] this term only | 127 |
| **4** | #1 or #2 or #3 | 1196 |
| **5** | ("Watchful waiting" or "watch and wait" or WAW):ti,ab,kw | 941 |
| **6** | (Conservative):ti,ab,kw | 9816 |
| **7** | (non-surgical or nonsurgical or "non surgical" or non-operative or nonoperative or "non operative"):ti,ab,kw | 5413 |
| **8** | (hernia support):ti,ab,kw | 219 |
| **9** | (hernia NEXT truss*):ti,ab,kw | 0 |
| **10** | MeSH descriptor: [Watchful Waiting] this term only | 315 |
| **11** | MeSH descriptor: [Conservative Treatment] this term only | 145 |
| **12** | #5 OR #6 OR #7 OR #8 OR #9 OR #10 OR #11 | 15499 |
| **13** | #4 AND #12 | 56 |
| **14** | #13 AND Cochrane Database of Systematic Reviews | 3 |
| **15** | #13 AND Cochrane Central Register of Controlled Trials | 53 |

**KQ 4: What are the important outcomes measures in treatment of incisional hernias?**

**MEDLINE(R) ALL <1946 to March 15, 2021> (Ovid)**

**Search was conducted on 16^th^ March 2021.**

| # | search string | # of results |
| --- | --- | --- |
| 1 | (Randomized Controlled Trial or Controlled Clinical Trial or Pragmatic Clinical Trial or Equivalence Trial or Clinical Trial, Phase III).pt. | 618044 |
| 2 | Randomized Controlled Trial/ | 525030 |
| 3 | exp Randomized Controlled Trials as Topic/ | 144810 |
| 4 | Controlled Clinical Trial/ | 94095 |
| 5 | exp Controlled Clinical Trials as Topic/ | 150296 |
| 6 | Randomization/ | 104842 |
| 7 | Random Allocation/ | 104842 |
| 8 | Double-Blind Method/ | 162935 |
| 9 | Double Blind Procedure/ | 0 |
| 10 | Double-Blind Studies/ | 162935 |
| 11 | Single-Blind Method/ | 29867 |
| 12 | Single Blind Procedure/ | 0 |
| 13 | Single-Blind Studies/ | 29867 |
| 14 | Placebos/ | 35377 |
| 15 | Placebo/ | 0 |
| 16 | Control Groups/ | 1725 |
| 17 | Control Group/ | 1725 |
| 18 | (random* or sham or placebo*).ti,ab,hw,kf,kw. | 1572351 |
| 19 | ((singl* or doubl*) adj (blind* or dumm* or mask*)).ti,ab,hw,kf,kw. | 244459 |
| 20 | ((tripl* or trebl*) adj (blind* or dumm* or mask*)).ti,ab,hw,kf,kw. | 1144 |
| 21 | (control* adj3 (study or studies or trial* or group*)).ti,ab,kf,kw. | 1039014 |
| 22 | (Nonrandom* or non random* or non-random* or quasi-random* or quasirandom*).ti,ab,hw,kf,kw. | 46532 |
| 23 | allocated.ti,ab,hw. | 70017 |
| 24 | ((open label or open-label) adj5 (study or studies or trial*)).ti,ab,hw,kf,kw. | 36955 |
| 25 | ((equivalence or superiority or non-inferiority or noninferiority) adj3 (study or studies or trial*)).ti,ab,hw,kf,kw. | 9227 |
| 26 | (pragmatic study or pragmatic studies).ti,ab,hw,kf,kw. | 451 |
| 27 | ((pragmatic or practical) adj3 trial*).ti,ab,hw,kf,kw. | 5818 |
| 28 | ((quasiexperimental or quasi-experimental) adj3 (study or studies or trial*)).ti,ab,hw,kf,kw. | 8739 |
| 29 | (phase adj3 (III or "3") adj3 (study or studies or trial*)).ti,hw,kf,kw. | 30001 |
| 30 | 1 or 2 or 3 or 4 or 5 or 6 or 7 or 8 or 9 or 10 or 11 or 12 or 13 or 14 or 15 or 16 or 17 or 18 or 19 or 20 or 21 or 22 or 23 or 24 or 25 or 26 or 27 or 28 or 29 | 2258738 |
| 31 | meta-analysis.pt. | 128007 |
| 32 | meta-analysis/ or systematic review/ or meta-analysis as topic/ or "meta analysis (topic)"/ or "systematic review (topic)"/ or exp technology assessment, biomedical/ | 241293 |
| 33 | ((systematic* adj3 (review* or overview*)) or (methodologic* adj3 (review* or overview*))).ti,ab,kf,kw. | 219099 |
| 34 | ((quantitative adj3 (review* or overview* or synthes*)) or (research adj3 (integrati* or overview*))).ti,ab,kf,kw. | 11957 |
| 35 | ((integrative adj3 (review* or overview*)) or (collaborative adj3 (review* or overview*)) or (pool* adj3 analy*)).ti,ab,kf,kw. | 29667 |
| 36 | (data synthes* or data extraction* or data abstraction*).ti,ab,kf,kw. | 30033 |
| 37 | (handsearch* or hand search*).ti,ab,kf,kw. | 9705 |
| 38 | (mantel haenszel or peto or der simonian or dersimonian or fixed effect* or latin square*).ti,ab,kf,kw. | 28453 |
| 39 | (met analy* or metanaly* or technology assessment* or HTA or HTAs or technology overview* or technology appraisal*).ti,ab,kf,kw. | 9963 |
| 40 | (meta regression* or metaregression*).ti,ab,kf,kw. | 10163 |
| 41 | (meta-analy* or metaanaly* or systematic review* or biomedical technology assessment* or bio-medical technology assessment*).mp,hw. | 342728 |
| 42 | (medline or cochrane or pubmed or medlars or embase or cinahl).ti,ab,hw. | 248140 |
| 43 | (cochrane or (health adj2 technology assessment) or evidence report).jw. | 19999 |
| 44 | (comparative adj3 (efficacy or effectiveness)).ti,ab,kf,kw. | 14425 |
| 45 | (outcomes research or relative effectiveness).ti,ab,kf,kw. | 9841 |
| 46 | ((indirect or indirect treatment or mixed-treatment) adj comparison*).ti,ab,kf,kw. | 2302 |
| 47 | 31 or 32 or 33 or 34 or 35 or 36 or 37 or 38 or 39 or 40 or 41 or 42 or 43 or 44 or 45 or 46 | 516705 |
| 48 | Case-Control Studies/ or Control Groups/ or Matched-Pair Analysis/ or ((case* adj5 control*) or (case adj3 comparison*) or control group*).ti,ab,kw. | 823084 |
| 49 | cohort studies/ or longitudinal studies/ or follow-up studies/ or prospective studies/ or retrospective studies/ or cohort.ti,ab. or longitudinal.ti,ab. or prospective.ti,ab. or retrospective.ti,ab. | 2795784 |
| 50 | (autobiography or bibliography or biography or case reports or classical article or letter or patient education handout or dictionary or directory or editorial or historical article or interactive tutorial or interview or introductory journal article or lecture or legal case or legislation or news or newspaper article or personal narrative or portrait or video audio media or webcast).pt. | 4310851 |
| 51 | exp animals/ not humans.sh. | 4799766 |
| 52 | (exp infant/ or exp child/ or adolescent/) not exp adult/ | 1918444 |
| 53 | 50 or 51 or 52 | 10405865 |
| 54 | ((incisional or postoperative or post-operative or ventral) adj3 hernia?).ab,kf,kw,ti. | 8407 |
| 55 | hernia, ventral/ or incisional hernia/ | 7067 |
| 56 | 54 or 55 | 11266 |
| 57 | (Outcome? adj (assessment? or research* or study or studies or measure*)).ab,kf,kw,ti. | 277658 |
| 58 | exp "Outcome Assessment (Health Care)"/ | 1177268 |
| 59 | (Endpoint? or end-point?).ab,kf,kw,ti. | 178047 |
| 60 | Effect? Measure*.ab,kf,kw,ti. | 3361 |
| 61 | (Health status or health level? or "level of health" or clinical state or health state).ab,kf,kw,ti. | 75285 |
| 62 | exp health status/ | 356235 |
| 63 | ("quality of life" or Life quality or QoL or HRQL or HRQoL).ab,kf,kw,ti. | 309027 |
| 64 | (Patient-related outcome? or patient related outcome? or PROM or patient-reported outcome? or patient reported outcome?).ab,kf,kw,ti. | 26056 |
| 65 | ("Activit* of Daily Living" or Daily Living activit* or "Limitation of Activit*" or daily life activit* or ADL).ab,kf,kw,ti. | 36579 |
| 66 | exp "Activities of Daily Living"/ | 105839 |
| 67 | Acute hernia accident?.ab,kf,kw,ti. | 0 |
| 68 | 57 or 58 or 59 or 60 or 61 or 62 or 63 or 64 or 65 or 66 or 67 | 2031862 |
| 69 | 56 and 68 | 2682 |
| 70 | 69 not 53 | 2315 |
| 71 | limit 70 to english language | 2067 |
| 72 | 30 and 71 | 440 |
| 73 | 47 and 71 | 165 |
| 74 | 48 and 71 | 103 |
| 75 | 49 and 71 | 1524 |

**Embase <1974 to 2021 March 12> (Ovid)**

**Search was conducted on 14^th^ March 2021.**

| # | search string | # of results |
| --- | --- | --- |
| 1 | (Randomized Controlled Trial or Controlled Clinical Trial or Pragmatic Clinical Trial or Equivalence Trial or Clinical Trial, Phase III).pt. | 0 |
| 2 | Randomized Controlled Trial/ | 651778 |
| 3 | exp Randomized Controlled Trials as Topic/ | 198855 |
| 4 | "Randomized Controlled Trial (topic)"/ | 198855 |
| 5 | Controlled Clinical Trial/ | 466838 |
| 6 | exp Controlled Clinical Trials as Topic/ | 206807 |
| 7 | "Controlled Clinical Trial (topic)"/ | 11531 |
| 8 | Randomization/ | 90619 |
| 9 | Random Allocation/ | 86809 |
| 10 | Double-Blind Method/ | 158430 |
| 11 | Double Blind Procedure/ | 182771 |
| 12 | Double-Blind Studies/ | 142095 |
| 13 | Single-Blind Method/ | 40318 |
| 14 | Single Blind Procedure/ | 42335 |
| 15 | Single-Blind Studies/ | 42335 |
| 16 | Placebos/ | 309399 |
| 17 | Placebo/ | 365195 |
| 18 | Control Groups/ | 110539 |
| 19 | Control Group/ | 110539 |
| 20 | (random* or sham or placebo*).ti,ab,hw,kw. | 2172603 |
| 21 | ((singl* or doubl*) adj (blind* or dumm* or mask*)).ti,ab,hw,kw. | 320093 |
| 22 | ((tripl* or trebl*) adj (blind* or dumm* or mask*)).ti,ab,hw,kw. | 1517 |
| 23 | (control* adj3 (study or studies or trial* or group*)).ti,ab,kw. | 1457801 |
| 24 | (Nonrandom* or non random* or non-random* or quasi-random* or quasirandom*).ti,ab,hw,kw. | 58796 |
| 25 | allocated.ti,ab,hw. | 90951 |
| 26 | ((open label or open-label) adj5 (study or studies or trial*)).ti,ab,hw,kw. | 68645 |
| 27 | ((equivalence or superiority or non-inferiority or noninferiority) adj3 (study or studies or trial*)).ti,ab,hw,kw. | 13672 |
| 28 | (pragmatic study or pragmatic studies).ti,ab,hw,kw. | 674 |
| 29 | ((pragmatic or practical) adj3 trial*).ti,ab,hw,kw. | 6189 |
| 30 | ((quasiexperimental or quasi-experimental) adj3 (study or studies or trial*)).ti,ab,hw,kw. | 14063 |
| 31 | (phase adj3 (III or "3") adj3 (study or studies or trial*)).ti,hw,kw. | 98966 |
| 32 | 1 or 2 or 3 or 4 or 5 or 6 or 7 or 8 or 9 or 10 or 11 or 12 or 13 or 14 or 15 or 16 or 17 or 18 or 19 or 20 or 21 or 22 or 23 or 24 or 25 or 26 or 27 or 28 or 29 or 30 or 31 | 3232963 |
| 33 | meta-analysis.pt. | 0 |
| 34 | meta-analysis/ or systematic review/ or meta-analysis as topic/ or "meta analysis (topic)"/ or "systematic review (topic)"/ or exp technology assessment, biomedical/ | 455174 |
| 35 | ((systematic* adj3 (review* or overview*)) or (methodologic* adj3 (review* or overview*))).ti,ab,kw. | 274697 |
| 36 | ((quantitative adj3 (review* or overview* or synthes*)) or (research adj3 (integrati* or overview*))).ti,ab,kw. | 14248 |
| 37 | ((integrative adj3 (review* or overview*)) or (collaborative adj3 (review* or overview*)) or (pool* adj3 analy*)).ti,ab,kw. | 42507 |
| 38 | (data synthes* or data extraction* or data abstraction*).ti,ab,kw. | 37317 |
| 39 | (handsearch* or hand search*).ti,ab,kw. | 11847 |
| 40 | (mantel haenszel or peto or der simonian or dersimonian or fixed effect* or latin square*).ti,ab,kw. | 37755 |
| 41 | (met analy* or metanaly* or technology assessment* or HTA or HTAs or technology overview* or technology appraisal*).ti,ab,kw. | 16298 |
| 42 | (meta regression* or metaregression*).ti,ab,kw. | 12758 |
| 43 | (meta-analy* or metaanaly* or systematic review* or biomedical technology assessment* or bio-medical technology assessment*).mp,hw. | 547838 |
| 44 | (medline or cochrane or pubmed or medlars or embase or cinahl).ti,ab,hw. | 328261 |
| 45 | (cochrane or (health adj2 technology assessment) or evidence report).jx. | 27593 |
| 46 | (comparative adj3 (efficacy or effectiveness)).ti,ab,kw. | 21142 |
| 47 | (outcomes research or relative effectiveness).ti,ab,kw. | 14232 |
| 48 | ((indirect or indirect treatment or mixed-treatment) adj comparison*).ti,ab,kw. | 4384 |
| 49 | 33 or 34 or 35 or 36 or 37 or 38 or 39 or 40 or 41 or 42 or 43 or 44 or 45 or 46 or 47 or 48 | 756660 |
| 50 | exp case control study/ or control group/ or statistical analysis/ or ((case* adj5 control*) or (case adj3 comparison*) or control group*).ti,kw,ab. | 1227352 |
| 51 | cohort analysis/ or longitudinal study/ or follow up/ or prospective study/ or retrospective study/ or cohort.ti,ab. or longitudinal.ti,ab. or prospective.ti,ab. or retrospective.ti,ab. | 4290705 |
| 52 | (conference abstract or "conference review" or editorial or letter or note or short survey).pt. | 7133520 |
| 53 | (exp animal/ or animal experiment/ or nonhuman/) not (exp human/ or human experiment/) | 6666363 |
| 54 | exp juvenile/ not exp adult/ | 2278466 |
| 55 | 52 or 53 or 54 | 14870312 |
| 56 | ((incisional or postoperative or post-operative or ventral) adj3 hernia?).ab,kw,ti. | 12011 |
| 57 | abdominal wall hernia/ or incisional hernia/ | 15742 |
| 58 | 56 or 57 | 18639 |
| 59 | (Outcome? adj (assessment? or research* or study or studies or measure*)).ab,kw,ti. | 369555 |
| 60 | (Endpoint? or end-point?).ab,kw,ti. | 316568 |
| 61 | Effect? Measure*.ab,kw,ti. | 3928 |
| 62 | (Health status or health level or "level of health" or clinical state or health state).ab,kw,ti. | 100097 |
| 63 | ("quality of life" or Life quality or QoL or HRQL or HRQoL).ab,kw,ti. | 502101 |
| 64 | (Patient-related outcome? or patient related outcome? or PROM or patient-reported outcome? or patient reported outcome?).ab,kw,ti. | 44182 |
| 65 | ("Activit* of Daily Living" or Daily Living activit* or "Limitation of Activit*" or daily life activit* or ADL).ab,kw,ti. | 55485 |
| 66 | Acute hernia accident?.ab,kw,ti. | 0 |
| 67 | outcome assessment/ | 581677 |
| 68 | exp health status/ | 253253 |
| 69 | "quality of life"/ | 501051 |
| 70 | daily life activity/ | 94681 |
| 71 | 59 or 60 or 61 or 62 or 63 or 64 or 65 or 66 or 67 or 68 or 69 or 70 | 1985897 |
| 72 | 58 and 71 | 2836 |
| 73 | 72 not 55 | 2055 |
| 74 | limit 73 to english language | 1983 |
| 75 | 32 and 74 | 542 |
| 76 | 49 and 74 | 218 |
| 77 | 50 and 74 | 102 |
| 78 | 51 and 74 | 1374 |

**Cochrane Library**

[**Cochrane Database of Systematic Reviews**](https://www.cochranelibrary.com/)**, Issue 3 of 12, March 2021**

**Cochrane Central Register of Controlled Trials, Issue 3 of 12, March 2021**

**Search was conducted on 18^th^ March 2021.**

| **#** | **search string** | **# of results** |
| --- | --- | --- |
| **1** | ((incisional or postoperative or post-operative or ventral) NEAR/3 hernia?):ti,ab,kw | 1196 |
| **2** | MeSH descriptor: [Hernia, Ventral] this term only | 268 |
| **3** | MeSH descriptor: [Incisional Hernia] this term only | 127 |
| **4** | #1 or #2 or #3 | 1196 |
| **5** | (Outcome? NEXT (assessment? or research* or study or studies or measure*)):ti,ab,kw | 135517 |
| **6** | MeSH descriptor: [Outcome Assessment, Health Care] explode all trees | 148864 |
| **7** | (Endpoint? or end-point?):ti,ab,kw | 132938 |
| **8** | (Effect? NEXT Measure*):ti,ab,kw | 1120 |
| **9** | ("Health status" or (health NEXT level?) or "level of health" or "clinical state" or "health state"):ti,ab,kw | 15320 |
| **10** | MeSH descriptor: [Health Status] explode all trees | 30363 |
| **11** | ("quality of life" or "Life quality" or QoL or HRQL or HRQoL):ti,ab,kw | 118235 |
| **12** | ((Patient-related NEXT outcome?) or (patient NEXT related NEXT outcome?) or PROM or (patient-reported NEXT outcome?) or (patient NEXT reported NEXT outcome?)):ti,ab,kw | 9852 |
| **13** | ("Activity of Daily Living" or "Activities of daily living" or (Daily NEXT Living NEXT activit*) or "Limitation of Activity" or "Limitation of activities" or (daily NEXT life NEXT activit*) or ADL):ti,ab,kw | 15195 |
| **14** | MeSH descriptor: [Activities of Daily Living] explode all trees | 9486 |
| **15** | (Acute NEXT hernia NEXT accident?):ti,ab,kw | 0 |
| **16** | #5 OR #6 OR #7 OR #8 OR #9 OR #10 OR #11 OR #12 OR #13 OR #14 OR #15 | 444571 |
| **17** | #4 AND #16 | 513 |
| **18** | #17 AND Cochrane Database of Systematic Reviews | 5 |
| **19** | #17 AND Cochrane Central Register of Controlled Trials | 508 |

**KQ 5: What are the important modifiable risk factors that should be optimised preoperatively? What is the effect of pre-optimisation?**

**MEDLINE(R) ALL <1946 to March 12, 2021> (Ovid)**

**Search was conducted on 15^th^ March 2021.**

| # | search string | # of results |
| --- | --- | --- |
| 1 | (Randomized Controlled Trial or Controlled Clinical Trial or Pragmatic Clinical Trial or Equivalence Trial or Clinical Trial, Phase III).pt. | 617974 |
| 2 | Randomized Controlled Trial/ | 524960 |
| 3 | exp Randomized Controlled Trials as Topic/ | 144784 |
| 4 | Controlled Clinical Trial/ | 94095 |
| 5 | exp Controlled Clinical Trials as Topic/ | 150269 |
| 6 | Randomization/ | 104832 |
| 7 | Random Allocation/ | 104832 |
| 8 | Double-Blind Method/ | 162912 |
| 9 | Double Blind Procedure/ | 0 |
| 10 | Double-Blind Studies/ | 162912 |
| 11 | Single-Blind Method/ | 29863 |
| 12 | Single Blind Procedure/ | 0 |
| 13 | Single-Blind Studies/ | 29863 |
| 14 | Placebos/ | 35374 |
| 15 | Placebo/ | 0 |
| 16 | Control Groups/ | 1725 |
| 17 | Control Group/ | 1725 |
| 18 | (random* or sham or placebo*).ti,ab,hw,kf,kw. | 1571724 |
| 19 | ((singl* or doubl*) adj (blind* or dumm* or mask*)).ti,ab,hw,kf,kw. | 244397 |
| 20 | ((tripl* or trebl*) adj (blind* or dumm* or mask*)).ti,ab,hw,kf,kw. | 1142 |
| 21 | (control* adj3 (study or studies or trial* or group*)).ti,ab,kf,kw. | 1038525 |
| 22 | (Nonrandom* or non random* or non-random* or quasi-random* or quasirandom*).ti,ab,hw,kf,kw. | 46508 |
| 23 | allocated.ti,ab,hw. | 69975 |
| 24 | ((open label or open-label) adj5 (study or studies or trial*)).ti,ab,hw,kf,kw. | 36939 |
| 25 | ((equivalence or superiority or non-inferiority or noninferiority) adj3 (study or studies or trial*)).ti,ab,hw,kf,kw. | 9217 |
| 26 | (pragmatic study or pragmatic studies).ti,ab,hw,kf,kw. | 451 |
| 27 | ((pragmatic or practical) adj3 trial*).ti,ab,hw,kf,kw. | 5816 |
| 28 | ((quasiexperimental or quasi-experimental) adj3 (study or studies or trial*)).ti,ab,hw,kf,kw. | 8734 |
| 29 | (phase adj3 (III or "3") adj3 (study or studies or trial*)).ti,hw,kf,kw. | 29992 |
| 30 | 1 or 2 or 3 or 4 or 5 or 6 or 7 or 8 or 9 or 10 or 11 or 12 or 13 or 14 or 15 or 16 or 17 or 18 or 19 or 20 or 21 or 22 or 23 or 24 or 25 or 26 or 27 or 28 or 29 | 2257829 |
| 31 | meta-analysis.pt. | 127955 |
| 32 | meta-analysis/ or systematic review/ or meta-analysis as topic/ or "meta analysis (topic)"/ or "systematic review (topic)"/ or exp technology assessment, biomedical/ | 241161 |
| 33 | ((systematic* adj3 (review* or overview*)) or (methodologic* adj3 (review* or overview*))).ti,ab,kf,kw. | 218882 |
| 34 | ((quantitative adj3 (review* or overview* or synthes*)) or (research adj3 (integrati* or overview*))).ti,ab,kf,kw. | 11951 |
| 35 | ((integrative adj3 (review* or overview*)) or (collaborative adj3 (review* or overview*)) or (pool* adj3 analy*)).ti,ab,kf,kw. | 29641 |
| 36 | (data synthes* or data extraction* or data abstraction*).ti,ab,kf,kw. | 30017 |
| 37 | (handsearch* or hand search*).ti,ab,kf,kw. | 9705 |
| 38 | (mantel haenszel or peto or der simonian or dersimonian or fixed effect* or latin square*).ti,ab,kf,kw. | 28434 |
| 39 | (met analy* or metanaly* or technology assessment* or HTA or HTAs or technology overview* or technology appraisal*).ti,ab,kf,kw. | 9958 |
| 40 | (meta regression* or metaregression*).ti,ab,kf,kw. | 10148 |
| 41 | (meta-analy* or metaanaly* or systematic review* or biomedical technology assessment* or bio-medical technology assessment*).mp,hw. | 342442 |
| 42 | (medline or cochrane or pubmed or medlars or embase or cinahl).ti,ab,hw. | 247915 |
| 43 | (cochrane or (health adj2 technology assessment) or evidence report).jw. | 19996 |
| 44 | (comparative adj3 (efficacy or effectiveness)).ti,ab,kf,kw. | 14417 |
| 45 | (outcomes research or relative effectiveness).ti,ab,kf,kw. | 9838 |
| 46 | ((indirect or indirect treatment or mixed-treatment) adj comparison*).ti,ab,kf,kw. | 2299 |
| 47 | 31 or 32 or 33 or 34 or 35 or 36 or 37 or 38 or 39 or 40 or 41 or 42 or 43 or 44 or 45 or 46 | 516309 |
| 48 | Case-Control Studies/ or Control Groups/ or Matched-Pair Analysis/ or ((case* adj5 control*) or (case adj3 comparison*) or control group*).ti,ab,kw. | 822747 |
| 49 | cohort studies/ or longitudinal studies/ or follow-up studies/ or prospective studies/ or retrospective studies/ or cohort.ti,ab. or longitudinal.ti,ab. or prospective.ti,ab. or retrospective.ti,ab. | 2794459 |
| 50 | (autobiography or bibliography or biography or case reports or classical article or letter or patient education handout or dictionary or directory or editorial or historical article or interactive tutorial or interview or introductory journal article or lecture or legal case or legislation or news or newspaper article or personal narrative or portrait or video audio media or webcast).pt. | 4310001 |
| 51 | exp animals/ not humans.sh. | 4799281 |
| 52 | (exp infant/ or exp child/ or adolescent/) not exp adult/ | 1918215 |
| 53 | 50 or 51 or 52 | 10404345 |
| 54 | ((incisional or postoperative or post-operative or ventral) adj3 hernia?).ab,kf,kw,ti. | 8404 |
| 55 | hernia, ventral/ or incisional hernia/ | 7067 |
| 56 | 54 or 55 | 11263 |
| 57 | (prehabilitat* or pre-habilitat* or "pre habilitat*" or prehab? or pre-hab? or "pre hab?" or Optimis* or Optimiz* or pre-optimis* or pre-optimiz* or preoptimis* or preoptimiz*).ab,kf,kw,ti. | 456814 |
| 58 | ((presurgery or pre-surgery or "pre surgery" or pre-surgical or presurgical or "pre surgical" or Preoperative* or Pre-operative* or "Pre operative*") adj3 (exercise? or conditioning? or rehabilitation? or habilitation?)).ab,kf,kw,ti. | 949 |
| 59 | Preoperative Exercise/ | 38 |
| 60 | 57 or 58 or 59 | 457599 |
| 61 | 56 and 60 | 146 |
| 62 | 61 not 53 | 132 |
| 63 | limit 62 to english language | 121 |
| 64 | 30 and 63 | 23 |
| 65 | 47 and 63 | 11 |
| 66 | 48 and 63 | 2 |
| 67 | 49 and 63 | 56 |

**Embase <1974 to 2021 March 12> (Ovid)**

**Search was conducted on 14^th^ March 2021.**

| # | search string | # of results |
| --- | --- | --- |
| 1 | (Randomized Controlled Trial or Controlled Clinical Trial or Pragmatic Clinical Trial or Equivalence Trial or Clinical Trial, Phase III).pt. | 0 |
| 2 | Randomized Controlled Trial/ | 651778 |
| 3 | exp Randomized Controlled Trials as Topic/ | 198855 |
| 4 | "Randomized Controlled Trial (topic)"/ | 198855 |
| 5 | Controlled Clinical Trial/ | 466838 |
| 6 | exp Controlled Clinical Trials as Topic/ | 206807 |
| 7 | "Controlled Clinical Trial (topic)"/ | 11531 |
| 8 | Randomization/ | 90619 |
| 9 | Random Allocation/ | 86809 |
| 10 | Double-Blind Method/ | 158430 |
| 11 | Double Blind Procedure/ | 182771 |
| 12 | Double-Blind Studies/ | 142095 |
| 13 | Single-Blind Method/ | 40318 |
| 14 | Single Blind Procedure/ | 42335 |
| 15 | Single-Blind Studies/ | 42335 |
| 16 | Placebos/ | 309399 |
| 17 | Placebo/ | 365195 |
| 18 | Control Groups/ | 110539 |
| 19 | Control Group/ | 110539 |
| 20 | (random* or sham or placebo*).ti,ab,hw,kw. | 2172603 |
| 21 | ((singl* or doubl*) adj (blind* or dumm* or mask*)).ti,ab,hw,kw. | 320093 |
| 22 | ((tripl* or trebl*) adj (blind* or dumm* or mask*)).ti,ab,hw,kw. | 1517 |
| 23 | (control* adj3 (study or studies or trial* or group*)).ti,ab,kw. | 1457801 |
| 24 | (Nonrandom* or non random* or non-random* or quasi-random* or quasirandom*).ti,ab,hw,kw. | 58796 |
| 25 | allocated.ti,ab,hw. | 90951 |
| 26 | ((open label or open-label) adj5 (study or studies or trial*)).ti,ab,hw,kw. | 68645 |
| 27 | ((equivalence or superiority or non-inferiority or noninferiority) adj3 (study or studies or trial*)).ti,ab,hw,kw. | 13672 |
| 28 | (pragmatic study or pragmatic studies).ti,ab,hw,kw. | 674 |
| 29 | ((pragmatic or practical) adj3 trial*).ti,ab,hw,kw. | 6189 |
| 30 | ((quasiexperimental or quasi-experimental) adj3 (study or studies or trial*)).ti,ab,hw,kw. | 14063 |
| 31 | (phase adj3 (III or "3") adj3 (study or studies or trial*)).ti,hw,kw. | 98966 |
| 32 | 1 or 2 or 3 or 4 or 5 or 6 or 7 or 8 or 9 or 10 or 11 or 12 or 13 or 14 or 15 or 16 or 17 or 18 or 19 or 20 or 21 or 22 or 23 or 24 or 25 or 26 or 27 or 28 or 29 or 30 or 31 | 3232963 |
| 33 | meta-analysis.pt. | 0 |
| 34 | meta-analysis/ or systematic review/ or meta-analysis as topic/ or "meta analysis (topic)"/ or "systematic review (topic)"/ or exp technology assessment, biomedical/ | 455174 |
| 35 | ((systematic* adj3 (review* or overview*)) or (methodologic* adj3 (review* or overview*))).ti,ab,kw. | 274697 |
| 36 | ((quantitative adj3 (review* or overview* or synthes*)) or (research adj3 (integrati* or overview*))).ti,ab,kw. | 14248 |
| 37 | ((integrative adj3 (review* or overview*)) or (collaborative adj3 (review* or overview*)) or (pool* adj3 analy*)).ti,ab,kw. | 42507 |
| 38 | (data synthes* or data extraction* or data abstraction*).ti,ab,kw. | 37317 |
| 39 | (handsearch* or hand search*).ti,ab,kw. | 11847 |
| 40 | (mantel haenszel or peto or der simonian or dersimonian or fixed effect* or latin square*).ti,ab,kw. | 37755 |
| 41 | (met analy* or metanaly* or technology assessment* or HTA or HTAs or technology overview* or technology appraisal*).ti,ab,kw. | 16298 |
| 42 | (meta regression* or metaregression*).ti,ab,kw. | 12758 |
| 43 | (meta-analy* or metaanaly* or systematic review* or biomedical technology assessment* or bio-medical technology assessment*).mp,hw. | 547838 |
| 44 | (medline or cochrane or pubmed or medlars or embase or cinahl).ti,ab,hw. | 328261 |
| 45 | (cochrane or (health adj2 technology assessment) or evidence report).jx. | 27593 |
| 46 | (comparative adj3 (efficacy or effectiveness)).ti,ab,kw. | 21142 |
| 47 | (outcomes research or relative effectiveness).ti,ab,kw. | 14232 |
| 48 | ((indirect or indirect treatment or mixed-treatment) adj comparison*).ti,ab,kw. | 4384 |
| 49 | 33 or 34 or 35 or 36 or 37 or 38 or 39 or 40 or 41 or 42 or 43 or 44 or 45 or 46 or 47 or 48 | 756660 |
| 50 | exp case control study/ or control group/ or statistical analysis/ or ((case* adj5 control*) or (case adj3 comparison*) or control group*).ti,kw,ab. | 1227352 |
| 51 | cohort analysis/ or longitudinal study/ or follow up/ or prospective study/ or retrospective study/ or cohort.ti,ab. or longitudinal.ti,ab. or prospective.ti,ab. or retrospective.ti,ab. | 4290705 |
| 52 | (conference abstract or "conference review" or editorial or letter or note or short survey).pt. | 7133520 |
| 53 | (exp animal/ or animal experiment/ or nonhuman/) not (exp human/ or human experiment/) | 6666363 |
| 54 | exp juvenile/ not exp adult/ | 2278466 |
| 55 | 52 or 53 or 54 | 14870312 |
| 56 | ((incisional or postoperative or post-operative or ventral) adj3 hernia?).ab,kw,ti. | 12011 |
| 57 | abdominal wall hernia/ or incisional hernia/ | 15742 |
| 58 | 56 or 57 | 18639 |
| 59 | (prehabilitat* or pre-habilitat* or "pre habilitat*" or prehab? or pre-hab? or "pre hab?" or Optimis* or Optimiz* or pre-optimis* or pre-optimiz* or preoptimis* or preoptimiz*).ab,kw,ti. | 598768 |
| 60 | ((presurgery or pre-surgery or "pre surgery" or pre-surgical or presurgical or "pre surgical" or Preoperative* or Pre-operative* or "Pre operative*") adj3 (exercise? or conditioning? or rehabilitation? or habilitation?)).ab,kw,ti. | 1431 |
| 61 | 59 or 60 | 599941 |
| 62 | 58 and 61 | 298 |
| 63 | 62 not 55 | 184 |
| 64 | limit 63 to english language | 169 |
| 65 | 32 and 64 | 30 |
| 66 | 49 and 64 | 13 |
| 67 | 50 and 64 | 4 |
| 68 | 51 and 64 | 90 |

**Cochrane Library**

**[Cochrane Database of Systematic Reviews](https://www.cochranelibrary.com/), Issue 3 of 12, March 2021**

**Cochrane Central Register of Controlled Trials, Issue 3 of 12, March 2021**

**Search was conducted on 18^th^ March 2021.**

| **#** | **search string** | **# of results** |
| --- | --- | --- |
| **1** | ((incisional or postoperative or post-operative or ventral) NEAR/3 hernia?):ti,ab,kw | 1196 |
| **2** | MeSH descriptor: [Hernia, Ventral] this term only | 268 |
| **3** | MeSH descriptor: [Incisional Hernia] this term only | 127 |
| **4** | #1 or #2 or #3 | 1196 |
| **5** | (prehabilitat* or pre-habilitat* or (pre NEXT habilitat*) or prehab? or pre-hab? or (pre NEXT hab?) or Optimis* or Optimiz* or pre-optimis* or pre-optimiz* or preoptimis* or preoptimiz*):ti,ab,kw | 23017 |
| **6** | ((presurgery or pre-surgery or "pre surgery" or pre-surgical or presurgical or "pre surgical" or Preoperative* or Pre-operative* or (Pre NEXT operative*)) NEAR/3 (exercise? or conditioning? or rehabilitation? or habilitation?)):ti,ab,kw | 400 |
| **7** | MeSH descriptor: [Preoperative Exercise] this term only | 5 |
| **8** | #5 OR #6 OR #7 | 23311 |
| **9** | #4 and #8 | 29 |
| **10** | #9 AND Cochrane Database of Systematic Reviews | 0 |
| **11** | #9 AND Cochrane Central Register of Controlled Trials | 29 |

**KQ 6: What is the difference in outcome for mesh versus suture repair in incisional hernia repair?**

**MEDLINE(R) ALL <1946 to March 15, 2021> (Ovid)**

**Search was conducted on 16^th^ March 2021.**

| # | search string | # of results |
| --- | --- | --- |
| 1 | (Randomized Controlled Trial or Controlled Clinical Trial or Pragmatic Clinical Trial or Equivalence Trial or Clinical Trial, Phase III).pt. | 618044 |
| 2 | Randomized Controlled Trial/ | 525030 |
| 3 | exp Randomized Controlled Trials as Topic/ | 144810 |
| 4 | Controlled Clinical Trial/ | 94095 |
| 5 | exp Controlled Clinical Trials as Topic/ | 150296 |
| 6 | Randomization/ | 104842 |
| 7 | Random Allocation/ | 104842 |
| 8 | Double-Blind Method/ | 162935 |
| 9 | Double Blind Procedure/ | 0 |
| 10 | Double-Blind Studies/ | 162935 |
| 11 | Single-Blind Method/ | 29867 |
| 12 | Single Blind Procedure/ | 0 |
| 13 | Single-Blind Studies/ | 29867 |
| 14 | Placebos/ | 35377 |
| 15 | Placebo/ | 0 |
| 16 | Control Groups/ | 1725 |
| 17 | Control Group/ | 1725 |
| 18 | (random* or sham or placebo*).ti,ab,hw,kf,kw. | 1572351 |
| 19 | ((singl* or doubl*) adj (blind* or dumm* or mask*)).ti,ab,hw,kf,kw. | 244459 |
| 20 | ((tripl* or trebl*) adj (blind* or dumm* or mask*)).ti,ab,hw,kf,kw. | 1144 |
| 21 | (control* adj3 (study or studies or trial* or group*)).ti,ab,kf,kw. | 1039014 |
| 22 | (Nonrandom* or non random* or non-random* or quasi-random* or quasirandom*).ti,ab,hw,kf,kw. | 46532 |
| 23 | allocated.ti,ab,hw. | 70017 |
| 24 | ((open label or open-label) adj5 (study or studies or trial*)).ti,ab,hw,kf,kw. | 36955 |
| 25 | ((equivalence or superiority or non-inferiority or noninferiority) adj3 (study or studies or trial*)).ti,ab,hw,kf,kw. | 9227 |
| 26 | (pragmatic study or pragmatic studies).ti,ab,hw,kf,kw. | 451 |
| 27 | ((pragmatic or practical) adj3 trial*).ti,ab,hw,kf,kw. | 5818 |
| 28 | ((quasiexperimental or quasi-experimental) adj3 (study or studies or trial*)).ti,ab,hw,kf,kw. | 8739 |
| 29 | (phase adj3 (III or "3") adj3 (study or studies or trial*)).ti,hw,kf,kw. | 30001 |
| 30 | 1 or 2 or 3 or 4 or 5 or 6 or 7 or 8 or 9 or 10 or 11 or 12 or 13 or 14 or 15 or 16 or 17 or 18 or 19 or 20 or 21 or 22 or 23 or 24 or 25 or 26 or 27 or 28 or 29 | 2258738 |
| 31 | meta-analysis.pt. | 128007 |
| 32 | meta-analysis/ or systematic review/ or meta-analysis as topic/ or "meta analysis (topic)"/ or "systematic review (topic)"/ or exp technology assessment, biomedical/ | 241293 |
| 33 | ((systematic* adj3 (review* or overview*)) or (methodologic* adj3 (review* or overview*))).ti,ab,kf,kw. | 219099 |
| 34 | ((quantitative adj3 (review* or overview* or synthes*)) or (research adj3 (integrati* or overview*))).ti,ab,kf,kw. | 11957 |
| 35 | ((integrative adj3 (review* or overview*)) or (collaborative adj3 (review* or overview*)) or (pool* adj3 analy*)).ti,ab,kf,kw. | 29667 |
| 36 | (data synthes* or data extraction* or data abstraction*).ti,ab,kf,kw. | 30033 |
| 37 | (handsearch* or hand search*).ti,ab,kf,kw. | 9705 |
| 38 | (mantel haenszel or peto or der simonian or dersimonian or fixed effect* or latin square*).ti,ab,kf,kw. | 28453 |
| 39 | (met analy* or metanaly* or technology assessment* or HTA or HTAs or technology overview* or technology appraisal*).ti,ab,kf,kw. | 9963 |
| 40 | (meta regression* or metaregression*).ti,ab,kf,kw. (10163) | 10163 |
| 41 | (meta-analy* or metaanaly* or systematic review* or biomedical technology assessment* or bio-medical technology assessment*).mp,hw. | 342728 |
| 42 | (medline or cochrane or pubmed or medlars or embase or cinahl).ti,ab,hw. | 248140 |
| 43 | (cochrane or (health adj2 technology assessment) or evidence report).jw. | 19999 |
| 44 | (comparative adj3 (efficacy or effectiveness)).ti,ab,kf,kw. | 14425 |
| 45 | (outcomes research or relative effectiveness).ti,ab,kf,kw. | 9841 |
| 46 | ((indirect or indirect treatment or mixed-treatment) adj comparison*).ti,ab,kf,kw. | 2302 |
| 47 | 31 or 32 or 33 or 34 or 35 or 36 or 37 or 38 or 39 or 40 or 41 or 42 or 43 or 44 or 45 or 46 | 516705 |
| 48 | Case-Control Studies/ or Control Groups/ or Matched-Pair Analysis/ or ((case* adj5 control*) or (case adj3 comparison*) or control group*).ti,ab,kw. | 823084 |
| 49 | cohort studies/ or longitudinal studies/ or follow-up studies/ or prospective studies/ or retrospective studies/ or cohort.ti,ab. or longitudinal.ti,ab. or prospective.ti,ab. or retrospective.ti,ab. | 2795784 |
| 50 | (autobiography or bibliography or biography or case reports or classical article or letter or patient education handout or dictionary or directory or editorial or historical article or interactive tutorial or interview or introductory journal article or lecture or legal case or legislation or news or newspaper article or personal narrative or portrait or video audio media or webcast).pt. | 4310851 |
| 51 | exp animals/ not humans.sh. | 4799766 |
| 52 | (exp infant/ or exp child/ or adolescent/) not exp adult/ | 1918444 |
| 53 | 50 or 51 or 52 | 10405865 |
| 54 | ((incisional or postoperative or post-operative or ventral) adj3 hernia?).ab,kf,kw,ti. | 8407 |
| 55 | hernia, ventral/ or incisional hernia/ | 7067 |
| 56 | 54 or 55 | 11266 |
| 57 | (mesh or meshes).ab,kf,kw,ti. | 40970 |
| 58 | Surgical Mesh/ | 14242 |
| 59 | prosthes#s.ab,kf,kw,ti. | 89390 |
| 60 | 57 or 58 or 59 | 132491 |
| 61 | Suture Techniques/ | 43336 |
| 62 | Sutures/ | 17441 |
| 63 | (suture? or suturing).ab,kf,kw,ti. | 78857 |
| 64 | (non-mesh or "non mesh" or "without mesh" or non-meshes or "non meshes" or "without meshes" or "without surgical mesh" or "without surgical meshes").ab,kf,kw,ti. | 588 |
| 65 | primary repair?.ab,kf,kw,ti. | 4781 |
| 66 | tissue repair?.ab,kf,kw,ti. | 13374 |
| 67 | herniotom*.ab,kf,kw,ti. | 677 |
| 68 | 61 or 62 or 63 or 64 or 65 or 66 or 67 | 123043 |
| 69 | 56 and 60 and 68 | 1183 |
| 70 | 69 not 53 | 942 |
| 71 | limit 70 to english language | 784 |
| 72 | 30 and 71 | 159 |
| 73 | 47 and 71 | 63 |
| 74 | 48 and 71 | 29 |
| 75 | 49 and 71 | 429 |

**Embase <1974 to 2021 March 12> (Ovid)**

**Search was conducted on 14^th^ March 2021.**

| # | search string | # of results |
| --- | --- | --- |
| 1 | (Randomized Controlled Trial or Controlled Clinical Trial or Pragmatic Clinical Trial or Equivalence Trial or Clinical Trial, Phase III).pt. | 0 |
| 2 | Randomized Controlled Trial/ | 651778 |
| 3 | exp Randomized Controlled Trials as Topic/ | 198855 |
| 4 | "Randomized Controlled Trial (topic)"/ | 198855 |
| 5 | Controlled Clinical Trial/ | 466838 |
| 6 | exp Controlled Clinical Trials as Topic/ | 206807 |
| 7 | "Controlled Clinical Trial (topic)"/ | 11531 |
| 8 | Randomization/ | 90619 |
| 9 | Random Allocation/ | 86809 |
| 10 | Double-Blind Method/ | 158430 |
| 11 | Double Blind Procedure/ | 182771 |
| 12 | Double-Blind Studies/ | 142095 |
| 13 | Single-Blind Method/ | 40318 |
| 14 | Single Blind Procedure/ | 42335 |
| 15 | Single-Blind Studies/ | 42335 |
| 16 | Placebos/ | 309399 |
| 17 | Placebo/ | 365195 |
| 18 | Control Groups/ | 110539 |
| 19 | Control Group/ | 110539 |
| 20 | (random* or sham or placebo*).ti,ab,hw,kw. | 2172603 |
| 21 | ((singl* or doubl*) adj (blind* or dumm* or mask*)).ti,ab,hw,kw. | 320093 |
| 22 | ((tripl* or trebl*) adj (blind* or dumm* or mask*)).ti,ab,hw,kw. | 1517 |
| 23 | (control* adj3 (study or studies or trial* or group*)).ti,ab,kw. | 1457801 |
| 24 | (Nonrandom* or non random* or non-random* or quasi-random* or quasirandom*).ti,ab,hw,kw. | 58796 |
| 25 | allocated.ti,ab,hw. | 90951 |
| 26 | ((open label or open-label) adj5 (study or studies or trial*)).ti,ab,hw,kw. | 68645 |
| 27 | ((equivalence or superiority or non-inferiority or noninferiority) adj3 (study or studies or trial*)).ti,ab,hw,kw. | 13672 |
| 28 | (pragmatic study or pragmatic studies).ti,ab,hw,kw. | 674 |
| 29 | ((pragmatic or practical) adj3 trial*).ti,ab,hw,kw. | 6189 |
| 30 | ((quasiexperimental or quasi-experimental) adj3 (study or studies or trial*)).ti,ab,hw,kw. | 14063 |
| 31 | (phase adj3 (III or "3") adj3 (study or studies or trial*)).ti,hw,kw. | 98966 |
| 32 | 1 or 2 or 3 or 4 or 5 or 6 or 7 or 8 or 9 or 10 or 11 or 12 or 13 or 14 or 15 or 16 or 17 or 18 or 19 or 20 or 21 or 22 or 23 or 24 or 25 or 26 or 27 or 28 or 29 or 30 or 31 | 3232963 |
| 33 | meta-analysis.pt. | 0 |
| 34 | meta-analysis/ or systematic review/ or meta-analysis as topic/ or "meta analysis (topic)"/ or "systematic review (topic)"/ or exp technology assessment, biomedical/ | 455174 |
| 35 | ((systematic* adj3 (review* or overview*)) or (methodologic* adj3 (review* or overview*))).ti,ab,kw. | 274697 |
| 36 | ((quantitative adj3 (review* or overview* or synthes*)) or (research adj3 (integrati* or overview*))).ti,ab,kw. | 14248 |
| 37 | ((integrative adj3 (review* or overview*)) or (collaborative adj3 (review* or overview*)) or (pool* adj3 analy*)).ti,ab,kw. | 42507 |
| 38 | (data synthes* or data extraction* or data abstraction*).ti,ab,kw. | 37317 |
| 39 | (handsearch* or hand search*).ti,ab,kw. | 11847 |
| 40 | (mantel haenszel or peto or der simonian or dersimonian or fixed effect* or latin square*).ti,ab,kw. | 37755 |
| 41 | (met analy* or metanaly* or technology assessment* or HTA or HTAs or technology overview* or technology appraisal*).ti,ab,kw. | 16298 |
| 42 | (meta regression* or metaregression*).ti,ab,kw. | 12758 |
| 43 | (meta-analy* or metaanaly* or systematic review* or biomedical technology assessment* or bio-medical technology assessment*).mp,hw. | 547838 |
| 44 | (medline or cochrane or pubmed or medlars or embase or cinahl).ti,ab,hw. | 328261 |
| 45 | (cochrane or (health adj2 technology assessment) or evidence report).jx. | 27593 |
| 46 | (comparative adj3 (efficacy or effectiveness)).ti,ab,kw. | 21142 |
| 47 | (outcomes research or relative effectiveness).ti,ab,kw. | 14232 |
| 48 | ((indirect or indirect treatment or mixed-treatment) adj comparison*).ti,ab,kw. | 4384 |
| 49 | 33 or 34 or 35 or 36 or 37 or 38 or 39 or 40 or 41 or 42 or 43 or 44 or 45 or 46 or 47 or 48 | 756660 |
| 50 | exp case control study/ or control group/ or statistical analysis/ or ((case* adj5 control*) or (case adj3 comparison*) or control group*).ti,kw,ab. | 1227352 |
| 51 | cohort analysis/ or longitudinal study/ or follow up/ or prospective study/ or retrospective study/ or cohort.ti,ab. or longitudinal.ti,ab. or prospective.ti,ab. or retrospective.ti,ab. | 4290705 |
| 52 | (conference abstract or "conference review" or editorial or letter or note or short survey).pt. | 7133520 |
| 53 | (exp animal/ or animal experiment/ or nonhuman/) not (exp human/ or human experiment/) | 6666363 |
| 54 | exp juvenile/ not exp adult/ | 2278466 |
| 55 | 52 or 53 or 54 | 14870312 |
| 56 | ((incisional or postoperative or post-operative or ventral) adj3 hernia?).ab,kw,ti. | 12011 |
| 57 | abdominal wall hernia/ or incisional hernia/ | 15742 |
| 58 | 56 or 57 | 18639 |
| 59 | (mesh or meshes).ab,kw,ti. | 59999 |
| 60 | exp surgical mesh/ | 18998 |
| 61 | prosthes#s.ab,kw,ti. | 104577 |
| 62 | 59 or 60 or 61 | 171871 |
| 63 | suture technique/ | 4615 |
| 64 | exp suture/ | 65380 |
| 65 | (suture? or suturing).ab,kw,ti. | 105034 |
| 66 | (non-mesh or "non mesh" or "without mesh" or non-meshes or "non meshes" or "without meshes" or "without surgical mesh" or "without surgical meshes").ab,kw,ti. | 1286 |
| 67 | primary repair?.ab,kw,ti. | 6284 |
| 68 | tissue repair?.ab,kw,ti. | 17940 |
| 69 | herniotom*.ab,kw,ti. | 791 |
| 70 | 63 or 64 or 65 or 66 or 67 or 68 or 69 | 156021 |
| 71 | 58 and 62 and 70 | 1905 |
| 72 | 71 not 55 | 1056 |
| 73 | limit 72 to english language | 932 |
| 74 | 32 and 73 | 174 |
| 75 | 49 and 73 | 79 |
| 76 | 50 and 73 | 39 |
| 77 | 51 and 73 | 534 |

**Cochrane Library**

[**Cochrane Database of Systematic Reviews**](https://www.cochranelibrary.com/)**, Issue 3 of 12, March 2021**

**Cochrane Central Register of Controlled Trials, Issue 3 of 12, March 2021**

**Search was conducted on 18^th^ March 2021.**

| **#** | **search string** | **# of results** |
| --- | --- | --- |
| **1** | ((incisional or postoperative or post-operative or ventral) NEAR/3 hernia?):ti,ab,kw | 1196 |
| **2** | MeSH descriptor: [Hernia, Ventral] this term only | 268 |
| **3** | MeSH descriptor: [Incisional Hernia] this term only | 127 |
| **4** | #1 or #2 or #3 | 1196 |
| **5** | (mesh or meshes):ti,ab,kw | 3399 |
| **6** | MeSH descriptor: [Surgical Mesh] this term only | 747 |
| **7** | (prosthesis or prostheses):ti,ab,kw | 13115 |
| **8** | #5 OR #6 OR #7 | 16261 |
| **9** | MeSH descriptor: [Suture Techniques] this term only | 1793 |
| **10** | MeSH descriptor: [Sutures] this term only | 926 |
| **11** | (suture? or suturing):ti,ab,kw | 8902 |
| **12** | (non-mesh or "non mesh" or "without mesh" or non-meshes or "non meshes" or "without meshes" or "without surgical mesh" or "without surgical meshes"):ti,ab,kw | 140 |
| **13** | (primary NEXT repair?):ti,ab,kw | 206 |
| **14** | (tissue NEXT repair?):ti,ab,kw | 509 |
| **15** | (herniotom*):ti,ab,kw | 194 |
| **16** | #9 OR #10 OR #11 OR #12 OR #13 OR #14 OR #15 | 9820 |
| **17** | #4 and #8 and #16 | 205 |
| **18** | #17 AND Cochrane Database of Systematic Reviews Publication | 3 |
| **19** | #17 AND Cochrane Central Register of Controlled Trials Date | 202 |

**KQ 7: What is the difference in outcome considering different positions of mesh in incisional hernia repair?**

**MEDLINE(R) ALL <** **1946 to March 15, 2021> (Ovid)**

**Search was conducted on 16^th^ March 2021.**

| # | search string | # of results |
| --- | --- | --- |
| 1 | (Randomized Controlled Trial or Controlled Clinical Trial or Pragmatic Clinical Trial or Equivalence Trial or Clinical Trial, Phase III).pt. | 618044 |
| 2 | Randomized Controlled Trial/ | 525030 |
| 3 | exp Randomized Controlled Trials as Topic/ | 144810 |
| 4 | Controlled Clinical Trial/ | 94095 |
| 5 | exp Controlled Clinical Trials as Topic/ | 150296 |
| 6 | Randomization/ | 104842 |
| 7 | Random Allocation/ | 104842 |
| 8 | Double-Blind Method/ | 162935 |
| 9 | Double Blind Procedure/ | 0 |
| 10 | Double-Blind Studies/ | 162935 |
| 11 | Single-Blind Method/ | 29867 |
| 12 | Single Blind Procedure/ | 0 |
| 13 | Single-Blind Studies/ | 29867 |
| 14 | Placebos/ | 35377 |
| 15 | Placebo/ | 0 |
| 16 | Control Groups/ | 1725 |
| 17 | Control Group/ | 1725 |
| 18 | (random* or sham or placebo*).ti,ab,hw,kf,kw. | 1572351 |
| 19 | ((singl* or doubl*) adj (blind* or dumm* or mask*)).ti,ab,hw,kf,kw. | 244459 |
| 20 | ((tripl* or trebl*) adj (blind* or dumm* or mask*)).ti,ab,hw,kf,kw. | 1144 |
| 21 | (control* adj3 (study or studies or trial* or group*)).ti,ab,kf,kw. | 1039014 |
| 22 | (Nonrandom* or non random* or non-random* or quasi-random* or quasirandom*).ti,ab,hw,kf,kw. | 46532 |
| 23 | allocated.ti,ab,hw. | 70017 |
| 24 | ((open label or open-label) adj5 (study or studies or trial*)).ti,ab,hw,kf,kw. | 36955 |
| 25 | ((equivalence or superiority or non-inferiority or noninferiority) adj3 (study or studies or trial*)).ti,ab,hw,kf,kw. | 9227 |
| 26 | (pragmatic study or pragmatic studies).ti,ab,hw,kf,kw. | 451 |
| 27 | ((pragmatic or practical) adj3 trial*).ti,ab,hw,kf,kw. | 5818 |
| 28 | ((quasiexperimental or quasi-experimental) adj3 (study or studies or trial*)).ti,ab,hw,kf,kw. | 8739 |
| 29 | (phase adj3 (III or "3") adj3 (study or studies or trial*)).ti,hw,kf,kw. | 30001 |
| 30 | 1 or 2 or 3 or 4 or 5 or 6 or 7 or 8 or 9 or 10 or 11 or 12 or 13 or 14 or 15 or 16 or 17 or 18 or 19 or 20 or 21 or 22 or 23 or 24 or 25 or 26 or 27 or 28 or 29 | 2258738 |
| 31 | meta-analysis.pt. | 128007 |
| 32 | meta-analysis/ or systematic review/ or meta-analysis as topic/ or "meta analysis (topic)"/ or "systematic review (topic)"/ or exp technology assessment, biomedical/ | 241293 |
| 33 | ((systematic* adj3 (review* or overview*)) or (methodologic* adj3 (review* or overview*))).ti,ab,kf,kw. | 219099 |
| 34 | ((quantitative adj3 (review* or overview* or synthes*)) or (research adj3 (integrati* or overview*))).ti,ab,kf,kw. | 11957 |
| 35 | ((integrative adj3 (review* or overview*)) or (collaborative adj3 (review* or overview*)) or (pool* adj3 analy*)).ti,ab,kf,kw. | 29667 |
| 36 | (data synthes* or data extraction* or data abstraction*).ti,ab,kf,kw. | 30033 |
| 37 | (handsearch* or hand search*).ti,ab,kf,kw. | 9705 |
| 38 | (mantel haenszel or peto or der simonian or dersimonian or fixed effect* or latin square*).ti,ab,kf,kw. | 28453 |
| 39 | (met analy* or metanaly* or technology assessment* or HTA or HTAs or technology overview* or technology appraisal*).ti,ab,kf,kw. | 9963 |
| 40 | (meta regression* or metaregression*).ti,ab,kf,kw. | 10163 |
| 41 | (meta-analy* or metaanaly* or systematic review* or biomedical technology assessment* or bio-medical technology assessment*).mp,hw. | 342728 |
| 42 | (medline or cochrane or pubmed or medlars or embase or cinahl).ti,ab,hw. | 248140 |
| 43 | (cochrane or (health adj2 technology assessment) or evidence report).jw. | 19999 |
| 44 | (comparative adj3 (efficacy or effectiveness)).ti,ab,kf,kw. | 14425 |
| 45 | (outcomes research or relative effectiveness).ti,ab,kf,kw. | 9841 |
| 46 | ((indirect or indirect treatment or mixed-treatment) adj comparison*).ti,ab,kf,kw. | 2302 |
| 47 | 31 or 32 or 33 or 34 or 35 or 36 or 37 or 38 or 39 or 40 or 41 or 42 or 43 or 44 or 45 or 46 | 516705 |
| 48 | Case-Control Studies/ or Control Groups/ or Matched-Pair Analysis/ or ((case* adj5 control*) or (case adj3 comparison*) or control group*).ti,ab,kw. | 823084 |
| 49 | cohort studies/ or longitudinal studies/ or follow-up studies/ or prospective studies/ or retrospective studies/ or cohort.ti,ab. or longitudinal.ti,ab. or prospective.ti,ab. or retrospective.ti,ab. | 2795784 |
| 50 | (autobiography or bibliography or biography or case reports or classical article or letter or patient education handout or dictionary or directory or editorial or historical article or interactive tutorial or interview or introductory journal article or lecture or legal case or legislation or news or newspaper article or personal narrative or portrait or video audio media or webcast).pt. | 4310851 |
| 51 | exp animals/ not humans.sh. | 4799766 |
| 52 | (exp infant/ or exp child/ or adolescent/) not exp adult/ | 1918444 |
| 53 | 50 or 51 or 52 | 10405865 |
| 54 | ((incisional or postoperative or post-operative or ventral) adj3 hernia?).ab,kf,kw,ti. | 8407 |
| 55 | hernia, ventral/ or incisional hernia/ | 7067 |
| 56 | 54 or 55 | 11266 |
| 57 | (onlay or inlay or retrorectus or retro-rectus or retromuscular or retro-muscular or preperitoneal or Rives Stoppa or sublay or underlay or intraperitoneal or intra-peritoneal or IPOM).ab,kf,kw,ti. | 90071 |
| 58 | 56 and 57 | 1486 |
| 59 | 58 not 53 | 1172 |
| 60 | limit 59 to english language | 1022 |
| 61 | 30 and 60 | 181 |
| 62 | 47 and 60 | 62 |
| 63 | 48 and 60 | 36 |
| 64 | 49 and 60 | 641 |

**Embase <1974 to 2021 March 12> (Ovid)**

**Search was conducted on 14^th^ March 2021.**

| # | search string | # of results |
| --- | --- | --- |
| 1 | (Randomized Controlled Trial or Controlled Clinical Trial or Pragmatic Clinical Trial or Equivalence Trial or Clinical Trial, Phase III).pt. | 0 |
| 2 | Randomized Controlled Trial/ | 651778 |
| 3 | exp Randomized Controlled Trials as Topic/ | 198855 |
| 4 | "Randomized Controlled Trial (topic)"/ | 198855 |
| 5 | Controlled Clinical Trial/ | 466838 |
| 6 | exp Controlled Clinical Trials as Topic/ | 206807 |
| 7 | "Controlled Clinical Trial (topic)"/ | 11531 |
| 8 | Randomization/ | 90619 |
| 9 | Random Allocation/ | 86809 |
| 10 | Double-Blind Method/ | 158430 |
| 11 | Double Blind Procedure/ | 182771 |
| 12 | Double-Blind Studies/ | 142095 |
| 13 | Single-Blind Method/ | 40318 |
| 14 | Single Blind Procedure/ | 42335 |
| 15 | Single-Blind Studies/ | 42335 |
| 16 | Placebos/ | 309399 |
| 17 | Placebo/ | 365195 |
| 18 | Control Groups/ | 110539 |
| 19 | Control Group/ | 110539 |
| 20 | (random* or sham or placebo*).ti,ab,hw,kw. | 2172603 |
| 21 | ((singl* or doubl*) adj (blind* or dumm* or mask*)).ti,ab,hw,kw. | 320093 |
| 22 | ((tripl* or trebl*) adj (blind* or dumm* or mask*)).ti,ab,hw,kw. | 1517 |
| 23 | (control* adj3 (study or studies or trial* or group*)).ti,ab,kw. | 1457801 |
| 24 | (Nonrandom* or non random* or non-random* or quasi-random* or quasirandom*).ti,ab,hw,kw. | 58796 |
| 25 | allocated.ti,ab,hw. | 90951 |
| 26 | ((open label or open-label) adj5 (study or studies or trial*)).ti,ab,hw,kw. | 68645 |
| 27 | ((equivalence or superiority or non-inferiority or noninferiority) adj3 (study or studies or trial*)).ti,ab,hw,kw. | 13672 |
| 28 | (pragmatic study or pragmatic studies).ti,ab,hw,kw. | 674 |
| 29 | ((pragmatic or practical) adj3 trial*).ti,ab,hw,kw. | 6189 |
| 30 | ((quasiexperimental or quasi-experimental) adj3 (study or studies or trial*)).ti,ab,hw,kw. | 14063 |
| 31 | (phase adj3 (III or "3") adj3 (study or studies or trial*)).ti,hw,kw. | 98966 |
| 32 | 1 or 2 or 3 or 4 or 5 or 6 or 7 or 8 or 9 or 10 or 11 or 12 or 13 or 14 or 15 or 16 or 17 or 18 or 19 or 20 or 21 or 22 or 23 or 24 or 25 or 26 or 27 or 28 or 29 or 30 or 31 | 3232963 |
| 33 | meta-analysis.pt. | 0 |
| 34 | meta-analysis/ or systematic review/ or meta-analysis as topic/ or "meta analysis (topic)"/ or "systematic review (topic)"/ or exp technology assessment, biomedical/ | 455174 |
| 35 | ((systematic* adj3 (review* or overview*)) or (methodologic* adj3 (review* or overview*))).ti,ab,kw. | 274697 |
| 36 | ((quantitative adj3 (review* or overview* or synthes*)) or (research adj3 (integrati* or overview*))).ti,ab,kw. | 14248 |
| 37 | ((integrative adj3 (review* or overview*)) or (collaborative adj3 (review* or overview*)) or (pool* adj3 analy*)).ti,ab,kw. | 42507 |
| 38 | (data synthes* or data extraction* or data abstraction*).ti,ab,kw. | 37317 |
| 39 | (handsearch* or hand search*).ti,ab,kw. | 11847 |
| 40 | (mantel haenszel or peto or der simonian or dersimonian or fixed effect* or latin square*).ti,ab,kw. | 37755 |
| 41 | (met analy* or metanaly* or technology assessment* or HTA or HTAs or technology overview* or technology appraisal*).ti,ab,kw. | 16298 |
| 42 | (meta regression* or metaregression*).ti,ab,kw. | 12758 |
| 43 | (meta-analy* or metaanaly* or systematic review* or biomedical technology assessment* or bio-medical technology assessment*).mp,hw. | 547838 |
| 44 | (medline or cochrane or pubmed or medlars or embase or cinahl).ti,ab,hw. | 328261 |
| 45 | (cochrane or (health adj2 technology assessment) or evidence report).jx. | 27593 |
| 46 | (comparative adj3 (efficacy or effectiveness)).ti,ab,kw. | 21142 |
| 47 | (outcomes research or relative effectiveness).ti,ab,kw. | 14232 |
| 48 | ((indirect or indirect treatment or mixed-treatment) adj comparison*).ti,ab,kw. | 4384 |
| 49 | 33 or 34 or 35 or 36 or 37 or 38 or 39 or 40 or 41 or 42 or 43 or 44 or 45 or 46 or 47 or 48 | 756660 |
| 50 | exp case control study/ or control group/ or statistical analysis/ or ((case* adj5 control*) or (case adj3 comparison*) or control group*).ti,kw,ab. | 1227352 |
| 51 | cohort analysis/ or longitudinal study/ or follow up/ or prospective study/ or retrospective study/ or cohort.ti,ab. or longitudinal.ti,ab. or prospective.ti,ab. or retrospective.ti,ab. | 4290705 |
| 52 | (conference abstract or "conference review" or editorial or letter or note or short survey).pt. | 7133520 |
| 53 | (exp animal/ or animal experiment/ or nonhuman/) not (exp human/ or human experiment/) | 6666363 |
| 54 | exp juvenile/ not exp adult/ | 2278466 |
| 55 | 52 or 53 or 54 | 14870312 |
| 56 | ((incisional or postoperative or post-operative or ventral) adj3 hernia?).ab,kw,ti. | 12011 |
| 57 | abdominal wall hernia/ or incisional hernia/ | 15742 |
| 58 | 56 or 57 | 18639 |
| 59 | (onlay or inlay or retrorectus or retro-rectus or retromuscular or retro-muscular or preperitoneal or Rives Stoppa or sublay or underlay or intraperitoneal or intra-peritoneal or IPOM).ab,kw,ti. | 118237 |
| 60 | 58 and 59 | 2458 |
| 61 | 60 not 55 | 1441 |
| 62 | limit 61 to english language | 1234 |
| 63 | 32 and 62 | 200 |
| 64 | 49 and 62 | 63 |
| 65 | 50 and 62 | 38 |
| 66 | 51 and 62 | 775 |

**Cochrane Library**

[**Cochrane Database of Systematic Reviews**](https://www.cochranelibrary.com/)**, Issue 3 of 12, March 2021**

**Cochrane Central Register of Controlled Trials, Issue 3 of 12, March 2021**

**Search was conducted on 18^th^ March 2021.**

| **#** | **search string** | **# of results** |
| --- | --- | --- |
| **1** | ((incisional or postoperative or post-operative or ventral) NEAR/3 hernia?):ti,ab,kw | 1196 |
| **2** | MeSH descriptor: [Hernia, Ventral] this term only | 268 |
| **3** | MeSH descriptor: [Incisional Hernia] this term only | 127 |
| **4** | #1 or #2 or #3 | 1196 |
| **5** | (onlay or inlay or retrorectus or retro-rectus or retromuscular or retro-muscular or preperitoneal or Rives Stoppa or sublay or underlay or intraperitoneal or intra-peritoneal or IPOM):ti,ab,kw | 3936 |
| **6** | #4 and #5 | 250 |
| **7** | #6 AND Cochrane Database of Systematic Reviews Publication | 1 |
| **8** | #6 AND Cochrane Central Register of Controlled Trials Date | 249 |

**KQ 8: What is the difference in outcome between techniques for incisional hernia repair?**

**MEDLINE(R) ALL <1946 to March 15, 2021> (Ovid)**

**Search was conducted on 16^th^ March 2021.**

| # | search string | # of results |
| --- | --- | --- |
| 1 | (Randomized Controlled Trial or Controlled Clinical Trial or Pragmatic Clinical Trial or Equivalence Trial or Clinical Trial, Phase III).pt. | 618044 |
| 2 | Randomized Controlled Trial/ | 525030 |
| 3 | exp Randomized Controlled Trials as Topic/ | 144810 |
| 4 | Controlled Clinical Trial/ | 94095 |
| 5 | exp Controlled Clinical Trials as Topic/ | 150296 |
| 6 | Randomization/ | 104842 |
| 7 | Random Allocation/ | 104842 |
| 8 | Double-Blind Method/ | 162935 |
| 9 | Double Blind Procedure/ | 0 |
| 10 | Double-Blind Studies/ | 162935 |
| 11 | Single-Blind Method/ | 29867 |
| 12 | Single Blind Procedure/ | 0 |
| 13 | Single-Blind Studies/ | 29867 |
| 14 | Placebos/ | 35377 |
| 15 | Placebo/ | 0 |
| 16 | Control Groups/ | 1725 |
| 17 | Control Group/ | 1725 |
| 18 | (random* or sham or placebo*).ti,ab,hw,kf,kw. | 1572351 |
| 19 | ((singl* or doubl*) adj (blind* or dumm* or mask*)).ti,ab,hw,kf,kw. | 244459 |
| 20 | ((tripl* or trebl*) adj (blind* or dumm* or mask*)).ti,ab,hw,kf,kw. | 1144 |
| 21 | (control* adj3 (study or studies or trial* or group*)).ti,ab,kf,kw. | 1039014 |
| 22 | (Nonrandom* or non random* or non-random* or quasi-random* or quasirandom*).ti,ab,hw,kf,kw. | 46532 |
| 23 | allocated.ti,ab,hw. | 70017 |
| 24 | ((open label or open-label) adj5 (study or studies or trial*)).ti,ab,hw,kf,kw. | 36955 |
| 25 | ((equivalence or superiority or non-inferiority or noninferiority) adj3 (study or studies or trial*)).ti,ab,hw,kf,kw. | 9227 |
| 26 | (pragmatic study or pragmatic studies).ti,ab,hw,kf,kw. | 451 |
| 27 | ((pragmatic or practical) adj3 trial*).ti,ab,hw,kf,kw. | 5818 |
| 28 | ((quasiexperimental or quasi-experimental) adj3 (study or studies or trial*)).ti,ab,hw,kf,kw. | 8739 |
| 29 | (phase adj3 (III or "3") adj3 (study or studies or trial*)).ti,hw,kf,kw. | 30001 |
| 30 | 1 or 2 or 3 or 4 or 5 or 6 or 7 or 8 or 9 or 10 or 11 or 12 or 13 or 14 or 15 or 16 or 17 or 18 or 19 or 20 or 21 or 22 or 23 or 24 or 25 or 26 or 27 or 28 or 29 | 2258738 |
| 31 | meta-analysis.pt. | 128007 |
| 32 | meta-analysis/ or systematic review/ or meta-analysis as topic/ or "meta analysis (topic)"/ or "systematic review (topic)"/ or exp technology assessment, biomedical/ | 241293 |
| 33 | ((systematic* adj3 (review* or overview*)) or (methodologic* adj3 (review* or overview*))).ti,ab,kf,kw. | 219099 |
| 34 | ((quantitative adj3 (review* or overview* or synthes*)) or (research adj3 (integrati* or overview*))).ti,ab,kf,kw. | 11957 |
| 35 | ((integrative adj3 (review* or overview*)) or (collaborative adj3 (review* or overview*)) or (pool* adj3 analy*)).ti,ab,kf,kw. | 29667 |
| 36 | (data synthes* or data extraction* or data abstraction*).ti,ab,kf,kw. | 30033 |
| 37 | (handsearch* or hand search*).ti,ab,kf,kw. | 9705 |
| 38 | (mantel haenszel or peto or der simonian or dersimonian or fixed effect* or latin square*).ti,ab,kf,kw. | 28453 |
| 39 | (met analy* or metanaly* or technology assessment* or HTA or HTAs or technology overview* or technology appraisal*).ti,ab,kf,kw. | 9963 |
| 40 | (meta regression* or metaregression*).ti,ab,kf,kw. | 10163 |
| 41 | (meta-analy* or metaanaly* or systematic review* or biomedical technology assessment* or bio-medical technology assessment*).mp,hw. | 342728 |
| 42 | (medline or cochrane or pubmed or medlars or embase or cinahl).ti,ab,hw. | 248140 |
| 43 | (cochrane or (health adj2 technology assessment) or evidence report).jw. | 19999 |
| 44 | (comparative adj3 (efficacy or effectiveness)).ti,ab,kf,kw. | 14425 |
| 45 | (outcomes research or relative effectiveness).ti,ab,kf,kw. | 9841 |
| 46 | ((indirect or indirect treatment or mixed-treatment) adj comparison*).ti,ab,kf,kw. | 2302 |
| 47 | 31 or 32 or 33 or 34 or 35 or 36 or 37 or 38 or 39 or 40 or 41 or 42 or 43 or 44 or 45 or 46 | 516705 |
| 48 | Case-Control Studies/ or Control Groups/ or Matched-Pair Analysis/ or ((case* adj5 control*) or (case adj3 comparison*) or control group*).ti,ab,kw. | 823084 |
| 49 | cohort studies/ or longitudinal studies/ or follow-up studies/ or prospective studies/ or retrospective studies/ or cohort.ti,ab. or longitudinal.ti,ab. or prospective.ti,ab. or retrospective.ti,ab. | 2795784 |
| 50 | (autobiography or bibliography or biography or case reports or classical article or letter or patient education handout or dictionary or directory or editorial or historical article or interactive tutorial or interview or introductory journal article or lecture or legal case or legislation or news or newspaper article or personal narrative or portrait or video audio media or webcast).pt. | 4310851 |
| 51 | exp animals/ not humans.sh. | 4799766 |
| 52 | (exp infant/ or exp child/ or adolescent/) not exp adult/ | 1918444 |
| 53 | 50 or 51 or 52 | 10405865 |
| 54 | ((incisional or postoperative or post-operative or ventral) adj3 hernia?).ab,kf,kw,ti. | 8407 |
| 55 | hernia, ventral/ or incisional hernia/ | 7067 |
| 56 | 54 or 55 | 11266 |
| 57 | Herniorrhaphy/mt [Methods] | 3485 |
| 58 | ((Laparosc* or open or Robotic or totally extraperitoneal or Less-open or mini-open or Robot-assisted) adj5 (repair? or technique?)).ab,kf,kw,ti. | 38264 |
| 59 | ((surgical or robotic) adj approach*).ab,kf,kw,ti. | 40433 |
| 60 | (TARUP or eTEP or MILOS or IPOM).ti,ab,kf,kw. | 462 |
| 61 | (repair? and technique?).ab,kf,kw,ti. | 49398 |
| 62 | 57 or 58 or 59 or 60 or 61 | 120531 |
| 63 | 56 and 62 | 3720 |
| 64 | 63 not 53 | 2987 |
| 65 | limit 64 to english language | 2733 |
| 66 | 30 and 65 | 404 |
| 67 | 47 and 65 | 215 |
| 68 | 48 and 65 | 86 |
| 69 | 49 and 65 | 1600 |

**Embase <1974 to 2021 March 12> (Ovid)**

**Search was conducted on 14^th^ March 2021.**

| # | search string | # of results |
| --- | --- | --- |
| 1 | (Randomized Controlled Trial or Controlled Clinical Trial or Pragmatic Clinical Trial or Equivalence Trial or Clinical Trial, Phase III).pt. | 0 |
| 2 | Randomized Controlled Trial/ | 651778 |
| 3 | exp Randomized Controlled Trials as Topic/ | 198855 |
| 4 | "Randomized Controlled Trial (topic)"/ | 198855 |
| 5 | Controlled Clinical Trial/ | 466838 |
| 6 | exp Controlled Clinical Trials as Topic/ | 206807 |
| 7 | "Controlled Clinical Trial (topic)"/ | 11531 |
| 8 | Randomization/ | 90619 |
| 9 | Random Allocation/ | 86809 |
| 10 | Double-Blind Method/ | 158430 |
| 11 | Double Blind Procedure/ | 182771 |
| 12 | Double-Blind Studies/ | 142095 |
| 13 | Single-Blind Method/ | 40318 |
| 14 | Single Blind Procedure/ | 42335 |
| 15 | Single-Blind Studies/ | 42335 |
| 16 | Placebos/ | 309399 |
| 17 | Placebo/ | 365195 |
| 18 | Control Groups/ | 110539 |
| 19 | Control Group/ | 110539 |
| 20 | (random* or sham or placebo*).ti,ab,hw,kw. | 2172603 |
| 21 | ((singl* or doubl*) adj (blind* or dumm* or mask*)).ti,ab,hw,kw. | 320093 |
| 22 | ((tripl* or trebl*) adj (blind* or dumm* or mask*)).ti,ab,hw,kw. | 1517 |
| 23 | (control* adj3 (study or studies or trial* or group*)).ti,ab,kw. | 1457801 |
| 24 | (Nonrandom* or non random* or non-random* or quasi-random* or quasirandom*).ti,ab,hw,kw. | 58796 |
| 25 | allocated.ti,ab,hw. | 90951 |
| 26 | ((open label or open-label) adj5 (study or studies or trial*)).ti,ab,hw,kw. | 68645 |
| 27 | ((equivalence or superiority or non-inferiority or noninferiority) adj3 (study or studies or trial*)).ti,ab,hw,kw. | 13672 |
| 28 | (pragmatic study or pragmatic studies).ti,ab,hw,kw. | 674 |
| 29 | ((pragmatic or practical) adj3 trial*).ti,ab,hw,kw. | 6189 |
| 30 | ((quasiexperimental or quasi-experimental) adj3 (study or studies or trial*)).ti,ab,hw,kw. | 14063 |
| 31 | (phase adj3 (III or "3") adj3 (study or studies or trial*)).ti,hw,kw. | 98966 |
| 32 | 1 or 2 or 3 or 4 or 5 or 6 or 7 or 8 or 9 or 10 or 11 or 12 or 13 or 14 or 15 or 16 or 17 or 18 or 19 or 20 or 21 or 22 or 23 or 24 or 25 or 26 or 27 or 28 or 29 or 30 or 31 | 3232963 |
| 33 | meta-analysis.pt. | 0 |
| 34 | meta-analysis/ or systematic review/ or meta-analysis as topic/ or "meta analysis (topic)"/ or "systematic review (topic)"/ or exp technology assessment, biomedical/ | 455174 |
| 35 | ((systematic* adj3 (review* or overview*)) or (methodologic* adj3 (review* or overview*))).ti,ab,kw. | 274697 |
| 36 | ((quantitative adj3 (review* or overview* or synthes*)) or (research adj3 (integrati* or overview*))).ti,ab,kw. | 14248 |
| 37 | ((integrative adj3 (review* or overview*)) or (collaborative adj3 (review* or overview*)) or (pool* adj3 analy*)).ti,ab,kw. | 42507 |
| 38 | (data synthes* or data extraction* or data abstraction*).ti,ab,kw. | 37317 |
| 39 | (handsearch* or hand search*).ti,ab,kw. | 11847 |
| 40 | (mantel haenszel or peto or der simonian or dersimonian or fixed effect* or latin square*).ti,ab,kw. | 37755 |
| 41 | (met analy* or metanaly* or technology assessment* or HTA or HTAs or technology overview* or technology appraisal*).ti,ab,kw. | 16298 |
| 42 | (meta regression* or metaregression*).ti,ab,kw. | 12758 |
| 43 | (meta-analy* or metaanaly* or systematic review* or biomedical technology assessment* or bio-medical technology assessment*).mp,hw. | 547838 |
| 44 | (medline or cochrane or pubmed or medlars or embase or cinahl).ti,ab,hw. | 328261 |
| 45 | (cochrane or (health adj2 technology assessment) or evidence report).jx. | 27593 |
| 46 | (comparative adj3 (efficacy or effectiveness)).ti,ab,kw. | 21142 |
| 47 | (outcomes research or relative effectiveness).ti,ab,kw. | 14232 |
| 48 | ((indirect or indirect treatment or mixed-treatment) adj comparison*).ti,ab,kw. | 4384 |
| 49 | 33 or 34 or 35 or 36 or 37 or 38 or 39 or 40 or 41 or 42 or 43 or 44 or 45 or 46 or 47 or 48 | 756660 |
| 50 | exp case control study/ or control group/ or statistical analysis/ or ((case* adj5 control*) or (case adj3 comparison*) or control group*).ti,kw,ab. | 1227352 |
| 51 | cohort analysis/ or longitudinal study/ or follow up/ or prospective study/ or retrospective study/ or cohort.ti,ab. or longitudinal.ti,ab. or prospective.ti,ab. or retrospective.ti,ab. | 4290705 |
| 52 | (conference abstract or "conference review" or editorial or letter or note or short survey).pt. | 7133520 |
| 53 | (exp animal/ or animal experiment/ or nonhuman/) not (exp human/ or human experiment/) | 6666363 |
| 54 | exp juvenile/ not exp adult/ | 2278466 |
| 55 | 52 or 53 or 54 | 14870312 |
| 56 | ((incisional or postoperative or post-operative or ventral) adj3 hernia?).ab,kw,ti. | 12011 |
| 57 | abdominal wall hernia/ or incisional hernia/ | 15742 |
| 58 | 56 or 57 | 18639 |
| 59 | ((Laparosc* or open or Robotic or totally extraperitoneal or Less-open or mini-open or Robot-assisted) adj5 (repair? or technique?)).ab,kw,ti. | 58017 |
| 60 | ((surgical or robotic) adj approach*).ab,kw,ti. | 56746 |
| 61 | (TARUP or eTEP or MILOS or IPOM).ti,ab,kw. | 656 |
| 62 | (repair? and technique?).ab,kw,ti. | 67545 |
| 63 | 59 or 60 or 61 or 62 | 168109 |
| 64 | 58 and 63 | 5627 |
| 65 | 64 not 55 | 3458 |
| 66 | limit 65 to english language | 3161 |
| 67 | 32 and 66 | 470 |
| 68 | 49 and 66 | 235 |
| 69 | 50 and 66 | 91 |
| 70 | 51 and 66 | 1801 |

**Cochrane Library**

[**Cochrane Database of Systematic Reviews**](https://www.cochranelibrary.com/)**, Issue 3 of 12, March 2021**

**Cochrane Central Register of Controlled Trials, Issue 3 of 12, March 2021**

**Search was conducted on 18^th^ March 2021.**

| **#** | **search string** | **# of results** |
| --- | --- | --- |
| **1** | MeSH descriptor: [Hernia, Ventral] this term only | 268 |
| **2** | MeSH descriptor: [Incisional Hernia] this term only | 127 |
| **3** | #1 or #2 or #3 | 1196 |
| **4** | MeSH descriptor: [Herniorrhaphy] this term only and with qualifier(s): [methods - MT] | 274 |
| **5** | ((Laparosc* or open or Robotic or "totally extraperitoneal" or Less-open or mini-open or Robot-assisted) NEAR/5 (repair? or technique?)):ti,ab,kw | 4044 |
| **6** | ((surgical or robotic) NEXT approach*):ti,ab,kw | 2654 |
| **7** | (TARUP or eTEP or MILOS or IPOM):ti,ab,kw | 89 |
| **8** | (repair? and technique?):ti,ab,kw | 3503 |
| **9** | #5 OR #6 OR #7 OR #8 OR #9 | 9214 |
| **10** | #4 and #10 | 496 |
| **12** | #10 AND Cochrane Database of Systematic Reviews Publication | 8 |
| **13** | #10 AND Cochrane Central Register of Controlled Trials Date | 488 |

**KQ 9: Is there a benefit of primary fascial closure in incisional hernia mesh repair?**

**MEDLINE(R) ALL <1946 to March 15, 2021> (Ovid)**

**Search was conducted on 16^th^ March 2021.**

| # | search string | # of results |
| --- | --- | --- |
| 1 | (Randomized Controlled Trial or Controlled Clinical Trial or Pragmatic Clinical Trial or Equivalence Trial or Clinical Trial, Phase III).pt. | 618044 |
| 2 | Randomized Controlled Trial/ | 525030 |
| 3 | exp Randomized Controlled Trials as Topic/ | 144810 |
| 4 | Controlled Clinical Trial/ | 94095 |
| 5 | exp Controlled Clinical Trials as Topic/ | 150296 |
| 6 | Randomization/ | 104842 |
| 7 | Random Allocation/ | 104842 |
| 8 | Double-Blind Method/ | 162935 |
| 9 | Double Blind Procedure/ | 0 |
| 10 | Double-Blind Studies/ | 162935 |
| 11 | Single-Blind Method/ | 29867 |
| 12 | Single Blind Procedure/ | 0 |
| 13 | Single-Blind Studies/ | 29867 |
| 14 | Placebos/ | 35377 |
| 15 | Placebo/ | 0 |
| 16 | Control Groups/ | 1725 |
| 17 | Control Group/ | 1725 |
| 18 | (random* or sham or placebo*).ti,ab,hw,kf,kw. | 1572351 |
| 19 | ((singl* or doubl*) adj (blind* or dumm* or mask*)).ti,ab,hw,kf,kw. | 244459 |
| 20 | ((tripl* or trebl*) adj (blind* or dumm* or mask*)).ti,ab,hw,kf,kw. | 1144 |
| 21 | (control* adj3 (study or studies or trial* or group*)).ti,ab,kf,kw. | 1039014 |
| 22 | (Nonrandom* or non random* or non-random* or quasi-random* or quasirandom*).ti,ab,hw,kf,kw. | 46532 |
| 23 | allocated.ti,ab,hw. | 70017 |
| 24 | ((open label or open-label) adj5 (study or studies or trial*)).ti,ab,hw,kf,kw. | 36955 |
| 25 | ((equivalence or superiority or non-inferiority or noninferiority) adj3 (study or studies or trial*)).ti,ab,hw,kf,kw. | 9227 |
| 26 | (pragmatic study or pragmatic studies).ti,ab,hw,kf,kw. | 451 |
| 27 | ((pragmatic or practical) adj3 trial*).ti,ab,hw,kf,kw. | 5818 |
| 28 | ((quasiexperimental or quasi-experimental) adj3 (study or studies or trial*)).ti,ab,hw,kf,kw. | 8739 |
| 29 | (phase adj3 (III or "3") adj3 (study or studies or trial*)).ti,hw,kf,kw. | 30001 |
| 30 | 1 or 2 or 3 or 4 or 5 or 6 or 7 or 8 or 9 or 10 or 11 or 12 or 13 or 14 or 15 or 16 or 17 or 18 or 19 or 20 or 21 or 22 or 23 or 24 or 25 or 26 or 27 or 28 or 29 | 2258738 |
| 31 | meta-analysis.pt. | 128007 |
| 32 | meta-analysis/ or systematic review/ or meta-analysis as topic/ or "meta analysis (topic)"/ or "systematic review (topic)"/ or exp technology assessment, biomedical/ | 241293 |
| 33 | ((systematic* adj3 (review* or overview*)) or (methodologic* adj3 (review* or overview*))).ti,ab,kf,kw. | 219099 |
| 34 | ((quantitative adj3 (review* or overview* or synthes*)) or (research adj3 (integrati* or overview*))).ti,ab,kf,kw. | 11957 |
| 35 | ((integrative adj3 (review* or overview*)) or (collaborative adj3 (review* or overview*)) or (pool* adj3 analy*)).ti,ab,kf,kw. | 29667 |
| 36 | (data synthes* or data extraction* or data abstraction*).ti,ab,kf,kw. | 30033 |
| 37 | (handsearch* or hand search*).ti,ab,kf,kw. | 9705 |
| 38 | (mantel haenszel or peto or der simonian or dersimonian or fixed effect* or latin square*).ti,ab,kf,kw. | 28453 |
| 39 | (met analy* or metanaly* or technology assessment* or HTA or HTAs or technology overview* or technology appraisal*).ti,ab,kf,kw. | 9963 |
| 40 | (meta regression* or metaregression*).ti,ab,kf,kw. | 10163 |
| 41 | (meta-analy* or metaanaly* or systematic review* or biomedical technology assessment* or bio-medical technology assessment*).mp,hw. | 342728 |
| 42 | (medline or cochrane or pubmed or medlars or embase or cinahl).ti,ab,hw. | 248140 |
| 43 | (cochrane or (health adj2 technology assessment) or evidence report).jw. | 19999 |
| 44 | (comparative adj3 (efficacy or effectiveness)).ti,ab,kf,kw. | 14425 |
| 45 | (outcomes research or relative effectiveness).ti,ab,kf,kw. | 9841 |
| 46 | ((indirect or indirect treatment or mixed-treatment) adj comparison*).ti,ab,kf,kw. | 2302 |
| 47 | 31 or 32 or 33 or 34 or 35 or 36 or 37 or 38 or 39 or 40 or 41 or 42 or 43 or 44 or 45 or 46 | 516705 |
| 48 | Case-Control Studies/ or Control Groups/ or Matched-Pair Analysis/ or ((case* adj5 control*) or (case adj3 comparison*) or control group*).ti,ab,kw. | 823084 |
| 49 | cohort studies/ or longitudinal studies/ or follow-up studies/ or prospective studies/ or retrospective studies/ or cohort.ti,ab. or longitudinal.ti,ab. or prospective.ti,ab. or retrospective.ti,ab. | 2795784 |
| 50 | (autobiography or bibliography or biography or case reports or classical article or letter or patient education handout or dictionary or directory or editorial or historical article or interactive tutorial or interview or introductory journal article or lecture or legal case or legislation or news or newspaper article or personal narrative or portrait or video audio media or webcast).pt. | 4310851 |
| 51 | exp animals/ not humans.sh. | 4799766 |
| 52 | (exp infant/ or exp child/ or adolescent/) not exp adult/ | 1918444 |
| 53 | 50 or 51 or 52 | 10405865 |
| 54 | ((incisional or postoperative or post-operative or ventral) adj3 hernia?).ab,kf,kw,ti. | 8407 |
| 55 | hernia, ventral/ or incisional hernia/ | 7067 |
| 56 | 54 or 55 | 11266 |
| 57 | ((fascia? or gap? or defect? or sheath?) and (closure? or opposition? or close? or oppose? or approximat*)).ab,kf,kw,ti. | 72511 |
| 58 | (loss of domain or LOD).ab,kf,kw,ti. | 22300 |
| 59 | 57 or 58 | 94675 |
| 60 | 56 and 59 | 1199 |
| 61 | 60 not 53 | 938 |
| 62 | limit 61 to english language | 850 |
| 63 | 30 and 62 | 123 |
| 64 | 47 and 62 | 62 |
| 65 | 48 and 62 | 21 |
| 66 | 49 and 62 | 498 |

**Embase <1974 to 2021 March 12> (Ovid)**

**Search was conducted on 14^th^ March 2021.**

| # | search string | # of results |
| --- | --- | --- |
| 1 | (Randomized Controlled Trial or Controlled Clinical Trial or Pragmatic Clinical Trial or Equivalence Trial or Clinical Trial, Phase III).pt. | 0 |
| 2 | Randomized Controlled Trial/ | 651778 |
| 3 | exp Randomized Controlled Trials as Topic/ | 198855 |
| 4 | "Randomized Controlled Trial (topic)"/ | 198855 |
| 5 | Controlled Clinical Trial/ | 466838 |
| 6 | exp Controlled Clinical Trials as Topic/ | 206807 |
| 7 | "Controlled Clinical Trial (topic)"/ | 11531 |
| 8 | Randomization/ | 90619 |
| 9 | Random Allocation/ | 86809 |
| 10 | Double-Blind Method/ | 158430 |
| 11 | Double Blind Procedure/ | 182771 |
| 12 | Double-Blind Studies/ | 142095 |
| 13 | Single-Blind Method/ | 40318 |
| 14 | Single Blind Procedure/ | 42335 |
| 15 | Single-Blind Studies/ | 42335 |
| 16 | Placebos/ | 309399 |
| 17 | Placebo/ | 365195 |
| 18 | Control Groups/ | 110539 |
| 19 | Control Group/ | 110539 |
| 20 | (random* or sham or placebo*).ti,ab,hw,kw. | 2172603 |
| 21 | ((singl* or doubl*) adj (blind* or dumm* or mask*)).ti,ab,hw,kw. | 320093 |
| 22 | ((tripl* or trebl*) adj (blind* or dumm* or mask*)).ti,ab,hw,kw. | 1517 |
| 23 | (control* adj3 (study or studies or trial* or group*)).ti,ab,kw. | 1457801 |
| 24 | (Nonrandom* or non random* or non-random* or quasi-random* or quasirandom*).ti,ab,hw,kw. | 58796 |
| 25 | allocated.ti,ab,hw. | 90951 |
| 26 | ((open label or open-label) adj5 (study or studies or trial*)).ti,ab,hw,kw. | 68645 |
| 27 | ((equivalence or superiority or non-inferiority or noninferiority) adj3 (study or studies or trial*)).ti,ab,hw,kw. | 13672 |
| 28 | (pragmatic study or pragmatic studies).ti,ab,hw,kw. | 674 |
| 29 | ((pragmatic or practical) adj3 trial*).ti,ab,hw,kw. | 6189 |
| 30 | ((quasiexperimental or quasi-experimental) adj3 (study or studies or trial*)).ti,ab,hw,kw. | 14063 |
| 31 | (phase adj3 (III or "3") adj3 (study or studies or trial*)).ti,hw,kw. | 98966 |
| 32 | 1 or 2 or 3 or 4 or 5 or 6 or 7 or 8 or 9 or 10 or 11 or 12 or 13 or 14 or 15 or 16 or 17 or 18 or 19 or 20 or 21 or 22 or 23 or 24 or 25 or 26 or 27 or 28 or 29 or 30 or 31 | 3232963 |
| 33 | meta-analysis.pt. | 0 |
| 34 | meta-analysis/ or systematic review/ or meta-analysis as topic/ or "meta analysis (topic)"/ or "systematic review (topic)"/ or exp technology assessment, biomedical/ | 455174 |
| 35 | ((systematic* adj3 (review* or overview*)) or (methodologic* adj3 (review* or overview*))).ti,ab,kw. | 274697 |
| 36 | ((quantitative adj3 (review* or overview* or synthes*)) or (research adj3 (integrati* or overview*))).ti,ab,kw. | 14248 |
| 37 | ((integrative adj3 (review* or overview*)) or (collaborative adj3 (review* or overview*)) or (pool* adj3 analy*)).ti,ab,kw. | 42507 |
| 38 | (data synthes* or data extraction* or data abstraction*).ti,ab,kw. | 37317 |
| 39 | (handsearch* or hand search*).ti,ab,kw. | 11847 |
| 40 | (mantel haenszel or peto or der simonian or dersimonian or fixed effect* or latin square*).ti,ab,kw. | 37755 |
| 41 | (met analy* or metanaly* or technology assessment* or HTA or HTAs or technology overview* or technology appraisal*).ti,ab,kw. | 16298 |
| 42 | (meta regression* or metaregression*).ti,ab,kw. | 12758 |
| 43 | (meta-analy* or metaanaly* or systematic review* or biomedical technology assessment* or bio-medical technology assessment*).mp,hw. | 547838 |
| 44 | (medline or cochrane or pubmed or medlars or embase or cinahl).ti,ab,hw. | 328261 |
| 45 | (cochrane or (health adj2 technology assessment) or evidence report).jx. | 27593 |
| 46 | (comparative adj3 (efficacy or effectiveness)).ti,ab,kw. | 21142 |
| 47 | (outcomes research or relative effectiveness).ti,ab,kw. | 14232 |
| 48 | ((indirect or indirect treatment or mixed-treatment) adj comparison*).ti,ab,kw. | 4384 |
| 49 | 33 or 34 or 35 or 36 or 37 or 38 or 39 or 40 or 41 or 42 or 43 or 44 or 45 or 46 or 47 or 48 | 756660 |
| 50 | exp case control study/ or control group/ or statistical analysis/ or ((case* adj5 control*) or (case adj3 comparison*) or control group*).ti,kw,ab. | 1227352 |
| 51 | cohort analysis/ or longitudinal study/ or follow up/ or prospective study/ or retrospective study/ or cohort.ti,ab. or longitudinal.ti,ab. or prospective.ti,ab. or retrospective.ti,ab. | 4290705 |
| 52 | (conference abstract or "conference review" or editorial or letter or note or short survey).pt. | 7133520 |
| 53 | (exp animal/ or animal experiment/ or nonhuman/) not (exp human/ or human experiment/) | 6666363 |
| 54 | exp juvenile/ not exp adult/ | 2278466 |
| 55 | 52 or 53 or 54 | 14870312 |
| 56 | ((incisional or postoperative or post-operative or ventral) adj3 hernia?).ab,kw,ti. | 12011 |
| 57 | abdominal wall hernia/ or incisional hernia/ | 15742 |
| 58 | 56 or 57 | 18639 |
| 59 | ((fascia? or gap? or defect? or sheath?) and (closure? or opposition? or close? or oppose? or approximat*)).ab,kw,ti. | 91573 |
| 60 | (loss of domain or LOD).ab,kw,ti. | 28053 |
| 61 | 59 or 60 | 119452 |
| 62 | 58 and 61 | 2045 |
| 63 | 62 not 55 | 1217 |
| 64 | limit 63 to english language | 1093 |
| 65 | 32 and 64 | 134 |
| 66 | 49 and 64 | 72 |
| 67 | 50 and 64 | 24 |
| 68 | 51 and 64 | 584 |

**Cochrane Library**

[**Cochrane Database of Systematic Reviews**](https://www.cochranelibrary.com/)**, Issue 3 of 12, March 2021**

**Cochrane Central Register of Controlled Trials, Issue 3 of 12, March 2021**

**Search was conducted on 18^th^ March 2021.**

| **#** | **search string** | **# of results** |
| --- | --- | --- |
| **1** | ((incisional or postoperative or post-operative or ventral) NEAR/3 hernia?):ti,ab,kw | 1196 |
| **2** | MeSH descriptor: [Hernia, Ventral] this term only | 268 |
| **3** | MeSH descriptor: [Incisional Hernia] this term only | 127 |
| **4** | #1 or #2 or #3 | 1196 |
| **5** | ((fascia? or gap? or defect? or sheath?) and (closure? or opposition? or close? or oppose? or approximat*)):ti,ab,kw | 3746 |
| **6** | ("loss of domain" or LOD):ti,ab,kw | 237 |
| **7** | #5 or #6 | 3981 |
| **8** | #4 and #7 | 161 |
| **9** | #8 AND Cochrane Database of Systematic Reviews Publication | 2 |
| **10** | #8 AND Cochrane Central Register of Controlled Trials Date | 159 |

**KQ 10: What is the difference in the outcome using different techniques for mesh fixation in: (a) intraperitoneal and (b) extraperitoneal mesh placement for incisional hernia repair?**

**MEDLINE(R) ALL <1946 to March 15, 2021> (Ovid)**

**Search was conducted on 16^th^ March 2021.**

| # | search string | # of results |
| --- | --- | --- |
| 1 | (Randomized Controlled Trial or Controlled Clinical Trial or Pragmatic Clinical Trial or Equivalence Trial or Clinical Trial, Phase III).pt. | 618044 |
| 2 | Randomized Controlled Trial/ | 525030 |
| 3 | exp Randomized Controlled Trials as Topic/ | 144810 |
| 4 | Controlled Clinical Trial/ | 94095 |
| 5 | exp Controlled Clinical Trials as Topic/ | 150296 |
| 6 | Randomization/ | 104842 |
| 7 | Random Allocation/ | 104842 |
| 8 | Double-Blind Method/ | 162935 |
| 9 | Double Blind Procedure/ | 0 |
| 10 | Double-Blind Studies/ | 162935 |
| 11 | Single-Blind Method/ | 29867 |
| 12 | Single Blind Procedure/ | 0 |
| 13 | Single-Blind Studies/ | 29867 |
| 14 | Placebos/ | 35377 |
| 15 | Placebo/ | 0 |
| 16 | Control Groups/ | 1725 |
| 17 | Control Group/ | 1725 |
| 18 | (random* or sham or placebo*).ti,ab,hw,kf,kw. | 1572351 |
| 19 | ((singl* or doubl*) adj (blind* or dumm* or mask*)).ti,ab,hw,kf,kw. | 244459 |
| 20 | ((tripl* or trebl*) adj (blind* or dumm* or mask*)).ti,ab,hw,kf,kw. | 1144 |
| 21 | (control* adj3 (study or studies or trial* or group*)).ti,ab,kf,kw. | 1039014 |
| 22 | (Nonrandom* or non random* or non-random* or quasi-random* or quasirandom*).ti,ab,hw,kf,kw. | 46532 |
| 23 | allocated.ti,ab,hw. | 70017 |
| 24 | ((open label or open-label) adj5 (study or studies or trial*)).ti,ab,hw,kf,kw. | 36955 |
| 25 | ((equivalence or superiority or non-inferiority or noninferiority) adj3 (study or studies or trial*)).ti,ab,hw,kf,kw. | 9227 |
| 26 | (pragmatic study or pragmatic studies).ti,ab,hw,kf,kw. | 451 |
| 27 | ((pragmatic or practical) adj3 trial*).ti,ab,hw,kf,kw. | 5818 |
| 28 | ((quasiexperimental or quasi-experimental) adj3 (study or studies or trial*)).ti,ab,hw,kf,kw. | 8739 |
| 29 | (phase adj3 (III or "3") adj3 (study or studies or trial*)).ti,hw,kf,kw. | 30001 |
| 30 | 1 or 2 or 3 or 4 or 5 or 6 or 7 or 8 or 9 or 10 or 11 or 12 or 13 or 14 or 15 or 16 or 17 or 18 or 19 or 20 or 21 or 22 or 23 or 24 or 25 or 26 or 27 or 28 or 29 | 2258738 |
| 31 | meta-analysis.pt. | 128007 |
| 32 | meta-analysis/ or systematic review/ or meta-analysis as topic/ or "meta analysis (topic)"/ or "systematic review (topic)"/ or exp technology assessment, biomedical/ | 241293 |
| 33 | ((systematic* adj3 (review* or overview*)) or (methodologic* adj3 (review* or overview*))).ti,ab,kf,kw. | 219099 |
| 34 | ((quantitative adj3 (review* or overview* or synthes*)) or (research adj3 (integrati* or overview*))).ti,ab,kf,kw. | 11957 |
| 35 | ((integrative adj3 (review* or overview*)) or (collaborative adj3 (review* or overview*)) or (pool* adj3 analy*)).ti,ab,kf,kw. | 29667 |
| 36 | (data synthes* or data extraction* or data abstraction*).ti,ab,kf,kw. | 30033 |
| 37 | (handsearch* or hand search*).ti,ab,kf,kw. | 9705 |
| 38 | (mantel haenszel or peto or der simonian or dersimonian or fixed effect* or latin square*).ti,ab,kf,kw. | 28453 |
| 39 | (met analy* or metanaly* or technology assessment* or HTA or HTAs or technology overview* or technology appraisal*).ti,ab,kf,kw. | 9963 |
| 40 | (meta regression* or metaregression*).ti,ab,kf,kw. | 10163 |
| 41 | (meta-analy* or metaanaly* or systematic review* or biomedical technology assessment* or bio-medical technology assessment*).mp,hw. | 342728 |
| 42 | (medline or cochrane or pubmed or medlars or embase or cinahl).ti,ab,hw. | 248140 |
| 43 | (cochrane or (health adj2 technology assessment) or evidence report).jw. | 19999 |
| 44 | (comparative adj3 (efficacy or effectiveness)).ti,ab,kf,kw. | 14425 |
| 45 | (outcomes research or relative effectiveness).ti,ab,kf,kw. | 9841 |
| 46 | ((indirect or indirect treatment or mixed-treatment) adj comparison*).ti,ab,kf,kw. | 2302 |
| 47 | 31 or 32 or 33 or 34 or 35 or 36 or 37 or 38 or 39 or 40 or 41 or 42 or 43 or 44 or 45 or 46 | 516705 |
| 48 | Case-Control Studies/ or Control Groups/ or Matched-Pair Analysis/ or ((case* adj5 control*) or (case adj3 comparison*) or control group*).ti,ab,kw. | 823084 |
| 49 | cohort studies/ or longitudinal studies/ or follow-up studies/ or prospective studies/ or retrospective studies/ or cohort.ti,ab. or longitudinal.ti,ab. or prospective.ti,ab. or retrospective.ti,ab. | 2795784 |
| 50 | (autobiography or bibliography or biography or case reports or classical article or letter or patient education handout or dictionary or directory or editorial or historical article or interactive tutorial or interview or introductory journal article or lecture or legal case or legislation or news or newspaper article or personal narrative or portrait or video audio media or webcast).pt. | 4310851 |
| 51 | exp animals/ not humans.sh. | 4799766 |
| 52 | (exp infant/ or exp child/ or adolescent/) not exp adult/ | 1918444 |
| 53 | 50 or 51 or 52 | 10405865 |
| 54 | ((incisional or postoperative or post-operative or ventral) adj3 hernia?).ab,kf,kw,ti. | 8407 |
| 55 | hernia, ventral/ or incisional hernia/ | 7067 |
| 56 | 54 or 55 | 11266 |
| 57 | (mesh or meshes).ab,kf,kw,ti. | 40970 |
| 58 | Surgical Mesh/ | 14242 |
| 59 | prosthes#s.ab,kf,kw,ti. | 89390 |
| 60 | Sutures/ | 17441 |
| 61 | Suture Techniques/ | 43336 |
| 62 | (suture? or suturing).ab,kf,kw,ti. | 78857 |
| 63 | 57 or 58 or 59 or 60 or 61 or 62 | 231860 |
| 64 | fixation?.ab,kf,kw,ti. | 154960 |
| 65 | 63 and 64 | 12335 |
| 66 | (tack? or stapled or mechanical or non-mechanical or "non mechanical" or glue or tacker? or tacking or transfascial or "trans-fascial" or histo-acryl or histoacryl or self-fixing or self-gripping).ab,kf,kw,ti. | 377784 |
| 67 | 65 or 66 | 388604 |
| 68 | 56 and 67 | 754 |
| 69 | 68 not 53 | 522 |
| 70 | limit 69 to english language | 470 |
| 71 | 30 and 70 | 77 |
| 72 | 47 and 70 | 25 |
| 73 | 48 and 70 | 20 |
| 74 | 49 and 70 | 232 |

**Embase <1974 to 2021 March 12> (Ovid)**

**Search was conducted on 14^th^ March 2021.**

| # | search string | # of results |
| --- | --- | --- |
| 1 | (Randomized Controlled Trial or Controlled Clinical Trial or Pragmatic Clinical Trial or Equivalence Trial or Clinical Trial, Phase III).pt. | 0 |
| 2 | Randomized Controlled Trial/ | 651778 |
| 3 | exp Randomized Controlled Trials as Topic/ | 198855 |
| 4 | "Randomized Controlled Trial (topic)"/ | 198855 |
| 5 | Controlled Clinical Trial/ | 466838 |
| 6 | exp Controlled Clinical Trials as Topic/ | 206807 |
| 7 | "Controlled Clinical Trial (topic)"/ | 11531 |
| 8 | Randomization/ | 90619 |
| 9 | Random Allocation/ | 86809 |
| 10 | Double-Blind Method/ | 158430 |
| 11 | Double Blind Procedure/ | 182771 |
| 12 | Double-Blind Studies/ | 142095 |
| 13 | Single-Blind Method/ | 40318 |
| 14 | Single Blind Procedure/ | 42335 |
| 15 | Single-Blind Studies/ | 42335 |
| 16 | Placebos/ | 309399 |
| 17 | Placebo/ | 365195 |
| 18 | Control Groups/ | 110539 |
| 19 | Control Group/ | 110539 |
| 20 | (random* or sham or placebo*).ti,ab,hw,kw. | 2172603 |
| 21 | ((singl* or doubl*) adj (blind* or dumm* or mask*)).ti,ab,hw,kw. | 320093 |
| 22 | ((tripl* or trebl*) adj (blind* or dumm* or mask*)).ti,ab,hw,kw. | 1517 |
| 23 | (control* adj3 (study or studies or trial* or group*)).ti,ab,kw. | 1457801 |
| 24 | (Nonrandom* or non random* or non-random* or quasi-random* or quasirandom*).ti,ab,hw,kw. | 58796 |
| 25 | allocated.ti,ab,hw. | 90951 |
| 26 | ((open label or open-label) adj5 (study or studies or trial*)).ti,ab,hw,kw. | 68645 |
| 27 | ((equivalence or superiority or non-inferiority or noninferiority) adj3 (study or studies or trial*)).ti,ab,hw,kw. | 13672 |
| 28 | (pragmatic study or pragmatic studies).ti,ab,hw,kw. | 674 |
| 29 | ((pragmatic or practical) adj3 trial*).ti,ab,hw,kw. | 6189 |
| 30 | ((quasiexperimental or quasi-experimental) adj3 (study or studies or trial*)).ti,ab,hw,kw. | 14063 |
| 31 | (phase adj3 (III or "3") adj3 (study or studies or trial*)).ti,hw,kw. | 98966 |
| 32 | 1 or 2 or 3 or 4 or 5 or 6 or 7 or 8 or 9 or 10 or 11 or 12 or 13 or 14 or 15 or 16 or 17 or 18 or 19 or 20 or 21 or 22 or 23 or 24 or 25 or 26 or 27 or 28 or 29 or 30 or 31 | 3232963 |
| 33 | meta-analysis.pt. | 0 |
| 34 | meta-analysis/ or systematic review/ or meta-analysis as topic/ or "meta analysis (topic)"/ or "systematic review (topic)"/ or exp technology assessment, biomedical/ | 455174 |
| 35 | ((systematic* adj3 (review* or overview*)) or (methodologic* adj3 (review* or overview*))).ti,ab,kw. | 274697 |
| 36 | ((quantitative adj3 (review* or overview* or synthes*)) or (research adj3 (integrati* or overview*))).ti,ab,kw. | 14248 |
| 37 | ((integrative adj3 (review* or overview*)) or (collaborative adj3 (review* or overview*)) or (pool* adj3 analy*)).ti,ab,kw. | 42507 |
| 38 | (data synthes* or data extraction* or data abstraction*).ti,ab,kw. | 37317 |
| 39 | (handsearch* or hand search*).ti,ab,kw. | 11847 |
| 40 | (mantel haenszel or peto or der simonian or dersimonian or fixed effect* or latin square*).ti,ab,kw. | 37755 |
| 41 | (met analy* or metanaly* or technology assessment* or HTA or HTAs or technology overview* or technology appraisal*).ti,ab,kw. | 16298 |
| 42 | (meta regression* or metaregression*).ti,ab,kw. | 12758 |
| 43 | (meta-analy* or metaanaly* or systematic review* or biomedical technology assessment* or bio-medical technology assessment*).mp,hw. | 547838 |
| 44 | (medline or cochrane or pubmed or medlars or embase or cinahl).ti,ab,hw. | 328261 |
| 45 | (cochrane or (health adj2 technology assessment) or evidence report).jx. | 27593 |
| 46 | (comparative adj3 (efficacy or effectiveness)).ti,ab,kw. | 21142 |
| 47 | (outcomes research or relative effectiveness).ti,ab,kw. | 14232 |
| 48 | ((indirect or indirect treatment or mixed-treatment) adj comparison*).ti,ab,kw. | 4384 |
| 49 | 33 or 34 or 35 or 36 or 37 or 38 or 39 or 40 or 41 or 42 or 43 or 44 or 45 or 46 or 47 or 48 | 756660 |
| 50 | exp case control study/ or control group/ or statistical analysis/ or ((case* adj5 control*) or (case adj3 comparison*) or control group*).ti,kw,ab. | 1227352 |
| 51 | cohort analysis/ or longitudinal study/ or follow up/ or prospective study/ or retrospective study/ or cohort.ti,ab. or longitudinal.ti,ab. or prospective.ti,ab. or retrospective.ti,ab. | 4290705 |
| 52 | (conference abstract or "conference review" or editorial or letter or note or short survey).pt. | 7133520 |
| 53 | (exp animal/ or animal experiment/ or nonhuman/) not (exp human/ or human experiment/) | 6666363 |
| 54 | exp juvenile/ not exp adult/ | 2278466 |
| 55 | 52 or 53 or 54 | 14870312 |
| 56 | ((incisional or postoperative or post-operative or ventral) adj3 hernia?).ab,kw,ti. | 12011 |
| 57 | abdominal wall hernia/ or incisional hernia/ | 15742 |
| 58 | 56 or 57 | 18639 |
| 59 | (mesh or meshes).ab,kw,ti. | 59999 |
| 60 | exp surgical mesh/ | 18998 |
| 61 | prosthes#s.ab,kw,ti. | 104577 |
| 62 | suture technique/ | 4615 |
| 63 | exp suture/ | 65380 |
| 64 | (suture? or suturing).ab,kw,ti. | 105034 |
| 65 | 59 or 60 or 61 or 62 or 63 or 64 | 294062 |
| 66 | fixation?.ab,kw,ti. | 173016 |
| 67 | 65 and 66 | 16197 |
| 68 | (tack? or stapled or mechanical or non-mechanical or "non mechanical" or glue or tacker? or tacking or transfascial or "trans-fascial" or histo-acryl or histoacryl or self-fixing or self-gripping).ab,kw,ti. | 465292 |
| 69 | 67 or 68 | 479267 |
| 70 | 58 and 69 | 1482 |
| 71 | 70 not 55 | 714 |
| 72 | limit 71 to english language | 642 |
| 73 | 32 and 72 | 94 |
| 74 | 49 and 72 | 34 |
| 75 | 50 and 72 | 18 |
| 76 | 51 and 72 | 329 |

**Cochrane Library**

[**Cochrane Database of Systematic Reviews**](https://www.cochranelibrary.com/)**, Issue 3 of 12, March 2021**

**Cochrane Central Register of Controlled Trials, Issue 3 of 12, March 2021**

**Search was conducted on 18^th^ March 2021.**

| **#** | **search string** | **# of results** |
| --- | --- | --- |
| **1** | ((incisional or postoperative or post-operative or ventral) NEAR/3 hernia?):ti,ab,kw | 1196 |
| **2** | MeSH descriptor: [Hernia, Ventral] this term only | 268 |
| **3** | MeSH descriptor: [Incisional Hernia] this term only | 127 |
| **4** | #1 or #2 or #3 | 1196 |
| **5** | (mesh or meshes):ti,ab,kw | 3399 |
| **6** | MeSH descriptor: [Surgical Mesh] this term only | 747 |
| **7** | (prosthesis or prostheses):ti,ab,kw | 13115 |
| **8** | MeSH descriptor: [Suture Techniques] this term only | 1793 |
| **9** | MeSH descriptor: [Sutures] this term only | 926 |
| **10** | (suture? or suturing):ti,ab,kw | 8902 |
| **11** | #5 OR #6 OR #7 OR #8 OR #9 OR #10 | 24260 |
| **12** | (fixation?):ti,ab,kw | 9089 |
| **13** | (tack? or stapled or mechanical or non-mechanical or "non mechanical" or glue or tacker? or tacking or transfascial or "trans-fascial" or histo-acryl or histoacryl or self-fixing or self-gripping):ti,ab,kw | 25223 |
| **14** | #13 or #14 | 26522 |
| **15** | #4 and #15 | 125 |
| **16** | #15 AND Cochrane Database of Systematic Reviews Publication | 2 |
| **17** | #15 AND Cochrane Central Register of Controlled Trials Date | 123 |

**KQ 11: What is the benefit of ERAS in incisional hernia repair?**

**MEDLINE(R) ALL <1946 to March 15, 2021> (Ovid)**

**Search was conducted on 16^th^ March 2021.**

| # | search string | # of results |
| --- | --- | --- |
| 1 | (Randomized Controlled Trial or Controlled Clinical Trial or Pragmatic Clinical Trial or Equivalence Trial or Clinical Trial, Phase III).pt. | 618044 |
| 2 | Randomized Controlled Trial/ | 525030 |
| 3 | exp Randomized Controlled Trials as Topic/ | 144810 |
| 4 | Controlled Clinical Trial/ | 94095 |
| 5 | exp Controlled Clinical Trials as Topic/ | 150296 |
| 6 | Randomization/ | 104842 |
| 7 | Random Allocation/ | 104842 |
| 8 | Double-Blind Method/ | 162935 |
| 9 | Double Blind Procedure/ | 0 |
| 10 | Double-Blind Studies/ | 162935 |
| 11 | Single-Blind Method/ | 29867 |
| 12 | Single Blind Procedure/ | 0 |
| 13 | Single-Blind Studies/ | 29867 |
| 14 | Placebos/ | 35377 |
| 15 | Placebo/ | 0 |
| 16 | Control Groups/ | 1725 |
| 17 | Control Group/ | 1725 |
| 18 | (random* or sham or placebo*).ti,ab,hw,kf,kw. | 1572351 |
| 19 | ((singl* or doubl*) adj (blind* or dumm* or mask*)).ti,ab,hw,kf,kw. | 244459 |
| 20 | ((tripl* or trebl*) adj (blind* or dumm* or mask*)).ti,ab,hw,kf,kw. | 1144 |
| 21 | (control* adj3 (study or studies or trial* or group*)).ti,ab,kf,kw. | 1039014 |
| 22 | (Nonrandom* or non random* or non-random* or quasi-random* or quasirandom*).ti,ab,hw,kf,kw. | 46532 |
| 23 | allocated.ti,ab,hw. | 70017 |
| 24 | ((open label or open-label) adj5 (study or studies or trial*)).ti,ab,hw,kf,kw. | 36955 |
| 25 | ((equivalence or superiority or non-inferiority or noninferiority) adj3 (study or studies or trial*)).ti,ab,hw,kf,kw. | 9227 |
| 26 | (pragmatic study or pragmatic studies).ti,ab,hw,kf,kw. | 451 |
| 27 | ((pragmatic or practical) adj3 trial*).ti,ab,hw,kf,kw. | 5818 |
| 28 | ((quasiexperimental or quasi-experimental) adj3 (study or studies or trial*)).ti,ab,hw,kf,kw. | 8739 |
| 29 | (phase adj3 (III or "3") adj3 (study or studies or trial*)).ti,hw,kf,kw. | 30001 |
| 30 | 1 or 2 or 3 or 4 or 5 or 6 or 7 or 8 or 9 or 10 or 11 or 12 or 13 or 14 or 15 or 16 or 17 or 18 or 19 or 20 or 21 or 22 or 23 or 24 or 25 or 26 or 27 or 28 or 29 | 2258738 |
| 31 | meta-analysis.pt. | 128007 |
| 32 | meta-analysis/ or systematic review/ or meta-analysis as topic/ or "meta analysis (topic)"/ or "systematic review (topic)"/ or exp technology assessment, biomedical/ | 241293 |
| 33 | ((systematic* adj3 (review* or overview*)) or (methodologic* adj3 (review* or overview*))).ti,ab,kf,kw. | 219099 |
| 34 | ((quantitative adj3 (review* or overview* or synthes*)) or (research adj3 (integrati* or overview*))).ti,ab,kf,kw. | 11957 |
| 35 | ((integrative adj3 (review* or overview*)) or (collaborative adj3 (review* or overview*)) or (pool* adj3 analy*)).ti,ab,kf,kw. | 29667 |
| 36 | (data synthes* or data extraction* or data abstraction*).ti,ab,kf,kw. | 30033 |
| 37 | (handsearch* or hand search*).ti,ab,kf,kw. | 9705 |
| 38 | (mantel haenszel or peto or der simonian or dersimonian or fixed effect* or latin square*).ti,ab,kf,kw. | 28453 |
| 39 | (met analy* or metanaly* or technology assessment* or HTA or HTAs or technology overview* or technology appraisal*).ti,ab,kf,kw. | 9963 |
| 40 | (meta regression* or metaregression*).ti,ab,kf,kw. | 10163 |
| 41 | (meta-analy* or metaanaly* or systematic review* or biomedical technology assessment* or bio-medical technology assessment*).mp,hw. | 342728 |
| 42 | (medline or cochrane or pubmed or medlars or embase or cinahl).ti,ab,hw. | 248140 |
| 43 | (cochrane or (health adj2 technology assessment) or evidence report).jw. | 19999 |
| 44 | (comparative adj3 (efficacy or effectiveness)).ti,ab,kf,kw. | 14425 |
| 45 | (outcomes research or relative effectiveness).ti,ab,kf,kw. | 9841 |
| 46 | ((indirect or indirect treatment or mixed-treatment) adj comparison*).ti,ab,kf,kw. | 2302 |
| 47 | 31 or 32 or 33 or 34 or 35 or 36 or 37 or 38 or 39 or 40 or 41 or 42 or 43 or 44 or 45 or 46 | 516705 |
| 48 | Case-Control Studies/ or Control Groups/ or Matched-Pair Analysis/ or ((case* adj5 control*) or (case adj3 comparison*) or control group*).ti,ab,kw. | 823084 |
| 49 | cohort studies/ or longitudinal studies/ or follow-up studies/ or prospective studies/ or retrospective studies/ or cohort.ti,ab. or longitudinal.ti,ab. or prospective.ti,ab. or retrospective.ti,ab. | 2795784 |
| 50 | (autobiography or bibliography or biography or case reports or classical article or letter or patient education handout or dictionary or directory or editorial or historical article or interactive tutorial or interview or introductory journal article or lecture or legal case or legislation or news or newspaper article or personal narrative or portrait or video audio media or webcast).pt. | 4310851 |
| 51 | exp animals/ not humans.sh. | 4799766 |
| 52 | (exp infant/ or exp child/ or adolescent/) not exp adult/ | 1918444 |
| 53 | 50 or 51 or 52 | 10405865 |
| 54 | ((incisional or postoperative or post-operative or ventral) adj3 hernia?).ab,kf,kw,ti. | 8407 |
| 55 | hernia, ventral/ or incisional hernia/ | 7067 |
| 56 | 54 or 55 | 11266 |
| 57 | (ERAS or enhanced recover* or Enhanced Postsurgical Recover* or Enhanced Post-surgical Recover* or Enhanced Post surgical Recover* or Enhanced Postoperative Recover* or Enhanced Post-operative Recover* or Enhanced Post operative Recover*).ab,kf,kw,ti. | 7758 |
| 58 | Enhanced Recovery After Surgery/ | 513 |
| 59 | 57 or 58 | 7809 |
| 60 | 56 and 59 | 33 |
| 61 | 60 not 53 | 30 |
| 62 | limit 61 to english language | 30 |
| 63 | 30 and 62 | 6 |
| 64 | 47 and 62 | 3 |
| 65 | 48 and 62 | 4 |
| 66 | 49 and 62 | 24 |

**MEDLINE(R) ALL <1946 to March 15, 2021> (Ovid)**

**Search was conducted on 16^th^ March 2021.**

| # | search string | # of results |
| --- | --- | --- |
| 1 | (Randomized Controlled Trial or Controlled Clinical Trial or Pragmatic Clinical Trial or Equivalence Trial or Clinical Trial, Phase III).pt. | 618044 |
| 2 | Randomized Controlled Trial/ | 525030 |
| 3 | exp Randomized Controlled Trials as Topic/ | 144810 |
| 4 | Controlled Clinical Trial/ | 94095 |
| 5 | exp Controlled Clinical Trials as Topic/ | 150296 |
| 6 | Randomization/ | 104842 |
| 7 | Random Allocation/ | 104842 |
| 8 | Double-Blind Method/ | 162935 |
| 9 | Double Blind Procedure/ | 0 |
| 10 | Double-Blind Studies/ | 162935 |
| 11 | Single-Blind Method/ | 29867 |
| 12 | Single Blind Procedure/ | 0 |
| 13 | Single-Blind Studies/ | 29867 |
| 14 | Placebos/ | 35377 |
| 15 | Placebo/ | 0 |
| 16 | Control Groups/ | 1725 |
| 17 | Control Group/ | 1725 |
| 18 | (random* or sham or placebo*).ti,ab,hw,kf,kw. | 1572351 |
| 19 | ((singl* or doubl*) adj (blind* or dumm* or mask*)).ti,ab,hw,kf,kw. | 244459 |
| 20 | ((tripl* or trebl*) adj (blind* or dumm* or mask*)).ti,ab,hw,kf,kw. | 1144 |
| 21 | (control* adj3 (study or studies or trial* or group*)).ti,ab,kf,kw. | 1039014 |
| 22 | (Nonrandom* or non random* or non-random* or quasi-random* or quasirandom*).ti,ab,hw,kf,kw. | 46532 |
| 23 | allocated.ti,ab,hw. | 70017 |
| 24 | ((open label or open-label) adj5 (study or studies or trial*)).ti,ab,hw,kf,kw. | 36955 |
| 25 | ((equivalence or superiority or non-inferiority or noninferiority) adj3 (study or studies or trial*)).ti,ab,hw,kf,kw. | 9227 |
| 26 | (pragmatic study or pragmatic studies).ti,ab,hw,kf,kw. | 451 |
| 27 | ((pragmatic or practical) adj3 trial*).ti,ab,hw,kf,kw. | 5818 |
| 28 | ((quasiexperimental or quasi-experimental) adj3 (study or studies or trial*)).ti,ab,hw,kf,kw. | 8739 |
| 29 | (phase adj3 (III or "3") adj3 (study or studies or trial*)).ti,hw,kf,kw. | 30001 |
| 30 | 1 or 2 or 3 or 4 or 5 or 6 or 7 or 8 or 9 or 10 or 11 or 12 or 13 or 14 or 15 or 16 or 17 or 18 or 19 or 20 or 21 or 22 or 23 or 24 or 25 or 26 or 27 or 28 or 29 | 2258738 |
| 31 | meta-analysis.pt. | 128007 |
| 32 | meta-analysis/ or systematic review/ or meta-analysis as topic/ or "meta analysis (topic)"/ or "systematic review (topic)"/ or exp technology assessment, biomedical/ | 241293 |
| 33 | ((systematic* adj3 (review* or overview*)) or (methodologic* adj3 (review* or overview*))).ti,ab,kf,kw. | 219099 |
| 34 | ((quantitative adj3 (review* or overview* or synthes*)) or (research adj3 (integrati* or overview*))).ti,ab,kf,kw. | 11957 |
| 35 | ((integrative adj3 (review* or overview*)) or (collaborative adj3 (review* or overview*)) or (pool* adj3 analy*)).ti,ab,kf,kw. | 29667 |
| 36 | (data synthes* or data extraction* or data abstraction*).ti,ab,kf,kw. | 30033 |
| 37 | (handsearch* or hand search*).ti,ab,kf,kw. | 9705 |
| 38 | (mantel haenszel or peto or der simonian or dersimonian or fixed effect* or latin square*).ti,ab,kf,kw. | 28453 |
| 39 | (met analy* or metanaly* or technology assessment* or HTA or HTAs or technology overview* or technology appraisal*).ti,ab,kf,kw. | 9963 |
| 40 | (meta regression* or metaregression*).ti,ab,kf,kw. | 10163 |
| 41 | (meta-analy* or metaanaly* or systematic review* or biomedical technology assessment* or bio-medical technology assessment*).mp,hw. | 342728 |
| 42 | (medline or cochrane or pubmed or medlars or embase or cinahl).ti,ab,hw. | 248140 |
| 43 | (cochrane or (health adj2 technology assessment) or evidence report).jw. | 19999 |
| 44 | (comparative adj3 (efficacy or effectiveness)).ti,ab,kf,kw. | 14425 |
| 45 | (outcomes research or relative effectiveness).ti,ab,kf,kw. | 9841 |
| 46 | ((indirect or indirect treatment or mixed-treatment) adj comparison*).ti,ab,kf,kw. | 2302 |
| 47 | 31 or 32 or 33 or 34 or 35 or 36 or 37 or 38 or 39 or 40 or 41 or 42 or 43 or 44 or 45 or 46 | 516705 |
| 48 | Case-Control Studies/ or Control Groups/ or Matched-Pair Analysis/ or ((case* adj5 control*) or (case adj3 comparison*) or control group*).ti,ab,kw. | 823084 |
| 49 | cohort studies/ or longitudinal studies/ or follow-up studies/ or prospective studies/ or retrospective studies/ or cohort.ti,ab. or longitudinal.ti,ab. or prospective.ti,ab. or retrospective.ti,ab. | 2795784 |
| 50 | (autobiography or bibliography or biography or case reports or classical article or letter or patient education handout or dictionary or directory or editorial or historical article or interactive tutorial or interview or introductory journal article or lecture or legal case or legislation or news or newspaper article or personal narrative or portrait or video audio media or webcast).pt. | 4310851 |
| 51 | exp animals/ not humans.sh. | 4799766 |
| 52 | (exp infant/ or exp child/ or adolescent/) not exp adult/ | 1918444 |
| 53 | 50 or 51 or 52 | 10405865 |
| 54 | ((incisional or postoperative or post-operative or ventral) adj3 hernia?).ab,kf,kw,ti. | 8407 |
| 55 | hernia, ventral/ or incisional hernia/ | 7067 |
| 56 | 54 or 55 | 11266 |
| 57 | ((medical or surgical or surgery) adj4 clearance?).ab,kf,kw,ti. | 1148 |
| 58 | ((presurgery or pre-surgery or "pre surgery" or pre-surgical or presurgical or "pre surgical" or Preoperative* or Pre-operative* or "Pre operative*") adj4 (evaluat* or assess* or plan* or characteri* or clearance? or investigat* or information)).ab,kf,kw,ti. | 65652 |
| 59 | 57 or 58 | 66722 |
| 60 | 56 and 59 | 146 |
| 61 | 60 not 53 | 132 |
| 62 | limit 61 to english language | 125 |
| 63 | 30 and 62 | 14 |
| 64 | 47 and 62 | 3 |
| 65 | 48 and 62 | 7 |
| 66 | 49 and 62 | 81 |

**Embase <1974 to 2021 March 12> (Ovid)**

**Search was conducted on 14^th^ March 2021.**

| # | search string | # of results |
| --- | --- | --- |
| 1 | (Randomized Controlled Trial or Controlled Clinical Trial or Pragmatic Clinical Trial or Equivalence Trial or Clinical Trial, Phase III).pt. | 0 |
| 2 | Randomized Controlled Trial/ | 651778 |
| 3 | exp Randomized Controlled Trials as Topic/ | 198855 |
| 4 | "Randomized Controlled Trial (topic)"/ | 198855 |
| 5 | Controlled Clinical Trial/ | 466838 |
| 6 | exp Controlled Clinical Trials as Topic/ | 206807 |
| 7 | "Controlled Clinical Trial (topic)"/ | 11531 |
| 8 | Randomization/ | 90619 |
| 9 | Random Allocation/ | 86809 |
| 10 | Double-Blind Method/ | 158430 |
| 11 | Double Blind Procedure/ | 182771 |
| 12 | Double-Blind Studies/ | 142095 |
| 13 | Single-Blind Method/ | 40318 |
| 14 | Single Blind Procedure/ | 42335 |
| 15 | Single-Blind Studies/ | 42335 |
| 16 | Placebos/ | 309399 |
| 17 | Placebo/ | 365195 |
| 18 | Control Groups/ | 110539 |
| 19 | Control Group/ | 110539 |
| 20 | (random* or sham or placebo*).ti,ab,hw,kw. | 2172603 |
| 21 | ((singl* or doubl*) adj (blind* or dumm* or mask*)).ti,ab,hw,kw. | 320093 |
| 22 | ((tripl* or trebl*) adj (blind* or dumm* or mask*)).ti,ab,hw,kw. | 1517 |
| 23 | (control* adj3 (study or studies or trial* or group*)).ti,ab,kw. | 1457801 |
| 24 | (Nonrandom* or non random* or non-random* or quasi-random* or quasirandom*).ti,ab,hw,kw. | 58796 |
| 25 | allocated.ti,ab,hw. | 90951 |
| 26 | ((open label or open-label) adj5 (study or studies or trial*)).ti,ab,hw,kw. | 68645 |
| 27 | ((equivalence or superiority or non-inferiority or noninferiority) adj3 (study or studies or trial*)).ti,ab,hw,kw. | 13672 |
| 28 | (pragmatic study or pragmatic studies).ti,ab,hw,kw. | 674 |
| 29 | ((pragmatic or practical) adj3 trial*).ti,ab,hw,kw. | 6189 |
| 30 | ((quasiexperimental or quasi-experimental) adj3 (study or studies or trial*)).ti,ab,hw,kw. | 14063 |
| 31 | (phase adj3 (III or "3") adj3 (study or studies or trial*)).ti,hw,kw. | 98966 |
| 32 | 1 or 2 or 3 or 4 or 5 or 6 or 7 or 8 or 9 or 10 or 11 or 12 or 13 or 14 or 15 or 16 or 17 or 18 or 19 or 20 or 21 or 22 or 23 or 24 or 25 or 26 or 27 or 28 or 29 or 30 or 31 | 3232963 |
| 33 | meta-analysis.pt. | 0 |
| 34 | meta-analysis/ or systematic review/ or meta-analysis as topic/ or "meta analysis (topic)"/ or "systematic review (topic)"/ or exp technology assessment, biomedical/ | 455174 |
| 35 | ((systematic* adj3 (review* or overview*)) or (methodologic* adj3 (review* or overview*))).ti,ab,kw. | 274697 |
| 36 | ((quantitative adj3 (review* or overview* or synthes*)) or (research adj3 (integrati* or overview*))).ti,ab,kw. | 14248 |
| 37 | ((integrative adj3 (review* or overview*)) or (collaborative adj3 (review* or overview*)) or (pool* adj3 analy*)).ti,ab,kw. | 42507 |
| 38 | (data synthes* or data extraction* or data abstraction*).ti,ab,kw. | 37317 |
| 39 | (handsearch* or hand search*).ti,ab,kw. | 11847 |
| 40 | (mantel haenszel or peto or der simonian or dersimonian or fixed effect* or latin square*).ti,ab,kw. | 37755 |
| 41 | (met analy* or metanaly* or technology assessment* or HTA or HTAs or technology overview* or technology appraisal*).ti,ab,kw. | 16298 |
| 42 | (meta regression* or metaregression*).ti,ab,kw. | 12758 |
| 43 | (meta-analy* or metaanaly* or systematic review* or biomedical technology assessment* or bio-medical technology assessment*).mp,hw. | 547838 |
| 44 | (medline or cochrane or pubmed or medlars or embase or cinahl).ti,ab,hw. | 328261 |
| 45 | (cochrane or (health adj2 technology assessment) or evidence report).jx. | 27593 |
| 46 | (comparative adj3 (efficacy or effectiveness)).ti,ab,kw. | 21142 |
| 47 | (outcomes research or relative effectiveness).ti,ab,kw. | 14232 |
| 48 | ((indirect or indirect treatment or mixed-treatment) adj comparison*).ti,ab,kw. | 4384 |
| 49 | 33 or 34 or 35 or 36 or 37 or 38 or 39 or 40 or 41 or 42 or 43 or 44 or 45 or 46 or 47 or 48 | 756660 |
| 50 | exp case control study/ or control group/ or statistical analysis/ or ((case* adj5 control*) or (case adj3 comparison*) or control group*).ti,kw,ab. | 1227352 |
| 51 | cohort analysis/ or longitudinal study/ or follow up/ or prospective study/ or retrospective study/ or cohort.ti,ab. or longitudinal.ti,ab. or prospective.ti,ab. or retrospective.ti,ab. | 4290705 |
| 52 | (conference abstract or "conference review" or editorial or letter or note or short survey).pt. | 7133520 |
| 53 | (exp animal/ or animal experiment/ or nonhuman/) not (exp human/ or human experiment/) | 6666363 |
| 54 | exp juvenile/ not exp adult/ | 2278466 |
| 55 | 52 or 53 or 54 | 14870312 |
| 56 | ((incisional or postoperative or post-operative or ventral) adj3 hernia?).ab,kw,ti. | 12011 |
| 57 | abdominal wall hernia/ or incisional hernia/ | 15742 |
| 58 | 56 or 57 | 18639 |
| 59 | (ERAS or enhanced recover* or Enhanced Postsurgical Recover* or Enhanced Post-surgical Recover* or Enhanced Post surgical Recover* or Enhanced Postoperative Recover* or Enhanced Post-operative Recover* or Enhanced Post operative Recover*).ab,kw,ti. | 12946 |
| 60 | enhanced recovery after surgery/ | 1533 |
| 61 | 59 or 60 | 13153 |
| 62 | 58 and 61 | 84 |
| 63 | 62 not 55 | 53 |
| 64 | limit 63 to english language | 53 |
| 65 | 32 and 64 | 18 |
| 66 | 49 and 64 | 6 |
| 67 | 50 and 64 | 8 |
| 68 | 51 and 64 | 33 |

**Embase <1974 to 2021 March 12> (Ovid)**

**Search was conducted on 14^th^ March 2021.**

| # | search string | # of results |
| --- | --- | --- |
| 1 | (Randomized Controlled Trial or Controlled Clinical Trial or Pragmatic Clinical Trial or Equivalence Trial or Clinical Trial, Phase III).pt. | 0 |
| 2 | Randomized Controlled Trial/ | 651778 |
| 3 | exp Randomized Controlled Trials as Topic/ | 198855 |
| 4 | "Randomized Controlled Trial (topic)"/ | 198855 |
| 5 | Controlled Clinical Trial/ | 466838 |
| 6 | exp Controlled Clinical Trials as Topic/ | 206807 |
| 7 | "Controlled Clinical Trial (topic)"/ | 11531 |
| 8 | Randomization/ | 90619 |
| 9 | Random Allocation/ | 86809 |
| 10 | Double-Blind Method/ | 158430 |
| 11 | Double Blind Procedure/ | 182771 |
| 12 | Double-Blind Studies/ | 142095 |
| 13 | Single-Blind Method/ | 40318 |
| 14 | Single Blind Procedure/ | 42335 |
| 15 | Single-Blind Studies/ | 42335 |
| 16 | Placebos/ | 309399 |
| 17 | Placebo/ | 365195 |
| 18 | Control Groups/ | 110539 |
| 19 | Control Group/ | 110539 |
| 20 | (random* or sham or placebo*).ti,ab,hw,kw. | 2172603 |
| 21 | ((singl* or doubl*) adj (blind* or dumm* or mask*)).ti,ab,hw,kw. | 320093 |
| 22 | ((tripl* or trebl*) adj (blind* or dumm* or mask*)).ti,ab,hw,kw. | 1517 |
| 23 | (control* adj3 (study or studies or trial* or group*)).ti,ab,kw. | 1457801 |
| 24 | (Nonrandom* or non random* or non-random* or quasi-random* or quasirandom*).ti,ab,hw,kw. | 58796 |
| 25 | allocated.ti,ab,hw. | 90951 |
| 26 | ((open label or open-label) adj5 (study or studies or trial*)).ti,ab,hw,kw. | 68645 |
| 27 | ((equivalence or superiority or non-inferiority or noninferiority) adj3 (study or studies or trial*)).ti,ab,hw,kw. | 13672 |
| 28 | (pragmatic study or pragmatic studies).ti,ab,hw,kw. | 674 |
| 29 | ((pragmatic or practical) adj3 trial*).ti,ab,hw,kw. | 6189 |
| 30 | ((quasiexperimental or quasi-experimental) adj3 (study or studies or trial*)).ti,ab,hw,kw. | 14063 |
| 31 | (phase adj3 (III or "3") adj3 (study or studies or trial*)).ti,hw,kw. | 98966 |
| 32 | 1 or 2 or 3 or 4 or 5 or 6 or 7 or 8 or 9 or 10 or 11 or 12 or 13 or 14 or 15 or 16 or 17 or 18 or 19 or 20 or 21 or 22 or 23 or 24 or 25 or 26 or 27 or 28 or 29 or 30 or 31 | 3232963 |
| 33 | meta-analysis.pt. | 0 |
| 34 | meta-analysis/ or systematic review/ or meta-analysis as topic/ or "meta analysis (topic)"/ or "systematic review (topic)"/ or exp technology assessment, biomedical/ | 455174 |
| 35 | ((systematic* adj3 (review* or overview*)) or (methodologic* adj3 (review* or overview*))).ti,ab,kw. | 274697 |
| 36 | ((quantitative adj3 (review* or overview* or synthes*)) or (research adj3 (integrati* or overview*))).ti,ab,kw. | 14248 |
| 37 | ((integrative adj3 (review* or overview*)) or (collaborative adj3 (review* or overview*)) or (pool* adj3 analy*)).ti,ab,kw. | 42507 |
| 38 | (data synthes* or data extraction* or data abstraction*).ti,ab,kw. | 37317 |
| 39 | (handsearch* or hand search*).ti,ab,kw. | 11847 |
| 40 | (mantel haenszel or peto or der simonian or dersimonian or fixed effect* or latin square*).ti,ab,kw. | 37755 |
| 41 | (met analy* or metanaly* or technology assessment* or HTA or HTAs or technology overview* or technology appraisal*).ti,ab,kw. | 16298 |
| 42 | (meta regression* or metaregression*).ti,ab,kw. | 12758 |
| 43 | (meta-analy* or metaanaly* or systematic review* or biomedical technology assessment* or bio-medical technology assessment*).mp,hw. | 547838 |
| 44 | (medline or cochrane or pubmed or medlars or embase or cinahl).ti,ab,hw. | 328261 |
| 45 | (cochrane or (health adj2 technology assessment) or evidence report).jx. | 27593 |
| 46 | (comparative adj3 (efficacy or effectiveness)).ti,ab,kw. | 21142 |
| 47 | (outcomes research or relative effectiveness).ti,ab,kw. | 14232 |
| 48 | ((indirect or indirect treatment or mixed-treatment) adj comparison*).ti,ab,kw. | 4384 |
| 49 | 33 or 34 or 35 or 36 or 37 or 38 or 39 or 40 or 41 or 42 or 43 or 44 or 45 or 46 or 47 or 48 | 756660 |
| 50 | exp case control study/ or control group/ or statistical analysis/ or ((case* adj5 control*) or (case adj3 comparison*) or control group*).ti,ab,kw. | 1227352 |
| 51 | cohort analysis/ or longitudinal study/ or follow up/ or prospective study/ or retrospective study/ or cohort.ti,ab. or longitudinal.ti,ab. or prospective.ti,ab. or retrospective.ti,ab. | 4290705 |
| 52 | (conference abstract or "conference review" or editorial or letter or note or short survey).pt. | 7133520 |
| 53 | (exp animal/ or animal experiment/ or nonhuman/) not (exp human/ or human experiment/) | 6666363 |
| 54 | exp juvenile/ not exp adult/ | 2278466 |
| 55 | 52 or 53 or 54 | 14870312 |
| 56 | ((incisional or postoperative or post-operative or ventral) adj3 hernia?).ab,kw,ti. | 12011 |
| 57 | abdominal wall hernia/ or incisional hernia/ | 15742 |
| 58 | 56 or 57 | 18639 |
| 59 | ((medical or surgical or surgery) adj4 clearance?).ab,kw,ti. | 1837 |
| 60 | ((presurgery or pre-surgery or "pre surgery" or pre-surgical or presurgical or "pre surgical" or Preoperative* or Pre-operative* or "Pre operative*") adj4 (evaluat* or assess* or plan* or characteri* or clearance? or investigat* or information)).ab,kw,ti. | 95498 |
| 61 | preoperative evaluation/ | 135926 |
| 62 | 59 or 60 or 61 | 199599 |
| 63 | 58 and 62 | 673 |
| 64 | 63 not 55 | 496 |
| 65 | limit 64 to english language | 468 |
| 66 | 32 and 65 | 59 |
| 67 | 49 and 65 | 11 |
| 68 | 50 and 65 | 19 |
| 69 | 51 and 65 | 295 |

**Cochrane Library**

[**Cochrane Database of Systematic Reviews**](https://www.cochranelibrary.com/)**, Issue 3 of 12, March 2021**

**Cochrane Central Register of Controlled Trials, Issue 3 of 12, March 2021**

**Search was conducted on 18^th^ March 2021.**

| **#** | **search string** | **# of results** |
| --- | --- | --- |
| **1** | ((incisional or postoperative or post-operative or ventral) NEAR/3 hernia?):ti,ab,kw | 1196 |
| **2** | MeSH descriptor: [Hernia, Ventral] this term only | 268 |
| **3** | MeSH descriptor: [Incisional Hernia] this term only | 127 |
| **4** | #1 or #2 or #3 | 1196 |
| **5** | (ERAS or (enhanced NEXT recover*) or (Enhanced NEXT Postsurgical NEXT Recover*) or (Enhanced NEXT Post-surgical NEXT Recover*) or (Enhanced NEXT Post NEXT surgical NEXT Recover*) or (Enhanced NEXT Postoperative NEXT Recover*) or (Enhanced NEXT Post-operative NEXT Recover*) or (Enhanced NEXT Post NEXT operative NEXT Recover*)):ti,ab,kw | 1421 |
| **6** | MeSH descriptor: [Enhanced Recovery After Surgery] this term only | 53 |
| **7** | #5 or #6 | 1421 |
| **8** | #4 and #7 | 11 |
| **9** | #8 AND Cochrane Database of Systematic Reviews Publication | 0 |
| **10** | #8 AND Cochrane Central Register of Controlled Trials Date | 11 |

**Cochrane Library**

[**Cochrane Database of Systematic Reviews**](https://www.cochranelibrary.com/)**, Issue 3 of 12, March 2021**

**Cochrane Central Register of Controlled Trials, Issue 3 of 12, March 2021**

**Search was conducted on 18^th^ March 2021.**

| **#** | **search string** | **# of results** |
| --- | --- | --- |
| **1** | ((incisional or postoperative or post-operative or ventral) NEAR/3 hernia?):ti,ab,kw | 1196 |
| **2** | MeSH descriptor: [Hernia, Ventral] this term only | 268 |
| **3** | MeSH descriptor: [Incisional Hernia] this term only | 127 |
| **4** | #1 or #2 or #3 | 1196 |
| **5** | ((medical or surgical or surgery) NEAR/4 clearance?):ti,ab,kw | 361 |
| **6** | ((presurgery or pre-surgery or "pre surgery" or pre-surgical or presurgical or "pre surgical" or Preoperative* or Pre-operative* or (Pre NEXT operative*)) NEAR/4 (evaluat* or assess* or plan* or characteri* or clearance? or investigat* or information)):ti,ab,kw | 9781 |
| **7** | #5 or #6 | 10125 |
| **8** | #4 and #7 | 37 |
| **9** | #8 AND Cochrane Database of Systematic Reviews Publication | 0 |
| **10** | #8 AND Cochrane Central Register of Controlled Trials Date | 37 |

**KQ 12: Should prophylactic antibiotics be used in the elective repair of incisional hernia in adult patients?**

**MEDLINE(R) ALL <1946 to March 15, 2021> (Ovid)**

**Search was conducted on 16^th^ March 2021.**

| # | search string | # of results |
| --- | --- | --- |
| 1 | (Randomized Controlled Trial or Controlled Clinical Trial or Pragmatic Clinical Trial or Equivalence Trial or Clinical Trial, Phase III).pt. | 618044 |
| 2 | Randomized Controlled Trial/ | 525030 |
| 3 | exp Randomized Controlled Trials as Topic/ | 144810 |
| 4 | Controlled Clinical Trial/ | 94095 |
| 5 | exp Controlled Clinical Trials as Topic/ | 150296 |
| 6 | Randomization/ | 104842 |
| 7 | Random Allocation/ | 104842 |
| 8 | Double-Blind Method/ | 162935 |
| 9 | Double Blind Procedure/ | 0 |
| 10 | Double-Blind Studies/ | 162935 |
| 11 | Single-Blind Method/ | 29867 |
| 12 | Single Blind Procedure/ | 0 |
| 13 | Single-Blind Studies/ | 29867 |
| 14 | Placebos/ | 35377 |
| 15 | Placebo/ | 0 |
| 16 | Control Groups/ | 1725 |
| 17 | Control Group/ | 1725 |
| 18 | (random* or sham or placebo*).ti,ab,hw,kf,kw. | 1572351 |
| 19 | ((singl* or doubl*) adj (blind* or dumm* or mask*)).ti,ab,hw,kf,kw. | 244459 |
| 20 | ((tripl* or trebl*) adj (blind* or dumm* or mask*)).ti,ab,hw,kf,kw. | 1144 |
| 21 | (control* adj3 (study or studies or trial* or group*)).ti,ab,kf,kw. | 1039014 |
| 22 | (Nonrandom* or non random* or non-random* or quasi-random* or quasirandom*).ti,ab,hw,kf,kw. | 46532 |
| 23 | allocated.ti,ab,hw. | 70017 |
| 24 | ((open label or open-label) adj5 (study or studies or trial*)).ti,ab,hw,kf,kw. | 36955 |
| 25 | ((equivalence or superiority or non-inferiority or noninferiority) adj3 (study or studies or trial*)).ti,ab,hw,kf,kw. | 9227 |
| 26 | (pragmatic study or pragmatic studies).ti,ab,hw,kf,kw. | 451 |
| 27 | ((pragmatic or practical) adj3 trial*).ti,ab,hw,kf,kw. | 5818 |
| 28 | ((quasiexperimental or quasi-experimental) adj3 (study or studies or trial*)).ti,ab,hw,kf,kw. | 8739 |
| 29 | (phase adj3 (III or "3") adj3 (study or studies or trial*)).ti,hw,kf,kw. | 30001 |
| 30 | 1 or 2 or 3 or 4 or 5 or 6 or 7 or 8 or 9 or 10 or 11 or 12 or 13 or 14 or 15 or 16 or 17 or 18 or 19 or 20 or 21 or 22 or 23 or 24 or 25 or 26 or 27 or 28 or 29 | 2258738 |
| 31 | meta-analysis.pt. | 128007 |
| 32 | meta-analysis/ or systematic review/ or meta-analysis as topic/ or "meta analysis (topic)"/ or "systematic review (topic)"/ or exp technology assessment, biomedical/ | 241293 |
| 33 | ((systematic* adj3 (review* or overview*)) or (methodologic* adj3 (review* or overview*))).ti,ab,kf,kw. | 219099 |
| 34 | ((quantitative adj3 (review* or overview* or synthes*)) or (research adj3 (integrati* or overview*))).ti,ab,kf,kw. | 11957 |
| 35 | ((integrative adj3 (review* or overview*)) or (collaborative adj3 (review* or overview*)) or (pool* adj3 analy*)).ti,ab,kf,kw. | 29667 |
| 36 | (data synthes* or data extraction* or data abstraction*).ti,ab,kf,kw. | 30033 |
| 37 | (handsearch* or hand search*).ti,ab,kf,kw. | 9705 |
| 38 | (mantel haenszel or peto or der simonian or dersimonian or fixed effect* or latin square*).ti,ab,kf,kw. | 28453 |
| 39 | (met analy* or metanaly* or technology assessment* or HTA or HTAs or technology overview* or technology appraisal*).ti,ab,kf,kw. | 9963 |
| 40 | (meta regression* or metaregression*).ti,ab,kf,kw. | 10163 |
| 41 | (meta-analy* or metaanaly* or systematic review* or biomedical technology assessment* or bio-medical technology assessment*).mp,hw. | 342728 |
| 42 | (medline or cochrane or pubmed or medlars or embase or cinahl).ti,ab,hw. | 248140 |
| 43 | (cochrane or (health adj2 technology assessment) or evidence report).jw. | 19999 |
| 44 | (comparative adj3 (efficacy or effectiveness)).ti,ab,kf,kw. | 14425 |
| 45 | (outcomes research or relative effectiveness).ti,ab,kf,kw. | 9841 |
| 46 | ((indirect or indirect treatment or mixed-treatment) adj comparison*).ti,ab,kf,kw. | 2302 |
| 47 | 31 or 32 or 33 or 34 or 35 or 36 or 37 or 38 or 39 or 40 or 41 or 42 or 43 or 44 or 45 or 46 | 516705 |
| 48 | Case-Control Studies/ or Control Groups/ or Matched-Pair Analysis/ or ((case* adj5 control*) or (case adj3 comparison*) or control group*).ti,ab,kw. | 823084 |
| 49 | cohort studies/ or longitudinal studies/ or follow-up studies/ or prospective studies/ or retrospective studies/ or cohort.ti,ab. or longitudinal.ti,ab. or prospective.ti,ab. or retrospective.ti,ab. | 2795784 |
| 50 | (autobiography or bibliography or biography or case reports or classical article or letter or patient education handout or dictionary or directory or editorial or historical article or interactive tutorial or interview or introductory journal article or lecture or legal case or legislation or news or newspaper article or personal narrative or portrait or video audio media or webcast).pt. | 4310851 |
| 51 | exp animals/ not humans.sh. | 4799766 |
| 52 | (exp infant/ or exp child/ or adolescent/) not exp adult/ | 1918444 |
| 53 | 50 or 51 or 52 | 10405865 |
| 54 | ((incisional or postoperative or post-operative or ventral) adj3 hernia?).ab,kf,kw,ti. | 8407 |
| 55 | hernia, ventral/ or incisional hernia/ | 7067 |
| 56 | 54 or 55 | 11266 |
| 57 | (Anti-Bacterial or Anti Bacterial or Antibacterial or Bacteriocidal or Bacteriocide? or Anti-Mycobacterial or Anti Mycobacterial or Antimycobacterial or antibiotic?).ab,kf,kw,ti. | 432999 |
| 58 | Antibiotic Prophylaxis/ | 14296 |
| 59 | 57 or 58 | 439307 |
| 60 | 56 and 59 | 235 |
| 61 | 60 not 53 | 176 |
| 62 | limit 61 to english language | 153 |
| 63 | 30 and 62 | 28 |
| 64 | 47 and 62 | 8 |
| 65 | 48 and 62 | 9 |
| 66 | 49 and 62 | 97 |

**Embase <1974 to 2021 March 12> (Ovid)**

**Search was conducted on 14^th^ March 2021.**

| # | search string | # of results |
| --- | --- | --- |
| 1 | (Randomized Controlled Trial or Controlled Clinical Trial or Pragmatic Clinical Trial or Equivalence Trial or Clinical Trial, Phase III).pt. | 0 |
| 2 | Randomized Controlled Trial/ | 651778 |
| 3 | exp Randomized Controlled Trials as Topic/ | 198855 |
| 4 | "Randomized Controlled Trial (topic)"/ | 198855 |
| 5 | Controlled Clinical Trial/ | 466838 |
| 6 | exp Controlled Clinical Trials as Topic/ | 206807 |
| 7 | "Controlled Clinical Trial (topic)"/ | 11531 |
| 8 | Randomization/ | 90619 |
| 9 | Random Allocation/ | 86809 |
| 10 | Double-Blind Method/ | 158430 |
| 11 | Double Blind Procedure/ | 182771 |
| 12 | Double-Blind Studies/ | 142095 |
| 13 | Single-Blind Method/ | 40318 |
| 14 | Single Blind Procedure/ | 42335 |
| 15 | Single-Blind Studies/ | 42335 |
| 16 | Placebos/ | 309399 |
| 17 | Placebo/ | 365195 |
| 18 | Control Groups/ | 110539 |
| 19 | Control Group/ | 110539 |
| 20 | (random* or sham or placebo*).ti,ab,hw,kw. | 2172603 |
| 21 | ((singl* or doubl*) adj (blind* or dumm* or mask*)).ti,ab,hw,kw. | 320093 |
| 22 | ((tripl* or trebl*) adj (blind* or dumm* or mask*)).ti,ab,hw,kw. | 1517 |
| 23 | (control* adj3 (study or studies or trial* or group*)).ti,ab,kw. | 1457801 |
| 24 | (Nonrandom* or non random* or non-random* or quasi-random* or quasirandom*).ti,ab,hw,kw. | 58796 |
| 25 | allocated.ti,ab,hw. | 90951 |
| 26 | ((open label or open-label) adj5 (study or studies or trial*)).ti,ab,hw,kw. | 68645 |
| 27 | ((equivalence or superiority or non-inferiority or noninferiority) adj3 (study or studies or trial*)).ti,ab,hw,kw. | 13672 |
| 28 | (pragmatic study or pragmatic studies).ti,ab,hw,kw. | 674 |
| 29 | ((pragmatic or practical) adj3 trial*).ti,ab,hw,kw. | 6189 |
| 30 | ((quasiexperimental or quasi-experimental) adj3 (study or studies or trial*)).ti,ab,hw,kw. | 14063 |
| 31 | (phase adj3 (III or "3") adj3 (study or studies or trial*)).ti,hw,kw. | 98966 |
| 32 | 1 or 2 or 3 or 4 or 5 or 6 or 7 or 8 or 9 or 10 or 11 or 12 or 13 or 14 or 15 or 16 or 17 or 18 or 19 or 20 or 21 or 22 or 23 or 24 or 25 or 26 or 27 or 28 or 29 or 30 or 31 | 3232963 |
| 33 | meta-analysis.pt. | 0 |
| 34 | meta-analysis/ or systematic review/ or meta-analysis as topic/ or "meta analysis (topic)"/ or "systematic review (topic)"/ or exp technology assessment, biomedical/ | 455174 |
| 35 | ((systematic* adj3 (review* or overview*)) or (methodologic* adj3 (review* or overview*))).ti,ab,kw. | 274697 |
| 36 | ((quantitative adj3 (review* or overview* or synthes*)) or (research adj3 (integrati* or overview*))).ti,ab,kw. | 14248 |
| 37 | ((integrative adj3 (review* or overview*)) or (collaborative adj3 (review* or overview*)) or (pool* adj3 analy*)).ti,ab,kw. | 42507 |
| 38 | (data synthes* or data extraction* or data abstraction*).ti,ab,kw. | 37317 |
| 39 | (handsearch* or hand search*).ti,ab,kw. | 11847 |
| 40 | (mantel haenszel or peto or der simonian or dersimonian or fixed effect* or latin square*).ti,ab,kw. | 37755 |
| 41 | (met analy* or metanaly* or technology assessment* or HTA or HTAs or technology overview* or technology appraisal*).ti,ab,kw. | 16298 |
| 42 | (meta regression* or metaregression*).ti,ab,kw. | 12758 |
| 43 | (meta-analy* or metaanaly* or systematic review* or biomedical technology assessment* or bio-medical technology assessment*).mp,hw. | 547838 |
| 44 | (medline or cochrane or pubmed or medlars or embase or cinahl).ti,ab,hw. | 328261 |
| 45 | (cochrane or (health adj2 technology assessment) or evidence report).jx. | 27593 |
| 46 | (comparative adj3 (efficacy or effectiveness)).ti,ab,kw. | 21142 |
| 47 | (outcomes research or relative effectiveness).ti,ab,kw. | 14232 |
| 48 | ((indirect or indirect treatment or mixed-treatment) adj comparison*).ti,ab,kw. | 4384 |
| 49 | 33 or 34 or 35 or 36 or 37 or 38 or 39 or 40 or 41 or 42 or 43 or 44 or 45 or 46 or 47 or 48 | 756660 |
| 50 | exp case control study/ or control group/ or statistical analysis/ or ((case* adj5 control*) or (case adj3 comparison*) or control group*).ti,kw,ab. | 1227352 |
| 51 | cohort analysis/ or longitudinal study/ or follow up/ or prospective study/ or retrospective study/ or cohort.ti,ab. or longitudinal.ti,ab. or prospective.ti,ab. or retrospective.ti,ab. | 4290705 |
| 52 | (conference abstract or "conference review" or editorial or letter or note or short survey).pt. | 7133520 |
| 53 | (exp animal/ or animal experiment/ or nonhuman/) not (exp human/ or human experiment/) | 6666363 |
| 54 | exp juvenile/ not exp adult/ ( | 2278466 |
| 55 | 52 or 53 or 54 | 14870312 |
| 56 | ((incisional or postoperative or post-operative or ventral) adj3 hernia?).ab,kw,ti. | 12011 |
| 57 | abdominal wall hernia/ or incisional hernia/ | 15742 |
| 58 | 56 or 57 | 18639 |
| 59 | (Anti-Bacterial or Anti Bacterial or Antibacterial or Bacteriocidal or Bacteriocide? or Anti-Mycobacterial or Anti Mycobacterial or Antimycobacterial or antibiotic?).ab,kw,ti. | 559369 |
| 60 | antibiotic prophylaxis/ | 32963 |
| 61 | 59 or 60 | 575557 |
| 62 | 58 and 61 | 543 |
| 63 | 62 not 55 | 333 |
| 64 | limit 63 to english language | 299 |
| 65 | 32 and 64 | 45 |
| 66 | 49 and 64 | 21 |
| 67 | 50 and 64 | 10 |
| 68 | 51 and 64 | 155 |

**Cochrane Library**

[**Cochrane Database of Systematic Reviews**](https://www.cochranelibrary.com/)**, Issue 3 of 12, March 2021**

**Cochrane Central Register of Controlled Trials, Issue 3 of 12, March 2021**

**Search was conducted on 18^th^ March 2021.**

| **#** | **search string** | **# of results** |
| --- | --- | --- |
| **1** | ((incisional or postoperative or post-operative or ventral) NEAR/3 hernia?):ti,ab,kw | 1196 |
| **2** | MeSH descriptor: [Hernia, Ventral] this term only | 268 |
| **3** | MeSH descriptor: [Incisional Hernia] this term only | 127 |
| **4** | #1 or #2 or #3 | 1196 |
| **5** | (Anti-Bacterial or "Anti Bacterial" or Antibacterial or Bacteriocidal or Bacteriocide? or Anti-Mycobacterial or "Anti Mycobacterial" or Antimycobacterial or antibiotic?):ti,ab,kw | 38278 |
| **6** | MeSH descriptor: [Antibiotic Prophylaxis] this term only | 1293 |
| **7** | #5 or #6 | 38278 |
| **8** | #4 and #7 | 58 |
| **9** | #8 AND Cochrane Database of Systematic Reviews Publication | 0 |
| **10** | #8 AND Cochrane Central Register of Controlled Trials Date | 58 |

**KQ 13: What information is important for patients following incisional hernia repair? What activities influence outcome?**

**MEDLINE(R) ALL <1946 to March 15, 2021> (Ovid)**

**Search was conducted on 16^th^ March 2021.**

| # | search string | # of results |
| --- | --- | --- |
| 1 | (Randomized Controlled Trial or Controlled Clinical Trial or Pragmatic Clinical Trial or Equivalence Trial or Clinical Trial, Phase III).pt. | 618044 |
| 2 | Randomized Controlled Trial/ | 525030 |
| 3 | exp Randomized Controlled Trials as Topic/ | 144810 |
| 4 | Controlled Clinical Trial/ | 94095 |
| 5 | exp Controlled Clinical Trials as Topic/ | 150296 |
| 6 | Randomization/ | 104842 |
| 7 | Random Allocation/ | 104842 |
| 8 | Double-Blind Method/ | 162935 |
| 9 | Double Blind Procedure/ | 0 |
| 10 | Double-Blind Studies/ | 162935 |
| 11 | Single-Blind Method/ | 29867 |
| 12 | Single Blind Procedure/ | 0 |
| 13 | Single-Blind Studies/ | 29867 |
| 14 | Placebos/ | 35377 |
| 15 | Placebo/ | 0 |
| 16 | Control Groups/ | 1725 |
| 17 | Control Group/ | 1725 |
| 18 | (random* or sham or placebo*).ti,ab,hw,kf,kw. | 1572351 |
| 19 | ((singl* or doubl*) adj (blind* or dumm* or mask*)).ti,ab,hw,kf,kw. | 244459 |
| 20 | ((tripl* or trebl*) adj (blind* or dumm* or mask*)).ti,ab,hw,kf,kw. | 1144 |
| 21 | (control* adj3 (study or studies or trial* or group*)).ti,ab,kf,kw. | 1039014 |
| 22 | (Nonrandom* or non random* or non-random* or quasi-random* or quasirandom*).ti,ab,hw,kf,kw. | 46532 |
| 23 | allocated.ti,ab,hw. | 70017 |
| 24 | ((open label or open-label) adj5 (study or studies or trial*)).ti,ab,hw,kf,kw. | 36955 |
| 25 | ((equivalence or superiority or non-inferiority or noninferiority) adj3 (study or studies or trial*)).ti,ab,hw,kf,kw. | 9227 |
| 26 | (pragmatic study or pragmatic studies).ti,ab,hw,kf,kw. | 451 |
| 27 | ((pragmatic or practical) adj3 trial*).ti,ab,hw,kf,kw. | 5818 |
| 28 | ((quasiexperimental or quasi-experimental) adj3 (study or studies or trial*)).ti,ab,hw,kf,kw. | 8739 |
| 29 | (phase adj3 (III or "3") adj3 (study or studies or trial*)).ti,hw,kf,kw. | 30001 |
| 30 | 1 or 2 or 3 or 4 or 5 or 6 or 7 or 8 or 9 or 10 or 11 or 12 or 13 or 14 or 15 or 16 or 17 or 18 or 19 or 20 or 21 or 22 or 23 or 24 or 25 or 26 or 27 or 28 or 29 | 2258738 |
| 31 | meta-analysis.pt. | 128007 |
| 32 | meta-analysis/ or systematic review/ or meta-analysis as topic/ or "meta analysis (topic)"/ or "systematic review (topic)"/ or exp technology assessment, biomedical/ | 241293 |
| 33 | ((systematic* adj3 (review* or overview*)) or (methodologic* adj3 (review* or overview*))).ti,ab,kf,kw. | 219099 |
| 34 | ((quantitative adj3 (review* or overview* or synthes*)) or (research adj3 (integrati* or overview*))).ti,ab,kf,kw. | 11957 |
| 35 | ((integrative adj3 (review* or overview*)) or (collaborative adj3 (review* or overview*)) or (pool* adj3 analy*)).ti,ab,kf,kw. | 29667 |
| 36 | (data synthes* or data extraction* or data abstraction*).ti,ab,kf,kw. | 30033 |
| 37 | (handsearch* or hand search*).ti,ab,kf,kw. | 9705 |
| 38 | (mantel haenszel or peto or der simonian or dersimonian or fixed effect* or latin square*).ti,ab,kf,kw. | 28453 |
| 39 | (met analy* or metanaly* or technology assessment* or HTA or HTAs or technology overview* or technology appraisal*).ti,ab,kf,kw. | 9963 |
| 40 | (meta regression* or metaregression*).ti,ab,kf,kw. | 10163 |
| 41 | (meta-analy* or metaanaly* or systematic review* or biomedical technology assessment* or bio-medical technology assessment*).mp,hw. | 342728 |
| 42 | (medline or cochrane or pubmed or medlars or embase or cinahl).ti,ab,hw. | 248140 |
| 43 | (cochrane or (health adj2 technology assessment) or evidence report).jw. | 19999 |
| 44 | (comparative adj3 (efficacy or effectiveness)).ti,ab,kf,kw. | 14425 |
| 45 | (outcomes research or relative effectiveness).ti,ab,kf,kw. | 9841 |
| 46 | ((indirect or indirect treatment or mixed-treatment) adj comparison*).ti,ab,kf,kw. | 2302 |
| 47 | 31 or 32 or 33 or 34 or 35 or 36 or 37 or 38 or 39 or 40 or 41 or 42 or 43 or 44 or 45 or 46 | 516705 |
| 48 | Case-Control Studies/ or Control Groups/ or Matched-Pair Analysis/ or ((case* adj5 control*) or (case adj3 comparison*) or control group*).ti,ab,kw. | 823084 |
| 49 | cohort studies/ or longitudinal studies/ or follow-up studies/ or prospective studies/ or retrospective studies/ or cohort.ti,ab. or longitudinal.ti,ab. or prospective.ti,ab. or retrospective.ti,ab. | 2795784 |
| 50 | (autobiography or bibliography or biography or case reports or classical article or letter or patient education handout or dictionary or directory or editorial or historical article or interactive tutorial or interview or introductory journal article or lecture or legal case or legislation or news or newspaper article or personal narrative or portrait or video audio media or webcast).pt. | 4310851 |
| 51 | exp animals/ not humans.sh. | 4799766 |
| 52 | (exp infant/ or exp child/ or adolescent/) not exp adult/ | 1918444 |
| 53 | 50 or 51 or 52 | 10405865 |
| 54 | ((incisional or postoperative or post-operative or ventral) adj3 hernia?).ab,kf,kw,ti. | 8407 |
| 55 | hernia, ventral/ or incisional hernia/ | 7067 |
| 56 | 54 or 55 | 11266 |
| 57 | Postoperative Complications/pc [Prevention & Control] | 47833 |
| 58 | Pain, Postoperative/pc [Prevention & Control] | 12002 |
| 59 | Incisional Hernia/rh [Rehabilitation] | 2 |
| 60 | Hernia, Ventral/rh [Rehabilitation] | 4 |
| 61 | Patient Education as Topic/ | 86488 |
| 62 | (recommendation? or binder? or pressure dressing? or physical rest or rehabilitation? or girdle? or belt? or lifting or weight bearing or weight-bearing or working or work or exercise? or sport or smoking or nicotine or tobacco or activit* or physiotherap* or Physical therap* or laxative? or analgesia).ab,kf,kw,ti. | 5238294 |
| 63 | (postoperative or post-operative or post operative or postsurgical or post-surgical or post surgical or postsurgery or post-surgery or post surgery).ab,kf,kw,ti. | 580966 |
| 64 | ((after or following) adj5 (repair? or surger*)).ab,kf,kw,ti. | 368256 |
| 65 | 63 or 64 | 816446 |
| 66 | 62 and 65 | 117417 |
| 67 | 57 or 58 or 59 or 60 or 61 or 66 | 254288 |
| 68 | 56 and 67 | 996 |
| 69 | 68 not 53 | 850 |
| 70 | limit 69 to english language | 709 |
| 71 | 30 and 70 | 204 |
| 72 | 47 and 71 | 42 |
| 73 | 48 and 70 | 45 |
| 74 | 49 and 70 | 435 |

**Embase <1974 to 2021 March 12> (Ovid)**

**Search was conducted on 14^th^ March 2021.**

| # | search string | # of results |
| --- | --- | --- |
| 1 | (Randomized Controlled Trial or Controlled Clinical Trial or Pragmatic Clinical Trial or Equivalence Trial or Clinical Trial, Phase III).pt. | 0 |
| 2 | Randomized Controlled Trial/ | 651778 |
| 3 | exp Randomized Controlled Trials as Topic/ | 198855 |
| 4 | "Randomized Controlled Trial (topic)"/ | 198855 |
| 5 | Controlled Clinical Trial/ | 466838 |
| 6 | exp Controlled Clinical Trials as Topic/ | 206807 |
| 7 | "Controlled Clinical Trial (topic)"/ | 11531 |
| 8 | Randomization/ | 90619 |
| 9 | Random Allocation/ | 86809 |
| 10 | Double-Blind Method/ | 158430 |
| 11 | Double Blind Procedure/ | 182771 |
| 12 | Double-Blind Studies/ | 142095 |
| 13 | Single-Blind Method/ | 40318 |
| 14 | Single Blind Procedure/ | 42335 |
| 15 | Single-Blind Studies/ | 42335 |
| 16 | Placebos/ | 309399 |
| 17 | Placebo/ | 365195 |
| 18 | Control Groups/ | 110539 |
| 19 | Control Group/ | 110539 |
| 20 | (random* or sham or placebo*).ti,ab,hw,kw. | 2172603 |
| 21 | ((singl* or doubl*) adj (blind* or dumm* or mask*)).ti,ab,hw,kw. | 320093 |
| 22 | ((tripl* or trebl*) adj (blind* or dumm* or mask*)).ti,ab,hw,kw. | 1517 |
| 23 | (control* adj3 (study or studies or trial* or group*)).ti,ab,kw. | 1457801 |
| 24 | (Nonrandom* or non random* or non-random* or quasi-random* or quasirandom*).ti,ab,hw,kw. | 58796 |
| 25 | allocated.ti,ab,hw. | 90951 |
| 26 | ((open label or open-label) adj5 (study or studies or trial*)).ti,ab,hw,kw. | 68645 |
| 27 | ((equivalence or superiority or non-inferiority or noninferiority) adj3 (study or studies or trial*)).ti,ab,hw,kw. | 13672 |
| 28 | (pragmatic study or pragmatic studies).ti,ab,hw,kw. | 674 |
| 29 | ((pragmatic or practical) adj3 trial*).ti,ab,hw,kw. | 6189 |
| 30 | ((quasiexperimental or quasi-experimental) adj3 (study or studies or trial*)).ti,ab,hw,kw. | 14063 |
| 31 | (phase adj3 (III or "3") adj3 (study or studies or trial*)).ti,hw,kw. | 98966 |
| 32 | 1 or 2 or 3 or 4 or 5 or 6 or 7 or 8 or 9 or 10 or 11 or 12 or 13 or 14 or 15 or 16 or 17 or 18 or 19 or 20 or 21 or 22 or 23 or 24 or 25 or 26 or 27 or 28 or 29 or 30 or 31 | 3232963 |
| 33 | meta-analysis.pt. | 0 |
| 34 | meta-analysis/ or systematic review/ or meta-analysis as topic/ or "meta analysis (topic)"/ or "systematic review (topic)"/ or exp technology assessment, biomedical/ | 455174 |
| 35 | ((systematic* adj3 (review* or overview*)) or (methodologic* adj3 (review* or overview*))).ti,ab,kw. | 274697 |
| 36 | ((quantitative adj3 (review* or overview* or synthes*)) or (research adj3 (integrati* or overview*))).ti,ab,kw. | 14248 |
| 37 | ((integrative adj3 (review* or overview*)) or (collaborative adj3 (review* or overview*)) or (pool* adj3 analy*)).ti,ab,kw. | 42507 |
| 38 | (data synthes* or data extraction* or data abstraction*).ti,ab,kw. | 37317 |
| 39 | (handsearch* or hand search*).ti,ab,kw. | 11847 |
| 40 | (mantel haenszel or peto or der simonian or dersimonian or fixed effect* or latin square*).ti,ab,kw. | 37755 |
| 41 | (met analy* or metanaly* or technology assessment* or HTA or HTAs or technology overview* or technology appraisal*).ti,ab,kw. | 16298 |
| 42 | (meta regression* or metaregression*).ti,ab,kw. | 12758 |
| 43 | (meta-analy* or metaanaly* or systematic review* or biomedical technology assessment* or bio-medical technology assessment*).mp,hw. | 547838 |
| 44 | (medline or cochrane or pubmed or medlars or embase or cinahl).ti,ab,hw. | 328261 |
| 45 | (cochrane or (health adj2 technology assessment) or evidence report).jx. | 27593 |
| 46 | (comparative adj3 (efficacy or effectiveness)).ti,ab,kw. | 21142 |
| 47 | (outcomes research or relative effectiveness).ti,ab,kw. | 14232 |
| 48 | ((indirect or indirect treatment or mixed-treatment) adj comparison*).ti,ab,kw. | 4384 |
| 49 | 33 or 34 or 35 or 36 or 37 or 38 or 39 or 40 or 41 or 42 or 43 or 44 or 45 or 46 or 47 or 48 | 756660 |
| 50 | exp case control study/ or control group/ or statistical analysis/ or ((case* adj5 control*) or (case adj3 comparison*) or control group*).ti,kw,ab. | 1227352 |
| 51 | cohort analysis/ or longitudinal study/ or follow up/ or prospective study/ or retrospective study/ or cohort.ti,ab. or longitudinal.ti,ab. or prospective.ti,ab. or retrospective.ti,ab. | 4290705 |
| 52 | (conference abstract or "conference review" or editorial or letter or note or short survey).pt. | 7133520 |
| 53 | (exp animal/ or animal experiment/ or nonhuman/) not (exp human/ or human experiment/) | 6666363 |
| 54 | exp juvenile/ not exp adult/ | 2278466 |
| 55 | 52 or 53 or 54 | 14870312 |
| 56 | ((incisional or postoperative or post-operative or ventral) adj3 hernia?).ab,kw,ti. | 12011 |
| 57 | abdominal wall hernia/ or incisional hernia/ | 15742 |
| 58 | 56 or 57 | 18639 |
| 59 | (recommendation? or binder? or pressure dressing? or physical rest or rehabilitation? or girdle? or belt? or lifting or weight bearing or weight-bearing or working or work or exercise? or sport or smoking or nicotine or tobacco or activit* or physiotherap* or Physical therap* or laxative? or analgesia).ab,kw,ti. | 6601717 |
| 60 | (postoperative or post-operative or post operative or postsurgical or post-surgical or post surgical or postsurgery or post-surgery or post surgery).ab,kw,ti. | 807010 |
| 61 | ((after or following) adj5 (repair? or surger*)).ab,kw,ti. | 513917 |
| 62 | postoperative complication/pc [Prevention] | 17966 |
| 63 | postoperative pain/pc, rh [Prevention, Rehabilitation] | 7684 |
| 64 | incisional hernia/rh [Rehabilitation] | 4 |
| 65 | abdominal wall hernia/rh [Rehabilitation] | 5 |
| 66 | patient education/ | 116218 |
| 67 | 60 or 61 | 1128729 |
| 68 | 59 and 67 | 177652 |
| 69 | 62 or 63 or 64 or 65 or 66 | 141224 |
| 70 | 68 or 69 | 313436 |
| 71 | 58 and 70 | 1446 |
| 72 | 71 not 55 | 934 |
| 73 | limit 72 to english language | 797 |
| 74 | 32 and 73 | 182 |
| 75 | 49 and 73 | 70 |
| 76 | 50 and 73 | 40 |
| 77 | 51 and 73 | 475 |

**Cochrane Library**

[**Cochrane Database of Systematic Reviews**](https://www.cochranelibrary.com/)**, Issue 3 of 12, March 2021**

**Cochrane Central Register of Controlled Trials, Issue 3 of 12, March 2021**

**Search was conducted on 18^th^ March 2021.**

| **#** | **search string** | **# of results** |
| --- | --- | --- |
| **1** | ((incisional or postoperative or post-operative or ventral) NEAR/3 hernia?):ti,ab,kw | 1196 |
| **2** | MeSH descriptor: [Hernia, Ventral] this term only | 268 |
| **3** | MeSH descriptor: [Incisional Hernia] this term only | 127 |
| **4** | #1 or #2 or #3 | 1196 |
| **5** | MeSH descriptor: [Postoperative Complications] this term only and with qualifier(s): [prevention & control - PC] | 6321 |
| **6** | MeSH descriptor: [Pain, Postoperative] this term only and with qualifier(s): [prevention & control - PC] | 5176 |
| **7** | MeSH descriptor: [Incisional Hernia] this term only and with qualifier(s): [rehabilitation - RH] | 0 |
| **8** | MeSH descriptor: [Hernia, Ventral] this term only and with qualifier(s): [rehabilitation - RH] | 0 |
| **9** | MeSH descriptor: [Patient Education as Topic] this term only | 8962 |
| **10** | (recommendation? or binder? or (pressure NEXT dressing?) or "physical rest" or rehabilitation? or girdle? or belt? or lifting or "weight bearing" or weight-bearing or working or work or exercise? or sport or smoking or nicotine or tobacco or activit* or physiotherap* or (Physical NEXT therap*) or laxative? or analgesia):ti,ab,kw | 400237 |
| **11** | (postoperative or post-operative or "post operative" or postsurgical or post-surgical or "post surgical" or postsurgery or post-surgery or "post surgery"):ti,ab,kw | 125485 |
| **12** | ((after or following) NEAR/5 (repair? or surger*)):ti,ab,kw | 65728 |
| **13** | #11 or #12 | 151270 |
| **14** | #10 and #13 | 42362 |
| **15** | #5 OR #6 OR #7 OR #8 OR #9 OR #14 | 58247 |
| **16** | #4 and #15 | 324 |
| **17** | #16 AND Cochrane Database of Systematic Reviews Publication | 5 |
| **18** | #16 AND Cochrane Central Register of Controlled Trials Date | 319 |

TABLE S2 SUMMARY OF FINDINGS FOR KQ1

**Key Question 1: What are the risk factors for developing an incisional hernia after previous abdominal surgery?**

| **Certainty assessment** | | | | | | | **№ of patients** | | **Effect** | | **Certainty** | **Importance** |
| --- | --- | --- | --- | --- | --- | --- | --- | --- | --- | --- | --- | --- |
| **№ of studies** | **Study design** | **Risk of bias** | **Inconsistency** | **Indirectness** | **Imprecision** | **Other considerations** | **exposure to a specific (risk) factor** | **no exposure to that specific (risk) factor** | **Relative (95% CI)** | **Absolute (95% CI)** |  |  |
| **Risk of incisional hernia for type of previous incision (follow up median 30 m) Offline vs Middline (follow up: median 30 months)** | | | | | | | | | | | | |
| 13 | randomised trials | very serious^a^ | not serious | not serious | not serious | none | 65/1240 (5.2%) | 106/1058 (10.0%) | **RR 0.47** (0.30 to 0.75) | **53 fewer per 1,000** (from 70 fewer to 25 fewer) | ⨁⨁◯◯ Low | CRITICAL |
| **SILS vs CLS on hernia occurance** | | | | | | | | | | | | |
| 32 | randomised trials | not serious | not serious | not serious | very serious^b^ | none | 27/1861 (1.5%) | 11/2156 (0.5%) | **OR 1.92** (0.94 to 3.91) | **5 more per 1,000** (from 0 fewer to 15 more) | ⨁⨁◯◯ Low | IMPORTANT |
| **Gender as risk to IH (Male vvs Female)** | | | | | | | | | | | | |
| 10 | observational studies | not serious | not serious | not serious | not serious | none | 214/2539 (8.4%) | 196/2618 (7.5%) | **OR 1.14** (0.90 to 1.45) | **10 more per 1,000** (from 7 fewer to 30 more) | ⨁⨁◯◯ Low | IMPORTANT |
| **Risk of IH with diabetes (diabetes vs no diabetes)** | | | | | | | | | | | | |
| 7 | observational studies | not serious | not serious | not serious | not serious | none | 69/471 (14.6%) | 272/3109 (8.7%) | **OR 1.73** (1.30 to 2.32) | **55 more per 1,000** (from 23 more to 94 more) | ⨁⨁◯◯ Low | CRITICAL |
| **Risk with immunosuppression (Immuno vs no)** | | | | | | | | | | | | |
| 4 | observational studies | not serious | not serious | serious^c^ | not serious | none | 73/700 (10.4%) | 156/1998 (7.8%) | **OR 1.75** (1.28 to 2.38) | **51 more per 1,000** (from 20 more to 90 more) | ⨁◯◯◯ Very low | CRITICAL |
| **Risk smoking (current)** | | | | | | | | | | | | |
| 4 | observational studies | not serious | very serious^d^ | not serious | serious^e^ | none | 111/617 (18.0%) | 169/2181 (7.7%) | **OR 1.87** (1.36 to 2.57) | **58 more per 1,000** (from 25 more to 100 more) | ⨁◯◯◯ Very low | CRITICAL |
| **Risk SSI (SSI vs NO SSI)** | | | | | | | | | | | | |
| 9 | observational studies | not serious | not serious | not serious | not serious | strong association | 76/391 (19.4%) | 315/4542 (6.9%) | **OR 3.38** (2.18 to 5.23) | **132 more per 1,000** (from 70 more to 211 more) | ⨁⨁⨁◯ Moderate | CRITICAL |
| **BMI Underweight <18.5 kg/m2OR (95% CI)** | | | | | | | | | | | | |
| 1 | observational studies | not serious | not serious | not serious | not serious | none | 0/3580 (0.0%) | 0/736726 (0.0%) | **OR 0.92** (0.55 to 1.29) | **0 fewer per 1,000** (from 0 fewer to 0 fewer) | ⨁⨁◯◯ Low | CRITICAL |
| **Overweight25.0-29.9 kg/m2OR (95% CI)** | | | | | | | | | | | | |
| 1 | observational studies | not serious | not serious | not serious | not serious | none | 0/0 | 0/0 | **OR 1.65** (1.56 to 1.76) | **2 fewer per 1,000** (from 2 fewer to 2 fewer) | ⨁⨁◯◯ Low | CRITICAL |
| **Obesity Class 1 30.0-34.9 kg/m2OR (95% CI)** | | | | | | | | | | | | |
| 1 | observational studies | not serious | not serious | not serious | not serious | none | 0/0 | 0/0 | **OR 2.51** (2.40 to 2.62) | **3 fewer per 1,000** (from 3 fewer to 2 fewer) | ⨁⨁◯◯ Low | CRITICAL |
| **Obesity Class 2 35.0-39.9 kg/m2OR (95% CI)** | | | | | | | | | | | | |
| 1 | observational studies | not serious | not serious | not serious | not serious | none | 0/0 | 0/0 | **OR 3.62** (3.51 to 3.75) | **4 fewer per 1,000** (from 4 fewer to 4 fewer) | ⨁⨁◯◯ Low | CRITICAL |
| **Obesity Class 3 ≥40.0 kg/m2OR (95% CI)** | | | | | | | | | | | | |
| 1 | observational studies | not serious | not serious | not serious | not serious | none | 0/0 | 0/0 | **OR 5.48** (5.36 to 5.60) | **5 fewer per 1,000** (from 6 fewer to 5 fewer) | ⨁⨁◯◯ Low | CRITICAL |

**CI:** confidence interval; **OR:** odds ratio; **RR:** risk ratio

#### Explanations

a. Unclear risk of bias for most included studies

b. Wide CI and only 38 events total

c. Different exposure (different drugs as immunosuppression)

d. Confidence intervals are not overlaping, I2 is 86%

e. CI of included studies is wide

TABLE S3 SUMMARY OF FINDINGS FOR KQ2

**Key Question 2:**

**a) Do all patients with an incisional hernia require imaging? b) What is the best modality?**

a: Summary of findings table for sub question - Abdominal Ultrasound (US) Vs Physical examination

| Outcome | № of studies (№ of patients) | Study design | Factors that may decrease certainty of evidence | | | | | Effect per 1 000 patients tested | | | | | | Test accuracy CoE |
| --- | --- | --- | --- | --- | --- | --- | --- | --- | --- | --- | --- | --- | --- | --- |
|  |  |  |  |  |  |  |  | pre-test probability of17.9% | | pre-test probability of13.6% | | pre-test probability of28.9% | |  |
|  |  |  | Risk of bias | Indirectness | Inconsistency | Imprecision | Publication bias | abdominal ultrasound scanning | physical examination of the abdomen only | abdominal ultrasound scanning | physical examination of the abdomen only | abdominal ultrasound scanning | physical examination of the abdomen only |  |
| **True positives** (patients with incisional hernia ) | 3 studies 832 patients | cross-sectional (cohort type accuracy study) | serious^a^ | not serious | serious^b^ | not serious | none^c^ | 136 to 179 | 0 to 0 | 103 to 136 | 0 to 0 | 220 to 289 | 0 to 0 | ⨁⨁◯◯ Low |
|  |  |  |  |  |  |  |  | **136 more to 179 more TP in abdominal ultrasound scanning** | | **103 more to 136 more TP in abdominal ultrasound scanning** | | **220 more to 289 more TP in abdominal ultrasound scanning** | |  |
| **False negatives** (patients incorrectly classified as not having incisional hernia ) |  |  |  |  |  |  |  | 0 to 43 | 179 to 179 | 0 to 33 | 136 to 136 | 0 to 69 | 289 to 289 |  |
|  |  |  |  |  |  |  |  | **136 fewer to 179 fewer FN in abdominal ultrasound scanning** | | **103 fewer to 136 fewer FN in abdominal ultrasound scanning** | | **220 fewer to 289 fewer FN in abdominal ultrasound scanning** | |  |
| **True negatives** (patients without incisional hernia ) | 3 studies 832 patients | cross-sectional (cohort type accuracy study) | serious^a^ | not serious | serious^b^ | not serious | none^c^ | 361 to 772 | 0 to 0 | 380 to 812 | 0 to 0 | 313 to 668 | 0 to 0 | ⨁⨁◯◯ Low |
|  |  |  |  |  |  |  |  | **361 more to 772 more TN in abdominal ultrasound scanning** | | **380 more to 812 more TN in abdominal ultrasound scanning** | | **313 more to 668 more TN in abdominal ultrasound scanning** | |  |
| **False positives** (patients incorrectly classified as having incisional hernia ) |  |  |  |  |  |  |  | 49 to 460 | 821 to 821 | 52 to 484 | 864 to 864 | 43 to 398 | 711 to 711 |  |
|  |  |  |  |  |  |  |  | **361 fewer to 772 fewer FP in abdominal ultrasound scanning** | | **380 fewer to 812 fewer FP in abdominal ultrasound scanning** | | **313 fewer to 668 fewer FP in abdominal ultrasound scanning** | |  |

#### Explanations

a. Index and reference tests were problematic in all three studies

b. CIs are not overlaping

c. Studies identified in one SR with not exhaustive search strategy.

b: Summary of findings table for sub question - Abdominal Computed tomography (CT) Vs Physical examination

| Outcome | № of studies (№ of patients) | Study design | Factors that may decrease certainty of evidence | | | | | Effect per 1 000 patients tested | | | | | | Test accuracy CoE |
| --- | --- | --- | --- | --- | --- | --- | --- | --- | --- | --- | --- | --- | --- | --- |
|  |  |  |  |  |  |  |  | pre-test probability of44.2% | | pre-test probability of18% | | pre-test probability of30% | |  |
|  |  |  | Risk of bias | Indirectness | Inconsistency | Imprecision | Publication bias | Medical imaging | physical examination of the abdomen only | Medical imaging | physical examination of the abdomen only | Medical imaging | physical examination of the abdomen only |  |
| **True positives** (patients with incisional hernia) | 4 studies 770 patients | cross-sectional (cohort type accuracy study) | serious^a^ | not serious | serious^b^ | not serious | none^c^ | 265 to 420 | 0 to 0 | 108 to 171 | 0 to 0 | 180 to 285 | 0 to 0 | ⨁⨁◯◯ Low |
|  |  |  |  |  |  |  |  | **265 more to 420 more TP in Medical imaging** | | **108 more to 171 more TP in Medical imaging** | | **180 more to 285 more TP in Medical imaging** | |  |
| **False negatives** (patients incorrectly classified as not having incisional hernia) |  |  |  |  |  |  |  | 22 to 177 | 442 to 442 | 9 to 72 | 180 to 180 | 15 to 120 | 300 to 300 |  |
|  |  |  |  |  |  |  |  | **265 fewer to 420 fewer FN in Medical imaging** | | **108 fewer to 171 fewer FN in Medical imaging** | | **180 fewer to 285 fewer FN in Medical imaging** | |  |
| **True negatives** (patients without incisional hernia) | 4 studies 770 patients | cross-sectional (cohort type accuracy study) | serious^a^ | not serious | serious^b^ | not serious | none | 335 to 536 | 0 to 0 | 492 to 787 | 0 to 0 | 420 to 672 | 0 to 0 | ⨁⨁◯◯ Low |
|  |  |  |  |  |  |  |  | **335 more to 536 more TN in Medical imaging** | | **492 more to 787 more TN in Medical imaging** | | **420 more to 672 more TN in Medical imaging** | |  |
| **False positives** (patients incorrectly classified as having incisional hernia) |  |  |  |  |  |  |  | 22 to 223 | 558 to 558 | 33 to 328 | 820 to 820 | 28 to 280 | 700 to 700 |  |
|  |  |  |  |  |  |  |  | **335 fewer to 536 fewer FP in Medical imaging** | | **492 fewer to 787 fewer FP in Medical imaging** | | **420 fewer to 672 fewer FP in Medical imaging** | |  |

#### Explanations

a. 3 of 4 studies has an issue in the patient selection, flow and timing, two with index test interpretation domains

b. Confidence intervals are not ideally overlapping nor do sensitivity and specificity across the studies

c. Studies were retrieved from systematic review with not extensive search strategy

c: Summary of findings table for sub question- Ultrasound Vs CT

| Outcome | № of studies (№ of patients) | Study design | Factors that may decrease certainty of evidence | | | | | Effect per 1 000 patients tested | | | | | | Test accuracy CoE |
| --- | --- | --- | --- | --- | --- | --- | --- | --- | --- | --- | --- | --- | --- | --- |
|  |  |  |  |  |  |  |  | pre-test probability of60% | | pre-test probability of54.7% | | pre-test probability of0% | |  |
|  |  |  | Risk of bias | Indirectness | Inconsistency | Imprecision | Publication bias | abdominal ultrasound scanning | computed tomography scanning | abdominal ultrasound scanning | computed tomography scanning | abdominal ultrasound scanning | computed tomography scanning |  |
| **True positives** (patients with incisional hernia) | 2 studies 221 patients | cross-sectional (cohort type accuracy study) | not serious | not serious | not serious | serious^a^ | none^b^ | 426 to 588 | 0 to 0 | 388 to 536 | 0 to 0 | 0 to 0 | 0 to 0 | ⨁⨁⨁◯ Moderate |
|  |  |  |  |  |  |  |  | **426 more to 588 more TP in abdominal ultrasound scanning** | | **388 more to 536 more TP in abdominal ultrasound scanning** | | **0 fewer to 0 fewer TP in abdominal ultrasound scanning** | |  |
| **False negatives** (patients incorrectly classified as not having incisional hernia) |  |  |  |  |  |  |  | 12 to 174 | 600 to 600 | 11 to 159 | 547 to 547 | 0 to 0 | 0 to 0 |  |
|  |  |  |  |  |  |  |  | **426 fewer to 588 fewer FN in abdominal ultrasound scanning** | | **388 fewer to 536 fewer FN in abdominal ultrasound scanning** | | **0 fewer to 0 fewer FN in abdominal ultrasound scanning** | |  |
| **True negatives** (patients without incisional hernia) | 2 studies 221 patients | cross-sectional (cohort type accuracy study) | not serious | not serious | not serious | serious^a^ | none | 352 to 400 | 0 to 0 | 399 to 453 | 0 to 0 | 880 to 1000 | 0 to 0 | ⨁⨁⨁◯ Moderate |
|  |  |  |  |  |  |  |  | **352 more to 400 more TN in abdominal ultrasound scanning** | | **399 more to 453 more TN in abdominal ultrasound scanning** | | **880 more to 1000 more TN in abdominal ultrasound scanning** | |  |
| **False positives** (patients incorrectly classified as having incisional hernia) |  |  |  |  |  |  |  | 0 to 48 | 400 to 400 | 0 to 54 | 453 to 453 | 0 to 120 | 1000 to 1000 |  |
|  |  |  |  |  |  |  |  | **352 fewer to 400 fewer FP in abdominal ultrasound scanning** | | **399 fewer to 453 fewer FP in abdominal ultrasound scanning** | | **880 fewer to 1000 fewer FP in abdominal ultrasound scanning** | |  |

#### Explanations

a. Only two studies with total number of participants 221

b. Only two studies were identified in one systematic review with limited search

TABLE S4 SUMMARY OF FINDINGS FOR KQ3

**Key Question 3: Is it possible to predict from imaging whether the fascial closure will be possible?**

.Is exposure to imaging methods helpful for predicting use of the facial closure technique in adults with incisional hernias?

| **Certainty assessment** | | | | | | | **№ of patients** | | **Effect** | | **Certainty** | **Importance** |
| --- | --- | --- | --- | --- | --- | --- | --- | --- | --- | --- | --- | --- |
| **№ of studies** | **Study design** | **Risk of bias** | **Inconsistency** | **Indirectness** | **Imprecision** | **Other considerations** | **fascial closure** | **no fascial closure** | **Relative (95% CI)** | **Absolute (95% CI)** |  |  |
| **CSI** | | | | | | | | | | | | |
| 2 | observational studies | not serious | not serious | not serious | serious^a^ | none | 108 | 36 | - | MD **0.07 lower** (0.08 lower to 0.05 lower) | ⨁◯◯◯ Very low | CRITICAL |
| **Defect width (cm)** | | | | | | | | | | | | |
| 2 | observational studies | not serious | not serious | not serious | serious^a^ | none | 138 | 24 | - | MD **3.39 lower** (4.99 lower to 1.8 lower) | ⨁◯◯◯ Very low | CRITICAL |
| **Defect lenght (cm)** | | | | | | | | | | | | |
| 2 | observational studies | not serious | not serious | not serious | serious^a^ | none | 138 | 24 | - | MD **3.82 lower** (6.53 lower to 1.12 lower) | ⨁◯◯◯ Very low | CRITICAL |
| **Defect surface area (cm)** | | | | | | | | | | | | |
| 2 | observational studies | not serious | not serious | not serious | serious^a^ | none | 138 | 24 | - | MD **78.21 lower** (137.51 lower to 18.91 lower) | ⨁◯◯◯ Very low | CRITICAL |

**CI:** confidence interval; **MD:** mean difference

#### Explanations

a. Just two studies with small sample size

**Question:** Myofascial release required compared to myofascial release not required for predicting successful fascial closure after progressive pre-operative pneumoperitoneum in adults with a midline incisional hernia

| **Certainty assessment** | | | | | | | **№ of patients** | | **Effect** | | **Certainty** | **Importance** |
| --- | --- | --- | --- | --- | --- | --- | --- | --- | --- | --- | --- | --- |
| **№ of studies** | **Study design** | **Risk of bias** | **Inconsistency** | **Indirectness** | **Imprecision** | **Other considerations** | **myofascial release required** | **myofascial release not required** | **Relative (95% CI)** | **Absolute (95% CI)** |  |  |
| **CSI** | | | | | | | | | | | | |
| 1 | observational studies | not serious | not serious | not serious | serious^a^ | none | 134 | 208 | - | MD **0.07 higher** (0.06 higher to 0.09 higher) | ⨁◯◯◯ Very low | CRITICAL |
| **Rectus defect ration** | | | | | | | | | | | | |
| 1 | observational studies | not serious | not serious | not serious | serious^a^ | none | 134 | 208 | - | MD **1.2 lower** (1.45 lower to 0.95 lower) | ⨁◯◯◯ Very low | CRITICAL |
| **Defect width (cm)** | | | | | | | | | | | | |
| 2 | observational studies | not serious | not serious | not serious | serious^a^ | none | 169 | 224 | - | MD **5.15 higher** (4.34 higher to 5.95 higher) | ⨁◯◯◯ Very low | CRITICAL |
| **Defect surface area (IHOA)** | | | | | | | | | | | | |
| 2 | observational studies | not serious | not serious | not serious | serious^a^ | none | 46 | 131 | - | MD **118.15 higher** (93.42 higher to 142.89 higher) | ⨁◯◯◯ Very low | CRITICAL |

**CI:** confidence interval; **MD:** mean difference

#### Explanations

a. Data just from one study

TABLE S5 SUMMARY OF FINDINGS FOR KQ4

**Key Question 4:**

**b) What are the important outcome measures in treatment of incisional hernias?**

**Question:** Surgery approach compared to non-surgery approach in treatment of incisional hernias?

| **Certainty assessment** | | | | | | | **№ of patients** | | **Effect** | | **Certainty** | **Importance** |
| --- | --- | --- | --- | --- | --- | --- | --- | --- | --- | --- | --- | --- |
| **№ of studies** | **Study design** | **Risk of bias** | **Inconsistency** | **Indirectness** | **Imprecision** | **Other considerations** | **Surgery approach** | **non-surgery approach** | **Relative (95% CI)** | **Absolute (95% CI)** |  |  |
| **Physical functioning** | | | | | | | | | | | | |
| 1 | observational studies | not serious | serious^a^ | not serious | not serious | none | 121 | 74 | - | MD **9.1 higher** (1.64 higher to 16.56 higher) | ⨁◯◯◯ Very low | CRITICAL |
| **Role physical sum score** | | | | | | | | | | | | |
| 1 | observational studies | not serious | serious^a^ | not serious | not serious | none | 121 | 74 | - | MD **15.9 higher** (10.58 higher to 21.22 higher) | ⨁◯◯◯ Very low | CRITICAL |
| **Bodily pain sum score** | | | | | | | | | | | | |
| 1 | observational studies | not serious | serious^a^ | not serious | not serious | none | 121 | 74 | - | MD **11.5 higher** (3.75 higher to 19.25 higher) | ⨁◯◯◯ Very low | CRITICAL |
| **General health** | | | | | | | | | | | | |
| 1 | observational studies | not serious | serious^a^ | not serious | not serious | none | 121 | 74 | - | MD **3.7 higher** (18.62 lower to 26.02 higher) | ⨁◯◯◯ Very low | CRITICAL |
| **Vitality sum score** | | | | | | | | | | | | |
| 1 | observational studies | not serious | serious^a^ | not serious | not serious | none | 121 | 74 | - | MD **8 higher** (1 higher to 15 higher) | ⨁◯◯◯ Very low | CRITICAL |
| **Social functioning** | | | | | | | | | | | | |
| 1 | observational studies | not serious | serious^a^ | not serious | not serious | none | 121 | 74 | - | MD **9.3 higher** (0.56 higher to 18.04 higher) | ⨁◯◯◯ Very low | CRITICAL |
| **Role emotional sum score** | | | | | | | | | | | | |
| 1 | observational studies | not serious | serious^a^ | not serious | not serious | none | 121 | 74 | - | MD **9.2 higher** (0.18 higher to 18.22 higher) | ⨁◯◯◯ Very low | CRITICAL |
| **Mental health sum score** | | | | | | | | | | | | |
| 1 | observational studies | not serious | serious^a^ | not serious | not serious | none | 121 | 74 | - | MD **1.7 higher** (1.13 lower to 4.53 higher) | ⨁◯◯◯ Very low | CRITICAL |

TABLE S7: SUMMARY OF FINDINGS FOR KQ6

**Key Question 6: What is the difference in outcome for mesh versus suture repair in incisional hernia repair?**

**Question:** Mesh compared to tissue repair with sutures for the elective surgical treatment of incisional hernias in adult patients

| **Certainty assessment** | | | | | | | **№ of patients** | | **Effect** | | **Certainty** | **Importance** |
| --- | --- | --- | --- | --- | --- | --- | --- | --- | --- | --- | --- | --- |
| **№ of studies** | **Study design** | **Risk of bias** | **Inconsistency** | **Indirectness** | **Imprecision** | **Other considerations** | **mesh** | **tissue repair with sutures** | **Relative (95% CI)** | **Absolute (95% CI)** |  |  |
| **Recurrence** | | | | | | | | | | | | |
| 5 | randomised trials | very serious^a^ | not serious | not serious | not serious | none | 58/490 (11.8%) | 135/444 (30.4%) | **OR 0.31** (0.21 to 0.44) | **185 fewer per 1 000** (from 220 fewer to 143 fewer) | ⨁⨁◯◯ Low | CRITICAL |
| **Recurrence - Mesh onlay (polypropylene)** | | | | | | | | | | | | |
| 3 | randomised trials | very serious^a^ | not serious | not serious | serious^b^ | none | 9/124 (7.3%) | 19/113 (16.8%) | **OR 0.39** (0.17 to 0.90) | **95 fewer per 1 000** (from 135 fewer to 14 fewer) | ⨁◯◯◯ Very low | CRITICAL |
| **Recurrence - Mesh sublay (polypropylene)** | | | | | | | | | | | | |
| 3 | randomised trials | very serious^a^ | not serious | not serious | not serious | none | 49/366 (13.4%) | 116/331 (35.0%) | **OR 0.29** (0.20 to 0.43) | **215 fewer per 1 000** (from 253 fewer to 162 fewer) | ⨁⨁◯◯ Low | CRITICAL |
| **Infection** | | | | | | | | | | | | |
| 2 | randomised trials | very serious^a^ | not serious | not serious | very serious^b^ | none | 6/71 (8.5%) | 5/63 (7.9%) | **OR 1.07** (0.33 to 3.49) | **5 more per 1 000** (from 52 fewer to 152 more) | ⨁◯◯◯ Very low | CRITICAL |
| **Infection - Mesh onlay (polypropylene)** | | | | | | | | | | | | |
| 2 | randomised trials | very serious^a^ | not serious | not serious | very serious^b^ | none | 6/71 (8.5%) | 5/63 (7.9%) | **OR 1.07** (0.33 to 3.49) | **5 more per 1 000** (from 52 fewer to 152 more) | ⨁◯◯◯ Very low | CRITICAL |
| **Infection - Mesh sublay (polypropylene)** | | | | | | | | | | | | |
| 0 |  |  |  |  |  |  | 0/0 | 0/0 | not pooled | see comment | - | CRITICAL |
| **Hematoma** | | | | | | | | | | | | |
| 3 | randomised trials | very serious^a^ | not serious | not serious | very serious^b^ | none | 0/226 (0.0%) | 13/163 (8.0%) | **OR 0.10** (0.02 to 0.43) | **71 fewer per 1 000** (from 78 fewer to 44 fewer) | ⨁◯◯◯ Very low | CRITICAL |
| **Hematoma - Mesh onlay (polypropylene)** | | | | | | | | | | | | |
| 3 | randomised trials | very serious^a^ | not serious | not serious | very serious^b^ | none | 0/124 (0.0%) | 10/113 (8.8%) | **OR 0.11** (0.02 to 0.60) | **78 fewer per 1 000** (from 87 fewer to 33 fewer) | ⨁◯◯◯ Very low | CRITICAL |
| **Hematoma - Mesh sublay (polypropylene)** | | | | | | | | | | | | |
| 1 | randomised trials | very serious^a^ | not serious | not serious | very serious^c^ | none | 0/102 (0.0%) | 3/50 (6.0%) | **OR 0.07** (0.00 to 1.31) | **56 fewer per 1 000** (from -- to 17 more) | ⨁◯◯◯ Very low | CRITICAL |
| **Seroma** | | | | | | | | | | | | |
| 3 | randomised trials | very serious^a^ | not serious | not serious | very serious^c^ | none | 43/226 (19.0%) | 11/163 (6.7%) | **OR 3.48** (1.75 to 6.93) | **134 more per 1 000** (from 45 more to 267 more) | ⨁◯◯◯ Very low | CRITICAL |
| **Seroma - Mesh onlay (polypropylene)** | | | | | | | | | | | | |
| 3 | randomised trials | very serious^a^ | not serious | not serious | very serious^c^ | none | 31/124 (25.0%) | 6/113 (5.3%) | **OR 6.78** (2.69 to 17.10) | **222 more per 1 000** (from 78 more to 436 more) | ⨁◯◯◯ Very low | CRITICAL |
| **Seroma - Mesh sublay (polypropylene)** | | | | | | | | | | | | |
| 1 | randomised trials | very serious^a^ | not serious | not serious | very serious^c^ | none | 12/102 (11.8%) | 5/50 (10.0%) | **OR 1.20** (0.40 to 3.62) | **18 more per 1 000** (from 57 fewer to 187 more) | ⨁◯◯◯ Very low | CRITICAL |
| **Lenght of stay** | | | | | | | | | | | | |
| 3 | randomised trials | very serious^a^ | not serious | not serious | serious^b^ | none | 226 | 163 | - | MD **1.08 higher** (0.53 higher to 1.63 higher) | ⨁◯◯◯ Very low | IMPORTANT |
| **Lenght of stay - Mesh onlay (polypropylene)** | | | | | | | | | | | | |
| 3 | randomised trials | very serious^a^ | not serious | not serious | serious^b^ | none | 124 | 113 | - | MD **1.55 higher** (0.83 higher to 2.27 higher) | ⨁◯◯◯ Very low | IMPORTANT |
| **Lenght of stay - Mesh sublay (polypropylene)** | | | | | | | | | | | | |
| 1 | randomised trials | very serious^a^ | not serious | not serious | serious^b^ | none | 102 | 50 | - | MD **0.4 higher** (0.46 lower to 1.26 higher) | ⨁◯◯◯ Very low | IMPORTANT |

**CI:** confidence interval; **MD:** mean difference; **OR:** odds ratio

#### Explanations

a. Most of the bias domains are unclear across all studies

b. Very small sample size

c. Small sample size and wide CI

TABLE S8: SUMMARY OF FINDINGS FOR KQ7

**Key Question 7: What is the difference in outcome considering different positions of mesh in incisional hernia repair?**

**Question:** Onlay mesh position compared to retro muscular (sublay) mesh position for the elective surgical repair of incisional hernia in adult patients

| **Certainty assessment** | | | | | | | **№ of patients** | | **Effect** | | **Certainty** | **Importance** |
| --- | --- | --- | --- | --- | --- | --- | --- | --- | --- | --- | --- | --- |
| **№ of studies** | **Study design** | **Risk of bias** | **Inconsistency** | **Indirectness** | **Imprecision** | **Other considerations** | **onlay mesh position** | **retro muscular (sublay) mesh position** | **Relative (95% CI)** | **Absolute (95% CI)** |  |  |
| **Reccurence** | | | | | | | | | | | | |
| 4 | randomised trials | serious^a^ | not serious | not serious | very serious^b^ | none | 14/194 (7.2%) | 4/187 (2.1%) | **OR 3.24** (1.10 to 9.52) | **45 more per 1 000** (from 2 more to 151 more) | ⨁◯◯◯ Very low | CRITICAL |
| **Seroma** | | | | | | | | | | | | |
| 4 | randomised trials | serious^a^ | not serious | not serious | serious^c^ | none | 66/198 (33.3%) | 26/188 (13.8%) | **OR 3.32** (1.96 to 5.62) | **209 more per 1 000** (from 101 more to 336 more) | ⨁⨁◯◯ Low | CRITICAL |
| **Lenght of stay** | | | | | | | | | | | | |
| 3 | randomised trials | serious^a^ | not serious | not serious | serious^d^ | none | 181 | 176 | - | MD **0.21 higher** (0.26 lower to 0.68 higher) | ⨁⨁◯◯ Low | CRITICAL |
| **Hematoma** | | | | | | | | | | | | |
| 3 | randomised trials | serious^a^ | not serious | not serious | extremely serious^c^ | none | 5/185 (2.7%) | 3/177 (1.7%) | **OR 1.68** (0.39 to 7.17) | **11 more per 1 000** (from 10 fewer to 93 more) | ⨁◯◯◯ Very low | CRITICAL |

**CI:** confidence interval; **MD:** mean difference; **OR:** odds ratio

#### Explanations

a. RoB across all included studies is serious, Randomization, Allocation concealment and blinding are unclear

b. Very small studies are included with very small number of events

c. Very small studies are included with small number of events

d. CI is wide

**Question:** Onlay mesh compared to intraperitoneal-mesh (open IPOM) for incisional hernia

| **Certainty assessment** | | | | | | | **№ of patients** | | **Effect** | | **Certainty** | **Importance** |
| --- | --- | --- | --- | --- | --- | --- | --- | --- | --- | --- | --- | --- |
| **№ of studies** | **Study design** | **Risk of bias** | **Inconsistency** | **Indirectness** | **Imprecision** | **Other considerations** | **onlay mesh** | **intraperitoneal-mesh (open IPOM)** | **Relative (95% CI)** | **Absolute (95% CI)** |  |  |
| **Seroma** | | | | | | | | | | | | |
| 1 | randomised trials | not serious | not serious | not serious | extremely serious^a^ | none | 7/22 (31.8%) | 0/19 (0.0%) | **OR 18.87** (1.00 to 356.74) | **0 fewer per 1 000** (from 0 fewer to 0 fewer) | ⨁◯◯◯ Very low | CRITICAL |
| **SSI** | | | | | | | | | | | | |
| 1 | randomised trials | not serious | not serious | not serious | extremely serious^a^ | none | 1/22 (4.5%) | 1/19 (5.3%) | **OR 0.86** (0.05 to 14.71) | **7 fewer per 1 000** (from 50 fewer to 397 more) | ⨁◯◯◯ Very low | CRITICAL |
| **Recurence after 1 year** | | | | | | | | | | | | |
| 1 | randomised trials | not serious | not serious | not serious | extremely serious^a^ | none | 6/22 (27.3%) | 0/19 (0.0%) | **OR 15.36** (0.80 to 293.60) | **0 fewer per 1 000** (from 0 fewer to 0 fewer) | ⨁◯◯◯ Very low | CRITICAL |
| **Relevant pain** | | | | | | | | | | | | |
| 1 | randomised trials | not serious | not serious | not serious | extremely serious^a^ | none | 1/22 (4.5%) | 6/19 (31.6%) | **OR 0.10** (0.01 to 0.96) | **272 fewer per 1 000** (from 311 fewer to 9 fewer) | ⨁◯◯◯ Very low | CRITICAL |

**CI:** confidence interval; **OR:** odds ratio

#### Explanations

a. Very small single study with huge CI

**Question:** Minimal invasive sublay (MILOS) compared to minimal invasive intraperitoneal mesh (lap. IPOM) for incisional hernia repair

| **Certainty assessment** | | | | | | | **№ of patients** | | **Effect** | | **Certainty** | **Importance** |
| --- | --- | --- | --- | --- | --- | --- | --- | --- | --- | --- | --- | --- |
| **№ of studies** | **Study design** | **Risk of bias** | **Inconsistency** | **Indirectness** | **Imprecision** | **Other considerations** | **minimal invasive sublay (MILOS)** | **minimal invasive intraperitoneal mesh (lap. IPOM)** | **Relative (95% CI)** | **Absolute (95% CI)** |  |  |
| **Seroma** | | | | | | | | | | | | |
| 1 | observational studies | not serious | not serious | not serious | serious^a^ | none | 3/541 (0.6%) | 18/541 (3.3%) | **OR 0.16** (0.05 to 0.55) | **28 fewer per 1 000** (from 32 fewer to 15 fewer) | ⨁◯◯◯ Very low | CRITICAL |
| **General complications** | | | | | | | | | | | | |
| 1 | observational studies | not serious | not serious | not serious | serious^a^ | none | 6/541 (1.1%) | 22/541 (4.1%) | **OR 0.26** (0.11 to 0.66) | **30 fewer per 1 000** (from 36 fewer to 13 fewer) | ⨁◯◯◯ Very low | CRITICAL |
| **SSO** | | | | | | | | | | | | |
| 1 | observational studies | not serious | not serious | not serious | serious^a^ | none | 7/541 (1.3%) | 31/541 (5.7%) | **OR 0.22** (0.09 to 0.49) | **44 fewer per 1 000** (from 52 fewer to 28 fewer) | ⨁◯◯◯ Very low | CRITICAL |
| **Hematoma** | | | | | | | | | | | | |
| 1 | observational studies | not serious | not serious | not serious | serious^a^ | none | 3/541 (0.6%) | 9/541 (1.7%) | **OR 0.33** (0.09 to 1.22) | **11 fewer per 1 000** (from 15 fewer to 4 more) | ⨁◯◯◯ Very low | CRITICAL |
| **Recurrence** | | | | | | | | | | | | |
| 1 | observational studies | not serious | not serious | not serious | serious^a^ | none | 10/463 (2.2%) | 34/463 (7.3%) | **OR 0.28** (0.14 to 0.57) | **52 fewer per 1 000** (from 62 fewer to 30 fewer) | ⨁◯◯◯ Very low | CRITICAL |
| **Chronic pain during activity** | | | | | | | | | | | | |
| 1 | observational studies | not serious | not serious | not serious | serious^a^ | none | 25/463 (5.4%) | 115/463 (24.8%) | **OR 0.17** (0.11 to 0.27) | **195 fewer per 1 000** (from 213 fewer to 166 fewer) | ⨁◯◯◯ Very low | CRITICAL |
| **Chronic pain requiring treatment** | | | | | | | | | | | | |
| 1 | observational studies | not serious | not serious | not serious | serious^a^ | none | 12/463 (2.6%) | 42/463 (9.1%) | **OR 0.27** (0.14 to 0.51) | **64 fewer per 1 000** (from 77 fewer to 42 fewer) | ⨁◯◯◯ Very low | CRITICAL |
| **Chronic pain at rest after 1 year** | | | | | | | | | | | | |
| 1 | observational studies | not serious | not serious | not serious | serious^a^ | none | 17/463 (3.7%) | 65/463 (14.0%) | **OR 0.23** (0.13 to 0.40) | **104 fewer per 1 000** (from 120 fewer to 79 fewer) | ⨁◯◯◯ Very low | CRITICAL |

**CI:** confidence interval; **OR:** odds ratio

#### Explanations

a. Just one study small number of events

TABLE S9: SUMMARY OF FINDINGS FOR KQ8

**Key Question 8: What is the difference in outcome between techniques (open, laparoscopic and robotic) for incisional hernia repair?**

**Question:** Open surgery compared to laparoscopy surgery for the elective surgical repair of incisional hernia in adult patients

| **Certainty assessment** | | | | | | | **№ of patients** | | **Effect** | | **Certainty** | **Importance** |
| --- | --- | --- | --- | --- | --- | --- | --- | --- | --- | --- | --- | --- |
| **№ of studies** | **Study design** | **Risk of bias** | **Inconsistency** | **Indirectness** | **Imprecision** | **Other considerations** | **open surgery** | **laparoscopy surgery** | **Relative (95% CI)** | **Absolute (95% CI)** |  |  |
| **Recurrence** | | | | | | | | | | | | |
| 3 | randomised trials | serious^a^ | not serious | not serious | serious^b^ | none | 16/246 (6.5%) | 24/242 (9.9%) | **OR 0.62** (0.31 to 1.25) | **35 fewer per 1 000** (from 66 fewer to 22 more) | ⨁⨁◯◯ Low | CRITICAL |
| **Surgical site infection** | | | | | | | | | | | | |
| 5 | randomised trials | serious^a^ | serious^c^ | not serious | serious^b^ | none | 30/277 (10.8%) | 8/261 (3.1%) | **OR 2.68** (0.58 to 12.31) | **47 more per 1 000** (from 13 fewer to 250 more) | ⨁◯◯◯ Very low | CRITICAL |
| **Deep infection** | | | | | | | | | | | | |
| 5 | randomised trials | serious^a^ | not serious | not serious | very serious^d^ | none | 5/277 (1.8%) | 4/261 (1.5%) | **OR 1.07** (0.30 to 3.83) | **1 more per 1 000** (from 11 fewer to 41 more) | ⨁◯◯◯ Very low | CRITICAL |
| **Laparotomy** | | | | | | | | | | | | |
| 5 | randomised trials | serious^a^ | not serious | not serious | serious^b^ | none | 9/277 (3.2%) | 12/261 (4.6%) | **OR 0.69** (0.28 to 1.66) | **14 fewer per 1 000** (from 33 fewer to 28 more) | ⨁⨁◯◯ Low | CRITICAL |

**CI:** confidence interval; **OR:** odds ratio

#### Explanations

a. RoB across all included studies is serious, Randomization, Allocation concealment and blinding are high or unclear

b. Small number of events

c. Unexplained statistical heterogeneity,

d. Very low number of events and wide confidence interval, unclear benefit or harm

**Question:** Laparoscopic surgery compared to robotic surgery for elective surgical repair of incisional hernia in adult patients

| **Certainty assessment** | | | | | | | **№ of patients** | | **Effect** | | **Certainty** | **Importance** |
| --- | --- | --- | --- | --- | --- | --- | --- | --- | --- | --- | --- | --- |
| **№ of studies** | **Study design** | **Risk of bias** | **Inconsistency** | **Indirectness** | **Imprecision** | **Other considerations** | **laparoscopic surgery** | **robotic surgery** | **Relative (95% CI)** | **Absolute (95% CI)** |  |  |
| **Recurrence** | | | | | | | | | | | | |
| 1 | randomised trials | not serious | not serious | not serious | extremely serious^a^ | none | 5/59 (8.5%) | 4/65 (6.2%) | **OR 1.41** (0.36 to 5.53) | **23 more per 1 000** (from 38 fewer to 205 more) | ⨁◯◯◯ Very low | CRITICAL |

**CI:** confidence interval; **OR:** odds ratio

#### Explanations

a. Small number of events, one study

**Question:** Onlay mesh compared to intraperitoneal-mesh (open IPOM) for incisional hernia

| **Certainty assessment** | | | | | | | **№ of patients** | | **Effect** | | **Certainty** | **Importance** |
| --- | --- | --- | --- | --- | --- | --- | --- | --- | --- | --- | --- | --- |
| **№ of studies** | **Study design** | **Risk of bias** | **Inconsistency** | **Indirectness** | **Imprecision** | **Other considerations** | **onlay mesh** | **intraperitoneal-mesh (open IPOM)** | **Relative (95% CI)** | **Absolute (95% CI)** |  |  |
| **Seroma** | | | | | | | | | | | | |
| 1 | randomised trials | not serious | not serious | not serious | extremely serious^a^ | none | 7/22 (31.8%) | 0/19 (0.0%) | **OR 18.87** (1.00 to 356.74) | **0 fewer per 1 000** (from 0 fewer to 0 fewer) | ⨁◯◯◯ Very low | CRITICAL |
| **SSI** | | | | | | | | | | | | |
| 1 | randomised trials | not serious | not serious | not serious | extremely serious^a^ | none | 1/22 (4.5%) | 1/19 (5.3%) | **OR 0.86** (0.05 to 14.71) | **7 fewer per 1 000** (from 50 fewer to 397 more) | ⨁◯◯◯ Very low | CRITICAL |
| **Recurence after 1 year** | | | | | | | | | | | | |
| 1 | randomised trials | not serious | not serious | not serious | extremely serious^a^ | none | 6/22 (27.3%) | 0/19 (0.0%) | **OR 15.36** (0.80 to 293.60) | **0 fewer per 1 000** (from 0 fewer to 0 fewer) | ⨁◯◯◯ Very low | CRITICAL |
| **Relevant pain** | | | | | | | | | | | | |
| 1 | randomised trials | not serious | not serious | not serious | extremely serious^a^ | none | 1/22 (4.5%) | 6/19 (31.6%) | **OR 0.10** (0.01 to 0.96) | **272 fewer per 1 000** (from 311 fewer to 9 fewer) | ⨁◯◯◯ Very low | CRITICAL |

**CI:** confidence interval; **OR:** odds ratio

#### Explanations

a. Very small single study with huge CI

**Question:** Minimal invasive sublay (MILOS) compared to minimal invasive intraperitoneal mesh (lap. IPOM) for incisional hernia repair

| **Certainty assessment** | | | | | | | **№ of patients** | | **Effect** | | **Certainty** | **Importance** |
| --- | --- | --- | --- | --- | --- | --- | --- | --- | --- | --- | --- | --- |
| **№ of studies** | **Study design** | **Risk of bias** | **Inconsistency** | **Indirectness** | **Imprecision** | **Other considerations** | **minimal invasive sublay (MILOS)** | **minimal invasive intraperitoneal mesh (lap. IPOM)** | **Relative (95% CI)** | **Absolute (95% CI)** |  |  |
| **Seroma** | | | | | | | | | | | | |
| 1 | observational studies | not serious | not serious | not serious | serious^a^ | none | 3/541 (0.6%) | 18/541 (3.3%) | **OR 0.16** (0.05 to 0.55) | **28 fewer per 1 000** (from 32 fewer to 15 fewer) | ⨁◯◯◯ Very low | CRITICAL |
| **General complications** | | | | | | | | | | | | |
| 1 | observational studies | not serious | not serious | not serious | serious^a^ | none | 6/541 (1.1%) | 22/541 (4.1%) | **OR 0.26** (0.11 to 0.66) | **30 fewer per 1 000** (from 36 fewer to 13 fewer) | ⨁◯◯◯ Very low | CRITICAL |
| **SSO** | | | | | | | | | | | | |
| 1 | observational studies | not serious | not serious | not serious | serious^a^ | none | 7/541 (1.3%) | 31/541 (5.7%) | **OR 0.22** (0.09 to 0.49) | **44 fewer per 1 000** (from 52 fewer to 28 fewer) | ⨁◯◯◯ Very low | CRITICAL |
| **Hematoma** | | | | | | | | | | | | |
| 1 | observational studies | not serious | not serious | not serious | serious^a^ | none | 3/541 (0.6%) | 9/541 (1.7%) | **OR 0.33** (0.09 to 1.22) | **11 fewer per 1 000** (from 15 fewer to 4 more) | ⨁◯◯◯ Very low | CRITICAL |
| **Recurrence** | | | | | | | | | | | | |
| 1 | observational studies | not serious | not serious | not serious | serious^a^ | none | 10/463 (2.2%) | 34/463 (7.3%) | **OR 0.28** (0.14 to 0.57) | **52 fewer per 1 000** (from 62 fewer to 30 fewer) | ⨁◯◯◯ Very low | CRITICAL |
| **Chronic pain during activity** | | | | | | | | | | | | |
| 1 | observational studies | not serious | not serious | not serious | serious^a^ | none | 25/463 (5.4%) | 115/463 (24.8%) | **OR 0.17** (0.11 to 0.27) | **195 fewer per 1 000** (from 213 fewer to 166 fewer) | ⨁◯◯◯ Very low | CRITICAL |
| **Chronic pain requiring treatment** | | | | | | | | | | | | |
| 1 | observational studies | not serious | not serious | not serious | serious^a^ | none | 12/463 (2.6%) | 42/463 (9.1%) | **OR 0.27** (0.14 to 0.51) | **64 fewer per 1 000** (from 77 fewer to 42 fewer) | ⨁◯◯◯ Very low | CRITICAL |
| **Chronic pain at rest after 1 year** | | | | | | | | | | | | |
| 1 | observational studies | not serious | not serious | not serious | serious^a^ | none | 17/463 (3.7%) | 65/463 (14.0%) | **OR 0.23** (0.13 to 0.40) | **104 fewer per 1 000** (from 120 fewer to 79 fewer) | ⨁◯◯◯ Very low | CRITICAL |

**CI:** confidence interval; **OR:** odds ratio

#### Explanations

a. just one study small number of events

TABLE S9: SUMMARY OF FINDINGS FOR KQ9

**Key Question 9:**

**Is there a benefit of primary fascial closure in midline incisional hernia mesh repair?**

**Question:** Fascial closure compared to bridging for the elective surgical repair of incisional hernia in adult patients

| **Certainty assessment** | | | | | | | **№ of patients** | | **Effect** | | **Certainty** | **Importance** |
| --- | --- | --- | --- | --- | --- | --- | --- | --- | --- | --- | --- | --- |
| **№ of studies** | **Study design** | **Risk of bias** | **Inconsistency** | **Indirectness** | **Imprecision** | **Other considerations** | **fascial closure** | **bridging** | **Relative (95% CI)** | **Absolute (95% CI)** |  |  |
| **Recurrence** | | | | | | | | | | | | |
| 3 | randomised trials | not serious | not serious | not serious | very serious^a^ | none | 7/168 (4.2%) | 12/177 (6.8%) | **OR 0.60** (0.23 to 1.57) | **26 fewer per 1 000** (from 51 fewer to 35 more) | ⨁⨁◯◯ Low | CRITICAL |
| **QoL (mAAS Scale) difference from baseline after 2 years** | | | | | | | | | | | | |
| 1 | randomised trials | not serious | not serious | not serious | very serious^b^ | none | 64 | 65 | - | MD **11.6 higher** (1.2 higher to 22 higher) | ⨁⨁◯◯ Low | CRITICAL |
| **Morbidity (Hematoma, Seroma)** | | | | | | | | | | | | |
| 3 | randomised trials | not serious | not serious | not serious | very serious^c^ | none | 14/228 (6.1%) | 27/239 (11.3%) | **OR 0.52** (0.27 to 1.02) | **51 fewer per 1 000** (from 80 fewer to 2 more) | ⨁⨁◯◯ Low | CRITICAL |
| **Morbidity (Hematoma, Seroma) - Hematoma** | | | | | | | | | | | | |
| 1 | randomised trials | not serious | not serious | not serious | extremely serious^b^ | none | 0/61 (0.0%) | 3/62 (4.8%) | **OR 0.14** (0.01 to 2.73) | **41 fewer per 1 000** (from 48 fewer to 74 more) | ⨁◯◯◯ Very low | CRITICAL |
| **Morbidity (Hematoma, Seroma) - Seroma** | | | | | | | | | | | | |
| 2 | randomised trials | not serious | not serious | not serious | very serious^c^ | none | 9/85 (10.6%) | 12/87 (13.8%) | **OR 0.75** (0.30 to 1.84) | **31 fewer per 1 000** (from 92 fewer to 90 more) | ⨁⨁◯◯ Low | CRITICAL |
| **Morbidity (Hematoma, Seroma) - Hematoma and Seroma combined** | | | | | | | | | | | | |
| 1 | randomised trials | not serious | not serious | not serious | extremely serious^b^ | none | 5/82 (6.1%) | 12/90 (13.3%) | **OR 0.42** (0.14 to 1.26) | **73 fewer per 1 000** (from 112 fewer to 29 more) | ⨁◯◯◯ Very low | CRITICAL |
| **Pain (VAS) chronic 6 - 24 months** | | | | | | | | | | | | |
| 3 | randomised trials | not serious | serious^d^ | not serious | serious^e^ | none | 161 | 168 | - | MD **0.01 higher** (0.32 lower to 0.33 higher) | ⨁⨁◯◯ Low | CRITICAL |

**CI:** confidence interval; **MD:** mean difference; **OR:** odds ratio

#### Explanations

a. Just 15 events total

b. Just one study with smaller sample size

c. For Hematoma and Seroma sub groups, there are very small samples and wide CI

d. Significant statistical heterogeneity

e. Small number of studies and participants

TABLE S10: SUMMARY OF FINDINGS FOR KQ10

**Key Question 10: What is the difference in the outcome using different techniques for mesh fixation in:**

**(a) intraperitoneal and**

**(b) extraperitoneal mesh placement for incisional hernia repair?**

Question: Absorbable tacks compared to non-absorbable tacks for mesh fixation in minimally invasive elective repair of incisional hernia in adult patients

| **Certainty assessment** | | | | | | | **№ of patients** | | **Effect** | | **Certainty** | **Importance** |
| --- | --- | --- | --- | --- | --- | --- | --- | --- | --- | --- | --- | --- |
| **№ of studies** | **Study design** | **Risk of bias** | **Inconsistency** | **Indirectness** | **Imprecision** | **Other considerations** | **absorbable tacks** | **non absorbable tack** | **Relative (95% CI)** | **Absolute (95% CI)** |  |  |
| **Seroma** | | | | | | | | | | | | |
| 2 | randomised trials | not serious | not serious | not serious | extremely serious | none | 6/71 (8.5%) | 8/70 (11.4%) | **OR 0.70** (0.22 to 2.17) | **31 fewer per 1 000** (from 87 fewer to 104 more) | ⨁◯◯◯ Very low | CRITICAL |
| **Length of stay** | | | | | | | | | | | | |
| 2 | randomised trials | not serious | not serious | not serious | extremely serious | none | 71 | 70 | - | MD **0.01 higher** (0.63 lower to 0.64 higher) | ⨁◯◯◯ Very low | CRITICAL |
| **QoL (WHO BREF)** | | | | | | | | | | | | |
| 1 | randomised trials | not serious | not serious | not serious | extremely serious | none | 45 | 45 | - | MD **0.1 higher** (0.15 lower to 0.35 higher) | ⨁◯◯◯ Very low | CRITICAL |
| **Recurrence** | | | | | | | | | | | | |
| 3 | randomised trials | not serious | not serious | not serious | extremely serious | none | 3/96 (3.1%) | 4/95 (4.2%) | **OR 0.72** (0.15 to 3.38) | **11 fewer per 1 000** (from 36 fewer to 87 more) | ⨁◯◯◯ Very low | CRITICAL |

**CI:** confidence interval; **MD:** mean difference; **OR:** odds ratio

#### Explanations

a. Only two small studies with very small number of events

b. Only two small studies with not overlapping CI

c. Only one very small study

d. Only three very small studies with very small number of events

Question: Glue compared to tacks for mesh fixation in minimally invasive elective repair of incisional hernia in adult patients

| **Certainty assessment** | | | | | | | **№ of patients** | | **Effect** | | **Certainty** | **Importance** |
| --- | --- | --- | --- | --- | --- | --- | --- | --- | --- | --- | --- | --- |
| **№ of studies** | **Study design** | **Risk of bias** | **Inconsistency** | **Indirectness** | **Imprecision** | **Other considerations** | **glue fixation** | **tack fixation** | **Relative (95% CI)** | **Absolute (95% CI)** |  |  |
| **Pain (Caroline scale)** | | | | | | | | | | | | |
| 1 | randomised trials | not serious | not serious | not serious | extremely serious | none | 5/25 (20.0%) | 7/50 (14.0%) | **OR 1.54** (0.43 to 5.44) | **60 more per 1 000** (from 75 fewer to 330 more) | ⨁◯◯◯ Very low | CRITICAL |
| **Recurrence** | | | | | | | | | | | | |
| 1 | randomised trials | not serious | not serious | not serious | extremely serious | none | 0/25 (0.0%) | 3/50 (6.0%) | **OR 0.27** (0.01 to 5.36) | **43 fewer per 1 000** (from 59 fewer to 195 more) | ⨁◯◯◯ Very low | CRITICAL |

**CI:** confidence interval; **OR:** odds ratio

#### Explanations

a. very small number of events

TABLE S11: SUMMARY OF FINDINGS FOR KQ11

**Key Question 11: What is the benefit of ERAS in incisional hernia repair?** (Sartori A, Botteri E, Agresta F, Gerardi C, Vettoretto N, Arezzo A, et al. Should enhanced recovery after surgery (ERAS) pathways be preferred over standard practice for patients undergoing abdominal wall reconstruction? A systematic review and meta-analysis. Hernia. 2020;18:18.)

**Question:** Enhanced Recovery After Surgery (ERAS) pathways be adopted compared to standard practice for patients undergoing for Abdominal Wall Reconstruction (AWR).

| **Certainty assessment** | | | | | | | **№ of patients** | | **Effect** | | **Certainty** | **Importance** |
| --- | --- | --- | --- | --- | --- | --- | --- | --- | --- | --- | --- | --- |
| **№ of studies** | **Study design** | **Risk of bias** | **Inconsistency** | **Indirectness** | **Imprecision** | **Other considerations** | **Enhanced Recovery After Surgery (ERAS) pathways be adopted** | **standard practice for patients undergoing** | **Relative (95% CI)** | **Absolute (95% CI)** |  |  |

**Postoperative morbidity**

| 5 | observational studies | very serious^a^ | very serious^b^ | not serious | serious^c^ | all plausible residual confounding would reduce the demonstrated effect | 85/382 (22.3%) | 134/458 (29.3%) | **OR 0.73**  (0.32 to 1.63) | **61 fewer per**  **1,000**  (from 176  fewer to 110 more) | ⨁◯◯◯  Very low | CRITICAL |
| --- | --- | --- | --- | --- | --- | --- | --- | --- | --- | --- | --- | --- |

**Abdominal wall morbidity**

| 4 | observational studies | very serious^a^ | very serious^b^ | not serious | very serious^c^ | all plausible residual confounding would reduce the demonstrated effect | 44/282 (15.6%) | 64/358 (17.9%) | **OR 1.05**  (0.40 to 2.73) | **7 more per**  **1,000**  (from 99  fewer to 194 more) | ⨁◯◯◯  Very low | CRITICAL |
| --- | --- | --- | --- | --- | --- | --- | --- | --- | --- | --- | --- | --- |

**Surgical Site Infection**

| 5 | observational studies | very serious^a^ | serious^b^ | not serious | serious^c^ | all plausible residual confounding would reduce the demonstrated effect | 26/382 (6.8%) | 51/458 (11.1%) | **OR 1.17**  (0.43 to 3.22) | **17 more per**  **1,000**  (from 60  fewer to 176 more) | ⨁◯◯◯  Very low | CRITICAL |
| --- | --- | --- | --- | --- | --- | --- | --- | --- | --- | --- | --- | --- |

**Time to discontinuation of narcotics**

| 2 | observational studies | very serious^a^ | very serious^b,d^ | not serious | very serious^c^ | all plausible residual confounding would reduce the demonstrated effect | 200 | 200 | - | SMD **0.61**  **lower**  (1.81 lower to  0.59 higher) | ⨁◯◯◯  Very low | IMPORTANT |
| --- | --- | --- | --- | --- | --- | --- | --- | --- | --- | --- | --- | --- |

**Time to urinary catheter removal**

| 2 | observational studies | serious^a^ | very serious^d^ | not serious | very serious^c^ | strong association all plausible residual  confounding would reduce the demonstrated effect | 200 | 200 | - | SMD **2.77**  **lower**  (6.05 lower to  0.51 higher) | ⨁◯◯◯  Very low | IMPORTANT |
| --- | --- | --- | --- | --- | --- | --- | --- | --- | --- | --- | --- | --- |

**Time to bowel function**

| 2 | observational studies | very serious^e^ | very serious^d^ | not serious | very serious^c^ | strong association all plausible residual  confounding would reduce the demonstrated effect | 151 | 227 | - | SMD **2.57**  **lower**  (5.32 lower to  0.17 higher) | ⨁◯◯◯  Very low | IMPORTANT |
| --- | --- | --- | --- | --- | --- | --- | --- | --- | --- | --- | --- | --- |

**Time to regular diet**

| 2 | observational studies | serious^f^ | very serious^d^ | not serious | very serious^c^ | all plausible residual confounding would reduce the demonstrated effect | 200 | 200 | - | SMD **0.77**  **lower**  (2.29 lower to  0.74 higher) | ⨁◯◯◯  Very low | IMPORTANT |
| --- | --- | --- | --- | --- | --- | --- | --- | --- | --- | --- | --- | --- |

**Readmission**

| 5 | observational studies | not serious | not serious | not serious | serious^c^ | all plausible residual confounding would reduce the demonstrated effect | 38/382 (9.9%) | 57/458 (12.4%) | **OR 0.82**  (0.52 to 1.27) | **20 fewer per**  **1,000**  (from 56  fewer to 28 more) | ⨁⨁◯◯  Low | CRITICAL |
| --- | --- | --- | --- | --- | --- | --- | --- | --- | --- | --- | --- | --- |

**Length of hospital stay**

| 5 | observational studies | serious^f^ | very serious^d^ | not serious | serious^c^ | all plausible residual confounding would reduce the demonstrated effect | 382 | 458 | - | SMD **0.93**  **lower**  (1.84 lower to  0.02 lower) | ⨁◯◯◯  Very low | IMPORTANT |
| --- | --- | --- | --- | --- | --- | --- | --- | --- | --- | --- | --- | --- |

**CI:** confidence interval; **OR:** odds ratio; **SMD:** standardised mean difference

**Explanations**

1. Before/after studies: different surgical interventions at probably different points of the surgeon's learning curve.
2. Substantial clinical and methodological heterogeneity
3. Optimal information size e is not met (small sample size e 840 patients)
4. Considerable clinical and methodological heterogeneity
5. Not clear if based on flatus or stools
6. Different ward protocols at different time periods (Before/After study)

TABLE S12: SUMMARY OF FINDINGS FOR KQ12

**Key Question 12: Should prophylactic antibiotics be used in the elective repair of incisional hernia in adult patients?**

Question: Should prophylactic antibiotics be used in the elective repair of incisional hernia in adult patients?

| **Certainty assessment** | | | | | | | **№ of patients** | | **Effect** | | **Certainty** | **Importance** |
| --- | --- | --- | --- | --- | --- | --- | --- | --- | --- | --- | --- | --- |
| **№ of studies** | **Study design** | **Risk of bias** | **Inconsistency** | **Indirectness** | **Imprecision** | **Other considerations** | **antibiotics** | **no antibiotics** | **Relative (95% CI)** | **Absolute (95% CI)** |  |  |
| **Wound infection** | | | | | | | | | | | | |
| 2 | observational studies | not serious | not serious | very serious^a^ | not serious | none | 104/11704 (0.9%) | 39/2025 (1.9%) | **OR 0.62** (0.42 to 0.93) | **7 fewer per 1 000** (from 11 fewer to 1 fewer) | ⨁◯◯◯ Very low | CRITICAL |
| **Deep SSI** | | | | | | | | | | | | |
| 1 | observational studies | not serious | not serious | very serious^a^ | serious^b^ | none | 49/11564 (0.4%) | 12/1949 (0.6%) | **OR 0.69** (0.36 to 1.29) | **2 fewer per 1 000** (from 4 fewer to 2 more) | ⨁◯◯◯ Very low | CRITICAL |
| **Wound infection** | | | | | | | | | | | | |
| 1 | randomised trials | very serious^c^ | not serious | not serious | extremely serious^d^ | none | 0/8 (0.0%) | 4/8 (50.0%) | **OR 0.06** (0.00 to 1.36) | **443 fewer per 1 000** (from -- to 76 more) | ⨁◯◯◯ Very low | CRITICAL |

**CI:** confidence interval; **OR:** odds ratio

#### Explanations

a. High number of other types of hernia

b. Small number of events

c. High risk of bias

d. Extreme impression only 4 events and 16 participants

TABLE S13: SUMMARY OF FINDINGS FOR KQ13

**Key Question 13:**

**a) What information is important for patients following incisional hernia repair?**

**b) What activities influence outcome?**

**Question:** Does the use of an abdominal binder improve outcomes in incisional hernia surgery?

| **Certainty assessment** | | | | | | | **№ of patients** | | **Effect** | | **Certainty** | **Importance** |
| --- | --- | --- | --- | --- | --- | --- | --- | --- | --- | --- | --- | --- |
| **№ of studies** | **Study design** | **Risk of bias** | **Inconsistency** | **Indirectness** | **Imprecision** | **Other considerations** | **abdominal binder** | **no abdominal binder** | **Relative (95% CI)** | **Absolute (95% CI)** |  |  |
| **Readmission day 30** | | | | | | | | | | | | |
| 1 | randomised trials | not serious | not serious | very serious^a^ | very serious^b^ | none | 2/29 (6.9%) | 2/31 (6.5%) | **OR 1.07** (0.14 to 8.17) | **4 more per 1 000** (from 55 fewer to 296 more) | ⨁◯◯◯ Very low | CRITICAL |
| **Seroma presence** | | | | | | | | | | | | |
| 1 | randomised trials | not serious | not serious | very serious^a^ | very serious^b^ | none | 27/29 (93.1%) | 26/31 (83.9%) | **OR 2.60** (0.46 to 14.59) | **92 more per 1 000** (from 134 fewer to 148 more) | ⨁◯◯◯ Very low | CRITICAL |
| **Complications day 30** | | | | | | | | | | | | |
| 1 | randomised trials | not serious | not serious | very serious^a^ | very serious^b^ | none | 0/29 (0.0%) | 4/31 (12.9%) | **OR 0.10** (0.01 to 2.01) | **114 fewer per 1 000** (from 128 fewer to 100 more) | ⨁◯◯◯ Very low | CRITICAL |
| **Quality of Live day 1 (total CCS score) (follow-up: median 1 days)** | | | | | | | | | | | | |
| 1 | randomised trials | not serious | not serious | very serious^a^ | very serious^b^ | none | 29 | 31 | - | median **60 8 higher** (1 higher to 80 higher) | ⨁◯◯◯ Very low | CRITICAL |
| **Pain activity day 1 (VAS) 0-100** | | | | | | | | | | | | |
| 1 | randomised trials | not serious | not serious | very serious^a^ | very serious^b^ | none | 29 | 31 | - | MD **20 lower** (52.45 lower to 12.45 higher) | ⨁◯◯◯ Very low | IMPORTANT |
| **Return to functional activity (follow-up: median 30 days)** | | | | | | | | | | | | |
| 1 | randomised trials | not serious | not serious | very serious^a^ | very serious^b^ | none | 29 | 31 | - | MD **15 lower** (49.68 lower to 19.68 higher) | ⨁◯◯◯ Very low | IMPORTANT |
| **Return to physical activity or work/lifting after IHS and risk of IHS reccurence.** | | | | | | | | | | | | |
| 2 | observational studies | very serious^c^ | not serious | very serious^a^ | very serious^d^ | none | In the literature there is a the lack of evidence on this important issue [2,3]. Early mobilization immediately after surgery is suggested, while there are insufficient data to justify a recommendation of reduced strain or activity after uncomplicated open incisional hernia mesh repair for more than 4 weeks [2,3]. | | | | ⨁◯◯◯ Very low | CRITICAL |

**CI:** confidence interval; **MD:** mean difference; **OR:** odds ratio

#### Explanations

a. Combination of primary or recurrent (incisional) umbilical or epigastric hernias

b. Just a single study with a very small sample size

c. High risk of bias of different study designs

d. Small sample sizes of different study desig
