# Supplementary material for: Midline incisional hernia guidelines: the European Hernia Society
Source: Br J Surg. 2023 Sep 19;110(12):1732–68. doi: 10.1093/bjs/znad284 (PMC10638550; doi:10.1093/bjs/znad284)
Supplement: znad284_Supplementary_Data [file znad284_supplementary_data.zip › TABLE S1.docx]

**TABLE S1: SEARCH STRATEGIES**

**A. Guideline search**

The search was conducted in guideline databases, repositories or on the guideline developer’s website on July 24, 2020, using keywords hernia, hernias, and their equivalents in other languages.

**Databases, repositories and websites searched**

| **Name** | **Website** |
| --- | --- |
| **Academy of Medicine of Malaysia** | <http://www.acadmed.org.my/index.cfm?menuid=67> |
| **American Hernia Society** | https:/[/www](http://www.americanherniasociety.org/).[americanherniasociety.org/](http://www.americanherniasociety.org/) |
| **Arbeitsgemeinschaft der**  **Wissenschaftlichen Medizinischen Fachgesellschaften (AWMF)** | https:/[/www](http://www.awmf.org/awmf-online-das-portal-der-).[awmf.org/awmf-online-das-portal-der-](http://www.awmf.org/awmf-online-das-portal-der-) wissenschaftlichen-medizin/awmf-aktuell.html |
| **Australian Clinical Practice**  **Guidelines** | https:/[/www](http://www.clinicalguidelines.gov.au/).[clinicalguidelines.gov.au/](http://www.clinicalguidelines.gov.au/) |
| **Belgian Health Care Knowledge**  **Centre** | https://kce.login.kanooh.be/en/publications/all-reports |
| **Biblioteca de Guías de Práctica Clínica del Sistema Nacional de**  **Salud (GuíaSalud)** | <http://portal.guiasalud.es/web/guest/guias-practica-clinica> |
| **BIGG international database of GRADE guidelinesBIGG base**  **internacional de guías GRADE** | <http://sites.bvsalud.org/bigg/biblio/> |
| **British Columbia Guidelines** | https://www2.gov.bc.ca/gov/content/health/practitioner- professional-resources/bc-guidelines/guidelines-by-  alphabetical-listing#D |
| **British Hernia Society** | https:/[/www](http://www.britishherniasociety.org/).[britishherniasociety.org/](http://www.britishherniasociety.org/) |
| **CPG Infobase: Clinical Practice**  **Guidelines** | https://joulecma.ca/cpg/homepage |
| **Domus medica Belgium** | https://domusmedica.be/richtlijnen |
| **Dynamed** | https:/[/www](http://www.dynamed.com/home/).[dynamed.com/home/](http://www.dynamed.com/home/) |
| **ECRI Institute Guideline Trust** | https://guidelines.ecri.org/ |
| **Epistemonikos GRADE guideline**  **repository** | https:/[/www](http://www.epistemonikos.org/en/groups/grade_guideline).[epistemonikos.org/en/groups/grade_guideline](http://www.epistemonikos.org/en/groups/grade_guideline) |
| **European Hernia Society** | https:/[/www](http://www.europeanherniasociety.eu/).[europeanherniasociety.eu/](http://www.europeanherniasociety.eu/) |
| **Guías de Práctica Clínica** | https://guidelines.international/gpc |
| **Guideline Central** | https:/[/www](http://www.guidelinecentral.com/).[guidelinecentral.com/](http://www.guidelinecentral.com/) |
| **Health Quality Ontario, Canada** | https:/[/www](http://www.hqontario.ca/Evidence-to-Improve-).[hqontario.ca/Evidence-to-Improve-](http://www.hqontario.ca/Evidence-to-Improve-) Care/Health-Technology-Assessment/Reviews-And-  Recommendations |
| **Institute for Clinical Systems**  **Improvement (ICSI)** | https:/[/www](http://www.icsi.org/guidelines/).[icsi.org/guidelines/](http://www.icsi.org/guidelines/) |
| **International HTA Database** | https://database.inahta.org/ |
| **MAGICapp** | https://app.magicapp.org/app#/guidelines |
| **MaHTAS – Health Technology**  **Assessment Section, Ministry of Health Malaysia** | <http://www.moh.gov.my/index.php/pages/view/135?mid=6> 7 |
| **Ministério da Sáudé, Brazil** | <http://www.saude.gov.br/protocolos-e-diretrizes> |
| **Ministerio de Salud, Chile** | <https://diprece.minsal.cl/programas-de-salud/guias-clinicas/> |
| **Ministerio de Salud, Columbia** | <http://gpc.minsalud.gov.co/gpc/SitePages/buscador_gpc.as>  px |
| **Ministerio de Salud, Peru** | https://web.ins.gob.pe/salud-publica/publicaciones-  unagesp/guias-de-practica-clinica |
| **National Institute for Clinical**  **Evidence (NICE)** | https:/[/www](http://www.nice.org.uk/guidance).[nice.org.uk/guidance](http://www.nice.org.uk/guidance) |
| **National Patient Safety Office,**  **Ireland** | https://health.gov.ie/national-patient-safety-  office/ncec/national-clinical-guidelines/ |
| **Nederland Huisartsen**  **Genootschap** | https:/[/www](http://www.nhg.org/nhg-standaarden).[nhg.org/nhg-standaarden](http://www.nhg.org/nhg-standaarden) |
| **NIPH – Norwegian Institute of**  **Public Health** | https:/[/www](http://www.fhi.no/en/qk/HTA/).[fhi.no/en/qk/HTA/](http://www.fhi.no/en/qk/HTA/) |
| **OSTEBA – Basque Office for Health**  **Technology Assessment** | <http://www.euskadi.eus/information/clinical-practice->  guidelines/web01-a3ikeost/en/ |
| **Scottish Intercollegiate Guidelines**  **Network (SIGN)** | https:/[/www](http://www.sign.ac.uk/).[sign.ac.uk/](http://www.sign.ac.uk/) |
| **The Canadian Task Force on**  **Preventive Health Care** | https://canadiantaskforce.ca/guidelines/published-  guidelines/ |
| **The GIN international guideline**  **libraryGuidelines International Network (G-I-N)** | https://g-i-n.net/international-guidelines- library<http://www.g-i-n.net/> |
| **Universidad Nacional de Colombia, Guías de Practica**  **Clínica** | https://unisalud.unal.edu.co/guias-practicas-clinica.html |
| **Washington State Health Care**  **Authority** | https:/[/www](http://www.hca.wa.gov/about-hca/health-technology-).[hca.wa.gov/about-hca/health-technology-](http://www.hca.wa.gov/about-hca/health-technology-)  assessment |
| **WHO** | https:/[/www](http://www.who.int/publications/guidelines/en/).[who.int/publications/guidelines/en/](http://www.who.int/publications/guidelines/en/) |
