# Supplementary material for: Midline incisional hernia guidelines: the European Hernia Society
Source: Br J Surg. 2023 Sep 19;110(12):1732–68. doi: 10.1093/bjs/znad284 (PMC10638550; doi:10.1093/bjs/znad284)
Supplement: znad284_Supplementary_Data [file znad284_supplementary_data.zip › TABLE S14.docx]

**TABLE S14: SUMMARY OF FINDINGS FOR KQ13**

# Key Question 13:

1. **What information is important for patients following incisional hernia repair?**
2. **What activities influence outcome?**

**Question:** Does the use of an abdominal binder improve outcomes in incisional hernia surgery?

| **Certainty assessment** | | | | | | | **№ of patients** | | **Effect** | | **Certainty** | **Importance** |
| --- | --- | --- | --- | --- | --- | --- | --- | --- | --- | --- | --- | --- |
| **№ of studies** | **Study design** | **Risk of bias** | **Inconsistency** | **Indirectness** | **Imprecision** | **Other considerations** | **abdominal binder** | **no abdominal binder** | **Relative (95% CI)** | **Absolute (95% CI)** |  |  |

**Readmission day 30**

| 1 | randomised trials | not serious | not serious | very seriousa | very seriousb | none | 2/29 (6.9%) | 2/31 (6.5%) | **OR 1.07**  (0.14 to 8.17) | **4 more per 1 000**  (from 55 fewer to 296 more) | ⨁◯◯◯  Very low | CRITICAL |
| --- | --- | --- | --- | --- | --- | --- | --- | --- | --- | --- | --- | --- |

**Seroma presence**

| 1 | randomised trials | not serious | not serious | very seriousa | very seriousb | none | 27/29 (93.1%) | 26/31 (83.9%) | **OR 2.60**  (0.46 to 14.59) | **92 more per 1 000**  (from 134 fewer  to 148 more) | ⨁◯◯◯  Very low | CRITICAL |
| --- | --- | --- | --- | --- | --- | --- | --- | --- | --- | --- | --- | --- |

**Complications day 30**

| 1 | randomised trials | not serious | not serious | very seriousa | very seriousb | none | 0/29 (0.0%) | 4/31 (12.9%) | **OR 0.10**  (0.01 to 2.01) | **114 fewer per 1 000**  (from 128 fewer  to 100 more) | ⨁◯◯◯  Very low | CRITICAL |
| --- | --- | --- | --- | --- | --- | --- | --- | --- | --- | --- | --- | --- |

**Quality of Live day 1 (total CCS score) (follow-up: median 1 days)**

| 1 | randomised trials | not serious | not serious | very seriousa | very seriousb | none | 29 | 31 | - | median **60 8 higher**  (1 higher to 80 higher) | ⨁◯◯◯  Very low | CRITICAL |
| --- | --- | --- | --- | --- | --- | --- | --- | --- | --- | --- | --- | --- |

**Pain activity day 1 (VAS) 0-100**

| 1 | randomised trials | not serious | not serious | very seriousa | very seriousb | none | 29 | 31 | - | MD **20 lower**  (52.45 lower to  12.45 higher) | ⨁◯◯◯  Very low | IMPORTANT |
| --- | --- | --- | --- | --- | --- | --- | --- | --- | --- | --- | --- | --- |

**Return to functional activity (follow-up: median 30 days)**

| 1 | randomised trials | not serious | not serious | very seriousa | very seriousb | none | 29 | 31 | - | MD **15 lower**  (49.68 lower to  19.68 higher) | ⨁◯◯◯  Very low | IMPORTANT |
| --- | --- | --- | --- | --- | --- | --- | --- | --- | --- | --- | --- | --- |

**Return to physical activity or work/lifting after IHS and risk of IHS reccurence.**

| **Certainty assessment** | | | | | | | **№ of patients** | | **Effect** | | **Certainty** | **Importance** |
| --- | --- | --- | --- | --- | --- | --- | --- | --- | --- | --- | --- | --- |
| **№ of studies** | **Study design** | **Risk of bias** | **Inconsistency** | **Indirectness** | **Imprecision** | **Other considerations** | **abdominal binder** | **no abdominal binder** | **Relative (95% CI)** | **Absolute (95% CI)** |  |  |
| 2 | observational studies | very seriousc | not serious | very seriousa | very seriousd | none | In the literature there is a the lack of evidence on this important issue [2,3]. Early mobilization immediately after surgery is suggested, while there are insufficient data to justify a recommendation of reduced strain or activity after uncomplicated open incisional hernia mesh repair for more than 4 weeks [2,3]. | | | | ⨁◯◯◯  Very low | CRITICAL |

**CI:** confidence interval; **MD:** mean difference; **OR:** odds ratio

**Explanations**

1. Combination of primary or recurrent (incisional) umbilical or epigastric hernias

b. Just a single study with a very small sample size

c. High risk of bias of different study designs

d. Small sample sizes of different study designs
