# Supplementary material for: Midline incisional hernia guidelines: the European Hernia Society
Source: Br J Surg. 2023 Sep 19;110(12):1732–68. doi: 10.1093/bjs/znad284 (PMC10638550; doi:10.1093/bjs/znad284)
Supplement: znad284_Supplementary_Data [file znad284_supplementary_data.zip › Table_S10.docx]

**TABLE S11: SUMMARY OF FINDINGS FOR KQ10**

**Key Question 10: What is the difference in the outcome using different techniques for mesh fixation in:**

**(a) intraperitoneal and**

**(b) extraperitoneal mesh placement for incisional hernia repair?**

Question: Absorbable tacks compared to non-absorbable tacks for mesh fixation in minimally invasive elective repair of incisional hernia in adult patients

| **Certainty assessment** | | | | | | | **№ of patients** | | **Effect** | | **Certainty** | **Importance** |
| --- | --- | --- | --- | --- | --- | --- | --- | --- | --- | --- | --- | --- |
| **№ of studies** | **Study design** | **Risk of bias** | **Inconsistency** | **Indirectness** | **Imprecision** | **Other considerations** | **absorbable tacks** | **non absorbable tack** | **Relative (95% CI)** | **Absolute (95% CI)** |  |  |
| **Seroma** | | | | | | | | | | | | |
| 2 | randomised trials | not serious | not serious | not serious | extremely serious | none | 6/71 (8.5%) | 8/70 (11.4%) | **OR 0.70** (0.22 to 2.17) | **31 fewer per 1 000** (from 87 fewer to 104 more) | ⨁◯◯◯ Very low | CRITICAL |
| **Length of stay** | | | | | | | | | | | | |
| 2 | randomised trials | not serious | not serious | not serious | extremely serious | none | 71 | 70 | - | MD **0.01 higher** (0.63 lower to 0.64 higher) | ⨁◯◯◯ Very low | CRITICAL |
| **QoL (WHO BREF)** | | | | | | | | | | | | |
| 1 | randomised trials | not serious | not serious | not serious | extremely serious | none | 45 | 45 | - | MD **0.1 higher** (0.15 lower to 0.35 higher) | ⨁◯◯◯ Very low | CRITICAL |
| **Recurrence** | | | | | | | | | | | | |
| 3 | randomised trials | not serious | not serious | not serious | extremely serious | none | 3/96 (3.1%) | 4/95 (4.2%) | **OR 0.72** (0.15 to 3.38) | **11 fewer per 1 000** (from 36 fewer to 87 more) | ⨁◯◯◯ Very low | CRITICAL |

**CI:** confidence interval; **MD:** mean difference; **OR:** odds ratio

#### Explanations

a. Only two small studies with very small number of events

b. Only two small studies with not overlapping CI

c. Only one very small study

d. Only three very small studies with very small number of events

Question: Glue compared to tacks for mesh fixation in minimally invasive elective repair of incisional hernia in adult patients

| **Certainty assessment** | | | | | | | **№ of patients** | | **Effect** | | **Certainty** | **Importance** |
| --- | --- | --- | --- | --- | --- | --- | --- | --- | --- | --- | --- | --- |
| **№ of studies** | **Study design** | **Risk of bias** | **Inconsistency** | **Indirectness** | **Imprecision** | **Other considerations** | **glue fixation** | **tack fixation** | **Relative (95% CI)** | **Absolute (95% CI)** |  |  |
| **Pain (Caroline scale)** | | | | | | | | | | | | |
| 1 | randomised trials | not serious | not serious | not serious | extremely serious | none | 5/25 (20.0%) | 7/50 (14.0%) | **OR 1.54** (0.43 to 5.44) | **60 more per 1 000** (from 75 fewer to 330 more) | ⨁◯◯◯ Very low | CRITICAL |
| **Recurrence** | | | | | | | | | | | | |
| 1 | randomised trials | not serious | not serious | not serious | extremely serious | none | 0/25 (0.0%) | 3/50 (6.0%) | **OR 0.27** (0.01 to 5.36) | **43 fewer per 1 000** (from 59 fewer to 195 more) | ⨁◯◯◯ Very low | CRITICAL |

**CI:** confidence interval; **OR:** odds ratio

#### Explanations

a. very small number of events
