# Supplementary material for: Midline incisional hernia guidelines: the European Hernia Society
Source: Br J Surg. 2023 Sep 19;110(12):1732–68. doi: 10.1093/bjs/znad284 (PMC10638550; doi:10.1093/bjs/znad284)
Supplement: znad284_Supplementary_Data [file znad284_supplementary_data.zip › Table_S11.docx]

**TABLE S12: SUMMARY OF FINDINGS FOR KQ11**

**Key Question 11: What is the benefit of ERAS in incisional hernia repair?** (Sartori A, Botteri E, Agresta F, Gerardi C, Vettoretto N, Arezzo A, et al. Should enhanced recovery after surgery (ERAS) pathways be preferred over standard practice for patients undergoing abdominal wall reconstruction? A systematic review and meta-analysis. Hernia. 2020;18:18.)

**Question:** Enhanced Recovery After Surgery (ERAS) pathways be adopted compared to standard practice for patients undergoing for Abdominal Wall Reconstruction (AWR).

| **Certainty assessment** | | | | | | | **№ of patients** | | **Effect** | | **Certainty** | **Importance** |
| --- | --- | --- | --- | --- | --- | --- | --- | --- | --- | --- | --- | --- |
| **№ of studies** | **Study design** | **Risk of bias** | **Inconsistency** | **Indirectness** | **Imprecision** | **Other considerations** | **Enhanced Recovery After Surgery (ERAS) pathways be adopted** | **standard practice for patients undergoing** | **Relative (95% CI)** | **Absolute (95% CI)** |  |  |

**Postoperative morbidity**

| 5 | observational studies | very serious^a^ | very serious^b^ | not serious | serious^c^ | all plausible residual confounding would reduce the demonstrated effect | 85/382 (22.3%) | 134/458 (29.3%) | **OR 0.73**  (0.32 to 1.63) | **61 fewer per**  **1,000**  (from 176  fewer to 110 more) | ⨁◯◯◯  Very low | CRITICAL |
| --- | --- | --- | --- | --- | --- | --- | --- | --- | --- | --- | --- | --- |

**Abdominal wall morbidity**

| 4 | observational studies | very serious^a^ | very serious^b^ | not serious | very serious^c^ | all plausible residual confounding would reduce the demonstrated effect | 44/282 (15.6%) | 64/358 (17.9%) | **OR 1.05**  (0.40 to 2.73) | **7 more per**  **1,000**  (from 99  fewer to 194 more) | ⨁◯◯◯  Very low | CRITICAL |
| --- | --- | --- | --- | --- | --- | --- | --- | --- | --- | --- | --- | --- |

**Surgical Site Infection**

| 5 | observational studies | very serious^a^ | serious^b^ | not serious | serious^c^ | all plausible residual confounding would reduce the demonstrated effect | 26/382 (6.8%) | 51/458 (11.1%) | **OR 1.17**  (0.43 to 3.22) | **17 more per**  **1,000**  (from 60  fewer to 176 more) | ⨁◯◯◯  Very low | CRITICAL |
| --- | --- | --- | --- | --- | --- | --- | --- | --- | --- | --- | --- | --- |

**Time to discontinuation of narcotics**

| 2 | observational studies | very serious^a^ | very serious^b,d^ | not serious | very serious^c^ | all plausible residual confounding would reduce the demonstrated effect | 200 | 200 | - | SMD **0.61**  **lower**  (1.81 lower to  0.59 higher) | ⨁◯◯◯  Very low | IMPORTANT |
| --- | --- | --- | --- | --- | --- | --- | --- | --- | --- | --- | --- | --- |

**Time to urinary catheter removal**

| 2 | observational studies | serious^a^ | very serious^d^ | not serious | very serious^c^ | strong association all plausible residual  confounding would reduce the demonstrated effect | 200 | 200 | - | SMD **2.77**  **lower**  (6.05 lower to  0.51 higher) | ⨁◯◯◯  Very low | IMPORTANT |
| --- | --- | --- | --- | --- | --- | --- | --- | --- | --- | --- | --- | --- |

**Time to bowel function**

| 2 | observational studies | very serious^e^ | very serious^d^ | not serious | very serious^c^ | strong association all plausible residual  confounding would reduce the demonstrated effect | 151 | 227 | - | SMD **2.57**  **lower**  (5.32 lower to  0.17 higher) | ⨁◯◯◯  Very low | IMPORTANT |
| --- | --- | --- | --- | --- | --- | --- | --- | --- | --- | --- | --- | --- |

**Time to regular diet**

| 2 | observational studies | serious^f^ | very serious^d^ | not serious | very serious^c^ | all plausible residual confounding would reduce the demonstrated effect | 200 | 200 | - | SMD **0.77**  **lower**  (2.29 lower to  0.74 higher) | ⨁◯◯◯  Very low | IMPORTANT |
| --- | --- | --- | --- | --- | --- | --- | --- | --- | --- | --- | --- | --- |

**Readmission**

| 5 | observational studies | not serious | not serious | not serious | serious^c^ | all plausible residual confounding would reduce the demonstrated effect | 38/382 (9.9%) | 57/458 (12.4%) | **OR 0.82**  (0.52 to 1.27) | **20 fewer per**  **1,000**  (from 56  fewer to 28 more) | ⨁⨁◯◯  Low | CRITICAL |
| --- | --- | --- | --- | --- | --- | --- | --- | --- | --- | --- | --- | --- |

**Length of hospital stay**

| 5 | observational studies | serious^f^ | very serious^d^ | not serious | serious^c^ | all plausible residual confounding would reduce the demonstrated effect | 382 | 458 | - | SMD **0.93**  **lower**  (1.84 lower to  0.02 lower) | ⨁◯◯◯  Very low | IMPORTANT |
| --- | --- | --- | --- | --- | --- | --- | --- | --- | --- | --- | --- | --- |

**CI:** confidence interval; **OR:** odds ratio; **SMD:** standardised mean difference

**Explanations**

1. Before/after studies: different surgical interventions at probably different points of the surgeon's learning curve.
2. Substantial clinical and methodological heterogeneity
3. Optimal information size e is not met (small sample size e 840 patients)
4. Considerable clinical and methodological heterogeneity
5. Not clear if based on flatus or stools
6. Different ward protocols at different time periods (Before/After study)
