# Supplementary material for: Midline incisional hernia guidelines: the European Hernia Society
Source: Br J Surg. 2023 Sep 19;110(12):1732–68. doi: 10.1093/bjs/znad284 (PMC10638550; doi:10.1093/bjs/znad284)
Supplement: znad284_Supplementary_Data [file znad284_supplementary_data.zip › Table_S12.docx]

**TABLE S13: SUMMARY OF FINDINGS FOR KQ12**

**Key Question 12: Should prophylactic antibiotics be used in the elective repair of incisional hernia in adult patients?**

Question: Should prophylactic antibiotics be used in the elective repair of incisional hernia in adult patients?

| **Certainty assessment** | | | | | | | **№ of patients** | | **Effect** | | **Certainty** | **Importance** |
| --- | --- | --- | --- | --- | --- | --- | --- | --- | --- | --- | --- | --- |
| **№ of studies** | **Study design** | **Risk of bias** | **Inconsistency** | **Indirectness** | **Imprecision** | **Other considerations** | **antibiotics** | **no antibiotics** | **Relative (95% CI)** | **Absolute (95% CI)** |  |  |
| **Wound infection** | | | | | | | | | | | | |
| 2 | observational studies | not serious | not serious | very serious^a^ | not serious | none | 104/11704 (0.9%) | 39/2025 (1.9%) | **OR 0.62** (0.42 to 0.93) | **7 fewer per 1 000** (from 11 fewer to 1 fewer) | ⨁◯◯◯ Very low | CRITICAL |
| **Deep SSI** | | | | | | | | | | | | |
| 1 | observational studies | not serious | not serious | very serious^a^ | serious^b^ | none | 49/11564 (0.4%) | 12/1949 (0.6%) | **OR 0.69** (0.36 to 1.29) | **2 fewer per 1 000** (from 4 fewer to 2 more) | ⨁◯◯◯ Very low | CRITICAL |
| **Wound infection** | | | | | | | | | | | | |
| 1 | randomised trials | very serious^c^ | not serious | not serious | extremely serious^d^ | none | 0/8 (0.0%) | 4/8 (50.0%) | **OR 0.06** (0.00 to 1.36) | **443 fewer per 1 000** (from -- to 76 more) | ⨁◯◯◯ Very low | CRITICAL |

**CI:** confidence interval; **OR:** odds ratio

#### Explanations

a. High number of other types of hernia

b. Small number of events

c. High risk of bias

d. Extreme impression only 4 events and 16 participants
