# Supplementary material for: Midline incisional hernia guidelines: the European Hernia Society
Source: Br J Surg. 2023 Sep 19;110(12):1732–68. doi: 10.1093/bjs/znad284 (PMC10638550; doi:10.1093/bjs/znad284)
Supplement: znad284_Supplementary_Data [file znad284_supplementary_data.zip › Table_S2.docx]

**TABLE S2 SUMMARY OF FINDINGS FOR KQ1**

**Key Question 1: What are the risk factors for developing an incisional hernia after previous abdominal surgery?**

| **Certainty assessment** | | | | | | | **№ of patients** | | **Effect** | | **Certainty** | **Importance** |
| --- | --- | --- | --- | --- | --- | --- | --- | --- | --- | --- | --- | --- |
| **№ of studies** | **Study design** | **Risk of bias** | **Inconsistency** | **Indirectness** | **Imprecision** | **Other considerations** | **exposure to a specific (risk) factor** | **no exposure to that specific (risk) factor** | **Relative (95% CI)** | **Absolute (95% CI)** |  |  |
| **Risk of incisional hernia for type of previous incision (follow up median 30 m) Offline vs Middline (follow up: median 30 months)** | | | | | | | | | | | | |
| 13 | randomised trials | very serious^a^ | not serious | not serious | not serious | none | 65/1240 (5.2%) | 106/1058 (10.0%) | **RR 0.47** (0.30 to 0.75) | **53 fewer per 1,000** (from 70 fewer to 25 fewer) | ⨁⨁◯◯ Low | CRITICAL |
| **SILS vs CLS on hernia occurance** | | | | | | | | | | | | |
| 32 | randomised trials | not serious | not serious | not serious | very serious^b^ | none | 27/1861 (1.5%) | 11/2156 (0.5%) | **OR 1.92** (0.94 to 3.91) | **5 more per 1,000** (from 0 fewer to 15 more) | ⨁⨁◯◯ Low | IMPORTANT |
| **Gender as risk to IH (Male vvs Female)** | | | | | | | | | | | | |
| 10 | observational studies | not serious | not serious | not serious | not serious | none | 214/2539 (8.4%) | 196/2618 (7.5%) | **OR 1.14** (0.90 to 1.45) | **10 more per 1,000** (from 7 fewer to 30 more) | ⨁⨁◯◯ Low | IMPORTANT |
| **Risk of IH with diabetes (diabetes vs no diabetes)** | | | | | | | | | | | | |
| 7 | observational studies | not serious | not serious | not serious | not serious | none | 69/471 (14.6%) | 272/3109 (8.7%) | **OR 1.73** (1.30 to 2.32) | **55 more per 1,000** (from 23 more to 94 more) | ⨁⨁◯◯ Low | CRITICAL |
| **Risk with immunosuppression (Immuno vs no)** | | | | | | | | | | | | |
| 4 | observational studies | not serious | not serious | serious^c^ | not serious | none | 73/700 (10.4%) | 156/1998 (7.8%) | **OR 1.75** (1.28 to 2.38) | **51 more per 1,000** (from 20 more to 90 more) | ⨁◯◯◯ Very low | CRITICAL |
| **Risk smoking (current)** | | | | | | | | | | | | |
| 4 | observational studies | not serious | very serious^d^ | not serious | serious^e^ | none | 111/617 (18.0%) | 169/2181 (7.7%) | **OR 1.87** (1.36 to 2.57) | **58 more per 1,000** (from 25 more to 100 more) | ⨁◯◯◯ Very low | CRITICAL |
| **Risk SSI (SSI vs NO SSI)** | | | | | | | | | | | | |
| 9 | observational studies | not serious | not serious | not serious | not serious | strong association | 76/391 (19.4%) | 315/4542 (6.9%) | **OR 3.38** (2.18 to 5.23) | **132 more per 1,000** (from 70 more to 211 more) | ⨁⨁⨁◯ Moderate | CRITICAL |
| **BMI Underweight <18.5 kg/m2OR (95% CI)** | | | | | | | | | | | | |
| 1 | observational studies | not serious | not serious | not serious | not serious | none | 0/3580 (0.0%) | 0/736726 (0.0%) | **OR 0.92** (0.55 to 1.29) | **0 fewer per 1,000** (from 0 fewer to 0 fewer) | ⨁⨁◯◯ Low | CRITICAL |
| **Overweight25.0-29.9 kg/m2OR (95% CI)** | | | | | | | | | | | | |
| 1 | observational studies | not serious | not serious | not serious | not serious | none | 0/0 | 0/0 | **OR 1.65** (1.56 to 1.76) | **2 fewer per 1,000** (from 2 fewer to 2 fewer) | ⨁⨁◯◯ Low | CRITICAL |
| **Obesity Class 1 30.0-34.9 kg/m2OR (95% CI)** | | | | | | | | | | | | |
| 1 | observational studies | not serious | not serious | not serious | not serious | none | 0/0 | 0/0 | **OR 2.51** (2.40 to 2.62) | **3 fewer per 1,000** (from 3 fewer to 2 fewer) | ⨁⨁◯◯ Low | CRITICAL |
| **Obesity Class 2 35.0-39.9 kg/m2OR (95% CI)** | | | | | | | | | | | | |
| 1 | observational studies | not serious | not serious | not serious | not serious | none | 0/0 | 0/0 | **OR 3.62** (3.51 to 3.75) | **4 fewer per 1,000** (from 4 fewer to 4 fewer) | ⨁⨁◯◯ Low | CRITICAL |
| **Obesity Class 3 ≥40.0 kg/m2OR (95% CI)** | | | | | | | | | | | | |
| 1 | observational studies | not serious | not serious | not serious | not serious | none | 0/0 | 0/0 | **OR 5.48** (5.36 to 5.60) | **5 fewer per 1,000** (from 6 fewer to 5 fewer) | ⨁⨁◯◯ Low | CRITICAL |

**CI:** confidence interval; **OR:** odds ratio; **RR:** risk ratio

#### Explanations

a. Unclear risk of bias for most included studies

b. Wide CI and only 38 events total

c. Different exposure (different drugs as immunosuppression)

d. Confidence intervals are not overlaping, I2 is 86%

e. CI of included studies is wide
