# Supplementary material for: Midline incisional hernia guidelines: the European Hernia Society
Source: Br J Surg. 2023 Sep 19;110(12):1732–68. doi: 10.1093/bjs/znad284 (PMC10638550; doi:10.1093/bjs/znad284)
Supplement: znad284_Supplementary_Data [file znad284_supplementary_data.zip › Table_S3.docx]

**TABLE S3 SUMMARY OF FINDINGS FOR KQ2**

**Key Question 2:**

**a) Do all patients with an incisional hernia require imaging? b) What is the best modality?**

a: Summary of findings table for sub question - Abdominal Ultrasound (US) Vs Physical examination

| Outcome | № of studies (№ of patients) | Study design | Factors that may decrease certainty of evidence | | | | | Effect per 1 000 patients tested | | | | | | Test accuracy CoE |
| --- | --- | --- | --- | --- | --- | --- | --- | --- | --- | --- | --- | --- | --- | --- |
|  |  |  |  |  |  |  |  | pre-test probability of17.9% | | pre-test probability of13.6% | | pre-test probability of28.9% | |  |
|  |  |  | Risk of bias | Indirectness | Inconsistency | Imprecision | Publication bias | abdominal ultrasound scanning | physical examination of the abdomen only | abdominal ultrasound scanning | physical examination of the abdomen only | abdominal ultrasound scanning | physical examination of the abdomen only |  |
| **True positives** (patients with incisional hernia ) | 3 studies 832 patients | cross-sectional (cohort type accuracy study) | serious^a^ | not serious | serious^b^ | not serious | none^c^ | 136 to 179 | 0 to 0 | 103 to 136 | 0 to 0 | 220 to 289 | 0 to 0 | ⨁⨁◯◯ Low |
|  |  |  |  |  |  |  |  | **136 more to 179 more TP in abdominal ultrasound scanning** | | **103 more to 136 more TP in abdominal ultrasound scanning** | | **220 more to 289 more TP in abdominal ultrasound scanning** | |  |
| **False negatives** (patients incorrectly classified as not having incisional hernia ) |  |  |  |  |  |  |  | 0 to 43 | 179 to 179 | 0 to 33 | 136 to 136 | 0 to 69 | 289 to 289 |  |
|  |  |  |  |  |  |  |  | **136 fewer to 179 fewer FN in abdominal ultrasound scanning** | | **103 fewer to 136 fewer FN in abdominal ultrasound scanning** | | **220 fewer to 289 fewer FN in abdominal ultrasound scanning** | |  |
| **True negatives** (patients without incisional hernia ) | 3 studies 832 patients | cross-sectional (cohort type accuracy study) | serious^a^ | not serious | serious^b^ | not serious | none^c^ | 361 to 772 | 0 to 0 | 380 to 812 | 0 to 0 | 313 to 668 | 0 to 0 | ⨁⨁◯◯ Low |
|  |  |  |  |  |  |  |  | **361 more to 772 more TN in abdominal ultrasound scanning** | | **380 more to 812 more TN in abdominal ultrasound scanning** | | **313 more to 668 more TN in abdominal ultrasound scanning** | |  |
| **False positives** (patients incorrectly classified as having incisional hernia ) |  |  |  |  |  |  |  | 49 to 460 | 821 to 821 | 52 to 484 | 864 to 864 | 43 to 398 | 711 to 711 |  |
|  |  |  |  |  |  |  |  | **361 fewer to 772 fewer FP in abdominal ultrasound scanning** | | **380 fewer to 812 fewer FP in abdominal ultrasound scanning** | | **313 fewer to 668 fewer FP in abdominal ultrasound scanning** | |  |

#### **Explanations**

a. Index and reference tests were problematic in all three studies

b. CIs are not overlaping

c. Studies identified in one SR with not exhaustive search strategy.

b: Summary of findings table for sub question - Abdominal Computed tomography (CT) Vs Physical examination

| Outcome | № of studies (№ of patients) | Study design | Factors that may decrease certainty of evidence | | | | | Effect per 1 000 patients tested | | | | | | Test accuracy CoE |
| --- | --- | --- | --- | --- | --- | --- | --- | --- | --- | --- | --- | --- | --- | --- |
|  |  |  |  |  |  |  |  | pre-test probability of44.2% | | pre-test probability of18% | | pre-test probability of30% | |  |
|  |  |  | Risk of bias | Indirectness | Inconsistency | Imprecision | Publication bias | Medical imaging | physical examination of the abdomen only | Medical imaging | physical examination of the abdomen only | Medical imaging | physical examination of the abdomen only |  |
| **True positives** (patients with incisional hernia) | 4 studies 770 patients | cross-sectional (cohort type accuracy study) | serious^a^ | not serious | serious^b^ | not serious | none^c^ | 265 to 420 | 0 to 0 | 108 to 171 | 0 to 0 | 180 to 285 | 0 to 0 | ⨁⨁◯◯ Low |
|  |  |  |  |  |  |  |  | **265 more to 420 more TP in Medical imaging** | | **108 more to 171 more TP in Medical imaging** | | **180 more to 285 more TP in Medical imaging** | |  |
| **False negatives** (patients incorrectly classified as not having incisional hernia) |  |  |  |  |  |  |  | 22 to 177 | 442 to 442 | 9 to 72 | 180 to 180 | 15 to 120 | 300 to 300 |  |
|  |  |  |  |  |  |  |  | **265 fewer to 420 fewer FN in Medical imaging** | | **108 fewer to 171 fewer FN in Medical imaging** | | **180 fewer to 285 fewer FN in Medical imaging** | |  |
| **True negatives** (patients without incisional hernia) | 4 studies 770 patients | cross-sectional (cohort type accuracy study) | serious^a^ | not serious | serious^b^ | not serious | none | 335 to 536 | 0 to 0 | 492 to 787 | 0 to 0 | 420 to 672 | 0 to 0 | ⨁⨁◯◯ Low |
|  |  |  |  |  |  |  |  | **335 more to 536 more TN in Medical imaging** | | **492 more to 787 more TN in Medical imaging** | | **420 more to 672 more TN in Medical imaging** | |  |
| **False positives** (patients incorrectly classified as having incisional hernia) |  |  |  |  |  |  |  | 22 to 223 | 558 to 558 | 33 to 328 | 820 to 820 | 28 to 280 | 700 to 700 |  |
|  |  |  |  |  |  |  |  | **335 fewer to 536 fewer FP in Medical imaging** | | **492 fewer to 787 fewer FP in Medical imaging** | | **420 fewer to 672 fewer FP in Medical imaging** | |  |

#### **Explanations**

a. 3 of 4 studies has an issue in the patient selection, flow and timing, two with index test interpretation domains

b. Confidence intervals are not ideally overlapping nor do sensitivity and specificity across the studies

c. Studies were retrieved from systematic review with not extensive search strategy

c: Summary of findings table for sub question- Ultrasound Vs CT

| Outcome | № of studies (№ of patients) | Study design | Factors that may decrease certainty of evidence | | | | | Effect per 1 000 patients tested | | | | | | Test accuracy CoE |
| --- | --- | --- | --- | --- | --- | --- | --- | --- | --- | --- | --- | --- | --- | --- |
|  |  |  |  |  |  |  |  | pre-test probability of60% | | pre-test probability of54.7% | | pre-test probability of0% | |  |
|  |  |  | Risk of bias | Indirectness | Inconsistency | Imprecision | Publication bias | abdominal ultrasound scanning | computed tomography scanning | abdominal ultrasound scanning | computed tomography scanning | abdominal ultrasound scanning | computed tomography scanning |  |
| **True positives** (patients with incisional hernia) | 2 studies 221 patients | cross-sectional (cohort type accuracy study) | not serious | not serious | not serious | serious^a^ | none^b^ | 426 to 588 | 0 to 0 | 388 to 536 | 0 to 0 | 0 to 0 | 0 to 0 | ⨁⨁⨁◯ Moderate |
|  |  |  |  |  |  |  |  | **426 more to 588 more TP in abdominal ultrasound scanning** | | **388 more to 536 more TP in abdominal ultrasound scanning** | | **0 fewer to 0 fewer TP in abdominal ultrasound scanning** | |  |
| **False negatives** (patients incorrectly classified as not having incisional hernia) |  |  |  |  |  |  |  | 12 to 174 | 600 to 600 | 11 to 159 | 547 to 547 | 0 to 0 | 0 to 0 |  |
|  |  |  |  |  |  |  |  | **426 fewer to 588 fewer FN in abdominal ultrasound scanning** | | **388 fewer to 536 fewer FN in abdominal ultrasound scanning** | | **0 fewer to 0 fewer FN in abdominal ultrasound scanning** | |  |
| **True negatives** (patients without incisional hernia) | 2 studies 221 patients | cross-sectional (cohort type accuracy study) | not serious | not serious | not serious | serious^a^ | none | 352 to 400 | 0 to 0 | 399 to 453 | 0 to 0 | 880 to 1000 | 0 to 0 | ⨁⨁⨁◯ Moderate |
|  |  |  |  |  |  |  |  | **352 more to 400 more TN in abdominal ultrasound scanning** | | **399 more to 453 more TN in abdominal ultrasound scanning** | | **880 more to 1000 more TN in abdominal ultrasound scanning** | |  |
| **False positives** (patients incorrectly classified as having incisional hernia) |  |  |  |  |  |  |  | 0 to 48 | 400 to 400 | 0 to 54 | 453 to 453 | 0 to 120 | 1000 to 1000 |  |
|  |  |  |  |  |  |  |  | **352 fewer to 400 fewer FP in abdominal ultrasound scanning** | | **399 fewer to 453 fewer FP in abdominal ultrasound scanning** | | **880 fewer to 1000 fewer FP in abdominal ultrasound scanning** | |  |

#### **Explanations**

a. Only two studies with total number of participants 221

b. Only two studies were identified in one systematic review with limited search
