# Supplementary material for: Midline incisional hernia guidelines: the European Hernia Society
Source: Br J Surg. 2023 Sep 19;110(12):1732–68. doi: 10.1093/bjs/znad284 (PMC10638550; doi:10.1093/bjs/znad284)
Supplement: znad284_Supplementary_Data [file znad284_supplementary_data.zip › Table_S4.docx]

**TABLE S4 SUMMARY OF FINDINGS FOR KQ3**

**Key Question 3: Is it possible to predict from imaging whether the fascial closure will be possible?**

.Is exposure to imaging methods helpful for predicting use of the facial closure technique in adults with incisional hernias?

| **Certainty assessment** | | | | | | | **№ of patients** | | **Effect** | | **Certainty** | **Importance** |
| --- | --- | --- | --- | --- | --- | --- | --- | --- | --- | --- | --- | --- |
| **№ of studies** | **Study design** | **Risk of bias** | **Inconsistency** | **Indirectness** | **Imprecision** | **Other considerations** | **fascial closure** | **no fascial closure** | **Relative (95% CI)** | **Absolute (95% CI)** |  |  |
| **CSI** | | | | | | | | | | | | |
| 2 | observational studies | not serious | not serious | not serious | serious^a^ | none | 108 | 36 | - | MD **0.07 lower** (0.08 lower to 0.05 lower) | ⨁◯◯◯ Very low | CRITICAL |
| **Defect width (cm)** | | | | | | | | | | | | |
| 2 | observational studies | not serious | not serious | not serious | serious^a^ | none | 138 | 24 | - | MD **3.39 lower** (4.99 lower to 1.8 lower) | ⨁◯◯◯ Very low | CRITICAL |
| **Defect lenght (cm)** | | | | | | | | | | | | |
| 2 | observational studies | not serious | not serious | not serious | serious^a^ | none | 138 | 24 | - | MD **3.82 lower** (6.53 lower to 1.12 lower) | ⨁◯◯◯ Very low | CRITICAL |
| **Defect surface area (cm)** | | | | | | | | | | | | |
| 2 | observational studies | not serious | not serious | not serious | serious^a^ | none | 138 | 24 | - | MD **78.21 lower** (137.51 lower to 18.91 lower) | ⨁◯◯◯ Very low | CRITICAL |

**CI:** confidence interval; **MD:** mean difference

#### Explanations

a. Just two studies with small sample size

**Question:** Myofascial release required compared to myofascial release not required for predicting successful fascial closure after progressive pre-operative pneumoperitoneum in adults with a midline incisional hernia

| **Certainty assessment** | | | | | | | **№ of patients** | | **Effect** | | **Certainty** | **Importance** |
| --- | --- | --- | --- | --- | --- | --- | --- | --- | --- | --- | --- | --- |
| **№ of studies** | **Study design** | **Risk of bias** | **Inconsistency** | **Indirectness** | **Imprecision** | **Other considerations** | **myofascial release required** | **myofascial release not required** | **Relative (95% CI)** | **Absolute (95% CI)** |  |  |
| **CSI** | | | | | | | | | | | | |
| 1 | observational studies | not serious | not serious | not serious | serious^a^ | none | 134 | 208 | - | MD **0.07 higher** (0.06 higher to 0.09 higher) | ⨁◯◯◯ Very low | CRITICAL |
| **Rectus defect ration** | | | | | | | | | | | | |
| 1 | observational studies | not serious | not serious | not serious | serious^a^ | none | 134 | 208 | - | MD **1.2 lower** (1.45 lower to 0.95 lower) | ⨁◯◯◯ Very low | CRITICAL |
| **Defect width (cm)** | | | | | | | | | | | | |
| 2 | observational studies | not serious | not serious | not serious | serious^a^ | none | 169 | 224 | - | MD **5.15 higher** (4.34 higher to 5.95 higher) | ⨁◯◯◯ Very low | CRITICAL |
| **Defect surface area (IHOA)** | | | | | | | | | | | | |
| 2 | observational studies | not serious | not serious | not serious | serious^a^ | none | 46 | 131 | - | MD **118.15 higher** (93.42 higher to 142.89 higher) | ⨁◯◯◯ Very low | CRITICAL |

**CI:** confidence interval; **MD:** mean difference

#### Explanations

a. Data just from one study
