# Supplementary material for: Midline incisional hernia guidelines: the European Hernia Society
Source: Br J Surg. 2023 Sep 19;110(12):1732–68. doi: 10.1093/bjs/znad284 (PMC10638550; doi:10.1093/bjs/znad284)
Supplement: znad284_Supplementary_Data [file znad284_supplementary_data.zip › Table_S5.docx]

**TABLE S5 SUMMARY OF FINDINGS FOR KQ4**

**Key Question 4:**

**b) What are the important outcome measures in treatment of incisional hernias?**

**Question:** Surgery approach compared to non-surgery approach in treatment of incisional hernias?

| **Certainty assessment** | | | | | | | **№ of patients** | | **Effect** | | **Certainty** | **Importance** |
| --- | --- | --- | --- | --- | --- | --- | --- | --- | --- | --- | --- | --- |
| **№ of studies** | **Study design** | **Risk of bias** | **Inconsistency** | **Indirectness** | **Imprecision** | **Other considerations** | **Surgery approach** | **non-surgery approach** | **Relative (95% CI)** | **Absolute (95% CI)** |  |  |
| **Physical functioning** | | | | | | | | | | | | |
| 1 | observational studies | not serious | serious^a^ | not serious | not serious | none | 121 | 74 | - | MD **9.1 higher** (1.64 higher to 16.56 higher) | ⨁◯◯◯ Very low | CRITICAL |
| **Role physical sum score** | | | | | | | | | | | | |
| 1 | observational studies | not serious | serious^a^ | not serious | not serious | none | 121 | 74 | - | MD **15.9 higher** (10.58 higher to 21.22 higher) | ⨁◯◯◯ Very low | CRITICAL |
| **Bodily pain sum score** | | | | | | | | | | | | |
| 1 | observational studies | not serious | serious^a^ | not serious | not serious | none | 121 | 74 | - | MD **11.5 higher** (3.75 higher to 19.25 higher) | ⨁◯◯◯ Very low | CRITICAL |
| **General health** | | | | | | | | | | | | |
| 1 | observational studies | not serious | serious^a^ | not serious | not serious | none | 121 | 74 | - | MD **3.7 higher** (18.62 lower to 26.02 higher) | ⨁◯◯◯ Very low | CRITICAL |
| **Vitality sum score** | | | | | | | | | | | | |
| 1 | observational studies | not serious | serious^a^ | not serious | not serious | none | 121 | 74 | - | MD **8 higher** (1 higher to 15 higher) | ⨁◯◯◯ Very low | CRITICAL |
| **Social functioning** | | | | | | | | | | | | |
| 1 | observational studies | not serious | serious^a^ | not serious | not serious | none | 121 | 74 | - | MD **9.3 higher** (0.56 higher to 18.04 higher) | ⨁◯◯◯ Very low | CRITICAL |
| **Role emotional sum score** | | | | | | | | | | | | |
| 1 | observational studies | not serious | serious^a^ | not serious | not serious | none | 121 | 74 | - | MD **9.2 higher** (0.18 higher to 18.22 higher) | ⨁◯◯◯ Very low | CRITICAL |
| **Mental health sum score** | | | | | | | | | | | | |
| 1 | observational studies | not serious | serious^a^ | not serious | not serious | none | 121 | 74 | - | MD **1.7 higher** (1.13 lower to 4.53 higher) | ⨁◯◯◯ Very low | CRITICAL |
