# Supplementary material for: Midline incisional hernia guidelines: the European Hernia Society
Source: Br J Surg. 2023 Sep 19;110(12):1732–68. doi: 10.1093/bjs/znad284 (PMC10638550; doi:10.1093/bjs/znad284)
Supplement: znad284_Supplementary_Data [file znad284_supplementary_data.zip › Table_S6.docx]

**TABLE S7: SUMMARY OF FINDINGS FOR KQ6**

**Key Question 6: What is the difference in outcome for mesh versus suture repair in incisional hernia repair?**

**Question:** Mesh compared to tissue repair with sutures for the elective surgical treatment of incisional hernias in adult patients

| **Certainty assessment** | | | | | | | **№ of patients** | | **Effect** | | **Certainty** | **Importance** |
| --- | --- | --- | --- | --- | --- | --- | --- | --- | --- | --- | --- | --- |
| **№ of studies** | **Study design** | **Risk of bias** | **Inconsistency** | **Indirectness** | **Imprecision** | **Other considerations** | **mesh** | **tissue repair with sutures** | **Relative (95% CI)** | **Absolute (95% CI)** |  |  |
| **Recurrence** | | | | | | | | | | | | |
| 5 | randomised trials | very serious^a^ | not serious | not serious | not serious | none | 58/490 (11.8%) | 135/444 (30.4%) | **OR 0.31** (0.21 to 0.44) | **185 fewer per 1 000** (from 220 fewer to 143 fewer) | ⨁⨁◯◯ Low | CRITICAL |
| **Recurrence - Mesh onlay (polypropylene)** | | | | | | | | | | | | |
| 3 | randomised trials | very serious^a^ | not serious | not serious | serious^b^ | none | 9/124 (7.3%) | 19/113 (16.8%) | **OR 0.39** (0.17 to 0.90) | **95 fewer per 1 000** (from 135 fewer to 14 fewer) | ⨁◯◯◯ Very low | CRITICAL |
| **Recurrence - Mesh sublay (polypropylene)** | | | | | | | | | | | | |
| 3 | randomised trials | very serious^a^ | not serious | not serious | not serious | none | 49/366 (13.4%) | 116/331 (35.0%) | **OR 0.29** (0.20 to 0.43) | **215 fewer per 1 000** (from 253 fewer to 162 fewer) | ⨁⨁◯◯ Low | CRITICAL |
| **Infection** | | | | | | | | | | | | |
| 2 | randomised trials | very serious^a^ | not serious | not serious | very serious^b^ | none | 6/71 (8.5%) | 5/63 (7.9%) | **OR 1.07** (0.33 to 3.49) | **5 more per 1 000** (from 52 fewer to 152 more) | ⨁◯◯◯ Very low | CRITICAL |
| **Infection - Mesh onlay (polypropylene)** | | | | | | | | | | | | |
| 2 | randomised trials | very serious^a^ | not serious | not serious | very serious^b^ | none | 6/71 (8.5%) | 5/63 (7.9%) | **OR 1.07** (0.33 to 3.49) | **5 more per 1 000** (from 52 fewer to 152 more) | ⨁◯◯◯ Very low | CRITICAL |
| **Infection - Mesh sublay (polypropylene)** | | | | | | | | | | | | |
| 0 |  |  |  |  |  |  | 0/0 | 0/0 | not pooled | see comment | - | CRITICAL |
| **Hematoma** | | | | | | | | | | | | |
| 3 | randomised trials | very serious^a^ | not serious | not serious | very serious^b^ | none | 0/226 (0.0%) | 13/163 (8.0%) | **OR 0.10** (0.02 to 0.43) | **71 fewer per 1 000** (from 78 fewer to 44 fewer) | ⨁◯◯◯ Very low | CRITICAL |
| **Hematoma - Mesh onlay (polypropylene)** | | | | | | | | | | | | |
| 3 | randomised trials | very serious^a^ | not serious | not serious | very serious^b^ | none | 0/124 (0.0%) | 10/113 (8.8%) | **OR 0.11** (0.02 to 0.60) | **78 fewer per 1 000** (from 87 fewer to 33 fewer) | ⨁◯◯◯ Very low | CRITICAL |
| **Hematoma - Mesh sublay (polypropylene)** | | | | | | | | | | | | |
| 1 | randomised trials | very serious^a^ | not serious | not serious | very serious^c^ | none | 0/102 (0.0%) | 3/50 (6.0%) | **OR 0.07** (0.00 to 1.31) | **56 fewer per 1 000** (from -- to 17 more) | ⨁◯◯◯ Very low | CRITICAL |
| **Seroma** | | | | | | | | | | | | |
| 3 | randomised trials | very serious^a^ | not serious | not serious | very serious^c^ | none | 43/226 (19.0%) | 11/163 (6.7%) | **OR 3.48** (1.75 to 6.93) | **134 more per 1 000** (from 45 more to 267 more) | ⨁◯◯◯ Very low | CRITICAL |
| **Seroma - Mesh onlay (polypropylene)** | | | | | | | | | | | | |
| 3 | randomised trials | very serious^a^ | not serious | not serious | very serious^c^ | none | 31/124 (25.0%) | 6/113 (5.3%) | **OR 6.78** (2.69 to 17.10) | **222 more per 1 000** (from 78 more to 436 more) | ⨁◯◯◯ Very low | CRITICAL |
| **Seroma - Mesh sublay (polypropylene)** | | | | | | | | | | | | |
| 1 | randomised trials | very serious^a^ | not serious | not serious | very serious^c^ | none | 12/102 (11.8%) | 5/50 (10.0%) | **OR 1.20** (0.40 to 3.62) | **18 more per 1 000** (from 57 fewer to 187 more) | ⨁◯◯◯ Very low | CRITICAL |
| **Lenght of stay** | | | | | | | | | | | | |
| 3 | randomised trials | very serious^a^ | not serious | not serious | serious^b^ | none | 226 | 163 | - | MD **1.08 higher** (0.53 higher to 1.63 higher) | ⨁◯◯◯ Very low | IMPORTANT |
| **Lenght of stay - Mesh onlay (polypropylene)** | | | | | | | | | | | | |
| 3 | randomised trials | very serious^a^ | not serious | not serious | serious^b^ | none | 124 | 113 | - | MD **1.55 higher** (0.83 higher to 2.27 higher) | ⨁◯◯◯ Very low | IMPORTANT |
| **Lenght of stay - Mesh sublay (polypropylene)** | | | | | | | | | | | | |
| 1 | randomised trials | very serious^a^ | not serious | not serious | serious^b^ | none | 102 | 50 | - | MD **0.4 higher** (0.46 lower to 1.26 higher) | ⨁◯◯◯ Very low | IMPORTANT |

**CI:** confidence interval; **MD:** mean difference; **OR:** odds ratio

#### Explanations

a. Most of the bias domains are unclear across all studies

b. Very small sample size

c. Small sample size and wide CI
