# Supplementary material for: Midline incisional hernia guidelines: the European Hernia Society
Source: Br J Surg. 2023 Sep 19;110(12):1732–68. doi: 10.1093/bjs/znad284 (PMC10638550; doi:10.1093/bjs/znad284)
Supplement: znad284_Supplementary_Data [file znad284_supplementary_data.zip › Table_S7.docx]

**TABLE S8: SUMMARY OF FINDINGS FOR KQ7**

**Key Question 7: What is the difference in outcome considering different positions of mesh in incisional hernia repair?**

**Question:** Onlay mesh position compared to retro muscular (sublay) mesh position for the elective surgical repair of incisional hernia in adult patients

| **Certainty assessment** | | | | | | | **№ of patients** | | **Effect** | | **Certainty** | **Importance** |
| --- | --- | --- | --- | --- | --- | --- | --- | --- | --- | --- | --- | --- |
| **№ of studies** | **Study design** | **Risk of bias** | **Inconsistency** | **Indirectness** | **Imprecision** | **Other considerations** | **onlay mesh position** | **retro muscular (sublay) mesh position** | **Relative (95% CI)** | **Absolute (95% CI)** |  |  |
| **Reccurence** | | | | | | | | | | | | |
| 4 | randomised trials | serious^a^ | not serious | not serious | very serious^b^ | none | 14/194 (7.2%) | 4/187 (2.1%) | **OR 3.24** (1.10 to 9.52) | **45 more per 1 000** (from 2 more to 151 more) | ⨁◯◯◯ Very low | CRITICAL |
| **Seroma** | | | | | | | | | | | | |
| 4 | randomised trials | serious^a^ | not serious | not serious | serious^c^ | none | 66/198 (33.3%) | 26/188 (13.8%) | **OR 3.32** (1.96 to 5.62) | **209 more per 1 000** (from 101 more to 336 more) | ⨁⨁◯◯ Low | CRITICAL |
| **Lenght of stay** | | | | | | | | | | | | |
| 3 | randomised trials | serious^a^ | not serious | not serious | serious^d^ | none | 181 | 176 | - | MD **0.21 higher** (0.26 lower to 0.68 higher) | ⨁⨁◯◯ Low | CRITICAL |
| **Hematoma** | | | | | | | | | | | | |
| 3 | randomised trials | serious^a^ | not serious | not serious | extremely serious^c^ | none | 5/185 (2.7%) | 3/177 (1.7%) | **OR 1.68** (0.39 to 7.17) | **11 more per 1 000** (from 10 fewer to 93 more) | ⨁◯◯◯ Very low | CRITICAL |

**CI:** confidence interval; **MD:** mean difference; **OR:** odds ratio

#### Explanations

a. RoB across all included studies is serious, Randomization, Allocation concealment and blinding are unclear

b. Very small studies are included with very small number of events

c. Very small studies are included with small number of events

d. CI is wide

**Question:** Onlay mesh compared to intraperitoneal-mesh (open IPOM) for incisional hernia

| **Certainty assessment** | | | | | | | **№ of patients** | | **Effect** | | **Certainty** | **Importance** |
| --- | --- | --- | --- | --- | --- | --- | --- | --- | --- | --- | --- | --- |
| **№ of studies** | **Study design** | **Risk of bias** | **Inconsistency** | **Indirectness** | **Imprecision** | **Other considerations** | **onlay mesh** | **intraperitoneal-mesh (open IPOM)** | **Relative (95% CI)** | **Absolute (95% CI)** |  |  |
| **Seroma** | | | | | | | | | | | | |
| 1 | randomised trials | not serious | not serious | not serious | extremely serious^a^ | none | 7/22 (31.8%) | 0/19 (0.0%) | **OR 18.87** (1.00 to 356.74) | **0 fewer per 1 000** (from 0 fewer to 0 fewer) | ⨁◯◯◯ Very low | CRITICAL |
| **SSI** | | | | | | | | | | | | |
| 1 | randomised trials | not serious | not serious | not serious | extremely serious^a^ | none | 1/22 (4.5%) | 1/19 (5.3%) | **OR 0.86** (0.05 to 14.71) | **7 fewer per 1 000** (from 50 fewer to 397 more) | ⨁◯◯◯ Very low | CRITICAL |
| **Recurence after 1 year** | | | | | | | | | | | | |
| 1 | randomised trials | not serious | not serious | not serious | extremely serious^a^ | none | 6/22 (27.3%) | 0/19 (0.0%) | **OR 15.36** (0.80 to 293.60) | **0 fewer per 1 000** (from 0 fewer to 0 fewer) | ⨁◯◯◯ Very low | CRITICAL |
| **Relevant pain** | | | | | | | | | | | | |
| 1 | randomised trials | not serious | not serious | not serious | extremely serious^a^ | none | 1/22 (4.5%) | 6/19 (31.6%) | **OR 0.10** (0.01 to 0.96) | **272 fewer per 1 000** (from 311 fewer to 9 fewer) | ⨁◯◯◯ Very low | CRITICAL |

**CI:** confidence interval; **OR:** odds ratio

#### Explanations

a. Very small single study with huge CI

**Question:** Minimal invasive sublay (MILOS) compared to minimal invasive intraperitoneal mesh (lap. IPOM) for incisional hernia repair

| **Certainty assessment** | | | | | | | **№ of patients** | | **Effect** | | **Certainty** | **Importance** |
| --- | --- | --- | --- | --- | --- | --- | --- | --- | --- | --- | --- | --- |
| **№ of studies** | **Study design** | **Risk of bias** | **Inconsistency** | **Indirectness** | **Imprecision** | **Other considerations** | **minimal invasive sublay (MILOS)** | **minimal invasive intraperitoneal mesh (lap. IPOM)** | **Relative (95% CI)** | **Absolute (95% CI)** |  |  |
| **Seroma** | | | | | | | | | | | | |
| 1 | observational studies | not serious | not serious | not serious | serious^a^ | none | 3/541 (0.6%) | 18/541 (3.3%) | **OR 0.16** (0.05 to 0.55) | **28 fewer per 1 000** (from 32 fewer to 15 fewer) | ⨁◯◯◯ Very low | CRITICAL |
| **General complications** | | | | | | | | | | | | |
| 1 | observational studies | not serious | not serious | not serious | serious^a^ | none | 6/541 (1.1%) | 22/541 (4.1%) | **OR 0.26** (0.11 to 0.66) | **30 fewer per 1 000** (from 36 fewer to 13 fewer) | ⨁◯◯◯ Very low | CRITICAL |
| **SSO** | | | | | | | | | | | | |
| 1 | observational studies | not serious | not serious | not serious | serious^a^ | none | 7/541 (1.3%) | 31/541 (5.7%) | **OR 0.22** (0.09 to 0.49) | **44 fewer per 1 000** (from 52 fewer to 28 fewer) | ⨁◯◯◯ Very low | CRITICAL |
| **Hematoma** | | | | | | | | | | | | |
| 1 | observational studies | not serious | not serious | not serious | serious^a^ | none | 3/541 (0.6%) | 9/541 (1.7%) | **OR 0.33** (0.09 to 1.22) | **11 fewer per 1 000** (from 15 fewer to 4 more) | ⨁◯◯◯ Very low | CRITICAL |
| **Recurrence** | | | | | | | | | | | | |
| 1 | observational studies | not serious | not serious | not serious | serious^a^ | none | 10/463 (2.2%) | 34/463 (7.3%) | **OR 0.28** (0.14 to 0.57) | **52 fewer per 1 000** (from 62 fewer to 30 fewer) | ⨁◯◯◯ Very low | CRITICAL |
| **Chronic pain during activity** | | | | | | | | | | | | |
| 1 | observational studies | not serious | not serious | not serious | serious^a^ | none | 25/463 (5.4%) | 115/463 (24.8%) | **OR 0.17** (0.11 to 0.27) | **195 fewer per 1 000** (from 213 fewer to 166 fewer) | ⨁◯◯◯ Very low | CRITICAL |
| **Chronic pain requiring treatment** | | | | | | | | | | | | |
| 1 | observational studies | not serious | not serious | not serious | serious^a^ | none | 12/463 (2.6%) | 42/463 (9.1%) | **OR 0.27** (0.14 to 0.51) | **64 fewer per 1 000** (from 77 fewer to 42 fewer) | ⨁◯◯◯ Very low | CRITICAL |
| **Chronic pain at rest after 1 year** | | | | | | | | | | | | |
| 1 | observational studies | not serious | not serious | not serious | serious^a^ | none | 17/463 (3.7%) | 65/463 (14.0%) | **OR 0.23** (0.13 to 0.40) | **104 fewer per 1 000** (from 120 fewer to 79 fewer) | ⨁◯◯◯ Very low | CRITICAL |

**CI:** confidence interval; **OR:** odds ratio

#### Explanations

a. Just one study small number of events
