# Supplementary material for: Midline incisional hernia guidelines: the European Hernia Society
Source: Br J Surg. 2023 Sep 19;110(12):1732–68. doi: 10.1093/bjs/znad284 (PMC10638550; doi:10.1093/bjs/znad284)
Supplement: znad284_Supplementary_Data [file znad284_supplementary_data.zip › Table_S8.docx]

**TABLE S9: SUMMARY OF FINDINGS FOR KQ8**

**Key Question 8: What is the difference in outcome between techniques (open, laparoscopic and robotic) for incisional hernia repair?**

**Question:** Open surgery compared to laparoscopy surgery for the elective surgical repair of incisional hernia in adult patients

| **Certainty assessment** | | | | | | | **№ of patients** | | **Effect** | | **Certainty** | **Importance** |
| --- | --- | --- | --- | --- | --- | --- | --- | --- | --- | --- | --- | --- |
| **№ of studies** | **Study design** | **Risk of bias** | **Inconsistency** | **Indirectness** | **Imprecision** | **Other considerations** | **open surgery** | **laparoscopy surgery** | **Relative (95% CI)** | **Absolute (95% CI)** |  |  |
| **Recurrence** | | | | | | | | | | | | |
| 3 | randomised trials | serious^a^ | not serious | not serious | serious^b^ | none | 16/246 (6.5%) | 24/242 (9.9%) | **OR 0.62** (0.31 to 1.25) | **35 fewer per 1 000** (from 66 fewer to 22 more) | ⨁⨁◯◯ Low | CRITICAL |
| **Surgical site infection** | | | | | | | | | | | | |
| 5 | randomised trials | serious^a^ | serious^c^ | not serious | serious^b^ | none | 30/277 (10.8%) | 8/261 (3.1%) | **OR 2.68** (0.58 to 12.31) | **47 more per 1 000** (from 13 fewer to 250 more) | ⨁◯◯◯ Very low | CRITICAL |
| **Deep infection** | | | | | | | | | | | | |
| 5 | randomised trials | serious^a^ | not serious | not serious | very serious^d^ | none | 5/277 (1.8%) | 4/261 (1.5%) | **OR 1.07** (0.30 to 3.83) | **1 more per 1 000** (from 11 fewer to 41 more) | ⨁◯◯◯ Very low | CRITICAL |
| **Laparotomy** | | | | | | | | | | | | |
| 5 | randomised trials | serious^a^ | not serious | not serious | serious^b^ | none | 9/277 (3.2%) | 12/261 (4.6%) | **OR 0.69** (0.28 to 1.66) | **14 fewer per 1 000** (from 33 fewer to 28 more) | ⨁⨁◯◯ Low | CRITICAL |

**CI:** confidence interval; **OR:** odds ratio

#### Explanations

a. RoB across all included studies is serious, Randomization, Allocation concealment and blinding are high or unclear

b. Small number of events

c. Unexplained statistical heterogeneity,

d. Very low number of events and wide confidence interval, unclear benefit or harm

**Question:** Laparoscopic surgery compared to robotic surgery for elective surgical repair of incisional hernia in adult patients

| **Certainty assessment** | | | | | | | **№ of patients** | | **Effect** | | **Certainty** | **Importance** |
| --- | --- | --- | --- | --- | --- | --- | --- | --- | --- | --- | --- | --- |
| **№ of studies** | **Study design** | **Risk of bias** | **Inconsistency** | **Indirectness** | **Imprecision** | **Other considerations** | **laparoscopic surgery** | **robotic surgery** | **Relative (95% CI)** | **Absolute (95% CI)** |  |  |
| **Recurrence** | | | | | | | | | | | | |
| 1 | randomised trials | not serious | not serious | not serious | extremely serious^a^ | none | 5/59 (8.5%) | 4/65 (6.2%) | **OR 1.41** (0.36 to 5.53) | **23 more per 1 000** (from 38 fewer to 205 more) | ⨁◯◯◯ Very low | CRITICAL |

**CI:** confidence interval; **OR:** odds ratio

#### Explanations

a. Small number of events, one study

**Question:** Onlay mesh compared to intraperitoneal-mesh (open IPOM) for incisional hernia

| **Certainty assessment** | | | | | | | **№ of patients** | | **Effect** | | **Certainty** | **Importance** |
| --- | --- | --- | --- | --- | --- | --- | --- | --- | --- | --- | --- | --- |
| **№ of studies** | **Study design** | **Risk of bias** | **Inconsistency** | **Indirectness** | **Imprecision** | **Other considerations** | **onlay mesh** | **intraperitoneal-mesh (open IPOM)** | **Relative (95% CI)** | **Absolute (95% CI)** |  |  |
| **Seroma** | | | | | | | | | | | | |
| 1 | randomised trials | not serious | not serious | not serious | extremely serious^a^ | none | 7/22 (31.8%) | 0/19 (0.0%) | **OR 18.87** (1.00 to 356.74) | **0 fewer per 1 000** (from 0 fewer to 0 fewer) | ⨁◯◯◯ Very low | CRITICAL |
| **SSI** | | | | | | | | | | | | |
| 1 | randomised trials | not serious | not serious | not serious | extremely serious^a^ | none | 1/22 (4.5%) | 1/19 (5.3%) | **OR 0.86** (0.05 to 14.71) | **7 fewer per 1 000** (from 50 fewer to 397 more) | ⨁◯◯◯ Very low | CRITICAL |
| **Recurence after 1 year** | | | | | | | | | | | | |
| 1 | randomised trials | not serious | not serious | not serious | extremely serious^a^ | none | 6/22 (27.3%) | 0/19 (0.0%) | **OR 15.36** (0.80 to 293.60) | **0 fewer per 1 000** (from 0 fewer to 0 fewer) | ⨁◯◯◯ Very low | CRITICAL |
| **Relevant pain** | | | | | | | | | | | | |
| 1 | randomised trials | not serious | not serious | not serious | extremely serious^a^ | none | 1/22 (4.5%) | 6/19 (31.6%) | **OR 0.10** (0.01 to 0.96) | **272 fewer per 1 000** (from 311 fewer to 9 fewer) | ⨁◯◯◯ Very low | CRITICAL |

**CI:** confidence interval; **OR:** odds ratio

#### Explanations

a. Very small single study with huge CI

**Question:** Minimal invasive sublay (MILOS) compared to minimal invasive intraperitoneal mesh (lap. IPOM) for incisional hernia repair

| **Certainty assessment** | | | | | | | **№ of patients** | | **Effect** | | **Certainty** | **Importance** |
| --- | --- | --- | --- | --- | --- | --- | --- | --- | --- | --- | --- | --- |
| **№ of studies** | **Study design** | **Risk of bias** | **Inconsistency** | **Indirectness** | **Imprecision** | **Other considerations** | **minimal invasive sublay (MILOS)** | **minimal invasive intraperitoneal mesh (lap. IPOM)** | **Relative (95% CI)** | **Absolute (95% CI)** |  |  |
| **Seroma** | | | | | | | | | | | | |
| 1 | observational studies | not serious | not serious | not serious | serious^a^ | none | 3/541 (0.6%) | 18/541 (3.3%) | **OR 0.16** (0.05 to 0.55) | **28 fewer per 1 000** (from 32 fewer to 15 fewer) | ⨁◯◯◯ Very low | CRITICAL |
| **General complications** | | | | | | | | | | | | |
| 1 | observational studies | not serious | not serious | not serious | serious^a^ | none | 6/541 (1.1%) | 22/541 (4.1%) | **OR 0.26** (0.11 to 0.66) | **30 fewer per 1 000** (from 36 fewer to 13 fewer) | ⨁◯◯◯ Very low | CRITICAL |
| **SSO** | | | | | | | | | | | | |
| 1 | observational studies | not serious | not serious | not serious | serious^a^ | none | 7/541 (1.3%) | 31/541 (5.7%) | **OR 0.22** (0.09 to 0.49) | **44 fewer per 1 000** (from 52 fewer to 28 fewer) | ⨁◯◯◯ Very low | CRITICAL |
| **Hematoma** | | | | | | | | | | | | |
| 1 | observational studies | not serious | not serious | not serious | serious^a^ | none | 3/541 (0.6%) | 9/541 (1.7%) | **OR 0.33** (0.09 to 1.22) | **11 fewer per 1 000** (from 15 fewer to 4 more) | ⨁◯◯◯ Very low | CRITICAL |
| **Recurrence** | | | | | | | | | | | | |
| 1 | observational studies | not serious | not serious | not serious | serious^a^ | none | 10/463 (2.2%) | 34/463 (7.3%) | **OR 0.28** (0.14 to 0.57) | **52 fewer per 1 000** (from 62 fewer to 30 fewer) | ⨁◯◯◯ Very low | CRITICAL |
| **Chronic pain during activity** | | | | | | | | | | | | |
| 1 | observational studies | not serious | not serious | not serious | serious^a^ | none | 25/463 (5.4%) | 115/463 (24.8%) | **OR 0.17** (0.11 to 0.27) | **195 fewer per 1 000** (from 213 fewer to 166 fewer) | ⨁◯◯◯ Very low | CRITICAL |
| **Chronic pain requiring treatment** | | | | | | | | | | | | |
| 1 | observational studies | not serious | not serious | not serious | serious^a^ | none | 12/463 (2.6%) | 42/463 (9.1%) | **OR 0.27** (0.14 to 0.51) | **64 fewer per 1 000** (from 77 fewer to 42 fewer) | ⨁◯◯◯ Very low | CRITICAL |
| **Chronic pain at rest after 1 year** | | | | | | | | | | | | |
| 1 | observational studies | not serious | not serious | not serious | serious^a^ | none | 17/463 (3.7%) | 65/463 (14.0%) | **OR 0.23** (0.13 to 0.40) | **104 fewer per 1 000** (from 120 fewer to 79 fewer) | ⨁◯◯◯ Very low | CRITICAL |

**CI:** confidence interval; **OR:** odds ratio

#### Explanations

a. just one study small number of events
