# Supplementary material for: Midline incisional hernia guidelines: the European Hernia Society
Source: Br J Surg. 2023 Sep 19;110(12):1732–68. doi: 10.1093/bjs/znad284 (PMC10638550; doi:10.1093/bjs/znad284)
Supplement: znad284_Supplementary_Data [file znad284_supplementary_data.zip › Table_S9.docx]

**TABLE S10: SUMMARY OF FINDINGS FOR KQ9**

**Key Question 9:**

**Is there a benefit of primary fascial closure in midline incisional hernia mesh repair?**

**Question:** Fascial closure compared to bridging for the elective surgical repair of incisional hernia in adult patients

| **Certainty assessment** | | | | | | | **№ of patients** | | **Effect** | | **Certainty** | **Importance** |
| --- | --- | --- | --- | --- | --- | --- | --- | --- | --- | --- | --- | --- |
| **№ of studies** | **Study design** | **Risk of bias** | **Inconsistency** | **Indirectness** | **Imprecision** | **Other considerations** | **fascial closure** | **bridging** | **Relative (95% CI)** | **Absolute (95% CI)** |  |  |
| **Recurrence** | | | | | | | | | | | | |
| 3 | randomised trials | not serious | not serious | not serious | very serious^a^ | none | 7/168 (4.2%) | 12/177 (6.8%) | **OR 0.60** (0.23 to 1.57) | **26 fewer per 1 000** (from 51 fewer to 35 more) | ⨁⨁◯◯ Low | CRITICAL |
| **QoL (mAAS Scale) difference from baseline after 2 years** | | | | | | | | | | | | |
| 1 | randomised trials | not serious | not serious | not serious | very serious^b^ | none | 64 | 65 | - | MD **11.6 higher** (1.2 higher to 22 higher) | ⨁⨁◯◯ Low | CRITICAL |
| **Morbidity (Hematoma, Seroma)** | | | | | | | | | | | | |
| 3 | randomised trials | not serious | not serious | not serious | very serious^c^ | none | 14/228 (6.1%) | 27/239 (11.3%) | **OR 0.52** (0.27 to 1.02) | **51 fewer per 1 000** (from 80 fewer to 2 more) | ⨁⨁◯◯ Low | CRITICAL |
| **Morbidity (Hematoma, Seroma) - Hematoma** | | | | | | | | | | | | |
| 1 | randomised trials | not serious | not serious | not serious | extremely serious^b^ | none | 0/61 (0.0%) | 3/62 (4.8%) | **OR 0.14** (0.01 to 2.73) | **41 fewer per 1 000** (from 48 fewer to 74 more) | ⨁◯◯◯ Very low | CRITICAL |
| **Morbidity (Hematoma, Seroma) - Seroma** | | | | | | | | | | | | |
| 2 | randomised trials | not serious | not serious | not serious | very serious^c^ | none | 9/85 (10.6%) | 12/87 (13.8%) | **OR 0.75** (0.30 to 1.84) | **31 fewer per 1 000** (from 92 fewer to 90 more) | ⨁⨁◯◯ Low | CRITICAL |
| **Morbidity (Hematoma, Seroma) - Hematoma and Seroma combined** | | | | | | | | | | | | |
| 1 | randomised trials | not serious | not serious | not serious | extremely serious^b^ | none | 5/82 (6.1%) | 12/90 (13.3%) | **OR 0.42** (0.14 to 1.26) | **73 fewer per 1 000** (from 112 fewer to 29 more) | ⨁◯◯◯ Very low | CRITICAL |
| **Pain (VAS) chronic 6 - 24 months** | | | | | | | | | | | | |
| 3 | randomised trials | not serious | serious^d^ | not serious | serious^e^ | none | 161 | 168 | - | MD **0.01 higher** (0.32 lower to 0.33 higher) | ⨁⨁◯◯ Low | CRITICAL |

**CI:** confidence interval; **MD:** mean difference; **OR:** odds ratio

#### Explanations

a. Just 15 events total

b. Just one study with smaller sample size

c. For Hematoma and Seroma sub groups, there are very small samples and wide CI

d. Significant statistical heterogeneity

e. Small number of studies and participants
